# Molecular diffusion enhanced performance evaluation of metal-organic frameworks for CO_2_ capture

**DOI:** 10.1038/s41467-026-73904-5

**Published:** 2026-06-17

**Authors:** Rodrigo Neumann Barros Ferreira, Yogesh V. Joshi, Felipe Lopes Oliveira, Anantha Sundaram, Ashish B. Mhadeshwar, Jayashree Kalyanaraman, Binquan Luan, Mathias B. Steiner

**Affiliations:** 1 IBM Research, Rio de Janeiro, RJ Brazil; 2 ExxonMobil Technology and Engineering Company, Annadale, NJ USA; 3 Instituto de Química, Universidade Federal do Rio de Janeiro, Rio de Janeiro, Brazil; 4 IBM Research, Yorktown Heights, NY USA

**Keywords:** Metal-organic frameworks, Porous materials, Chemical engineering, Computational methods

## Abstract

Molecular diffusion is a fundamental property that limits the performance of solid sorbents in carbon dioxide capture and separation applications. Unique to each sorbent, gas diffusion is determined by the physical and chemical interactions that occur between the gas molecules and a sorbent’s surface atoms. At the process level where carbon dioxide capture performance is validated, however, simulations are typically carried out using generalized parameters that omit the structure-specific, molecular kinetics occurring in each sorbent. Here, we report process-scale simulations of carbon dioxide capture performance in metal-organic frameworks (MOF) informed by molecular adsorption predictions that represent the unique structural properties of each MOF. By evaluating a total of 10,143 MOFs for post-combustion carbon dioxide capture, we demonstrate how the inclusion of the material-specific, molecular diffusion dynamics alters their simulated, process-level performance. The method could be applied to evaluate a broader class of solid sorbents, including covalent organic frameworks and zeolites.

## Abstract (web summary)

Metal-organic frameworks are candidates for carbon capture. Here the authors introduce a computational workflow to investigate their CO_2_ capture performance considering molecular diffusivity and identify promising water-tolerant systems.

## Introduction

Point-source capture of carbon dioxide (CO_2_) is a key feature of net-zero emission strategies^1–3^. Solid sorbents, such as, for example, activated carbons, zeolites, and covalent organic frameworks have been proposed as potentially high-performing CO_2_ capture materials for industrial applications^4^. Among the candidate materials, metal-organic frameworks (MOF) stand out due to their large chemical variability enabled by their building blocks, i.e., organic linkers and metal clusters^5^. While there exist trillions of MOF descriptions^6^, merely thousands of those structures have been realized and validated in the lab so far^7^. Therefore, analyzing and rank-ordering all known MOF structures with regards to their application performance remains a major challenge, even by using advanced computational methods^8,9^.

Computational screening workflows typically include calculations of geometric and adsorption properties of the specific MOF candidates to be evaluated, however, the predictions are often limited to the molecular scale^10–14^. Wilmer et al.^15^ performed a large-scale evaluation of MOFs for CH_4_ capture based solely on molecular-level adsorption uptake predictions. Similarly, Lin et al.^16^ derived a metric from molecular-level data for assessing and rank zeolites and zeolitic imidazolate frameworks (ZIF) for CO_2_ capture. While indicative of the potential at process scale, individual molecular-level metrics have yet failed to predict process-level material performance overall^17^.

Nevertheless, the application-specific gas separation performance of any given material must ultimately be assessed at the process level, by taking into consideration realistic figures-of-merits for purity, productivity, recovery and energy consumption^18^. It is, therefore, necessary to devise a multi-scale screening approach for integrating the molecular-to-process scale performance evaluation^19^.

Recent studies have provided molecular-level simulation data as inputs to macro-scale process models, however, the approach poses challenges^20^. Specifically, the CO_2_ uptake of a MOF structure, simulated at molecular scale either by means of Grand Canonical Monte Carlo (GCMC) or Ideal Adsorbed Solution Theory (IAST), is used as input for pressure- or vacuum-swing adsorption (PSA/VSA) models at process scale that comply with the 95% CO_2_-purity and 90% CO_2_-recovery targets, respectively, issued by the the U.S. Department of Energy^21–26^. Burns et al.^24^ integrated atomistic simulations with a VSA process simulator while Farmahini et al.^25^ studied the effect of mesoscale materials properties, such as size and porosity of the material constituents, on productivity and energy efficiency of the VSA process. These approaches, however, were based on the assumption that gas adsorption in MOFs does not depend on structure-dependent differences that occur at microscopic scales. Conceptually, this was reflected by assuming a constant mass transfer coefficient for all MOF candidates under investigation.

**Fig. 1:**
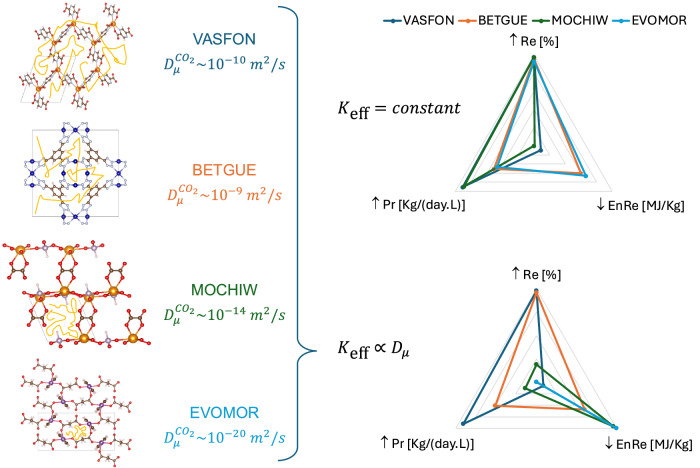
Impact of molecular diffusion in MOF on process performance in post-combustion CO_2_ capture. Four representative MOF structures are shown on the left. The inclusion of their material-specific, micro-pore diffusivity *D*_*μ*_ in the effective mass transfer coefficient *K*_eff_, see Equation (2), leads to significant changes in CO_2_ capture outcomes at process level: Recovery (higher is preferable), Productivity (higher is preferable) and Energy Requirement (lower is preferable).

An enhanced performance assessment at process level would require an effective mass transfer coefficient *K*_eff_ that captures both structure-independent, macro-pore diffusivity, as well as structure-dependent, micro-pore diffusivity^27^. Indeed, several investigations^28,29^ have already revealed a dependence of diffusivity on micro-pore, molecular loading. As gas diffusion and adsorption are, ultimately, determined by a MOF’s structural properties, we suggest including micro-pore gas diffusivity, provided for each MOF structure through MD simulations, into the process simulation of CO_2_ capture performance by means of *K*_eff_, as conceptually visualized in Fig. 1.

**Fig. 2:**
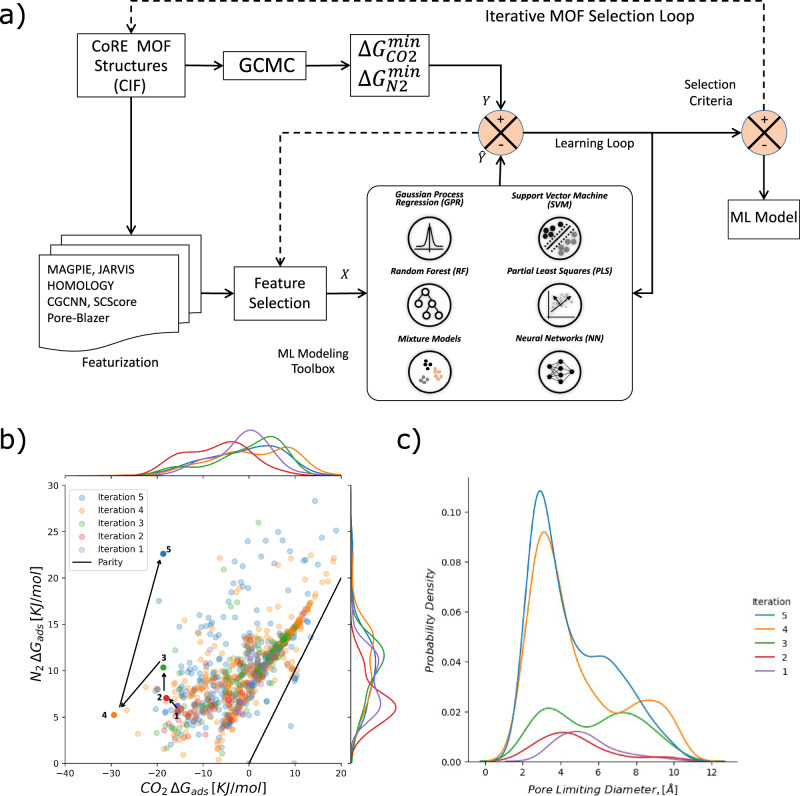
Active learning model for MOF selection. **a** Model architecture with iterative material selection workflow that targets CO_2_/N_2_ selectivity and CO_2_ capacity. **b** Evolution of 5 successive model iterations and the associated MOF distributions in terms of CO_2_ and N_2_ adsorption free energy, respectively. The highest-ranked material in each iteration exhibits a significant increase of CO_2_/N_2_ selectivity with regards to prior iterations. **c** Pore-limiting diameter distribution of selected MOFs for each of the 5 active-learning iterations. Source data are provided as a Source Data file.

In the following, we introduce a multi-scale, computational workflow to evaluate MOFs for CO_2_ post-combustion capture with temperature-swing absorption (TSA) in natural gas combined cycle (NGCC) power plants. The workflow, see Fig. 2a, uses molecular-level simulations for characterizing MOFs with high CO_2_ adsorption selectivity, which are shortlisted based on active learning cycles, and rank-ordered based on process simulations informed by molecular-scale, adsorption figures-of-merit. Finally, process optimizations at realistic operation conditions confirm each MOF’s performance at process level. We demonstrate that inclusion of molecular kinetics improves the workflow’s ability to discover false leads, which reduces computational costs significantly. Moreover, based on the top-20 MOF ranking results, we show for representative crystallite diameters how the inclusion of microscopic diffusivity affects process-level, CO_2_ capture performance predictions.

## Results

In the following sections, we report the results obtained by applying the computational workflow that integrates structure selection by active-learning, molecular simulation and process-level optimization. As reference database for our study, we have selected the non-disordered, all solvent removed (ASR) portion of CoRE-MOF-2019 (v1.1.2)^30,31^. From the initial dataset of 10,143 structures, we have removed 3659 spurious MOF structures using mofchecker^32^ as well as an element-specific, charge-based heuristics. Within the remaining 6484 MOFs, we have identified those structures providing the highest productivity associated with the lowest energy requirement, as defined in Supplementary Note 4, while achieving a purity of at least 95% as required for CO_2_ capture applications at NGCC power plants. In the following sections, we detail how this evaluation was performed.

### Active learning-based structure selection

We have built an active-learning model for predicting CO_2_ and N_2_ adsorption free energy. The training data was extracted by fitting model functions to the GCMC-simulated adsorption isotherms, see Methods section. The active learning workflow uses extensive featurization combining multiple tools. In short, feature selection is performed by combining Lasso regression and variable importance discerned from initial machine-learning (ML) models. Multiple ML models are built using the selected features and free energies of adsorption, see Fig. 2a. The best-performing model with the lowest uncertainty is used for Pareto-searching higher-performing materials for subsequent iterations. The material selection improves CO_2_ vs. N_2_ selectivity as well as CO_2_ capacity, and selects materials with lower SCScore^33^ as well as lower prediction uncertainty. Given that selectivity has been shown to be a poor descriptor of separation performance^9^, we have assigned higher priority to $\Delta {G}_{{{{\rm{CO}}}}_{2}}$ as it represents both selectivity and capacity of 4% CO_2_ NGCC flue gas in the Henry regime. Further details are provided in the Methods section.

Overall, 982 materials were subjected to GCMC simulations over 5 active learning iterations. The progression of material selectivity is shown in Fig. 2b. The best performing material in each iteration is depicted by solid symbols, showing stepwise selectivity improvements. As a result of the active learning process that targets CO_2_ selectivity, the distribution successively shifts towards structures with narrower pores, see Fig. 2c. Most materials in iterations 4 and 5, respectively, are MOFs having a pore-limiting diameter close to 2.5 Å. We note that for structures with pore dimensions similar to the size of the gas molecules, mass transfer is controlled by micro-pore diffusion^34^.

In the following, we will complement the analysis of the 982 MOF structures by simulating molecular diffusion of CO_2_ and N_2_, respectively, for each individual MOF structure within the refined data set. The diffusion data are then combined with the adsorption data for enabling the process-level CO_2_ capture evaluation of each MOF.

### Molecular- to process-level evaluation

**Fig. 3:**
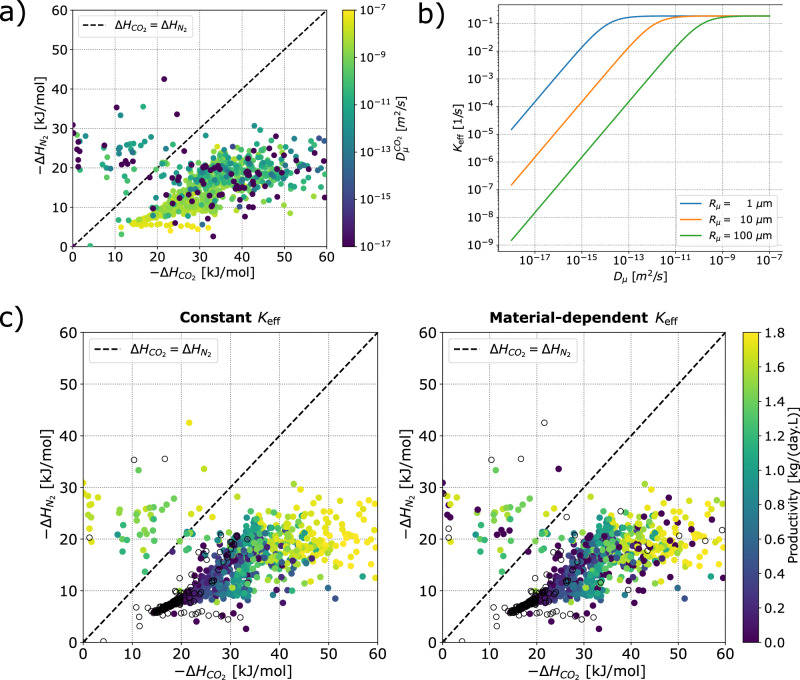
Effect of molecular diffusion on CO_2_ capture productivity. **a** Distribution of heat of adsorption for CO_2_ and N_2_, respectively, for a set of select MOF structures. For each structure, the diffusion coefficient is indicated by color. Note, that MOFs with a high $\Delta {H}_{C{O}_{2}}/\Delta {H}_{{N}_{2}}$ ratio can have negligible microscopic CO_2_ diffusion. **b** Effective mass transfer coefficient *K*_eff_ as function of the microscopic diffusion coefficient *D*_*μ*_ for three representative crystallite diameters *R*_*μ*_. Below a diameter-dependent diffusivity threshold, mass transfer is limited by microscopic diffusion. **c** CO_2_ capture productivity simulated at process-level with a material-independent, constant *K*_eff_ (left) as well as with a material-dependent *K*_eff_ that accounts for the microscopic diffusion coefficient of each MOF (right). Open symbols represent materials for which the process-level simulation did not yield a CO_2_ purity of at least 95%. Source data are provided as a Source Data file.

For all selected MOF structures, we have simulated CO_2_ and N_2_ molecular diffusion and adsorption uptake at representative pressure and temperature levels. The self-diffusion coefficient was calculated based on mean squared molecular displacement. The heat of adsorption was extracted from the adsorption isotherms by taking the dominant site of the best fit multi-site Langmuir model which is representative of adsorption affinity. In Fig. 3a, we plot the heat of adsorption for CO_2_ and N_2_, colored by the CO_2_ diffusion coefficient at *T* = 30 °C. We focus our analysis on the lower right region with regards to the parity line which contains promising capture materials candidates due to their comparatively higher CO_2_ adsorption affinity. Among those structures, MOFs with a low CO_2_ heat of adsorption, between 10 and 30 kJ/mol, tend to diffuse faster, ${D}_{\mu }^{{{{\rm{CO}}}}_{2}}\gtrsim 1{0}^{-9}\,{{{\rm{m}}}}^{2}/{{\rm{s}}}$. With increasing CO_2_ heat of adsorption, the corresponding diffusion coefficient tends to decrease several orders of magnitude, due to the stronger binding affinity and/or smaller pore size. We note that the N_2_ diffusion coefficient is usually 2–5 times larger, as shown in Supplementary Fig. 8.

The predominance of MOFs with high $\Delta {H}_{C{O}_{2}}/\Delta {H}_{{N}_{2}}$ ratio but low diffusivity (${D}_{\mu }^{{{{\rm{CO}}}}_{2}}\lesssim 1{0}^{-11}\,{{{\rm{m}}}}^{2}/{{\rm{s}}}$) has important implications. Generally, we expect that a high-performing CO_2_ capture material should provide conditions under which gas adsorbents efficiently diffuse within the pores *and* bind strongly to the adsorption sites, even though a trade-off exists. For analyzing the impact of molecular-level diffusivity in each MOF structure at the process level, the numerical diffusivity values, *D*_*μ*_, are propagated via the effective mass transfer coefficient *K*_eff_ in Equation (2). The *K*_eff_ *vs*. *D*_*μ*_ curves in Fig. 3b exhibit two distinct regimes: micro-porosity limited transfer, and macro-porosity limited transfer. Each value of the crystallite size *R*_*μ*_ is associated with a characteristic threshold below which mass transfer is limited by microscopic diffusion. As a rule-of-thumb, we conclude that the larger the crystallite diameter, the stronger the impact of microscopic diffusion.

To illustrate the crystallite size dependence of the results, we have chosen three characteristic diameters: 1, 10 and 100 μm. The 1-μm and 10-μm values characterize the lower and upper bounds, respectively, of typical crystal size distributions taken from commercially available samples. The 100-μm value serves as an extrapolation for confirming the sensitivity observed at the 10 μm-level. Nevertheless, from a volume fraction perspective, larger crystallites dominate despite their lower occurrence.

We now compare the process-level CO_2_ capture productivity for two scenarios: a constant, material-independent *K*_eff_, equivalent to setting *R*_*μ*_ = 0 in Equation (2), thus neglecting microscopic diffusivity limitations, as well as a structure-dependent *K*_eff_ accounting for microscopic diffusivity. In both cases, we have assumed a crystallite diameter of *R*_*μ*_ = 10 *μ*m and a CO_2_ purity of 95%. The resulting productivity values are indicated by color shades in Fig. 3c. For the group of select MOFs, the plot reveals a correlation between CO_2_ affinity, CO_2_/N_2_ selectivity, and capture productivity. Most MOFs in our selection have a low CO_2_ affinity, as evidenced by a small $\Delta {H}_{C{O}_{2}}-\Delta {H}_{{N}_{2}}$ difference. Due to the high concentration of N_2_ in the feed stream, their separation performance is low. Therefore, we focus our analysis on the right-hand side of the graphs. As a key result of the comparison, 16% of high-productivity MOFs, i.e., $\gtrsim 1\frac{{{\rm{kg}}}}{{{\rm{day}}}}.{{\rm{L}}}$, fail to sustain high productivity once molecular diffusivity is taken into account by means of a structure-dependent *K*_eff_. From a computational screening perspective, we consider these structures as “false leads” that can be effectively removed from the performance-based MOF rank-order.

### Process-level optimization and materials ranking

**Fig. 4:**
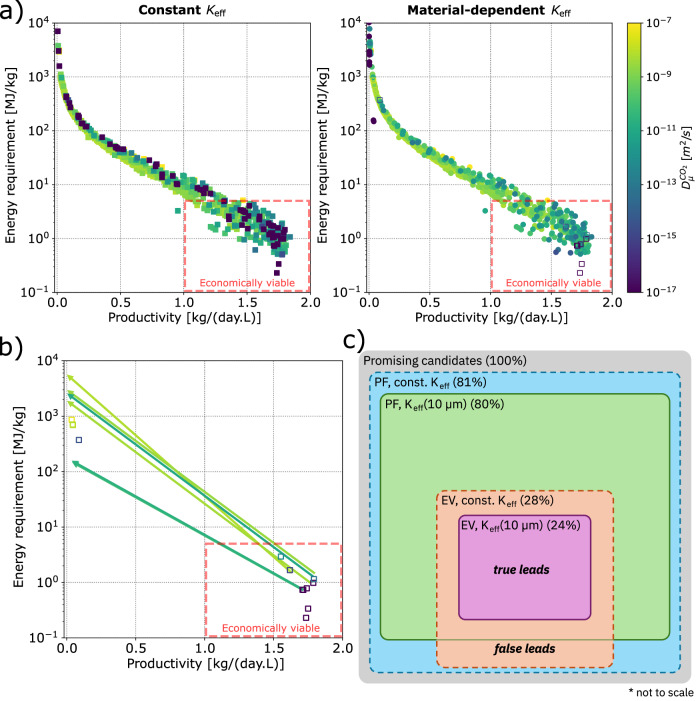
Effect of molecular diffusion on energy requirement and productivity. **a** Distribution of energy requirement *vs*. productivity for a crystallite diameter *R*_*μ*_ = 10 *μ*m, assuming a constant *K*_eff_ (left) and a material-dependent *K*_eff_ (right) that accounts for microscopic diffusivity. The respective CO_2_ diffusion coefficients are indicated by color. Solid squares represent “process-feasible” (PF) materials, i.e., those whose performance achieved 95% purity and 90% recovery, while open squares mark the positions of excluded materials whose performance failed to meet those targets. The dashed rectangle delineates the “economically viable” (EV) region with productivity higher than $1\frac{{{\rm{kg}}}}{{{\rm{day}}}.{{\rm{L}}}}$ and energy requirement lower than 5 MJ/kg of CO_2_. **b** Effect of microscopic diffusivity in *K*_eff_ on process performance for crystallite size *R*_*μ*_ = 10 *μ*m. Arrows indicate changes undergone by structures for which the inclusion of molecular diffusivity has led to a significant drop in process performance, pushing them out of the EV region. **c** Percentages of PF and EV materials with respect to the starting set of “promising candidates” as selected by the active-learning model, assuming constant (dashed lines) or material-specific (solid lines) *K*_eff_. The percentage of PF materials dropped from 81% (798 out of 982) to 80% (784 out of 982) upon inclusion of microscopic diffusivity. The prevalence of EV materials went from 28% (277 out of 982) to 24% (234 out of 982) of the starting set, or from 35% (277 out of 798) to 30% (234 out of 784) of their respective PF set. This corresponds to a 16% reduction (from 277 to 234) in the original EV population, representing the “false leads''. Source data are provided as a Source Data file.

For a given carbon dioxide capture process, the choice of the best capture material, ultimately, depends on the trade-off between productivity and energy requirement. For our select group of MOF structures, we plot in Fig. 4 energy requirement as function of productivity for a representative TSA process. We compare side-by-side the results obtained by assuming a constant and a material-dependent *K*_eff_. We note that the distribution of energy requirement spans several orders of magnitude while the productivity distribution is relatively narrow.

We introduce terminology for describing groups of materials at different performance screening levels. We call “promising candidates”, those out of the 6484 initial structures that were preselected by the active learning model to undergo multi-scale simulations. Among those, we term “process-feasible” (PF) those whose simulated process-level performance achieves 95% CO_2_ purity and 90% CO_2_ recovery targets. Finally, the subset of PF materials that achieve CO_2_ productivity higher than $1\frac{{{\rm{kg}}}}{{{\rm{day}}}.{{\rm{L}}}}$ and energy requirement lower than 5 MJ/kg of CO_2_ is labeled “economically viable” (EV).

In Fig. 4a, left panel, we plot energy requirement as function of productivity assuming a constant, material-independent *K*_eff_. In order to guide the reader and allow for comparison, we have assigned colors for indicating the respective CO_2_ diffusion coefficient of each structure, even though those diffusivity values were not used to calculate *K*_eff_ in the simulations that originated this plot. We note that many of the materials in the EV region have very small CO_2_ diffusivity values. Such small diffusivity values could lead to significant changes in CO_2_ capture performance if accounted for in the process model.

For analyzing the change, we have included the microscopic diffusion coefficients in the mass transfer coefficient *K*_eff_, see Equation (2), and recalculated energy requirement and productivity for all structures assuming a diffusivity-dependent *K*_eff_. The results are shown in the right panel of Fig. 4a. We note that high-performance MOFs that were initially located in the EV region are now excluded by failing to surpass the purity/recovery process feasibility targets. In the plot on the right, their initial positions are marked by open symbols. We find that this trend depends on crystallite size, having a smaller impact for *R*_*μ*_ = 1 *μ*m, see Supplementary Fig. 15, and a larger impact for *R*_*μ*_ = 100 *μ*m, see Supplementary Fig. 16.

In Fig. 4b–c, we have identified the structures whose process-level performance was the most affected. We compare their performance coordinates before and after inclusion of molecular diffusivity for *R*_*μ*_ = 10 μm. For reference, we plot open symbols in their original positions taken from the left panel of Fig. 4a. The arrows indicate MOFs that moved out of the EV region once their structure-dependent, molecular diffusivity was included into the calculation of *K*_eff_. The percentage of promising candidates that achieved the purity/recovery process feasibility targets was 81% under the constant *K*_eff_ approximation, but some of them no longer achieved these targets after the inclusion of microscopic diffusivity. The relative reduction in the PF population grows from 1%, to 2%, to 5% with increasing crystallite diameter *R*_*μ*_ = 1, 10 and 100 *μ*m, respectively. We now turn our attention to the subset of EV materials, which correspond to 28% of the promising candidates and 35% of the PF population, under the constant *K*_eff_ approximation. In this case, the relative reduction of the EV population was 14%, 16% and 20% for *R*_*μ*_ = 1, 10 and 100 μm, respectively. We term these materials as “false leads”, as they would have been wrongly ranked high despite their low performance due to diffusivity constraints.

**Fig. 5:**
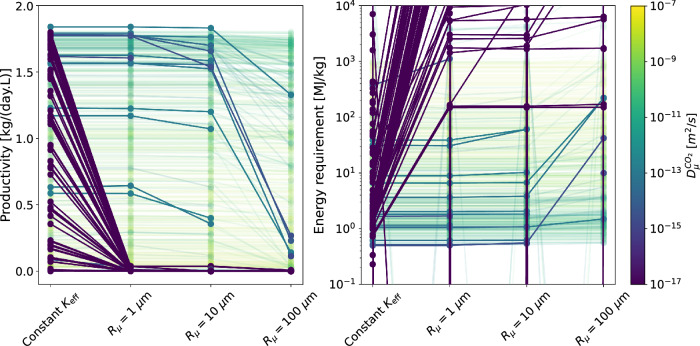
Effect of molecular diffusion on CO_2_ capture performance ranking. Productivity (left) and energy requirement (right) based on constant *K*_eff_, as well as structure-dependent *K*_eff_ for representative crystallite sizes *R*_*μ*_ = 1, 10 and 100 μm. Each curve represents a specific MOF structure and its color represents its microscopic diffusion coefficient. The higher (lower) the productivity (energy requirement), the higher the performance rank of the candidate material. High diffusion coefficients are displayed with transparency for making productivity drops and energy requirement increases visible in the plots. Data points for structures that fail to surpass the 95%-purity threshold are not shown. Source data are provided as a Source Data file.

We now discuss how the observed changes affect the CO_2_ capture performance rank-order. Potentially interesting materials, with high productivity and low energy requirement, may lose their high ranking upon consideration of microscopic diffusivity. Figure 5 depicts this effect whereby low diffusivity materials drop from the upper ranks upon increasing importance of microscopic diffusivity in the overall mass-transfer coefficient. Materials with higher diffusion coefficients are shown with nearly horizontal curves, as the increase in *R*_*μ*_ does not severely affect *K*_eff_ and hence process based performance. On the other hand, lower diffusivity materials are characterized by drops (rises) in productivity (energy requirement) upon increasing *R*_*μ*_. These results are in accordance with the behavior of *K*_eff_ in Fig. 3b.

As representative examples, we discuss in Supplementary Note 4 the properties of the two high-performing MOFs, MOCHIW and MOCHIW, as well as the two low-performing MOFs, BETGUE and EVOMOR, introduced in Fig. 1. Specifically, we provide for each MOF the adsorption isotherms (Supplementary Fig. 11), diffusion coefficients (Supplementary Fig. 12), breakthrough cycle profiles (Supplementary Fig. 13) and process-level Pareto plots (Supplementary Fig. 14). The plots enable a in-depth analysis of how the inclusion of molecular diffusion affects each step of the multi-scale performance evaluation.

## Discussion

Using a micro- to macro-scale, computational simulation and discovery workflow for MOFs, we have demonstrated how the inclusion of structure-dependent, molecular diffusion dynamics affects post-combustion CO_2_ capture figures-of-merits, i.e., purity, recovery, productivity, and energy requirement, of a simulated TSA process for application at NGCC power plants.

An active-learning, structure selection model has iteratively identified a group of 982 MOFs with high molecular CO_2_ / N_2_ selectivity and CO_2_ capacity $(\Delta {G}_{{{{\rm{N}}}}_{2}}-2\times \Delta {G}_{{{{\rm{CO}}}}_{2}})$. As the identified structures exhibit narrow pore diameters, of the order of the size of the gas molecules, their CO_2_ capture performance is limited by microscopic diffusion.

For quantifying the effect, we have compared the simulated TSA process performance obtained by using an effective mass transfer coefficient for all structures, as well as by calculating for each of the 982 MOFs in the selection a structure-dependent mass transfer coefficients accounting for molecular-scale diffusion dynamics. While pellet properties of representative MOFs have previously been used as decision variables for optimizing macro-pore mass transfer in vacuum-swing adsorption^25^, we have focused in our work on microscopic diffusivity in crystallites, mainly due to the pore size distribution of the MOF candidates under study.

Overall, the inclusion of molecular diffusion has led to the exclusion of up to 20% of MOF candidates from the initial rank-order. This is because either the structures did not achieve the 95% CO_2_ purity threshold or because of their performance loss in terms of productivity and energy requirement. In the rank-order obtained by using structure-dependent diffusivity and mass transfer coefficients, 27 MOFs have achieved volumetric productivity larger than 1.5 kg/(day.L) and specific energy consumption lower than 1 MJ/kg. For comparison, reference CO_2_ solid sorbents such as Zeolite 13X and well-known MOF materials UTSA-16 and Mg-MOF-74, have reported productivity and energy consumption of 1–3 kg/(day.L) and 0.5–1 MJ/kg, respectively, in vacuum-swing adsorption^17^. More recently, CALF-20 has achieved industrial-scale production with a productivity of 0.53 kg/(day.L) and an energy requirement of 0.72 MJ/kg, respectively, in pressure-vacuum-swing adsorption^35,36^.

In Supplementary Table 10, we provide our top-20 performance list that exemplifies the effect of microscopic diffusivity on rank-order that increases with crystallite diameter. For *R*_*μ*_ = 1, 10 and 100 *μ*m, respectively, a total of 3, 7, and 10 MOFs have dropped off the initial ranking. In case of the best-performing material, BOHGOU, the inclusion of molecular diffusivity in the process simulation has decreased volumetric productivity, from 1.84 to 1.33 kg/(day.L), and increased the energy requirement, from 1.07 to 1.50 MJ/kg of CO_2_. We note that in the case of *R*_*μ*_ = 100 *μ*m, BOHGOU drops out of the top-20 while the second best material for all other crystallite sizes, MOCHIW, moves to the first position. This highlights the importance of MOF crystallite size on CO_2_ capture performance.

**Fig. 6:**
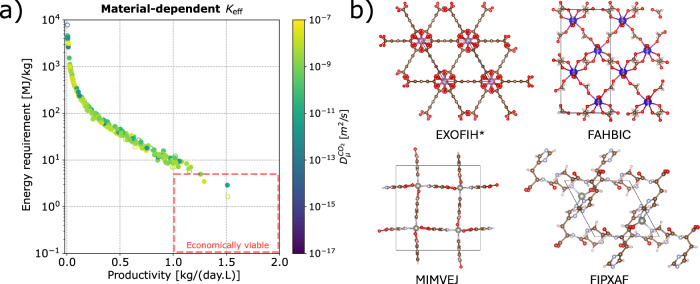
Water-tolerant MOF candidates. **a** Distribution of energy requirement *vs*. productivity for water-tolerant MOFs, i.e., with $\Delta {G}_{{{{\rm{H}}}}_{2}{{\rm{O}}}} > -8.1\,{{\rm{kJ}}}/{{\rm{mol}}}$, assuming a crystallite diameter of *R*_*μ*_ = 10 *μ*m. We have identified 4 promising MOF structures in the economically viable region. Empty symbols represent structures flagged as “bad” by MOSAEC^37^ **b** Crystal structures of the economically viable, water-tolerant MOF candidates. We marked EXOFIH to represent that it was flagged as “bad” by MOSAEC^37^. Source data are provided as a Source Data file.

In this work, we have focused on methodologically including microscopic diffusivity in computational material screening. To that end, we had to make reasonable simplifications to other equally challenging dimensions of material performance evaluation. In particular, we have assumed dry NGCC flue gas throughout the results reported in this manuscript. In order to address performance implications related to the presence of water, out of the 982 MOFs under consideration, we have removed those with higher water affinity than CALF-20^35^ ($\Delta {G}_{{{{\rm{H}}}}_{2}{{\rm{O}}}}\le -8.1\,{{\rm{kJ}}}/{{\rm{mol}}}$). To further validate of our findings, we performed an additional, structural quality check based on metal oxidation states, by using MOSAEC^37^. As shown in Fig. 6a, 4 out of the 381 remaining MOFs, referred to as “water-tolerant”, fall within the economically viable region. Note, that these MOFs have CO_2_, N_2_ and H_2_O affinities that are similar to or better than those reported for CALF-20, see Supplementary Note 4.3. We note that one of the structures, namely EXOFIH, was flagged as “bad” by MOSAEC. Interestingly, two of them have one-dimensional square pores similar to CALF-20. We would like to emphasize that, by virtue of including diffusion in the screening workflow, these water-tolerant MOFs would not be kinetically limited. To assess the impact of using EQeq charges, we re-ran the end-to-end simulation workflow for all the water-tolerant, economically-viable MOFs using DDEC06 charges computed with PACMOF^38^. Their relative rank order with regard to CO_2_ capture performance remained unchanged. The refined water tolerance screening is provided in Supplementary Note 4.4.

We conclude that the materials at the top of the diffusion-enabled MOF ranking are high-performing CO_2_ sorbents with the potential to challenge, or even surpass, the performance of the best solid sorbents deployed today. Future work should include structural checks with MOFClassifier^39^ and address stability and synthesizability within the screening protocol. For enabling their validation and exploration, we have made our data publicly available, see Data availability. The computational simulation workflow for molecular diffusivity, which is based on Crystallographic Information File (CIF) format and made available as open-source code, see Code availability, could be similarly applied to the evaluation of broader classes of solid sorbents, including covalent organic frameworks and zeolites.

## Methods

Methodologically, we have integrated machine-learning based structure selection, molecular-level simulation, as well as process-level simulation for optimizing CO_2_ capture performance. MOF properties derived from the molecular-level simulations are used as input for both the active-learning structure selection as well as the equations of the process-level temperature-swing adsorption model.

### Spurious structure removal

We have used mofchecker^32^ to eliminate 3246 CoRE-MOF-2019 structures from our data set based on a suite of composition, coordination and consistency checks. An additional set of 413 structures were eliminated by an element-specific, charge-based heuristics. The heuristic aims at the removal of structures whose atoms present partial charges at the top, or bottom, 1% of a particular element charge distribution. The breakdown of elimination criteria by percentage and acceptable charge ranges per element are provided in Supplementary Note 1.

For the analysis of water-tolerant MOFs, see Fig. 6, we applied additional, spurious structure removal criteria. White et al. proposed a pipeline that further refines the identification of spurious structures, leading to the removal of 224 structures that were flagged as “bad” by MOSAEC^37^.

### Active-learning based structure selection model

As shown in Fig. 2a, active learning modeling is done by combining extensive featurization with iterative material selection. The featurization workflow combines multiple independent featurizers such as MAGPIE^40^, JARVIS^41^, persistent homology^42^, CGCNN^43^, synthesis complexity score (SCScore)^33^, Zeo++^44^ and PoreBlazer^45^. As a fist step of the iterative materials section process, feature selection is carried out to reduce the feature space. This is done using combination of Lasso regression, variable contribution from random forest ensembles and shapely metrics. The reduced feature space is used to predict adsorption free energies Δ*G* for CO_2_ and N_2_. We employ an ensemble of models to predict the adsorption performance. Our ML modeling tool box comprised of GPR, RFE, SVM, PLS, NN with details given in Supplementary Note 2. The best model is selected based on performance on the test dataset, as well as the prediction uncertainty over the entire database. The selected model is used to Pareto search and select the next set of materials to be subjected to detailed GCMC simulations.

The Pareto search is performed by optimally trading-off $\Delta {G}_{{{{\rm{N}}}}_{2}}$ against $-2\times \Delta {G}_{{{{\rm{CO}}}}_{2}}$ to maximize CO_2_/N_2_ selectivity and CO_2_ capacity simultaneously. Within each Pareto front, the selected materials are sorted and filtered based on their SCScore and based on the prediction uncertainty for adsorption free energy. We carried out 5 such active learning iterations, each including: detailed molecular simulation, (re-)building machine learning models to predict adsorption performance and material selection for subsequent iterations.

### Molecular-level simulations

The molecular-level simulation framework consists of a combination of the Grand Canonical Monte Carlo (GCMC), Widom particle insertion and Molecular Dynamics (MD) methods for simulating the adsorption isotherms and diffusion coefficients, respectively. In all cases, we choose RASPA^46,47^ as our simulation engine. The interaction energies between non-bonded atoms were computed using the Coulomb and Lennard-Jones (LJ) potentials with Lorentz-Berthelot^48^ mixing rules

1 $${U}_{ij}({r}_{ij})=4{\varepsilon }_{ij}\left[{\left(\frac{{\sigma }_{ij}}{{r}_{ij}}\right)}^{12}-{\left(\frac{{\sigma }_{ij}}{{r}_{ij}}\right)}^{6}\right]+\frac{1}{4\pi {\epsilon }_{0}}\frac{{Q}_{i}{Q}_{j}}{{r}_{ij}},$$

where *r*_*ij*_ is the interatomic distance, ${\varepsilon }_{ij}=\sqrt{{\varepsilon }_{ii}{\varepsilon }_{jj}}$ is the well depth and ${\sigma }_{ij}=\frac{{\sigma }_{ii}+{\sigma }_{jj}}{2}$ is the well diameter between atoms *i* and *j*. The values of LJ parameters for framework atoms are a hybrid between the Universal Force Field (UFF)^49^ and DREIDING^50^ (see Supplementary Table 6), while the LJ parameters for the adsorbate molecules were taken from the TraPPE^51^ force field (see Supplementary Table 7). Electrostatic energy contributions were calculated using EQeq^52^ partial atomic charges *Q*_*i*_ and the Ewald sum technique. Despite the emergence of structure databases with DFT-derived partial atomic charges^53–55^, we have chosen EQeq charges which, although approximate and subject to certain limitations, offer certain benefits^56,57^.

The post-processing of GCMC time series and MD trajectories was fully automated. The equilibrated averages of molecular uptake were extracted from the GCMC time series using the Marginal Standard Error Rule (MSER) as implemented in pyMSER^58^ and shown in Supplementary Fig. 3. Adsorption isotherm curves for CO_2_, N_2_ and H_2_O were built by simulating the molecular uptake at several pressures, for each considered temperature, as shown in Supplementary Fig. 4. Multi-site Langmuir adsorption models (see Equation 1 on Supplementary Note 3.1) were adjusted to the data points, and the best fit was kept. The resulting analytical expressions for the adsorption as a function of pressure and temperature were input to the process-level model equations.

The self-diffusion coefficients were extracted from the MD trajectories using an automated routine that locates the normal diffusion regime (〈*r*^2^〉 ∝ *t*, see Supplementary Fig. 6) in the mean squared displacement curve, as shown in Supplementary Fig. 7. Then, the self-diffusion coefficients *D*_*μ*_ calculated at different temperatures were adjusted to an Arrhenius expression (see Equation 4 on Supplementary Note 3.2.4), as shown in Supplementary Fig. 9. Finally, we have used the fitted expression for *D*_*μ*_(*T*) as transport diffusivity under low loading approximation for the process modeling.

For more information on the approach to molecular-level modeling, please refer to Supplementary Note 3.

### Process-level simulations

The process-level model assumed a four-step temperature-swing adsorption cycle—i.e., adsorption, purge, desorption and cooling—in a fixed bed configuration, as depicted in Supplementary Fig. 10c. gPROMS® Model Builder software was used for the implementation. The packed bed is modeled as one-dimensional, including heat and mass transfer. The effective mass transfer coefficient^59^ is defined as

2 $${K}_{{{\rm{eff}}}}=\frac{15}{\left(\frac{{R}_{p}^{2}}{{D}_{p}^{*}}+\frac{{R}_{\mu }^{2}}{{D}_{\mu }}\right)},$$

where the pellet radius *R*_*p*_ = 2 mm and effective pellet diffusivity ${D}_{p}^{*}=5\times 1{0}^{-8}\,{{{\rm{m}}}}^{2}/{{\rm{s}}}$ represent the macropore (or intercrystalline) diffusion ignoring external film resistance. The crystallite radius *R*_*μ*_ and diffusivity *D*_*μ*_ represent the micropore (or intracrystalline) diffusion. Thermal management is achieved using a temperature-controlled water stream flowing through the heat exchange jacket of the bed as well as direct heat exchange through steam or cooler flue gas. The competitive adsorption of CO_2_ and N_2_ is modeled as a multi-component, mixed Langmuir isotherm derived from the combination of pure component isotherms during adsorption. The H_2_O is included in the process modeling as an inert purge gas during regeneration, with the purpose of accounting for the steam requirement within the specific energy evaluation.

Mass balance was imposed for the gas and adsorbed phases (Equation 6 on Supplementary Note 4.1). The rate of change in the concentration of the adsorbed phase was modeled as a linear restorative term proportional to *K*_eff_ (Equation (2)) towards the equilibrium concentration (Equation 1 on Supplementary Note 3.1). The energy balance at the gas and adsorbed phases, outer walls and external heat transfer fluid is described in Equation 7 on Supplementary Note 4.1. The momentum balance is modeled using the Ergun equation (Equation 6 on Supplementary Note 4.1) and the total pressure at each point is determined using the ideal gas law.

The desorption temperature and the four cycle step times, namely, adsorption step time, purge step time, desorption step time and cooling step time, are subject to an optimization routine to determine the combination of values that lead to the best performance. The possible choices for these parameters contain inherent performance trade-offs, defining a Pareto front. The point with 95% purity is selected from the Pareto front as representative of the material’s performance metrics.

## Supplementary information

Supplementary Information

Transparent Peer Review file

## Source data

Source Data

## Data Availability

The MOF structures, feature sets, isotherm data, diffusivity data, process performance data, cyclic simulation profiles, as well as MOF ranking data generated in this study have been deposited in the Zenodo database under accession code 10.5281/zenodo.13948294. The molecular-level simulation data, augmented with process-level metrics, is provided in JSON format. The process-level metrics data generated in this study are provided in the Supplementary Information/Source Data file. Source data are provided with this paper.

## Code availability

The molecular-level simulation framework uses ST4SD^60^ as the workflow orchestrator, and is available as an open source project on GitHub and Zenodo^61^.
