# Supplementary material for: Molecular diffusion enhanced performance evaluation of metal-organic frameworks for CO2 capture
Source: Nat Commun. 2026 Jun 17;17:7654. doi: 10.1038/s41467-026-73904-5 (PMC13434109; doi:10.1038/s41467-026-73904-5)
Supplement: Supplementary file 1 [file 41467_2026_73904_MOESM1_ESM.pdf]

Supplementary Information for

Molecular diffusion enhanced performance evaluation of metal-organic frameworks for CO_2 capture

Rodrigo Neumann Barros Ferreira[1, †], **Yogesh V. Joshi**[2, †], **Felipe Lopes Oliveira**[1, 3], **Anantha Sundaram**[2], **Ashish B. Mhadeshwar**[2], **Jayashree Kalyanaraman**[2], **Binquan Luan**[4], **and Mathias Steiner**[1,*]

[1]IBM Research, Av. República do Chile, 330, Rio de Janeiro, RJ, 20031-170, Brazil.
[2]ExxonMobil Technology and Engineering Company, 1545 Rt 22E, Annadale, New Jersey 08807, USA.
[3]Instituto de Química, Universidade Federal do Rio de Janeiro, Av. Athos da Silveira Ramos, 149, CT A-622, Cid. Univ., Rio de Janeiro, Rio de Janeiro, 21941-909 - Brazil.
[4]IBM Research, 1101 Kitchawan Road, Yorktown Heights, New York 10598, USA.
[*]mathiasbsteiner@gmail.com
[†]Equal contribution

Contents

Supplementary Note 1 Removal of spurious MOF structures **1**

Supplementary Note 2 Active learning structure selection model **1**
Supplementary Note 2.1 Featurizing the learning space . . . 6
Supplementary Note 2.2 Feature importance by Shapely Explanation . . . 8

Supplementary Note 3 Molecular-level simulations **8**
Supplementary Note 3.1 Grand Canonical Monte Carlo . . . 8
Supplementary Note 3.2 Molecular Dynamics . . . 11
Supplementary Note 3.2.1 Diffusion coefficient calculation . . . 12
Supplementary Note 3.2.2 Validation against experiments . . . 13
Supplementary Note 3.2.3 Distribution of diffusion coefficients for different adsorbates . . . 14
Supplementary Note 3.2.4 Temperature-dependent diffusion coefficients . . . 14

Supplementary Note 4 Process-based screening **16**
Supplementary Note 4.1 Temperature-swing adsorption process . . . 16
Supplementary Note 4.2 Process-level performance metrics . . . 18
Supplementary Note 4.3 Water affinity of top performing MOFs for dry flue gas separation . . . 20
Supplementary Note 4.4 Impact of charge assignment scheme on the rank ordering of water-tolerant, economically-viable MOFs . . . 22

Supplementary References **30**

Supplementary Note 1 Removal of spurious MOF structures

Besides using `mofchecker`[1] to eliminate 3,246 out of 10,142 structures from the CoRE-MOF-2019 database due to a combination of composition, coordination and consistency checks, we have also implemented our own charge-based filtering criteria. We found that the charge-based filter from `mofchecker`, which flags atoms with EQeq charges outside of the $[-3,+3]$ range, was not sufficient to eliminate all spurious structures from the CoRE-MOF-2019 database, mainly due to different elements having different oxidation states and different charge ranges. We have, therefore, collected a set of acceptable charge ranges by considering the 1st and 99th percentiles of the element-specific charge distributions in the QMOF[2] database. The Application of this additional filter has removed another 413 structures from the data set, leaving only 6,484 candidate structures for further evaluation. The breakdown of elimination criteria and their respective percentages is provided in Supplementary Table 1.

Supplementary Table 1. Percentage of CoRE-MOF-2019 structures flagged by each individual check.

#	Elimination criteria	% structures flagged
1	`has_carbon`	5.3%
2	`has_hydrogen`	10.8%
3	`has_atomic_overlaps`	0.6%
4	`has_overcoordinated_c`	0.6%
5	`has_overcoordinated_n`	0.1%
6	`has_overcoordinated_h`	0.5%
7	`has_undercoordinated_c`	10.4%
8	`has_undercoordinated_n`	7.3%
9	`has_undercoordinated_rare_earth`	0.8%
10	`has_metal`	0.0%
11	`has_lone_molecule`	11.1%
12	`has_high_charges`	1.4%
13	`is_porous`	0.1%
14	`has_suspicicious_terminal_oxo`	0.6%
15	`outside_qmof_min_max`	9.9%
	`mofchecker` only	32.0%
	`mofchecker` and `outside_qmof_min_max`	36.1%

We find that EQeq charges computed for the QMOF structures are well behaved and show narrower distributions than for CoRE-MOF-2019, possibly because the structures were DFT-optimized. As an example, we provide in Supplementary Figure 1 the charge distributions for Barium (Ba) in the QMOF and CoRE-MOF-2019 databases. In this example, the heuristics led us to exclude all CoRE-MOF-2019 structures containing Ba atoms with partial charges below 1.44 or above 2.33, as these are considered outliers. In Supplementary Table 2 and Supplementary Table 3, we provide the charge distribution metrics for all elements individually.

We also performed an additional, structural quality check based on metal oxidation states, as implemented by MOSAEC[3]. The overall effect of removing structures flagged as "bad" by MOSAEC was insignificant, except in the case of water-tolerant MOFs, in which one of the four structures identified in the economically viable region was labeled as such, see Figure 6. To demonstrate the effect on Figure 4a and Figure 5, we removed for comparison in Supplementary Figure 2 structures flagged as "bad" by MOSAEC. Overall, both plots appear very similar. A few scattered data points were removed, but the overall distributions, ranges, and trends remain the same.

Supplementary Note 2 Active learning structure selection model

The machine-learning approach taken here mirrors the active learning strategy used in deep-learning and other large-scale regression models. The active learning method dates to the original[4]. The basic principle is to have an intelligent user supply labels to data generated by a machine learning model. In our case the intelligent user is replaced by a combination of experts and the GCMC simulation estimating adsorption properties. The machine learning model in turn is retrained by returning the relabeled data to training the next model.

The machine learning models act as proxy leads to the molecular simulations to capture nascent structure-property indicators, by exploiting available complimentary knowledge from experts or from other datasets. They learn more and more of the physical and chemical interactions as the simulations expand. They are built iteratively, each iteration learning both new

Supplementary Table 2. Element-specific EQeq charge distribution in the QMOF database[2], from Hydrogen (H) to Ruthenium (Ru). We eliminated MOF structures with charges outside of the range from the 1st to the 99th percentile.

Element	Minimum	1st Percentile	50th Percentile	99th Percentile	Maximum
H	-0.49	-0.03	0.05	0.24	0.66
Li	0.53	0.53	0.56	0.58	0.59
Be	1.47	1.47	1.48	1.48	1.48
B	0.02	0.04	1.21	3.18	3.36
C	-1.25	-0.28	0.00	0.50	1.05
N	-0.60	-0.31	-0.19	0.05	0.19
O	-2.02	-1.07	-0.42	-0.08	0.17
F	-1.44	-0.90	-0.42	-0.27	-0.07
Na	0.52	0.56	0.59	0.60	0.61
Mg	1.41	1.43	1.47	1.48	1.49
Al	2.26	2.36	2.39	2.40	2.42
Si	0.51	0.51	0.91	2.98	2.98
P	-0.69	-0.09	0.41	0.64	1.02
S	-1.13	-0.60	0.02	0.73	1.21
Cl	-1.01	-0.76	-0.35	-0.14	0.02
K	0.43	0.58	0.66	0.71	0.77
Ca	1.45	1.46	1.51	1.53	1.55
Sc	2.28	2.28	2.34	2.35	2.35
Ti	3.05	3.06	3.11	3.14	3.15
V	1.58	1.66	1.82	1.86	1.87
Cr	1.10	1.10	1.52	1.65	1.65
Mn	0.99	1.15	1.29	1.35	1.45
Fe	0.81	0.97	1.16	1.29	1.33
Co	0.92	1.01	1.16	1.23	1.26
Ni	0.12	0.94	1.10	1.15	1.18
Cu	0.60	0.70	0.86	0.95	1.01
Zn	0.79	1.11	1.20	1.24	1.29
Ga	1.85	1.85	2.05	2.12	2.15
Ge	1.05	1.05	1.35	2.00	2.14
As	0.08	0.08	0.41	0.53	0.53
Se	-0.96	-0.92	-0.07	0.66	0.67
Br	-1.00	-0.71	-0.23	-0.07	0.08
Rb	0.58	0.62	0.69	0.72	0.76
Sr	1.44	1.46	1.53	1.54	1.55
Y	2.31	2.36	2.39	2.40	2.40
Zr	3.03	3.15	3.21	3.22	3.23
Nb	1.27	1.27	1.64	1.74	1.74
Mo	0.93	0.96	1.21	1.28	1.34
Tc	1.14	1.14	1.16	1.17	1.17
Ru	0.79	0.81	0.93	1.01	1.01

Supplementary Table 3. Element-specific EQeq charge distribution in the QMOF database[2], from Rhodium (Rh) to Plutonium (Pu). We eliminated MOF structures with charges outside of the range from the 1st to the 99th percentile.

Element	Minimum	1st Percentile	50th Percentile	99th Percentile	Maximum
Rh	0.75	0.76	0.88	0.93	0.93
Pd	0.54	0.57	0.72	0.84	0.86
Ag	0.40	0.44	0.54	0.61	0.67
Cd	0.98	1.10	1.21	1.25	1.28
In	1.84	1.87	1.98	2.03	2.04
Sn	0.63	0.65	1.30	1.33	1.34
Sb	0.20	0.21	0.45	1.37	1.37
Te	-0.86	-0.85	-0.60	0.51	0.60
I	-1.02	-0.77	-0.38	0.04	0.20
Cs	0.64	0.65	0.71	0.79	0.79
Ba	1.20	1.46	2.06	2.34	2.44
La	1.44	1.48	1.70	1.77	1.88
Ce	2.10	2.10	2.16	2.20	2.20
Pr	1.87	1.98	2.08	2.10	2.11
Nd	1.43	1.67	2.37	2.61	2.71
Sm	0.87	1.44	2.20	2.41	2.54
Eu	1.82	1.85	2.09	2.23	2.23
Gd	0.97	1.23	1.71	1.86	1.95
Tb	1.12	1.46	2.05	2.18	2.20
Dy	1.06	1.40	1.99	2.13	2.32
Ho	1.22	1.36	1.95	2.08	2.34
Er	1.02	1.33	1.90	2.06	2.09
Tm	1.44	1.44	1.59	1.62	1.66
Yb	1.41	1.44	1.57	1.60	1.60
Lu	1.06	1.06	1.28	1.32	1.32
Hf	1.21	1.24	1.47	1.52	1.53
W	0.00	0.11	0.53	1.29	1.30
Re	0.66	0.66	0.76	1.02	1.02
Ir	0.21	0.21	0.32	1.17	1.17
Pt	0.03	0.11	0.31	0.48	0.48
Au	0.14	0.24	0.28	0.46	0.51
Hg	-0.08	-0.04	0.12	0.29	0.33
Tl	0.55	0.55	0.65	0.77	0.77
Pb	1.03	1.14	1.24	1.30	1.30
Bi	1.70	1.77	1.85	1.92	1.92
Th	0.03	0.03	0.04	0.06	0.06
U	-0.01	0.01	0.04	0.15	0.23
Np	0.02	0.02	0.04	0.06	0.06
Pu	0.02	0.02	0.04	0.04	0.04

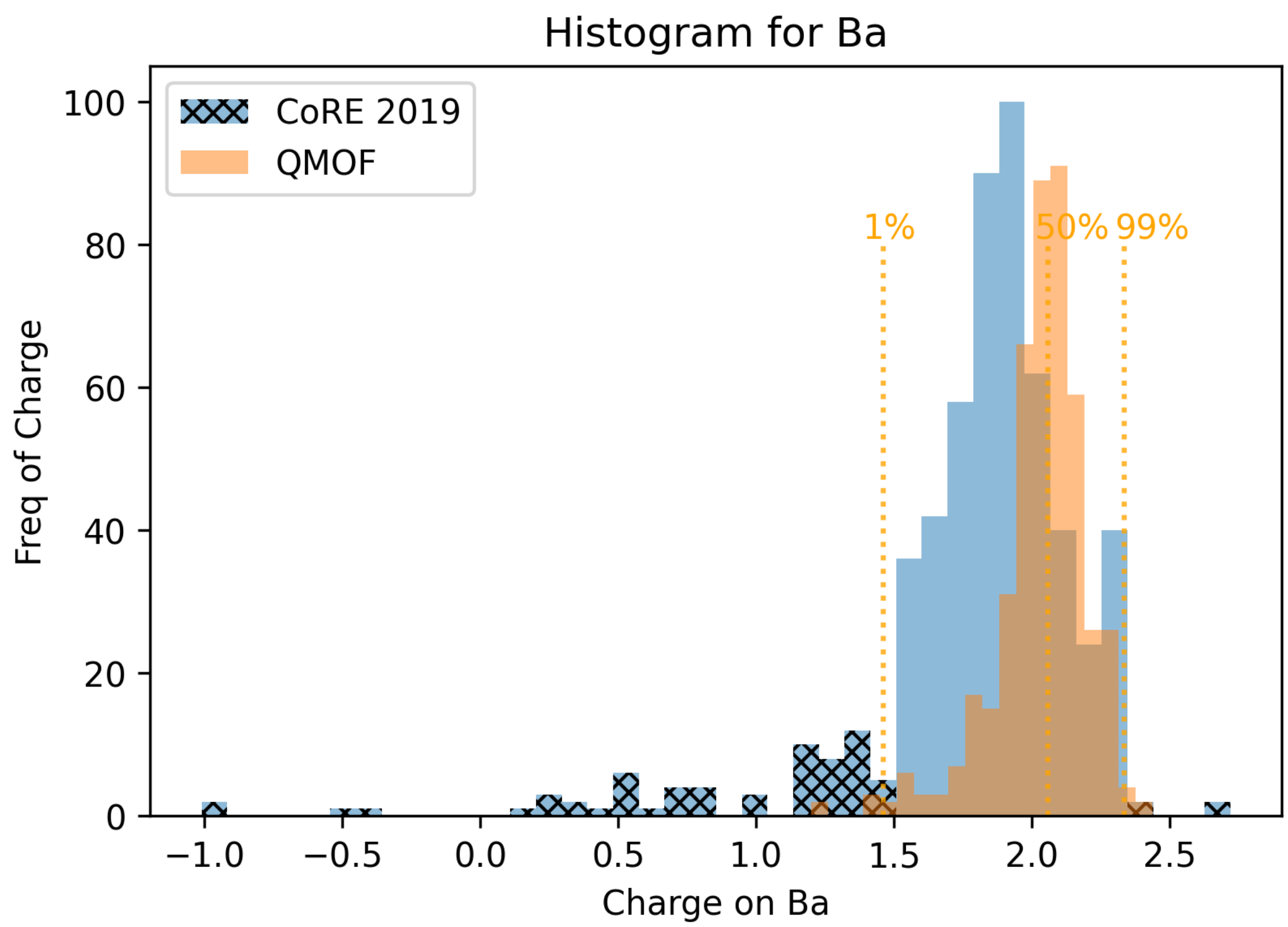

Supplementary Figure 1. Ba charge histogram Partial charge distribution for Barium in the QMOF (orange) and CoRE-MOF-2019 (blue) databases, overlaid to the 1st, 50th and 99th percentiles of the QMOF distribution. The hashed regions show the excluded MOF structures with unreasonable Ba charges.

features and new epistemic components of the models. Multiple modeling paradigms are considered at every iteration, with featurization, model construction and selection repeated every time. The winning model is chosen based on accuracy on extrapolation, structural parsimony, and prediction uncertainty. The chosen model is used to re-estimate the entire space of structures under consideration, for the properties of interest and rank a new set of candidates most promising for the next set of simulations.

These objectives are embedded in the following steps of the iterative active-learning approach. All the steps below were implemented in MATLAB®[5] using its Statistics and Machine Learning[6], Signal Processing[7] and Optimization toolboxes[8].

1. **Featurization**: Generate a large set of features across different structural motifs that are likely to influence the adsorption characteristics of the MOF. A more detailed note on featurization is provided in the next sub-section.
2. **Feature Selection**: Using the results from the latest GCMC iteration, select a subset of features that are strong predictors of two key properties, namely, the free adsorption energies of CO_2 (ΔG_{CO_2}) and N_2 (ΔG_{N_2}) at 30 °C. Features are selected before the model estimation process. To enforce uniformity, features are sub-selected using a combination of Lasso[9], variable contribution from random-forest ensembles[10] and Shapley-inspired metrics[11]. Here, the free energy of adsorption (ΔG) is calculated based on Enthalpy (ΔH) and Entropy (ΔS) of adsorption determined by a Langmuir isotherm model fit.
3. **Model Estimation**: The following models are constructed at every step, each with their own subset of features.
   - Support Vector Machine (SVM), linear and with gaussian kernels
   - Partial Least Squares (PLS) Regression
   - Random Forest Ensembles (RFE MATLAB's XGBoost)
   - Gaussian Process Regression (GPR)
4. **Model Selection**: Every model is trained on 80% of the data and testing on 20% of the data. The training step includes feature estimation and validation to extract the best set of features in the model. The best model among all regressors is

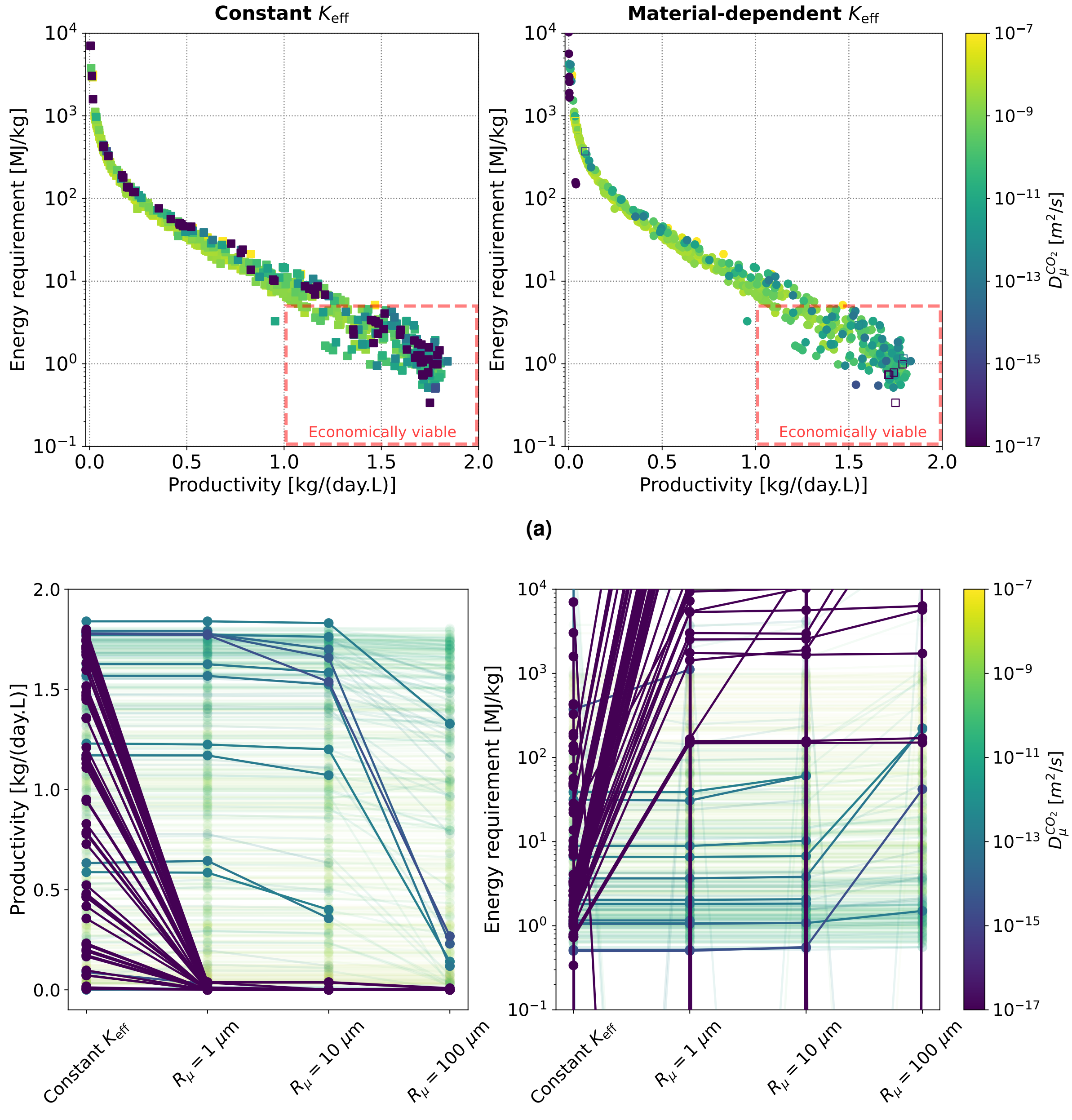

Supplementary Figure 2. Effect of removing structures flagged as "bad" by MOSAEC. (a) Distribution of energy requirement *vs.* productivity for a crystallite diameter $R_\mu = 10$ µm, assuming a constant K_{eff} (left) and a material-dependent K_{eff} (right) that accounts for microscopic diffusivity. (b) Effect of molecular diffusion on CO_2 capture performance ranking. Productivity (left) and energy requirement (right) based on constant K_{eff}, as well as structure-dependent K_{eff} for representative crystallite sizes $R_\mu = 1,\ 10$ and 100 µm.

selected based on performance on the testing set and estimated uncertainty. Uncertainty estimates are provided for each model by their training algorithms[6].

5. **Variable Contribution**: To provide early indication and insight into the nature of structural influence on the predicted properties, variable/feature contributions to the model are estimated for the selected model. The feature contribution ranking is determined using the method developed by Lundberg and Lee[12], where subsets of features enter into an n-person game[13] of training and prediction over split datasets. This method is model agnostic and is extracted over batches of data as opposed to other interpretable metrics that deal primarily with specific instances of model predictions.

6. **Prediction**: The selected (winning) models from (d) above is used to estimate/predict the properties of interest ΔG_{CO_2} and ΔG_{N_2}. In addition the synthetic complexity score, SCS[14] of the organics linker of MOF structure is added to the target set as a desired output. The uncertainty of the predictions is also added in from the model predictions for every structure in the database. These together form the automated design criteria for selection.

7. **Pareto search and MOF selection**: Using the above design criteria, a multi-objective search is conducted over the entire space to simultaneously

   (a) Minimize ΔG_{CO_2} (with higher importance) – stronger CO_2 adsorption capacity

   (b) Maximize ΔG_{N_2} – weaker N_2 adsorption

   (c) Minimize SCScore – simpler organic linker

   (d) Minimize model prediction uncertainty

   This is enforced by doing a formal Pareto search only on the first two criteria above. The Pareto approach follows the formalism established by the Pareto Differential Algorithm (PDE)[15]. For each solution in the database, the key properties (i.e., free energies of adsorption for CO_2 and N_2) are calculated using the selected models as well as their associated uncertainty estimates. The Pareto front is estimated using the dual maximization of $-\Delta G_{CO2}$ and ΔG_{N2}. We found that for 4% CO_2 at 1 bar, most of the MOFs under consideration are far from saturated – hence the 4% CO_2 adsorption capacity is well represented by the free energy of CO_2 adsorption in the Henry regime. To account for both selectivity and capacity of CO_2 in the Henry regime, we weighed ΔG_{CO_2} more strongly, effectively maximizing $\Delta G_{N_2} - 2 \times \Delta G_{CO_2}$ in the Pareto search procedure, alongside synthesis complexity score (SCScore) and model prediction uncertainty. To validate our choice of Pareto search using the free energy scheme described above, we plot the distribution of fractional CO_2 loading for all 982 MOFs using a multi-component isotherm model. The fractional loading is calculated using the isotherm model for NGCC composition (4% CO_2) at 30 °C. The histogram in Supplementary Figure 5a shows that most of the MOFs (759 out of 982) have fractional CO_2 loading below 15% for NGCC flue gas adsorption. This suggests that, for 4% NGCC flue gas, CO_2 loading is well within the Henry region for most of the MOFs considered here.

   Each front consists of solutions that are non-dominated, i.e., no solution is poorer in both criteria along the front. These solutions are "locally-efficient" as defined in the PDE[15] algorithm. Each solution is locally non-dominated within a short radius in the feature space defined by the two properties in this case. We deviate from a full Pareto approach at this stage by deferring to this sequence once the Pareto fronts have been identified. The entire space is filtered by deselecting candidates with very large uncertainties ($> 20-30\%$, typically). Finally, each front is re-ranked in the order of increasing complexity, as estimated by their SCScores.

   The top-n candidates selected via the Pareto search step above form the proposed set from the multi-objective active learner. One can choose from different fronts to progressively create different trade-offs. We could also re-sort the fronts in terms of average SCScore or relax uncertainty cut-offs to promote a diverse set of solutions. These criteria are considered by the experts to rank and propose new targets along with complimentary knowledge about the materials that are not codified in the models. The resulting rich set then becomes the focus of attention of the next set of simulations for following iterations.

   In cultivating newer and successively better models via lower uncertainty in each iteration, the active learner seek to improve accuracy. The Pareto fronts provide a balanced trade-off of "better" solutions based on properties that directly influence performance. Between these two, the active learning approach iteratively walks the trade-off between exploitation and exploration, dynamically altering the search space mappings and model structure to accomplish this balance.

Supplementary Note 2.1 Featurizing the learning space

The featurization step is performed by calculating and pooling together descriptors from different material metrics. While pooling a comprehensive set of descriptors for featurization is a one-time process performed at the beginning of the active learning cycle, feature selection from that comprehensive set is performed at every step and sometimes in consort with model construction. For this work the following measures were considered.

1. **MAGPIE**[16]: These are atomic (typically distance and nearness) averaged features covered in this work[16]. We have used the MATMINER[17]'s implementation of Magpie to generate the features, calling specific featurization vectors pertinent to geometric and electronic properties. The original work[16] is generic to both crystalline and amorphous materials and quite applicable to MOFs. There are 132 features calculated though MAGPIE with each of the feature name starting with "MagpieData".

2. **Force-field features**: Force-fields (electronic, magnetic) represent the different activation gradients underlying many material phenomena. When featurized they form powerful embodiment of structural influences on inter-atomic interactions by condensing them into functional attributes over performance properties. The These force-fields are best obtained by molecular-level simulations under various perturbations. Joint Automated Repository for Various Integrated Simulations (JARVIS)[18], available from NIST, constructs several force-fields of interest either via molecular or atomistic simulations or accurate ML surrogate models. These features have been widely applied in correlating solid structure to semi-conductor performance metrics. However, they also generate features useful to characterize atomic, electronic, topological, mechanical, magnetic and defect-inducing properties[19]. As in several of the MAGPIE calculations, many of them also cover interactions between neighbors, expanding by atomic distance/radii to first, second and beyond neighborhood interactions. There are 1,557 features (starting with "jml") per material from this sub workflow. They comprise of 378 radial charge-distribution based, 100 radial distribution of positions, 179 angle-distribution up to first and second neighboring based each, 179 dihedral angle and 100 nearest neighbor (NN) descriptors. The remaining are 438 average chemical based including various properties and 4 simulation-box-size based to add all to 1557.

3. **CGCNN**[20]: Crystal Graph Convolutional Neural Networks (CGCNN) are a special class of graph neural networks, that are constructed specifically for crystalline structures with periodicity. The message passing connectivity in CGCNN mirror atom-atom connectivity and nearness. The base transfer function and architecture are that of a convolutional neural network. In the CGCNN however the final layer of the neural network represents the latent dimensions of the structural features. Since the CGCNN is a supervised network, these latent dimensions are obtained in training with both input and output labels, the outputs corresponding to any fundamental material properties of interest. The latent dimensions therefore represent input-output correlations corresponding to each property it is trained on.

   In this work the original CGCNN architecture as presented in[20] was used, but the models were retrained on a more appropriate QMOF database[2] dataset curated further as presented in the main body of this paper. We modified the original CGCNN architecture to simultaneously train on multiple properties. This enhancement is motivated by the success of so-called transfer-learning in other areas[21,22] where designing or inversion of property to structure was also important. This also reduces the feature set dimensionality. However the testing performance with the simultaneously learned features was much poorer than the individually trained ones, for all the architectures examined. Hence the individually trained feature dimensions (i.e. the last layer of the QMOF trained CGCNNs for the two properties, namely, band gap and total energy of the structure) were retained at the end of the featurization process to go into the larger pool. It is likely that the structural representation of small molecules as is the domain of earlier works[21,22] is of a sufficiently lower dimension that is strongly cross-correlated across multiple properties, and therefore those networks benefit from simultaneous training approaches.

   Hyperparameter optimization was performed during the training using validation sets culled from the QMOF dataset. The CGCNN architecture was varied with 8, 16, 32 latent dimensions in the last layer and trained on the two property sets. The 32-dimensional latent space was chosen as providing the optimal training to testing performance across the QMOF database in both cases. The CGCNN feature set therefore consists of 292 features, nine sets of 32 features each set representing band gap, total energy, shear modulus, bulk modulus, Poisson ratio, Fermi energy, formation energy and energy per atom influences in structure respectively. On top there are additional features in terms of final predicted total energy, band gap for the material.

4. **Homology**: Persistent homology[23] captures deep topological effects inherent to the pore structure of the material. It incorporates the ability of internal spaces within materials to expand or contract to occlude or accommodate other molecules in transport. They are therefore an alternative pseudo-dynamic way of capturing shape effects inherent to crystalline structures. We embed these shape effects into atom-specific features following the procedure outlined in[24] for atom-specific persistent homology (ASPH). These features potentially reveal two-body and multi-body interactions in the homology. They are highly accurate predictors of formation energy[24]. Every feature we use is calculated as a persistence barcode from a point-clouds of atomic neighborhoods. For more details see the Methods section of[24]. There are 2,800 barcode features corresponding to 90 elements each with 35 barcodes related to minimum, maximum, mean, standard deviation and sum (x5) corresponding to 7 barcodes, namely, Betti-0, Betti-1 (start, stop and length) and Betti-2 (start, stop, length). The features generated from steps 1 through 4 are pooled and id-ed by their unique structural ID, to form the base feature pool for the rest of the active learning process. In that sense, the active learner is continuously forming nonlinear maps over subsets of features at every iteration based on new adsorption data. Every such new map informs the material selection for the next iteration

In additional to above described features, there are additional 32 features related to electronic structure of the elements in the material, atomic volume, atomic wight, density, melting point etc. Each of these have min, max, mean, standard deviation

and sum listed as separate features. In total the other features are 179.

Feature Type	Number of Features
Homology BarCodes	2800
JARVIS	1557
CGCNN	287
MAGPEI	132
Betti	35
Rest	179
**Total**	**4990**

Supplementary Table 4. Feature types and count from featurization workflow

Supplementary Note 2.2 Feature importance by Shapely Explanation

The models for CO_2 and N_2 adsorption free energy were analyzed for feature importance using Shapley explanation. The Shapley values are given in Supplementary Table 5 in decreasing order of important features. As it can be seen that the CO_2 adsorption free energy model is dominated by both geometric and electronic features while for N_2 adsorption free energy model dominated by mainly geometric features.

CO_2			N_2		
**Feature Name**	**Feature Type**	**Shapely Importance**	**Feature Name**	**Feature Type**	**Shapely Importance**
LCD	Geometric	1654.6	ASA_m2_cm3	Geometric	1257.0
MagpieDataAvg_dev	Electronic	1062.4	LCD	Geometric	968.2
GSmagmom_sum	Electronic	1035.3	NASA_m2_cm3	Geometric	770.0
GSmagmom_mean	Electronic	1007.2	jml_nn_70	Bond	355.4
AtomicVolume_min	Electronic	793.5	jml_C_0	Energy	313.8
AV_cm3_g	Geometric	688.6	totalenergy_latent_2	Energy	306.8
totalenergy_latent_0	Energy	650.9	jml_C_1	Energy	300.8
DipolePolarizability_std	Electronic	608.3	jml_nn_48	Bond	296.7
AV_VF	Electronic	603.1			
jml_nn_15	Bond	534.2			
jml_e3	Electronic	517.3			
jml_nn_49	Bond	498.8			
Column_max	Electronic	401.3			
ASA_m2_g	Geometric	393.1			

Supplementary Table 5. CO_2 (left) and N_2 (right) feature importance by Shapley explanation

Supplementary Note 3 Molecular-level simulations

The molecular-level simulations were performed with `RASPA-v2.0.47`.[25,26] The partial atomic charges were calculated using the EQeq method[27] as implemented in `pyeqeq-v0.0.9`. We used a hybrid[28] between UFF and DREIDING force fields for the framework atoms (see Supplementary Table 6), the TraPPE force field for the CO_2 and N_2 molecules, and the TIP5P model[29] for the H_2O molecule (see Supplementary Table 7).

All atoms in the framework were held fixed at their crystallographic positions. The number of unit cells used was different for each structure to ensure that the perpendicular lengths of the supercell were greater than twice the cutoff used. The cutoff for Lennard-Jones and charge-charge short-range interactions was $R_c = 12.8$ Å and the Ewald sum technique was applied to compute the long-range electrostatic interactions on a 0.1 Å grid with a relative precision of 10^{-6}. The Lennard-Jones potential was shifted to zero at the cutoff.

Supplementary Note 3.1 Grand Canonical Monte Carlo

For each temperature and pressure value, we performed 10,000 RASPA cycles. Each RASPA cycle corresponds to $\max(20, N_{\text{ads}})$ Monte Carlo steps, where N_{ads} is the number of adsorbed molecules in the simulation box at each point of the simulation. This way, each adsorbed molecule is subject to one move attempt per cycle (on average).

Atom	ε (K)	σ (Å)	Atom	ε (K)	σ (Å)	Atom	ε (K)	σ (Å)	Atom	ε (K)	σ (Å)
**B_**	47.804	3.582	**Se_**	216.376	3.591	**Ag_**	18.115	2.805	**Sm_**	4.026	3.137
**C_**	47.854	3.474	**Br_**	186.184	3.520	**Cd_**	114.73	2.538	**Eu_**	4.026	3.112
**H_**	7.649	2.847	**Ca_**	25.160	3.094	**Tl_**	342.176	3.873	**Gd_**	4.529	3.001
**N_**	38.948	3.263	**Sc_**	9.561	2.936	**Pb_**	333.622	3.829	**Tb_**	3.522	3.075
**O_**	48.156	3.034	**Ti_**	8.554	2.829	**Bi_**	260.658	3.894	**Dy_**	3.522	3.055
**P_**	161.024	3.698	**Cr_**	7.548	2.694	**Po_**	163.54	4.196	**Ho_**	3.522	3.038
**S_**	173.101	3.591	**Mn_**	6.542	2.638	**At_**	142.909	4.233	**Er_**	3.522	3.022
**F_**	36.482	3.094	**Fe_**	27.676	4.045	**Rn_**	124.794	4.246	**Tm_**	3.019	3.006
**I_**	256.632	3.698	**Co_**	7.045	2.559	**Cs_**	22.644	4.025	**Yb_**	114.73	2.990
**K_**	17.612	3.397	**Ni_**	7.548	2.525	**Ba_**	183.165	3.300	**Lu_**	20.631	3.243
**V_**	8.051	2.801	**Cu_**	2.516	3.114	**Hf_**	36.23	2.799	**Ac_**	16.606	3.099
**W_**	33.714	2.735	**Zn_**	27.676	4.045	**Ta_**	40.759	2.825	**Th_**	13.083	3.026
**Y_**	36.23	2.981	**In_**	276.760	4.090	**Re_**	33.211	2.632	**Pa_**	11.07	3.051
**U_**	11.07	3.025	**Sn_**	276.760	3.983	**Os_**	18.618	2.780	**Np_**	9.561	3.051
**Li_**	12.58	2.184	**Sb_**	276.760	3.876	**Ir_**	36.734	2.531	**Pu_**	8.051	3.051
**Be_**	42.772	2.446	**Te_**	286.824	3.769	**Pt_**	40.256	2.454	**Am_**	7.045	3.013
**Al_**	155.992	3.912	**Rb_**	20.128	3.666	**Au_**	19.625	2.934	**Cm_**	6.542	2.964
**T_**	155.992	3.805	**Sr_**	118.252	3.244	**Hg_**	193.732	2.410	**Bk_**	6.542	2.975
**Si_**	155.992	3.805	**Zr_**	34.721	2.784	**Fr_**	25.16	4.366	**Cf_**	6.542	2.952
**Cl_**	142.557	3.520	**Nb_**	29.689	2.820	**Ra_**	203.293	3.276	**Es_**	6.038	2.940
**Na_**	251.600	2.801	**Mo_**	28.179	2.720	**La_**	8.554	3.138	**Fm_**	6.038	2.928
**Mg_**	55.855	2.692	**Tc_**	24.154	2.671	**Ce_**	6.542	3.169	**Md_**	5.535	2.917
**Ga_**	201.280	3.912	**Ru_**	28.179	2.640	**Pr_**	5.032	3.213	**No_**	5.535	2.894
**Ge_**	201.280	3.805	**Rh_**	26.67	2.610	**Nd_**	5.032	3.186			
**As_**	206.312	3.698	**Pd_**	24.154	2.583	**Pm_**	4.529	3.161			

Supplementary Table 6. Lennard-Jones parameters describing well depths (ε) and diameters (σ) for framework atoms.

Atom	ε (K)	σ (Å)	q (e)
**C_co2**	27.000	2.800	0.700
**O_co2**	79.000	3.050	−0.350
**N_n2**	36.000	3.310	−0.482
**N_com**	——	——	0.964
**Ow**	89.633	3.097	0.000
**Hw**	——	——	0.241
**Lw**	——	——	−0.241

Supplementary Table 7. Lennard-Jones parameters describing well depths ε, well diameters σ and point charges q for molecule atoms.

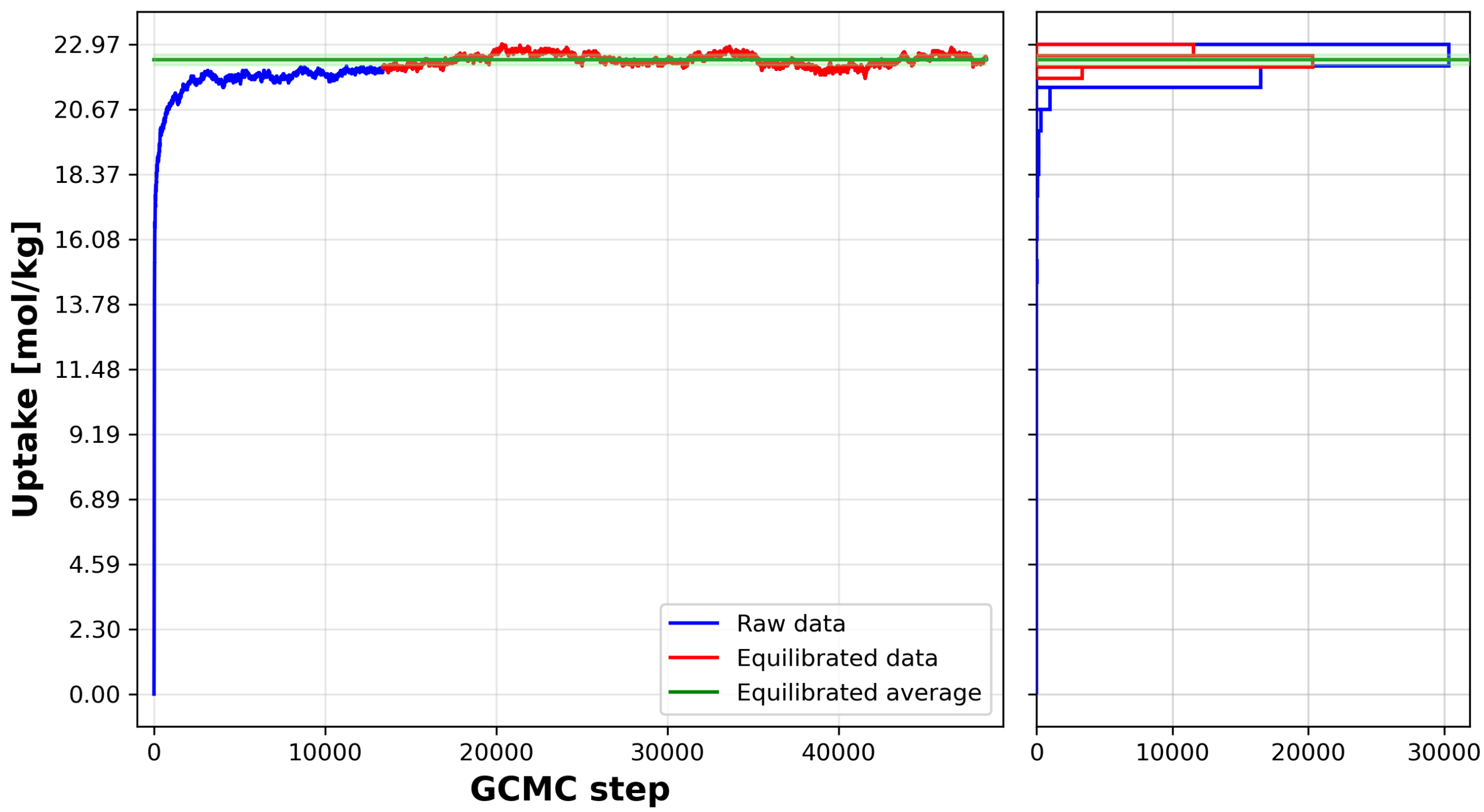

Supplementary Figure 3. Time series equilibration. The molecular uptake as a function of GCMC steps is treated as a time series (blue) and processed using the MSER rule. After the truncation point is determined, the equilibrated part of the time series (red) is averaged to obtain the representative thermodynamics observable (green).

In Supplementary Figure 3, we show how the resulting time series for the molecular uptake as a function of Grand Canonical Monte Carlo steps is processed by an equilibration routine using the Marginal Standard Error Rule (MSER)[30] as implemented in the pyMSER[31] open-source package. For each simulation, at a given temperature and pressure, an equilibrated average loading value is calculated alongside a representative uncertainty metric. We chose the uncorrelated standard deviation (uSD) as the representative uncertainty metric. We calculate it by taking the equilibrated part of the time series, as provided by the MSER method, and splitting it into chunks of length equal to the autocorrelation time t_{corr}. Then we take the average of each (uncorrelated) chunk and calculate the standard deviation of the collection of chunk averages.

We simulated single-component gas adsorption isotherms for CO_2, N_2 at $T = 298$, 323, 348 and 373 K, and from $P = 0.01$ to 50 bar at logarithmically spaced intervals. Similarly, H_2O isotherms were simulated at the same temperatures, but at pressures low enough to avoid condensation. The simulations at different pressures were chained, so that the final state of the simulation at pressure P_n was used as the initial state of the simulation at pressure P_{n+1}. This procedure allowed reaching equilibrium faster than if we started from an empty simulation box with zero molecules at each new pressure value.

Multi-site Langmuir models[32] were fitted to the simulated adsorption isotherm data with $S \in \{1,2,3\}$ sites using SciPy[33] optimization library for $i \in \{CO_2, N_2, H_2O\}$ species

$$q_i^{\mathrm{eq}}(P,T) = \sum_{j=1}^{S} q_{i,j}^{\mathrm{sat}} \frac{b_{i,j}(T) \cdot P}{1 + b_{i,j}(T) \cdot P}, \qquad (1)$$

where $q_{i,j}^{\mathrm{sat}}$ and $b_{i,j}(T)$ are the saturation capacity and equilibrium parameter associated with species i and site j, respectively. The S-site Langmuir model that achieved the lowest RMSE for CO_2 was selected to represent the simulated isotherms for all species, as shown in Supplementary Figure 4. The equilibrium parameters $b_{i,j}(T)$ can be modeled interchangeably by the Arrhenius[34] and Van't Hoff[35] equations by expressing the pre-exponential factor of the former as the temperature-independent term of the latter. For representing the isotherm at adsorption temperature, we calculate the free energy of adsorption, $\Delta G_{i,j}(30°\mathrm{C})$, using the estimated parameters from the Van't Hoff equation. Typically, at lower CO_2 partial pressure, such as in NGCC flue gas, the site with the lowest free energy of adsorption for CO_2 plays the most important role. The free energy values of this site are used in the active learning-based materials selection described in Supplementary Note 2. The resulting analytical expressions for the adsorption as a function of pressure and temperature were input to the process-level model equations with modifications to

account for multi-component adsorption as described by Supplementary Equation 6 as function of pressure, temperature and composition.

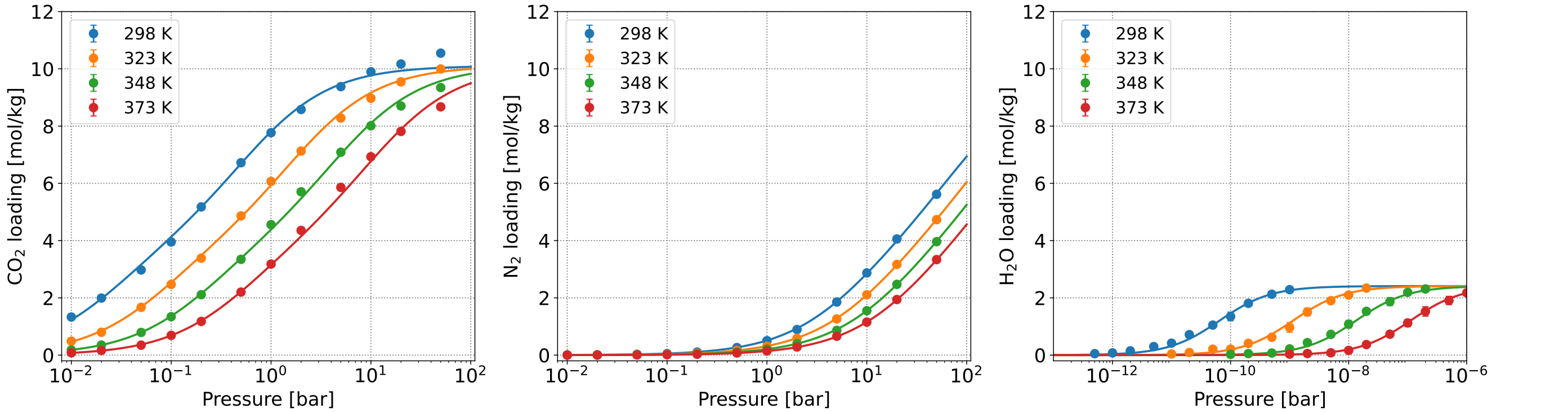

Supplementary Figure 4. Simulated isotherms and adjusted Langmuir models. In this example for material APAZOI, symbols represent the simulated adsorption values along an isotherm and lines represent the best fit Langmuir isotherm model, at different temperatures, for CO_2 (left), N_2 (center) and H_2O (right).

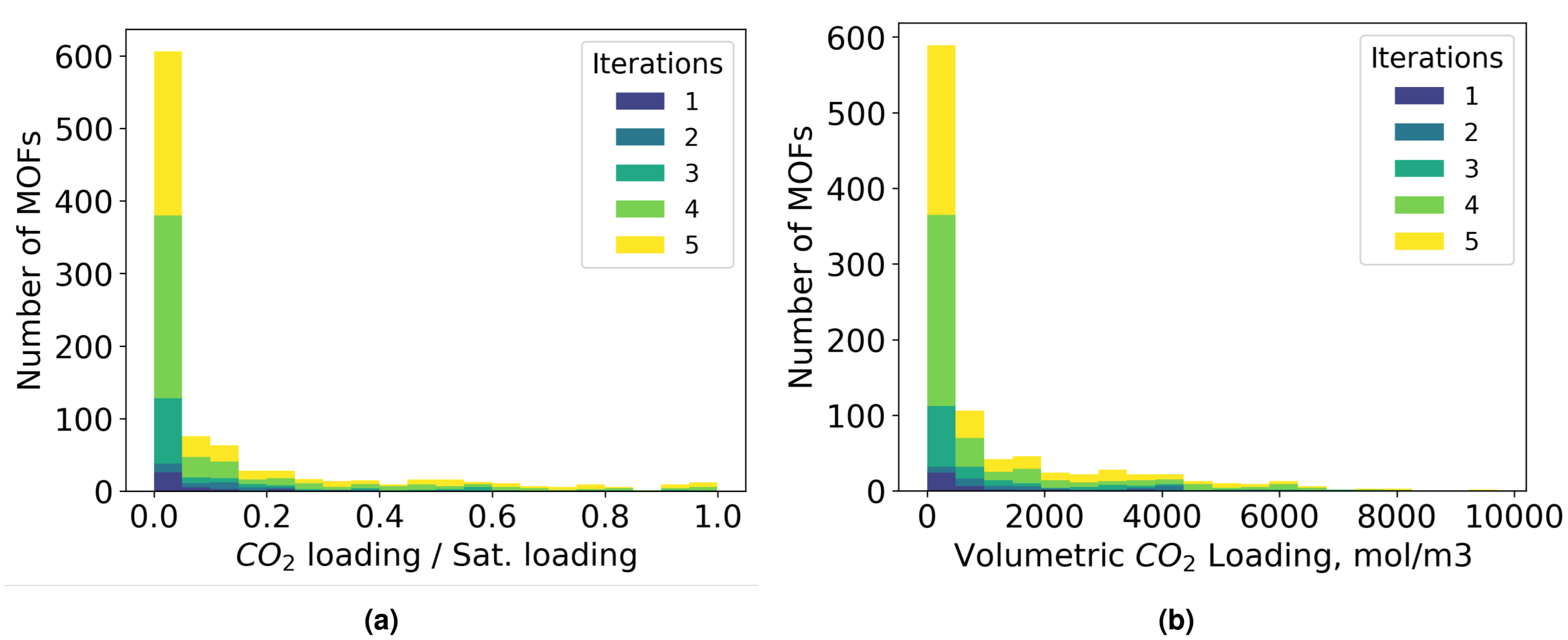

Supplementary Figure 5. Distribution of (a) fractional and (b) volumetric CO_2 loading calculated using isotherm model for 4% CO_2 NGCC flue gas at 30 °C. The stacked bars show the 982 recommended materials across 5 active learning iterations.

We have used the Widom particle insertion method[36] to probe the energy required for (or gained by) adding a probe molecule to the system, starting from an empty framework. CO_2, N_2 and H_2O were considered as probe molecules at temperatures $T = 298,\ 323,\ 348$ and 373 K. Each simulation performed 200,000 test insertions. From that, we have derived Henry constants at four temperatures, to which we have fitted ΔH and ΔS, from which we have then calculated the free energy of adsorption ΔG at 30 °C in Supplementary Table 11.

Supplementary Note 3.2 Molecular Dynamics

The Molecular Dynamics simulations for the calculation of diffusion coefficients were performed aiming for a constant, and finite, volumetric loading of adsorbed molecules following $1/R_c^3$. Given that $R_c = 12.8$ Å and the simulation box has at least $(2R_c)^3$ volume, this leads to a volumetric loading no larger than 792 mol/m^3, which translates to a low gravimetric loading of the order of 1 mol/kg. This led to different numbers of molecules per simulation box for each material, given that each structure had a different unit cell size. The NVT ensemble was used with a Nosé–Hoover thermostat, and the integration time step was 10 fs. We performed 1000 GCMC cycles for initialization, 9000 MD steps (90 ps) for equilibration and 5,000,000 MD steps (50 ns) for production. The mean squared displacement (MSD) was calculated based on the trajectories of the molecules by applying the n-order algorithm.[37]

Supplementary Note 3.2.1 Diffusion coefficient calculation

The microscopic diffusion coefficient can be computed using the Einstein equation[38]

$$D_\mu = \frac{1}{2d} \lim_{t\to\infty} \frac{\mathrm{d}}{\mathrm{d}t} \left\langle \frac{1}{N} \sum_{i=1}^{N} |\vec{r}_i(t) - \vec{r}_i(0)|^2 \right\rangle, \tag{2}$$

where N is the number of molecules (of a given type) inside the pore, t is the simulation time, $\vec{r}(t)$ is the position vector at time t, and d is the dimension of the system. The only requirement for the application of the Einstein equation is that the atoms or molecules in the system move randomly due to elastic collisions, *i.e.* Brownian motion.

One readily recognizes the term inside the angle brackets as the mean squared displacement (MSD) computed from the molecular trajectories $\vec{r}_i(t)$. Thus, the diffusion coefficient can be calculated from the slope of a linear fit of the $\mathrm{MSD}(t)$ curve according to

$$D_\mu = \frac{1}{2d} \frac{d}{dt} \mathrm{MSD}(t). \tag{3}$$

The *normal* diffusion regime is defined as the region in which the MSD changes linearly with time. But depending on the nature of the framework-molecule interactions, other (non-linear) diffusion regimes may manifest in the $\mathrm{MSD}(t)$ curve.[39] These are often called *anomalous* diffusion regimes and are described by a power law, $\mathrm{MSD}(t) = K_\alpha t^\alpha$ where K_α is the "generalized diffusion coefficient". Several examples of different diffusion regimes are illustrated on Supplementary Figure 6 and are listed below.

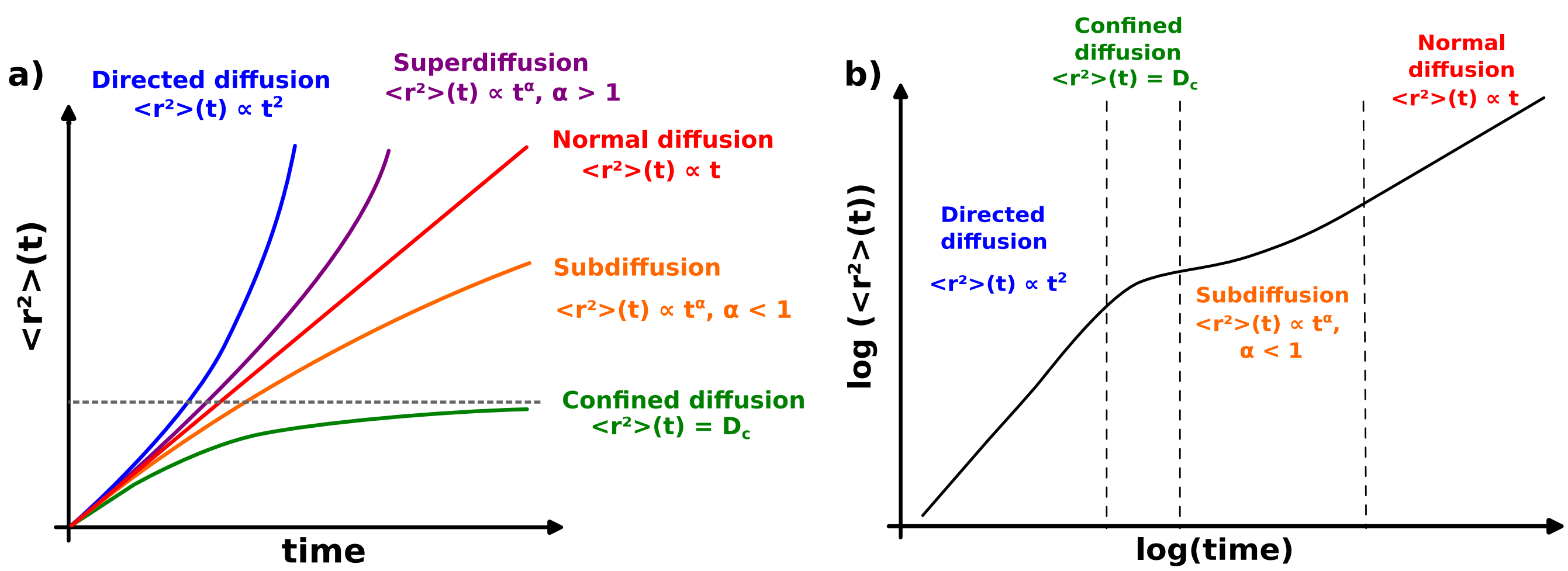

Supplementary Figure 6. Different diffusion regimes and their dependence on α. (a) MSD curve for different diffusion regimes and (b) log-log plot of the MSD curve for a typical molecular diffusion simulation, illustrating different regimes that may occur.[39]

- **Directed diffusion** ($\alpha = 2$): ballistic motion, similar to a particle moving at constant speed.
- **Superdiffusion** ($2 > \alpha > 1$): complex interplay between accelerating forces and dispersive interactions between the particles and its neighbors.
- **Normal diffusion** ($\alpha = 1$): regular Brownian motion.
- **Subdiffusion** ($0 < \alpha < 1$): dominated by dispersive interactions between the particle and its neighbors.
- **Confined diffusion** ($\alpha = 0$): when particles are confined to cages with no longe-range diffusion.

Given that both Supplementary Equation 2 and Supplementary Equation 3 only apply to the normal regime, one must first locate the MSD region that obeys this regime in order to extract the diffusion coefficient. This procedure is usually performed by visual inspection combined with simple heuristics. Although this approach might work when simulating a few materials, it is not compatible with high-throughput studies.

Here, we propose the following approach for the automated extraction of self-diffusion coefficients from the MSD curve:

1. Check if MSD is greater than the largest axis of the simulation box (λ); If not, consider "confined diffusion"; Otherwise...
2. Locate inflection points of log-log MSD(t) curve by taking its second derivative;
3. Split MSD(t) curve into segments at the inflection points;
4. Identify diffusion regime in each segment by fitting a linear slope;
5. Locate normal diffusion regime as the region with the slope closest to 1;
6. Calculate self-diffusion coefficient using Supplementary Equation 3 in the normal diffusion regime.

We illustrate in Supplementary Figure 7 the decision-tree algorithm used to obtain the diffusion coefficient. Supplementary Equation 3 is only applied to regions in which MSD $> \lambda$ and $\alpha \approx 1$. When a case of "confined diffusion" (MSD $< \lambda$ and $\alpha \approx 0$) is identified, the average of the MSD is taken as the confined diffusion coefficient D_c. Alternatively, when MSD $< \lambda$ but $\alpha \not\approx 0$, we conclude that the simulation does not contain enough time steps and needs to be run for longer. Finally, when MSD $> \lambda$ but $\alpha \not\approx 1$, we treat it as a case of anomalous diffusion and do not calculate any diffusion coefficient.

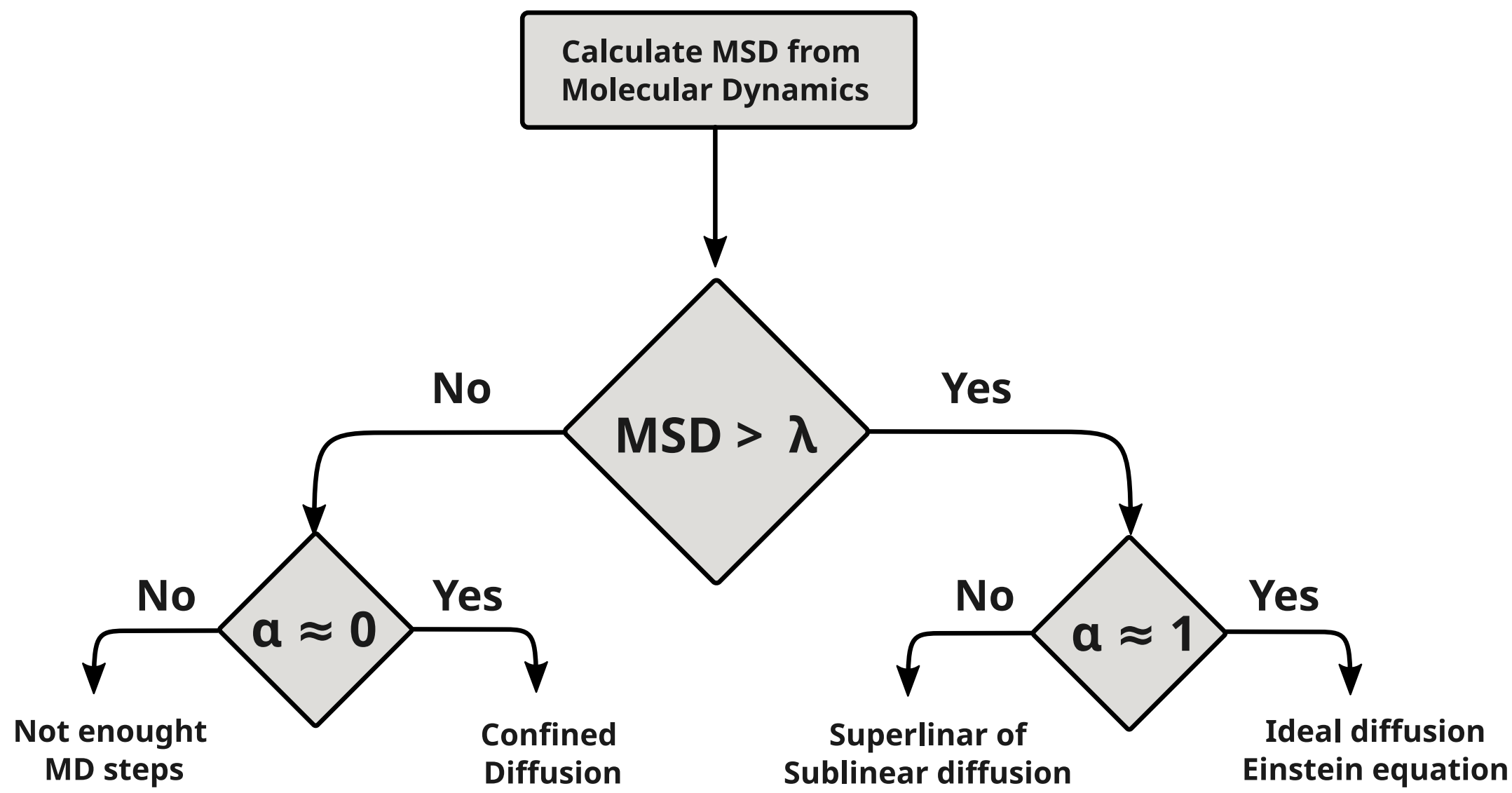

Supplementary Figure 7. Flowchart representing the algorithm used to identify diffusion regimes in the MSD curve. This decision tree is applied to each section of the MSD curve delimited by its inflection points. Here, α corresponds to the fit coefficient and λ represents the maximum perpendicular distance of the simulation box.

Supplementary Note 3.2.2 Validation against experiments

To validate our methodology for calculating the microscopic diffusion coefficient, we turn to the literature for experimental estimates. In Supplementary Table 8, we see the experimentally determined CO_2 diffusion coefficient for several porous materials. The conditions under which the simulations were performed always mimic those in the experiments reported in the references.

Our simulation results are generally of the same order of magnitude as the experimental benchmark, except for Zn_2(dobdc) and CID-Me. The former is overestimated by an order of magnitude, probably due to an incomplete treatment of the Zn open-metal site using a generic Lennard-Jones force field incapable of describing chemisorption. The latter is underestimated by several orders of magnitude, incorrectly classifying it as "confined diffusion". These two mismatches exemplify the limitations of the proposed methodology due to the choice of a non-bound, long-range classical potential. Although we are not able to experimentally validate all our simulations due to the scarcity of experimental data, the entries in Supplementary Table 8 provide an example of the types of behavior encountered. In rigid, physisorbent materials we expect to obtain agreement of simulated and experimental diffusion coefficients. For chemisorbents and flexible structures, however, we expect to overestimate and underestimate diffusion coefficients, respectively. Nevertheless, the proposed methodology could be enhanced by replacing the Lennard-Jones interatomic potential by a more accurate one, keeping all other computational steps in place.

Material	Experimental (m^2/s)	Simulated (m^2/s)	Reference
HKUST-1	1.7×10^{-9}	5.2×10^{-9}	Tovar *et al.*[40]
ZIF-8	1.9×10^{-10}	1.72×10^{-10}	Jannatabadi *et al.*[41]
Zn_2(dobdc)	1.5×10^{-9}	2.0×10^{-8}	Forse *et al.*[42]
Zn_2(dobpdc)	5.9×10^{-9}	1.13×10^{-8}	Forse *et al.*[42]
CID-Me	$10^{-13}-10^{-15}$	2.86×10^{-24}	Kurihara *et al.*[43]
D-MOF1	6.2×10^{-9}	5.83×10^{-9}	Peksa *et al.*[44]

Supplementary Table 8. Experimental and simulated CO_2 diffusion coefficients reported in the literature.

Supplementary Note 3.2.3 Distribution of diffusion coefficients for different adsorbates

We simulate the diffusion of CO_2 and N_2 molecules inside a host of selected materials. We plot in Supplementary Figure 8a the distribution of CO_2 and N_2 heats of adsorption extracted from the dominant site of the Langmuir model that best fits the single-component isotherms. The symbols are colored based on the balance between the microscopic diffusion coefficients: red for $D_\mu^{CO_2} \gg D_\mu^{N_2}$ and blue for $D_\mu^{CO_2} \ll D_\mu^{N_2}$. The vast majority of points lie below the parity line and, therefore, display higher adsorption affinity towards CO_2 (*i.e.* $-\Delta H_{CO_2} > -\Delta H_{N_2}$). On the other hand, the diffusion coefficient tends to decrease as the adsorption affinity increases. This negative correlation between adsorption and diffusion figures-of-merit generates competing effects at the process level and can hamper performance in materials with high CO_2/N_2 selectivity but low CO_2 diffusion.

In Supplementary Figure 8b, the $D_\mu^{CO_2}$ *vs.* $D_\mu^{N_2}$ marginal plot shows a bimodal distribution with a preponderance of $D_\mu^{N_2} \gtrapprox D_\mu^{CO_2}$ points (slightly above the parity line). The median diffusion coefficient is 2×10^{-9} m^2/s for CO_2 and 9×10^{-9} m^2/s for N_2. Although the greater part of the distribution lies between 10^{-14} and 10^{-6} m^2/s, approximately 13% of the materials have diffusion coefficients smaller than 10^{-18} m^2/s for either CO_2 (bright blue cluster), N_2 (bright red cluster) or both (faint red/blue cluster on the lower left).

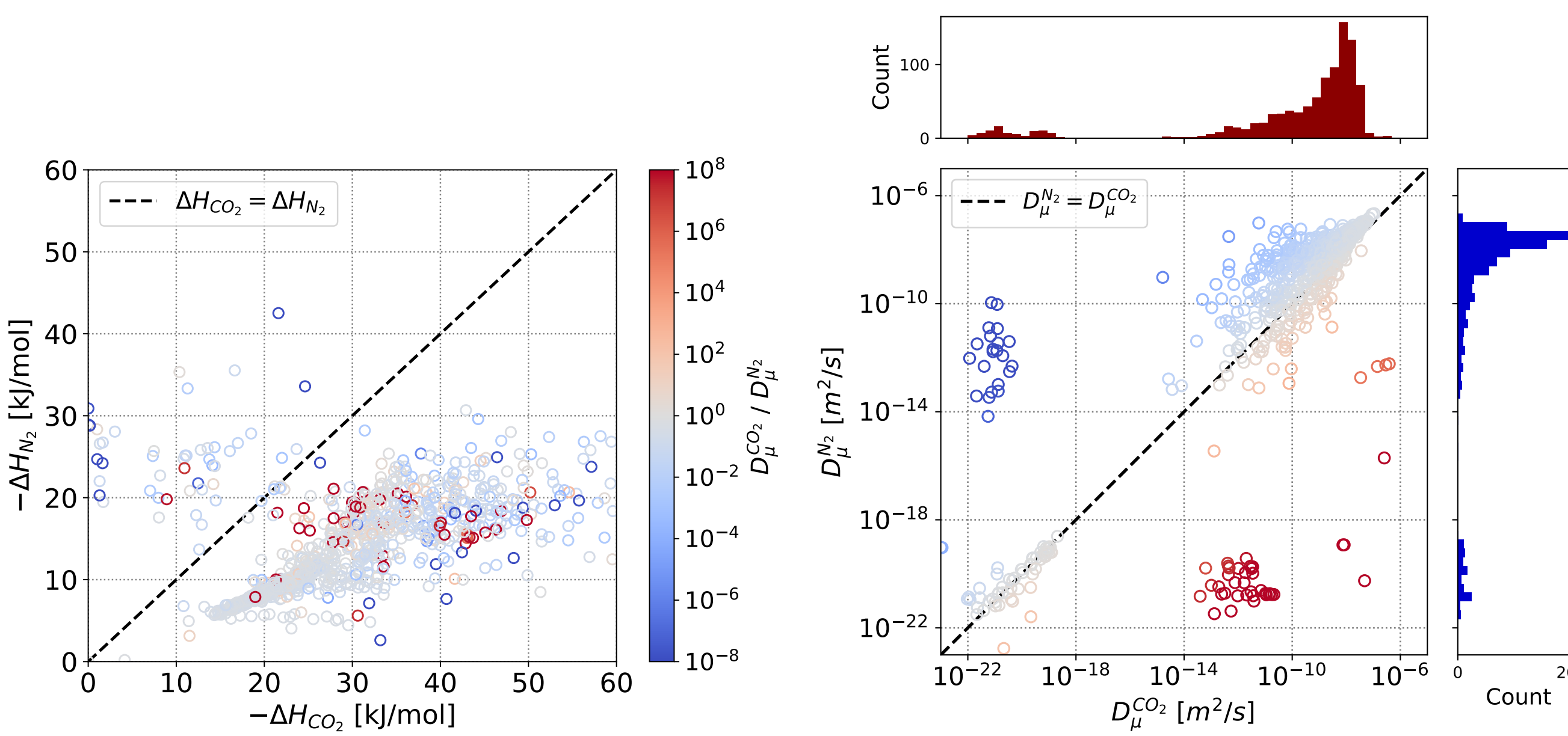

(a) **(b)**

Supplementary Figure 8. Distribution of CO_2 and N_2 diffusion coefficients. (a) Correlation between heat of adsorption and diffusion coefficient. Materials with high $\Delta H_{CO_2}/\Delta H_{N_2}$ ratio tend to display lower $D_\mu^{CO_2}/D_\mu^{N_2}$ ratio, which can hamper CO_2 capture productivity at process level. (b) Marginal plot of the bimodal distributions of diffusion coefficients for CO_2 and N_2, showing a slight preponderance of $D_\mu^{N_2} \gtrapprox D_\mu^{CO_2}$ values of the order of 10^{-9} m^2/s.

Supplementary Note 3.2.4 Temperature-dependent diffusion coefficients

Ideally, the procedure above should have been applied to, not one, but *many* independent trajectories representing an average of MSD(t) curves in order to decrease the statistical uncertainty of the diffusion coefficient so-obtained. To alleviate this

limitation, we perform molecular diffusion simulations at eight temperatures – from 348 to 698 K at regular intervals – and fit an Arrhenius-type equations to the data points, as shown in Supplementary Figure 9. The temperature-dependent diffusion model can be expressed as

$$D_\mu(T) = D_0 \exp\left(\frac{-E_a}{k_B T}\right) \tag{4}$$

where E_a denotes the energy barrier for the diffusion process, D_0 is the exponential pre-factor, k_B is the Boltzmann constant, and T the temperature. This equation can be linearised by taking the logarithm on both sides.

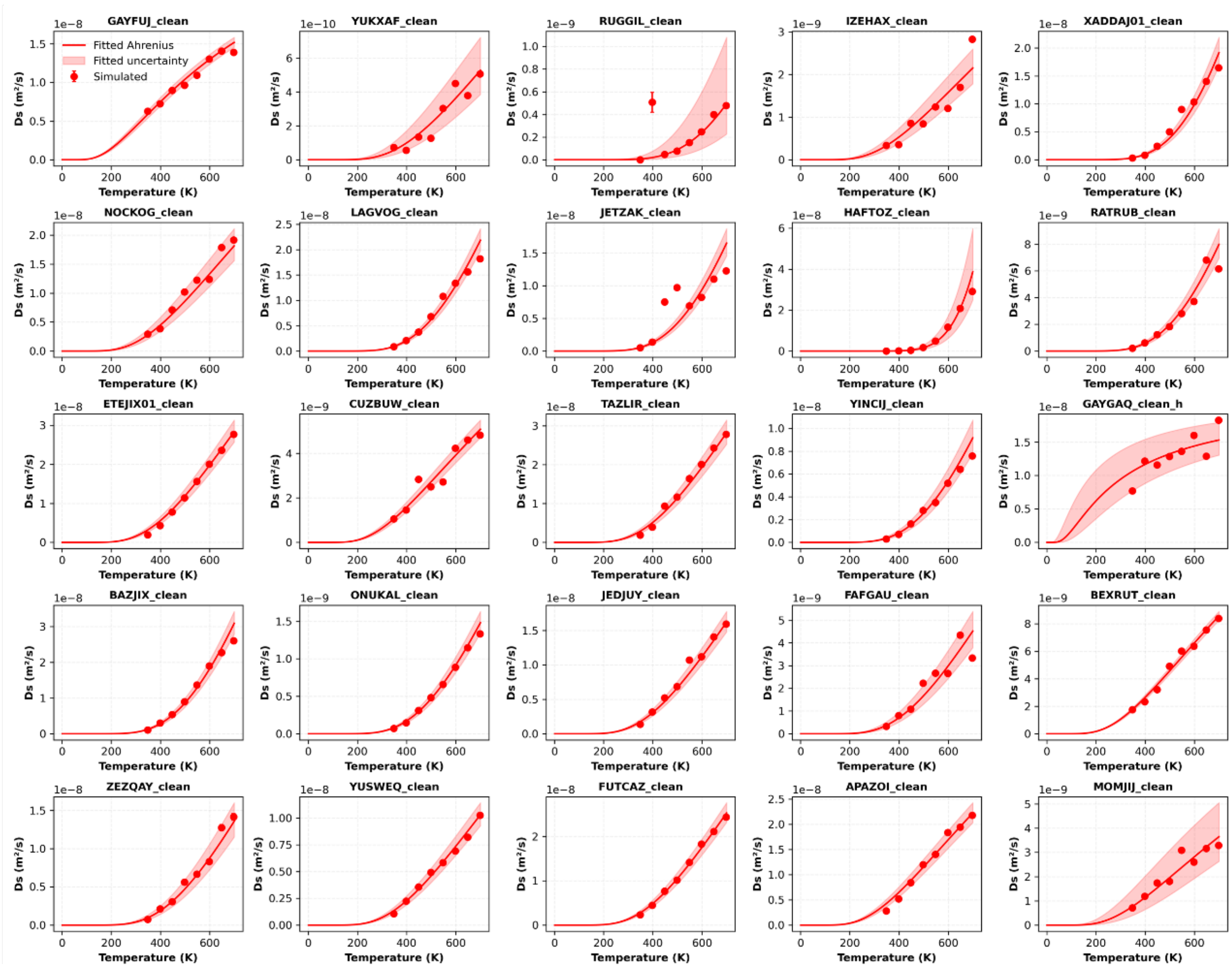

Supplementary Figure 9. Determination of temperature-dependent diffusion coefficients. Examples of simulated diffusion coefficients (symbols) and adjusted Arrhenius expressions (lines). The symbol error bars represent the uncertainty in the linear least-squares fit used to determine the diffusion coefficients with Supplementary Equation 3. The shaded area around the lines represent the linear least-squares fit uncertainty associated with the Arrhenius expression in Supplementary Equation 4.

The fit was performed using `scipy` version 1.7.4,[33] and incorporating the uncertainty as relative weights for the fit. The bounds $-\infty < E_a < 0$ and $0 \leq D_0 \leq 1$ were imposed to ensure the physical consistency of the model. The use of the uncertainty as relative weights in the fit was important because it de-emphasized outlier points gently, eliminating the need for data inspection and manual removal of outliers, which would introduce human bias and hamper reproducibility.

Materials with narrow pores, as well as materials with very high affinity for CO_2 due to open metal sites demonstrate thermally-activated diffusion. These materials display confined diffusion at lower temperatures and show a transition to normal diffusion at higher temperatures. To avoid such disparate diffusivity values, we intentionally remove any diffusivity below 10^{-19} m^2/s from the Arrhenius fit. Thus for some of the materials, the single component diffusivity values are extrapolated to low temperature from their high temperature behavior.

Supplementary Note 4 Process-based screening

We use a four-step temperature-swing adsorption (TSA) process model consisting of adsorption, purge, desorption and cooling steps, as depicted in Supplementary Figure 10, to screen carbon capture materials. The dynamic TSA cyclic process model was developed using gPROMS® Model Builder software.

Supplementary Note 4.1 Temperature-swing adsorption process

The contactor for the process is assumed to be a fixed column with a packed adsorbent bed, as illustrated in Supplementary Figure 10a. The heat transfer fluid flowing around the outer walls is always water, but at different temperatures, depending on the cycle step. Heating is achieved by simultaneously flowing steam and pressurized hot water, both at 105 °C, through the packed bed and heating jacket of the vessel, respectively. By comparing the effectiveness of indirect heat transfer between the heating jacket and direct heating by steam, we find that the heat transfer is dominated by the heating jacket, and the role of steam is reduced to that of a mere inert purge medium for desorbed CO_2. The system is cooled by pumping water at 30 °C through the heating jacket of the vessel, as well as purging the bed with room-temperature inert N_2. The temperature variation during the TSA cycle steps is shown in Supplementary Figure 10b.

The cycle steps detailed in Supplementary Figure 10c are described as follows. In the adsorption step, a flue gas stream consisting of 4.1% CO_2 and 95.9% N_2 enters the sorbent bed while cooling water flows through the outer jacket of the adsorption bed. We assumed dry flue gas as feed to simplify the process model and interpretation, with an implicit (not shown) moisture removal step before CO_2 capture. In this step, (mainly) CO_2 is adsorbed while the CO_2-depleted stream of N_2 flows out. In the subsequent purge step, the bed is heated as previously described, leading to N_2 desorption and purge from inter-particle bed volume. The N_2-rich purged gas is discarded. In the following desorption step, due to continued heating (up to 105 °C), stronger-bound CO_2 is desorbed and collected from the outlet as product alongside the steam used for heating the bed. In order to represented steam as a purge medium with reasonably competitive isotherm, we have resorted to representing the steam (H_2O) isotherm and the water diffusivity as if they were the same as in CO_2. The main purpose of the simplifying assumptions is to represent the steam/energy requirement at screening-level accuracy. This product stream is cooled to remove the steam/water before compressing CO_2 to required pressure for sequestrations. Both of these steps are not shown or accounted for in the process based screening. In the cooling step, the bed is brought down to 35 °C as previously described. During cooling, the bed is dried to remove H_2O using N_2 as inert stream. Since the cooling step is relatively small, the N_2 required for this step can be sourced from effluent of the adsorption step. Finally, the dry and cool bed undergoes cyclic TSA process again until cyclic steady state is achieved, based on having less than 0.1% cycle-to-cycle variation in the CO_2 purity and recovery.

The packed bed is modeled as one-dimensional, including heat and mass transfer. The effective mass transfer coefficient[45] is defined as

$$K_{\text{eff}} = \frac{15}{\left(\frac{R_p^2}{D_p^*} + \frac{R_\mu^2}{D_\mu}\right)}, \tag{5}$$

where the pellet radius $R_p = 2$ mm and effective pellet diffusivity $D_p^* = 5 \times 10^{-8}$ m^2/s represent the macropore (or intercrystalline) diffusion ignoring external film resistance. The crystallite radius R_μ and diffusivity D_μ represent the micropore (or intracrystalline) diffusion.

Mass balance is simulated for the various phases according to Supplementary Equation 6. The meaning and values of the variables and parameters are described in Supplementary Table 9. The effective linear driving mass transfer coefficient (K_{eff}) is described as Supplementary Equation 5. This equation ignores pellet film resistance as well as does simplifying assumptions for pellet void fraction (ε_p) and thermodynamic correction (q^*/c) to enable consistent comparison between constant diffusivity and mass transfer with consideration for molecular diffusion. We equate the temperature dependent self-diffusivity to transport diffusivity under the approximation of low loading. To justify this, we have calculated equilibrium volumetric CO_2 loadings using a multi-component isotherm model for all 982 MOFs. For 692 of them, the calculated equilibrium loading of CO_2 is lower than the 792 mol/m^3 loading threshold provided in Supplementary Note 3.2. The distribution of the equilibrium volumetric CO_2 loading is plotted in Supplementary Figure 5b.

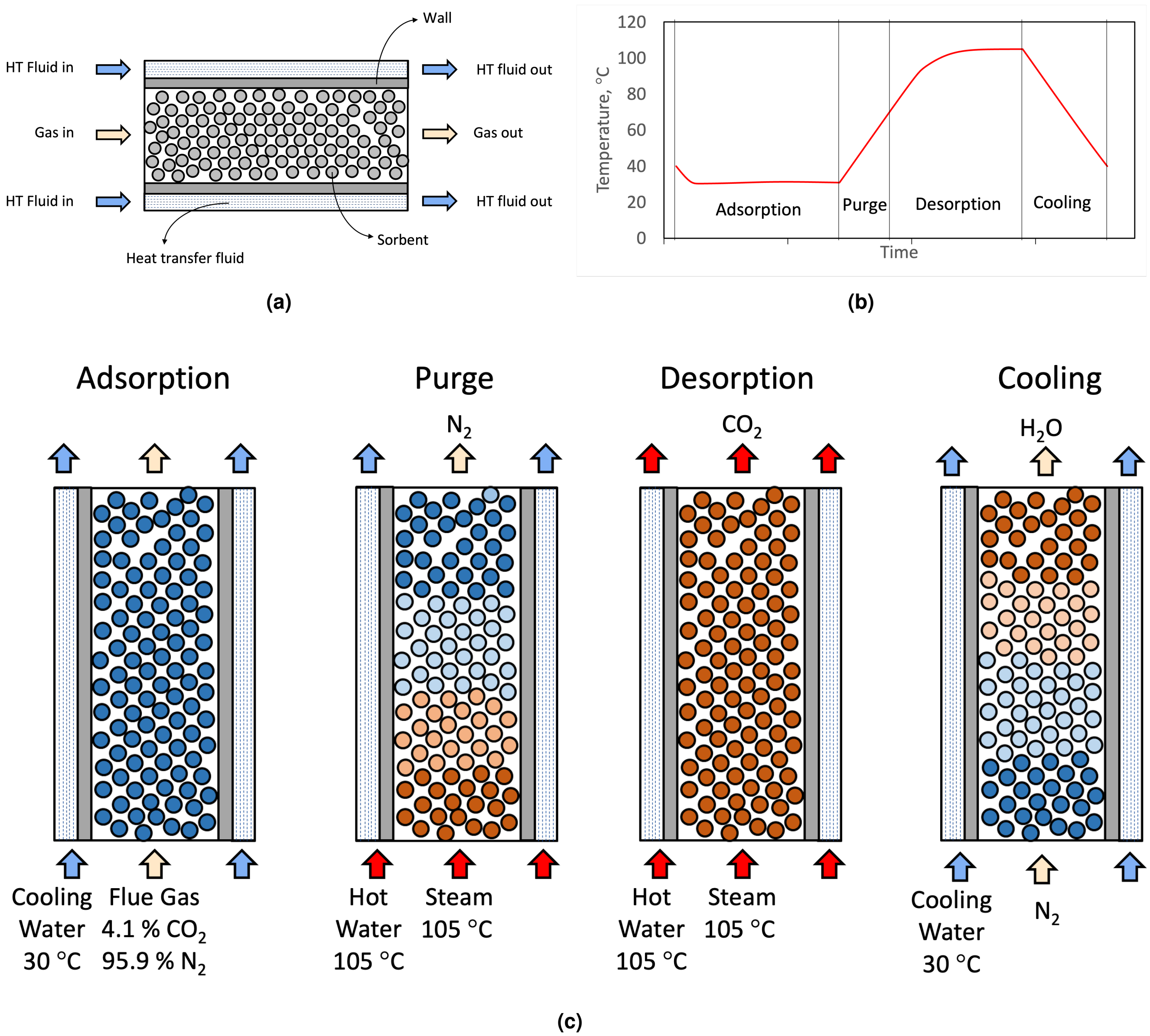

Supplementary Figure 10. Temperature-swing adsorption (TSA) process model. (a) Illustration of the sorbent bed in contact with the flue gas. (b) Variation of bed temperatures during TSA cycle. (c) TSA cycle steps, including adsorption, purge, desorption and cooling. Purge and desorption steps use steam as the energy source. All steps use water, albeit at different temperatures, as heat-transfer fluid.

$$
\begin{aligned}
\frac{\partial c_i}{\partial t} &= D_{\mathrm{ax}} \frac{\partial^2 c_i}{\partial z^2} - \frac{\partial (u_g c_i)}{\partial z} - \frac{(1-\varepsilon)\rho_s}{\varepsilon} \frac{\partial q_i}{\partial t} \\
\frac{\partial q_i}{\partial t} &= \sum_{j=1}^{S} \frac{\partial q_{i,j}}{\partial t} \\
\frac{\partial q_{i,j}}{\partial t} &= K_{\mathrm{eff}} \left(q_{\mathrm{eq},i,j} - q_{i,j} \right) \\
D_p^* &= D_p \frac{\varepsilon_p}{\rho_p} \frac{c}{q^*} \\
q_{\mathrm{eq},i,j} &= \frac{q_{\mathrm{sat},i,j}\, b_{i,j} \cdot c_{g,i}}{1 + \sum_k b_{j,k} \cdot c_{g,k}} \\
b_{i,j} &= \exp\left(-\frac{\Delta H_{\mathrm{ads},i,j} - T\Delta S_{\mathrm{ads},i,j}}{RT} \right)
\end{aligned} \tag{6}
$$

The energy balance equations for the various phases and components are described by Supplementary Equation 7 using the variables and parameters defined in Supplementary Table 9. For each material, we used a machine-learning model[46] to calculate the heat capacity at seven representative temperature points, ranging from 250 K to 400 K. The calculated heat-capacity values were fit with a fourth-order polynomial, which is used in the process model for calculating temperature-dependent heat capacities during the TSA-cycle simulations. The properties of the gas phase (subscript g), sorbent phase (subscript s), wall of the contactor shell (subscript w) and the external heat transfer fluid (subscript e) are modelled as follows.

$$
\begin{aligned}
\frac{\partial T_g}{\partial t} &= \frac{1}{\varepsilon \rho_g C_g}\left[\lambda_g \frac{\partial^2 T_g}{\partial z^2} - \varepsilon \rho_g C_g u_g \frac{\partial T_g}{\partial z} + \frac{6(1-\varepsilon)}{2R_p} h_{g,s}(T_s - T_g) + \frac{\pi d_{is} h \varepsilon}{V_s} h_{g,w}(T_w - T_g) + \frac{\pi d_{ot} h_{nT} \varepsilon}{V_s} h_{g,w} t\right] \\
\frac{\partial T_s}{\partial t} &= \frac{1}{(1-\varepsilon)\rho_s C_s}\left[\lambda_s \frac{\partial^2 T_s}{\partial z^2} - (1-\varepsilon)^2 \rho_s^2 C_s \sum_{i,k} \frac{\partial q_{i,k}}{\partial t} \Delta H_{i,k} + \frac{6(1-\varepsilon)}{2R_p} h_{g,s}(T_g - T_s) + \frac{\pi d_{is} h (1-\varepsilon)}{V_w} h_{s,w}(T_w - T_s)\right] \\
\frac{\partial T_w}{\partial t} &= \frac{1}{\rho_w C_w}\left[\lambda_w \frac{\partial^2 T_w}{\partial z^2} + \frac{\pi d_{is} h \varepsilon}{V_w} h_{g,w}(T_g - T_w) + \frac{\pi d_{is} h (1-\varepsilon)}{V_w} h_{s,w}(T_s - T_w) + \frac{\pi d_{os} h}{V_w} h_{e,w}(T_e - T_w)\right] \\
\frac{\partial T_e}{\partial t} &= \frac{1}{\rho_e C_e}\left[\lambda_e \frac{\partial^2 T_e}{\partial z^2} - \rho_e C_e u_e \frac{\partial T_e}{\partial z} + \frac{\pi d_{os} h}{V_e} h_{w,e}(T_w - T_e)\right]
\end{aligned}
\tag{7}
$$

Momentum balance for the column is solved using Ergun equation as shown in Supplementary Equation 8 where velocity and viscosity of gas are changing along the axis of the column. The meaning and values of the variables and parameters are described in Supplementary Table 9.

$$
-\frac{\partial P}{\partial z} = 150 \frac{\mu_g}{(2R_p)^2} \frac{(1-\varepsilon)^2}{\varepsilon^3} (\varepsilon u_g) + 1.75 \frac{\rho_g}{2R_p} \frac{(1-\varepsilon)}{\varepsilon^3} (\varepsilon u_g)^2 \tag{8}
$$

Supplementary Note 4.2 Process-level performance metrics

To illustrate the output of the process model we have selected 4 example MOFs, namely MOCHIW, BETGUE, MOCHIW and EVOMOR. These MOFs have been selected to show the impact of the adsorption capacity (isotherm) and diffusion coefficient on the performance and rank order of the MOFs. Out of these 4 selected MOFs, MOCHIW and MOCHIW are higher performing (Higher Ranked) MOFs and BETGUE and EVOMOR are lower performing (lower rank order) MOFs. MOCHIW and BETGUE show lower sensitivity towards crystal size while MOCHIW and EVOMOR show higher sensitivity towards crystal size due to diffusion limitation.

We show structures and isotherm information for these 4 MOFs in Supplementary Figure 11. The isotherm plots include the simulated CO_2, N_2 and H_2O adsorption capacities for these MOFs at multiple pressures and 4 different temperatures. The fitted isotherm model is shown on the same plot. Goodness of fit (Root Mean Square Error - RMSE) is used to The higher performing MOFs MOCHIW and MOCHIW, have significant capacity at 0.041 bar of CO_2 partial pressure while MOFs BETGUE and EVOMOR have lower CO_2 adsorption capacity at lower partial pressures.

In Supplementary Figure 12, we show diffusion constants from the molecular dynamic simulations performed for above mentioned MOFs and fitted activated diffusion model by lines. MOCHIW and BETGUE show higher diffusivity for both CO_2 and N_2 – with diffusion constant between 10^{-9} to 10^{-8} m^2/s depending on temperature. On the other hand MOCHIW and EVOMOR show limited diffusivity for both CO_2 and N_2. For MOCHIW, the diffusivity values based on higher temperature simulations, are 10^{-14} to 10^{-12} m^2/s. For EVOMOR diffusivity values are nearly very low (10^{-20} to 10^{-19} m^2/s.

In Supplementary Figure 13, we show cyclic simulation output in terms of the mole fraction of gas exiting the TSA column for CO_2, N_2 and H_2O. We also show column temperature at the exit of the adsorption column as a function of cycle time. The cycle time are shown by vertical lines on the cyclic profile. For MOCHIW and BETGUE the cyclic profile and process based performance of the CO_2 separation is same irrespective of the crystal size with molecular diffusion or with constant pellet diffusion. Hence, we report only cyclic simulation profile for constant diffusivity. Comparing Supplementary Figure 13a and Supplementary Figure 13b, MOCHIW with higher capacity has longer adsorption step while BETGUE with lower CO_2 capacity has shorter adsorption step. For MOCHIW and EVOMOR due to lower microscopic diffusivity, show cyclic simulation profile changing as a function of crystal size showing separation deteriorating with increasing crystal size. Comparing constant diffusivity cyclic profiles of MOCHIW (Supplementary Figure 13c and EVOMOR (Supplementary Figure 13e), they show similar behavior as of MOCHIW and BETGUE. However, comparing cyclic profiles for constant diffusivity (Supplementary

[1] Ideal gas law.
[2] Based on ideal crystal density.
[3] Machine-learning model from Moosavi et al.[46]

Supplementary Table 9. Process Model Parameters: Notations and values

Symbol	Description	Value(s)
	**Bed Geometry**	
	Bed Length	1 m
d_{is}	Packed Bed inside Diameter	3.00 cm
d_{os}	Packed Bed outside Diameter	3.18 cm
d_{ot}	Heat Exchange Jacket inside Diameter	3.36 cm
$V_s \mid V_w \mid V_e$	Total volume of {sorbent bed, wall, HTF}	Based on geometry
ε	Bed void fraction	0.35
	**Conditions and TSA Cycle Details**	
	Bed Outlet Pressure	1.0 bar
	Cycle Step Time (Ads I Purge I Des I Cool)	Determined by Optimizer
	Feed Gas Type (Ads I Purge I Des I Cool)	Flue I Steam I Steam I Nitrogen
	Feed Gas Flow Rate (Ads I Purge I Des I Cool)	200 I 60 I 60 I 200 cm^3/s
	Feed Gas Temperature (Ads I Purge I Des I Cool)	30 I 105 I 105 I 30 °C
	Heat Exchange Fluid Temperature (Ads I Purge I Des I Cool)	30 I 105 I 105 I 30 °C
$\rho_g \mid \rho_s \mid \rho_w \mid \rho_e$	Density of the {gas I sorbent I wall I HTF}	{[1] I [2] I 8 I 0.866} g/cm^3
$T_g \mid T_s \mid T_w \mid T_e$	Temperature of the {gas I sorbent I wall I HTF}	Output of Supplementary Equation 7
u_e	Inlet Exchange Fluid Velocity	0.2 m/s
u_g	Gas interstitial velocity	
P	Local pressure in sorbent bed	Output of Supplementary Equation 8
μ_g	Viscosity of gas	1.9^{-5} Pa.s
	**Sorbent Details and Mass Transfer**	
R_p	Sorbent particle radius	2 mm
R_μ	Sorbent crystal radius	0.5, 5, 50 µm
D_p	Sorbent intra-particle diffusivity	
D_p^*	Sorbent intra-particle effective diffusivity	5×10^{-8} m^2/s
D_μ	Sorbent intra-crystal diffusivity	
K_{eff}	Effective mass transfer coefficient	Output of Supplementary Equation 5
D_{ax}	Axial dispersion coefficient	5×10^{-7} m^2/s
	**Heat Transfer**	
$\lambda_g \mid \lambda_s \mid \lambda_w \mid \lambda_e$	Thermal conductivity of the {gas I sorbent I wall I HTF}	{0.03 I 0.5 I 15 I 0.13} W/(m.K)
$h_{g,s} \mid h_{g,w} \mid h_{s,w} \mid h_{e,w}$	Heat transfer coef {gas-sorbent I gas-wall I sorbent-wall I HTF-wall}	200 $W/(m^2.K)$
$C_g \mid C_s \mid C_w \mid C_e$	Heat Capacity of the {gas I sorbent I wall I HTF}	{1000 I [3] I 466 I 3075} J/kg/K
ρ_w	Density of the contactor's wall	8 g/cm^3
ρ_e	Density of the external heat transfer fluid	0.866 g/cm^3
	**Adsorption Isotherm**	
q_i	Concentration of component i in the adsorbed phase	Output of Supplementary Equation 6
c_i	Concentration of component i in the gas phase	Output of Supplementary Equation 6
$q_{\text{eq},i}$	Equilibrium concentration of component i in the adsorbed phase	Output of Supplementary Equation 1
$b_{i,j}$	Langmuir isotherm based adsorption affinity	Output of Supplementary Equation 6
$\Delta H_{\text{ads},i,j}$	Fitted enthalpy of adsorption for site i and species j	non-linear optimization
$\Delta S_{\text{ads},i,j}$	Fitted entropy of adsorption for site i and species j	non-linear optimization
$i \mid k$	Index that spans adsorbates	$i = \{CO_2, N_2, H_2O\}$
j	Index that spans Langmuir sites	$0 < j <= S$
S	Number of Langmuir sites considered	$S = \{1,2,3\}$

Figure 13c) and variable diffusivity (Supplementary Figure 13d) for MOCHIW, shows the cycle becoming shorter with earlier CO_2 breakthrough reducing the separation performance with inclusion of the microscopic diffusivity. Similarly, poorly performing MOF EVOMOR cycle becomes even shorter with the inclusion of the diffusivity. The short cycle due to very low diffusivity of CO_2 makes concentration and temperature arbitrary from the process optimization. For EVOMOR with diffusivity

considerations (Supplementary Figure 13f, the CO_2 breakthrough is observed very early in shortest adsorption step along with N_2 leading to no selectivity due to very low diffusivity, thereby limiting the possible purity or recovery in cyclic separation process.

The process modeling outcome also includes the trade-offs between the process-based figures of merits (i.e., CO_2 purity, recovery, specific energy consumption and volumetric productivity) reported in Supplementary Figure 14. The energy requirement is calculated by integrating the total energy input from external heating jacket and specific energy of steam used to purge N_2 and CO_2, during N_2 purge and CO_2 desorption steps, respectively. Our specific energy calculation does not include any additional contribution, including CO_2 compression, nor pump or blower energy. As shown in Supplementary Figure 14a and Supplementary Figure 14b, the purity vs. recovery and specific energy vs productivity are nearly unchanged as a function of diffusivity. In the case of MOCHIW (Supplementary Figure 14c), the purity vs. recovery Pareto fronts show gradual shift from top right corner to bottom left, moving from 1 µm crystal size to 10 µm and 100 µm. The maximum recovery possible decreases from near 98% for 1 µm crystal, to 89% for 10 µm, to 18% for 100 µm crystal. For EVOMOR, due to very poor diffusivity, the separation performance of MOF goes from good to very poor by just changing constant diffusivity to any crystal size as shown in Supplementary Figure 14d.

A representative MOF ranking is given in Supplementary Table 10 for the 20 highest-ranked MOFs by volumetric productivity at constant K_{eff}. For the same set of MOFs, the rank-order by volumetric productivity is also provided for crystallite diameters R_μ =1 µm, 10 µm, and 100 µm, respectively. These rankings account for structure-dependent, molecular diffusivity. As examples of the influence of diffusivity and crystallite diameter on rank-order, MOCHIW maintains second place in the ranking and becomes the highest-ranked MOF at R_μ =100 µm while (BOHGOU) drops out of the top-20 list. The MOF structure MOCHIW ranks successively lower as R_μ increases, falling from rank #12 to #495 at R_μ =100 µm.

Supplementary Table 10. Top-20 MOF ranked by volumetric productivity at a 95% CO_2-purity level for various crystallite diameters R_μ

MOF Name	K_{eff}=const.	R_μ =1 µm	R_μ =10 µm	R_μ =100 µm
BOHGOU	1	1	1	177
MOCHIW	2	2	2	1
NATXIR	3	3	3	2
CDLGLU11	4	698	695	721
VASFED	5	4	4	4
CAKXET	6	5	5	19
AZAFIS	7	6	6	3
MOCJAQ	8	7		
ESUMOU	9	697	692	675
CDLGLU01	10			
MUXHIW01	11	8	59	
MOCHES	12	11	88	495
IKETAU	13	9	7	9
RATVEP	14	10	9	21
MOCHIW	15	17	138	485
SUKREX	16	12	8	5
IPIJUM	17	13	10	6
NARMUR	18	14	11	12
MOCJEU	19	15	18	
VIDXOX	20	16	12	7

Supplementary Note 4.3 Water affinity of top performing MOFs for dry flue gas separation

In our screening study, we have used dry flue gas to screen MOFs. To estimate the affinity of the screened MOFs towards water, we have calculated the Henry region free energy of adsorption for CO_2, N_2, H_2O and compared them to well-known CALF-20[47], see Supplementary Table 11. Given that CO_2/N_2 selectivity and CO_2 capacity were included as criteria in the active learning material selection, but water affinity was not, the screening steered us towards a certain type of materials. The top 20 MOFs reported in our study have higher CO_2/N_2 selectivity ($\Delta G_{N_2} - \Delta G_{CO_2}$) than CALF-20, but also much higher water affinity (ΔG_{H_2O}) . This result reaffirms the inherent tradeoff between CO_2/N_2 selectivity and water affinity. In this work, we show that multiple MOF structures can achieve 90% recovery, and 95% purity target due to high selectivity under dry flue gas conditions.

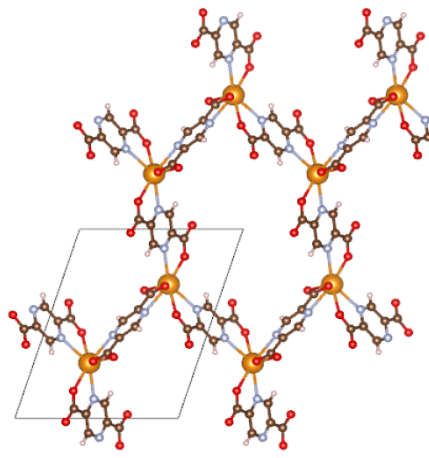

(a) VASFON

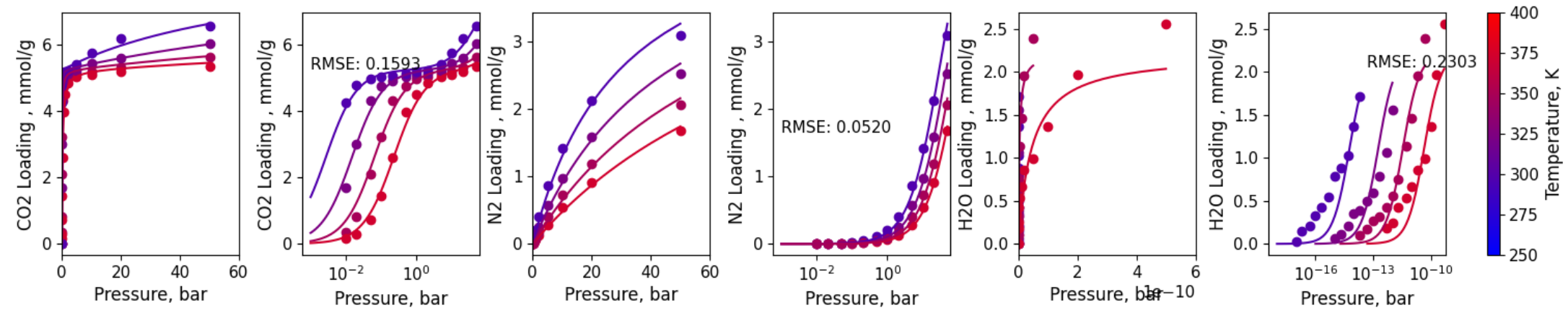

(b) CO_2, N_2 and H_2O adsorption isotherms for VASFON.

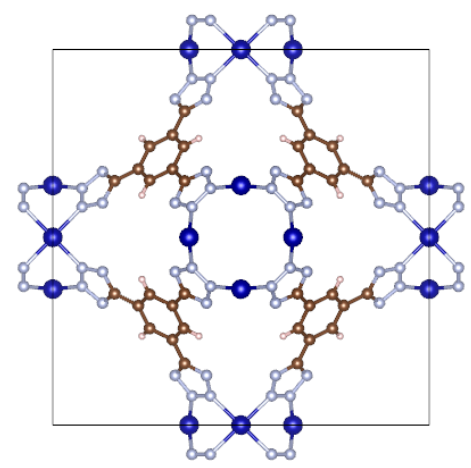

(c) BETGUE

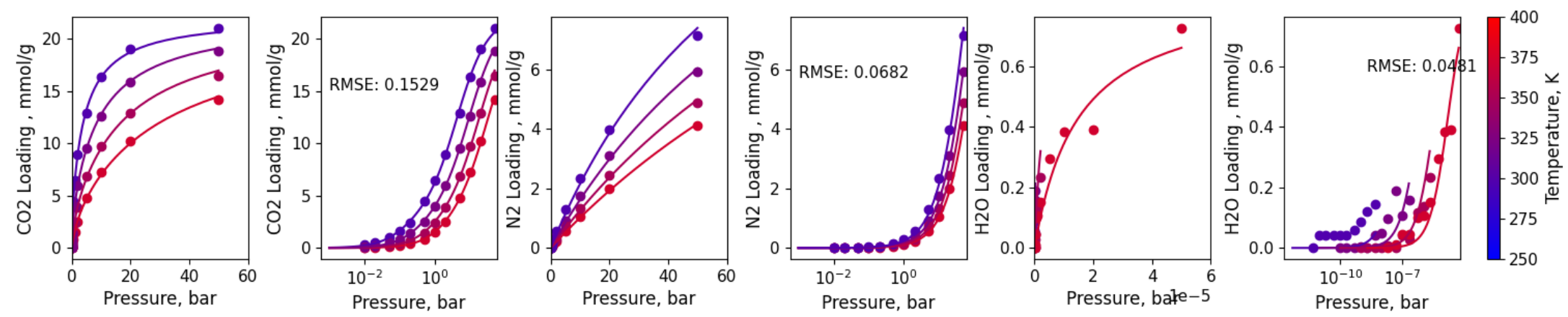

(d) CO_2, N_2 and H_2O adsorption isotherms for BETGUE.

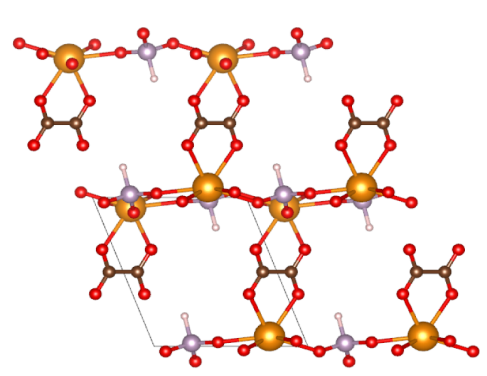

(e) MOCHIW

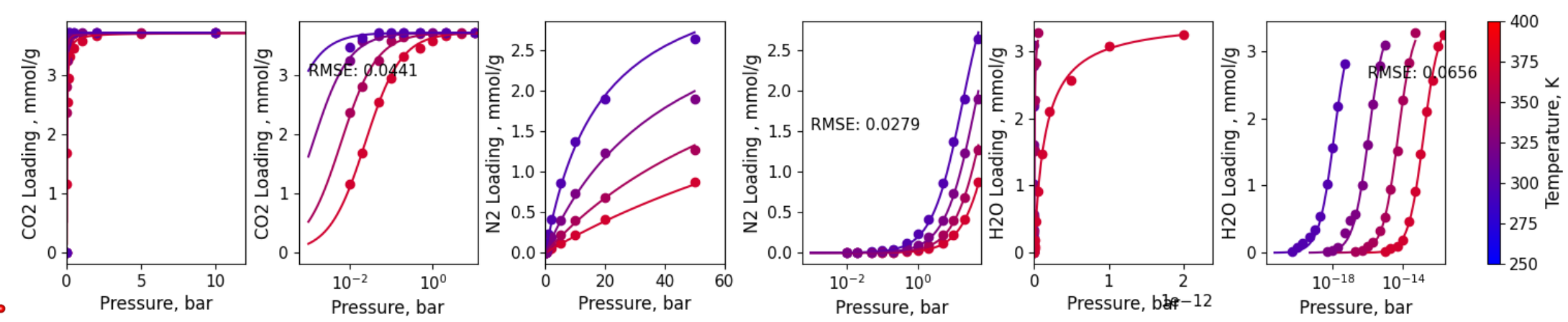

(f) CO_2, N_2 and H_2O adsorption isotherms for MOCHIW.

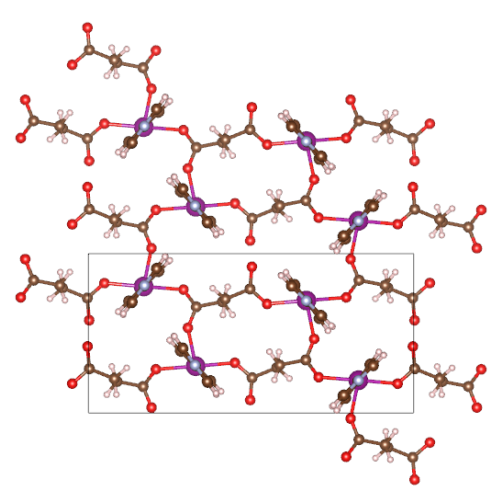

(g) EVOMOR

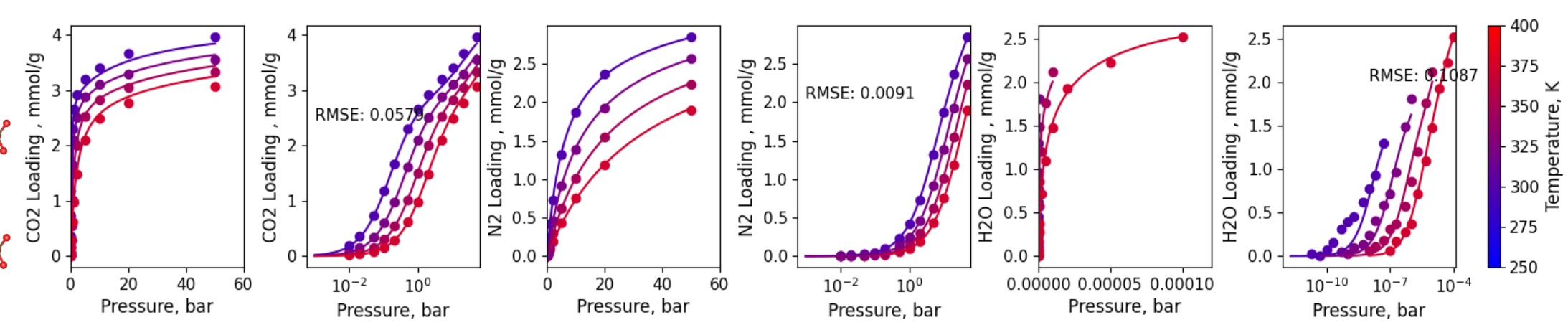

(h) CO_2, N_2 and H_2O adsorption isotherms for EVOMOR.

Supplementary Figure 11. MOF Structures and Isotherms (a) VASFON, (c) BETGUE, (e) MOCHIW and (g) EVOMOR structures and their associated adsorption isotherms (b), (d), (f) and (h), respectively. CO_2, N_2 and H_2O isotherm data (filled circles) and fitted models (lines).

To translate the Henry-region water affinities into adsorption isotherm data, we have simulated water adsorption isotherms for all MOFs reported in this manuscript. We have used a re-optimized TIP5P force field[29] to simulate water isotherms with a methodology similar to the one used for creating the CO_2 and N_2 isotherms reported in Supplementary Note 3.1. We plot water isotherm data for four representative MOFs in Supplementary Figure 11. The high affinity of water is reflected in the very low partial pressure at which the materials saturate with water, at about 10^{-10} to 10^{-15} bar.

In order to assess the impact of water adsorption on the performance rank ordering, out of the 982 MOFs under consideration, we have removed those structures with higher water affinity than that of CALF-20, i.e. $\Delta G_{\mathrm{H_2O}} < -8.1 kJ/mol$). The 381 remaining MOFs are referred to as "water-tolerant". As a key result shown in Figure 6, 4 out of the 381 remaining MOFs fall within the economically viable region. We provide their relative rank order, as well as their Widom-based free energies of adsorption in Supplementary Table 12. Although these MOFs rank lower compared to the high selectivity MOFs in Supplementary Table 10, they still show CO_2, N_2 and H_2O affinities comparable to those of CALF-20. In Supplementary

Supplementary Table 11. Free energy of adsorption at 30 °C for CO_2, N_2 and H_2O of the top 20 MOFs reported in this work, and CALF-20 (for comparison). All values calculated using the Widom particle insertion method.

MOF name	ΔG_{CO_2}, kJ/mol	ΔG_{N_2}, kJ/mol	ΔG_{H_2O}, kJ/mol
BOHGOU	-14.5	10.3	-65.2
MOCHIW	-14.6	3.9	-86.1
NATXIR	-13.8	7.2	-74.4
CDLGLU11	-13.9	13.3	-37.2
VASFED	-16.2	3.9	-90.1
CAKXET	-15.4	5.0	-97.7
AZAFIS	-18.6	0.9	-60.7
MOCJAQ	-12.7	11.6	-76.3
ESUMOU	-14.1	11.2	-52.0
CDLGLU01	-17.4	12.6	-39.1
MUXHIW01	-16.2	12.8	-66.9
MOCHES	-22.1	5.9	-99.4
IKETAU	-19.4	2.2	-89.2
RATVEP	-11.2	10.8	-63.1
MOCHIW	-23.1	5.8	-106.0
SUKREX	-16.5	2.6	-79.1
IPIJUM	-15.1	3.9	-67.3
NARMUR	-15.7	6.5	-106.5
MOCJEU	-11.6	12.5	-74.2
VIDXOX	-12.5	4.3	-66.9
CALF-20	-9.3	2.2	-8.1

Figure 17, we provide the structures and CO_2, N_2 and H_2O isotherms for these 4 economically viable, water-tolerant MOFs. Interestingly, 2 out of 4 have one-dimensional square channels similar to CALF-20.

Supplementary Table 12. Rank order and free energy of adsorption at 30 °C for CO_2, N_2 and H_2O of the top 4 water-tolerant MOFs (i.e., with $\Delta G_{H_2O} > -8.1$ kJ/mol), and CALF-20 (for comparison). All ΔG values calculated using the Widom particle insertion method. We marked EXOFIH to represent that it was flagged as "bad" by MOSAEC[3].

MOF	K_{eff} = const.	R_μ =1 µm	R_μ =10 µm	R_μ =100 µm	ΔG_{CO_2}, kJ/mol	ΔG_{N_2}, kJ/mol	ΔG_{H_2O}, kJ/mol
EXOFIH*	173	147	145	118	-10.5	-0.4	-8.0
FAHBIC	177	150	148	150	-7.8	5.9	-5.1
MIMVEJ	246	208	206	187	-10.3	-1.3	1.6
FIPXAF	260	222	220	200	-9.0	0.8	-5.6
CALF-20					-9.3	2.2	-8.1

Supplementary Note 4.4 Impact of charge assignment scheme on the rank ordering of water-tolerant, economically-viable MOFs

For quantifying the impact of the charge assignment scheme in the process-level rank ordering of MOFs, we re-ran end-to-end simulations for all four water-tolerant, economically-viable materials in Figure 6. We used PACMOF[48], a machine-learning based DDEC06 charge prediction model, to prepare CIF input files for our end-to-end workflow.

We observe that despite small performance changes, all materials remained economically-viable and maintained their relative rank. We re-calculated the free energy-based water tolerance, ΔG_{H_2O}, and confirmed that two of the materials remained water-tolerant, with MIMVEJ displaying a water affinity 6x lower than the industry benchmark CALF-20.

The lower sensitivity to the choice of charge assignment scheme is in line with findings published previously by some of the authors[49].

Supplementary Table 13. Comparison of CO_2 capture performance and H_2O tolerance metrics when using EQeq and PACMOF partial atomic charges. **Pr**oductivity (higher is preferable), **En**ergy **Re**quirement (lower is preferable) and free energy of H_2O adsorption (higher is preferable) for the four economically-viable, water-tolerant MOFs.

	EQeq			PACMOF		
	**Pr., kg/(day.L)**	**En. Re., MJ/kg**	**ΔG_{H_2O}, kJ/mol**	**Pr., kg/(day.L)**	**En. Re., MJ/kg**	**ΔG_{H_2O}, kJ/mol**
EXOFIH*	1.5	1.6	-8.0	1.5	1.4	-6.4
FAHBIC	1.5	2.9	-5.1	1.4	2.9	-20.8
FIPXAF	1.3	5.0	-5.6	1.1	4.8	-11.7
MIMVEJ	1.3	3.5	1.6	1.2	3.5	-1.0
CALF-20			-8.1			-5.6

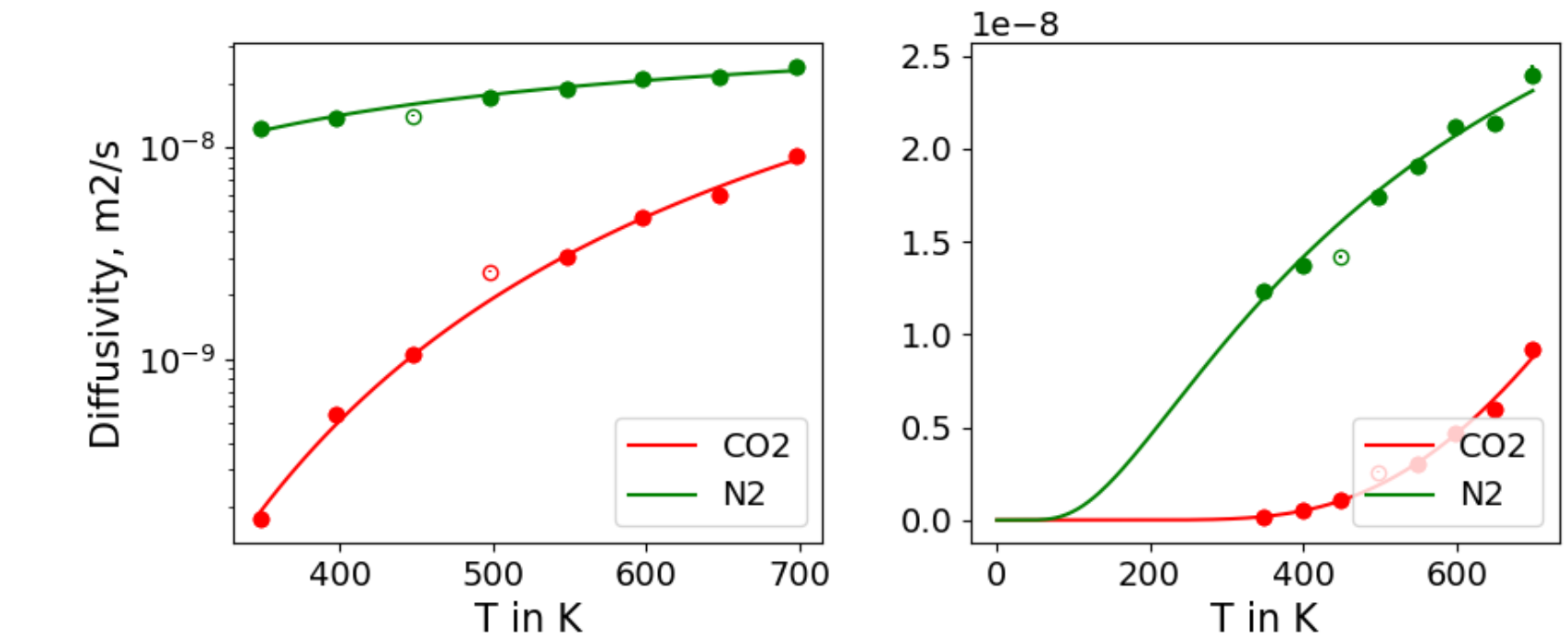

(a)

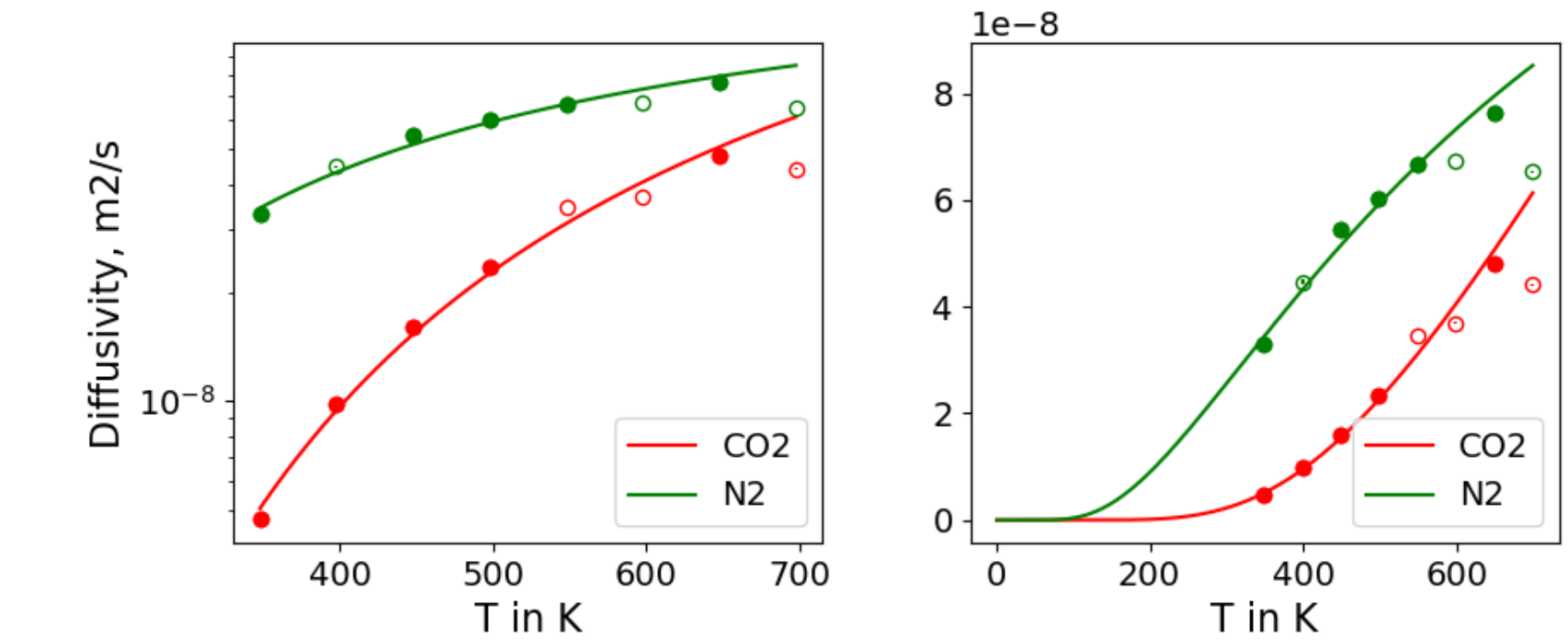

(b)

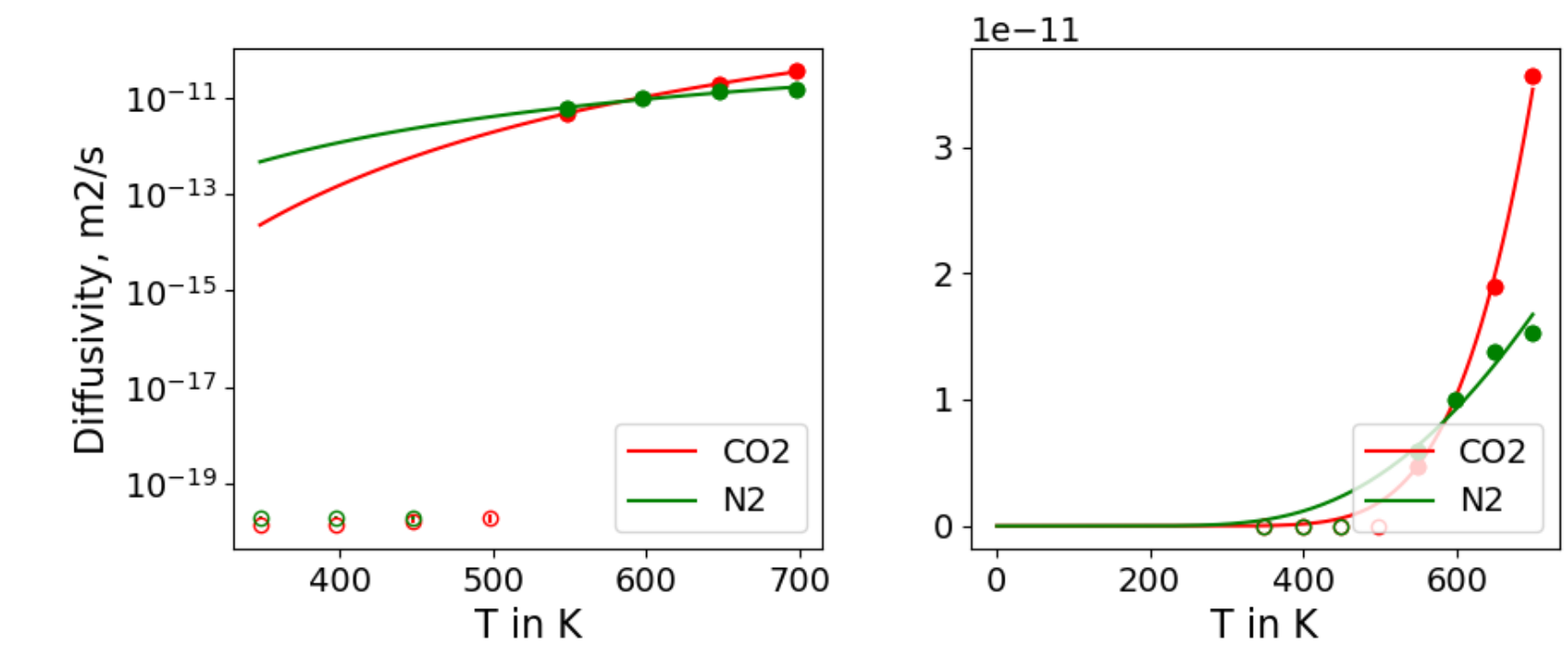

(c)

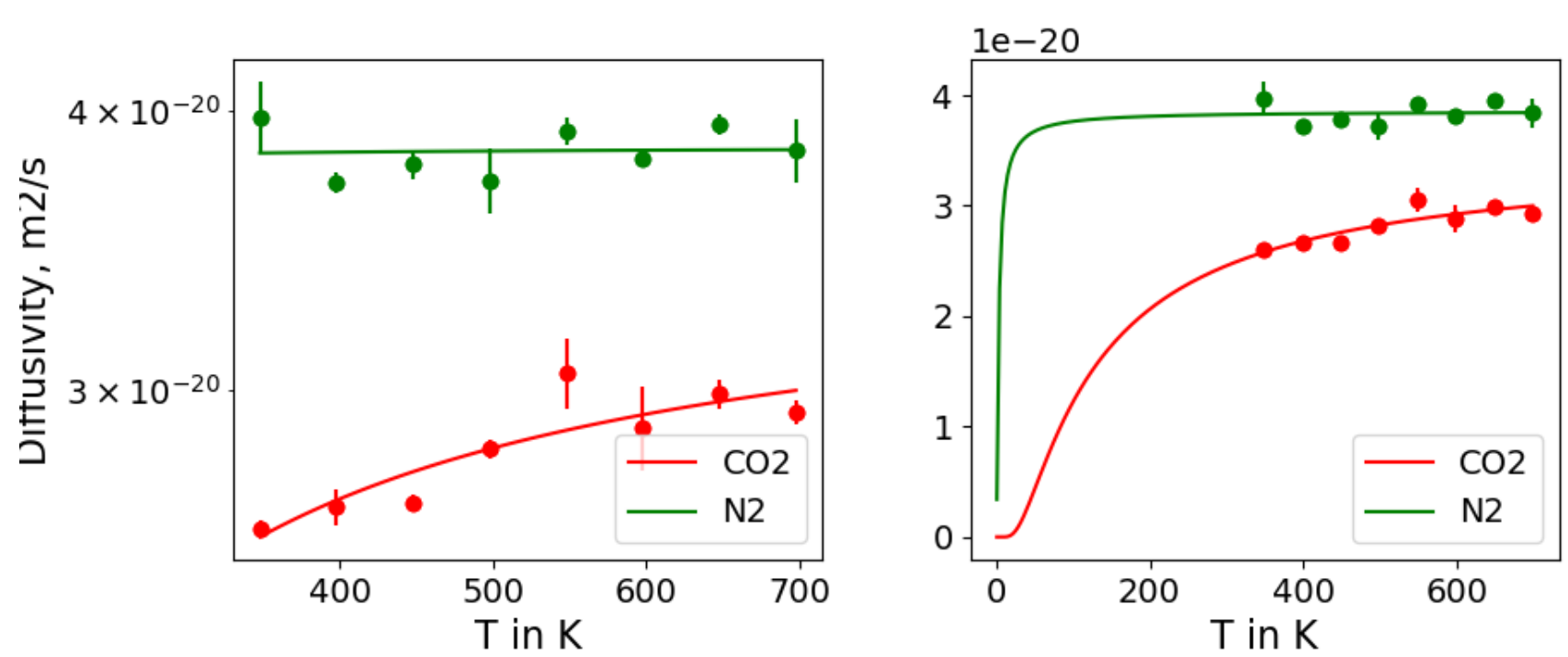

(d)

Supplementary Figure 12. Diffusion Coefficients CO_2, N_2 diffusivity data (filled and empty circles) and fitted models (lines) using data represented by filled circle for MOFs (a) VASFON (b) BETGUE, (c) MOCHIW, (d) EVOMOR in both normal and semi-log scale.

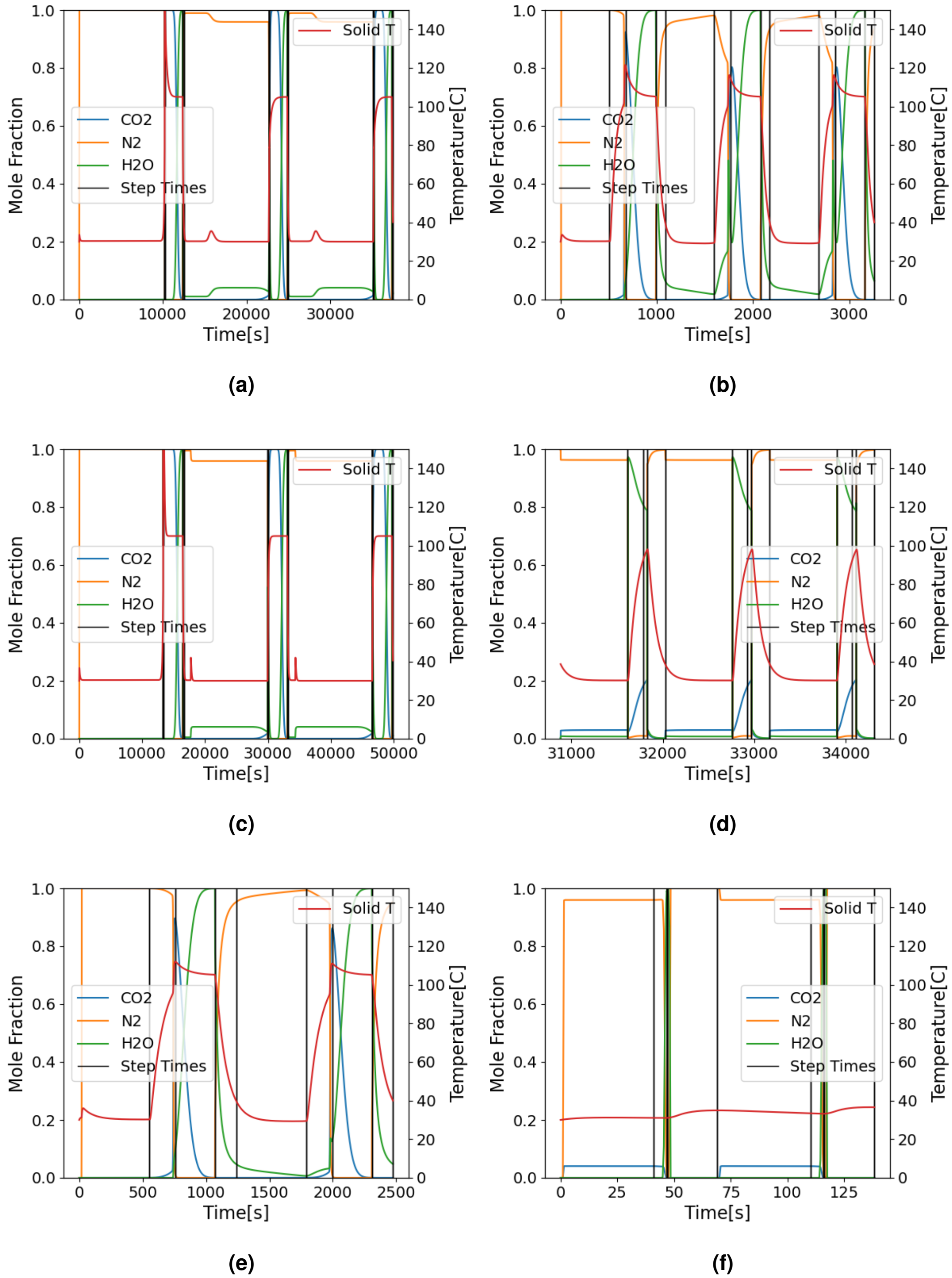

Supplementary Figure 13. **Process Model TSA Model Output** CO_2, N_2 and H_2O mole fractions and adsorbent temperature at the exit of the column plotted as function of time for (a) VASFON with constant diffusivity (b) BETGUE with constant diffusivity, (c) MOCHIW with constant diffusivity, (d) MOCHIW with variable diffusivity for 100 μm, (e) EVOMOR with constant diffusivity, (f) EVOMOR with variable diffusivity for 100 μm.

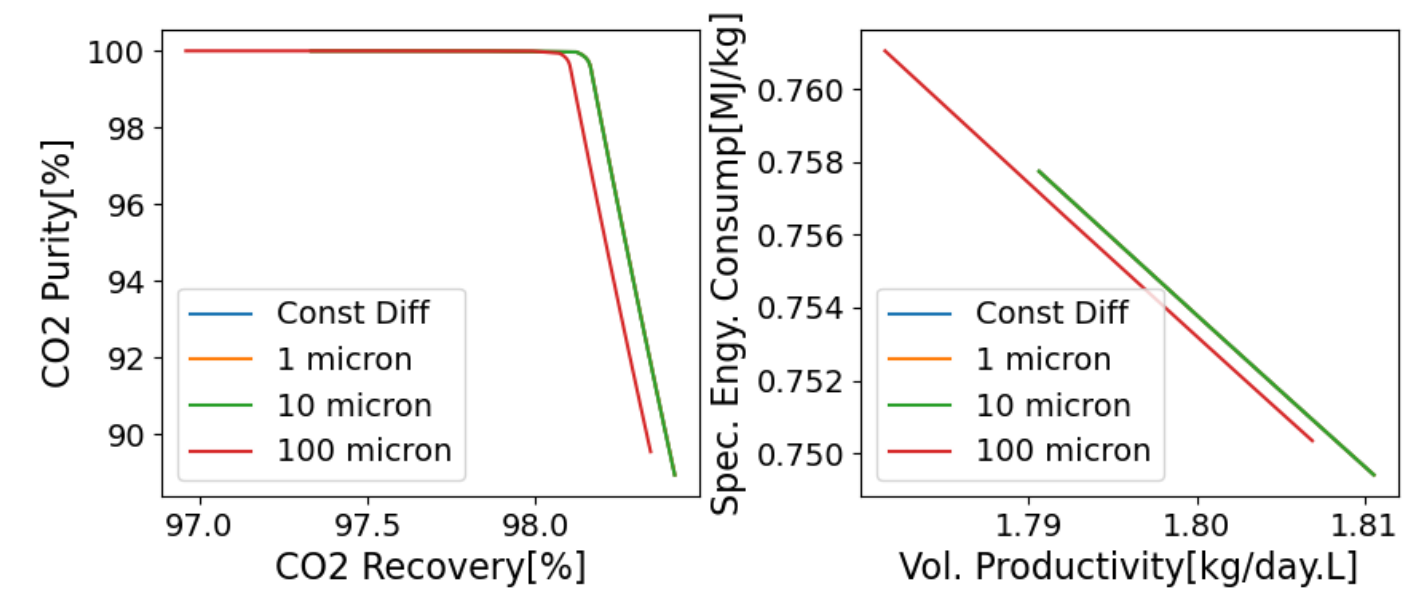

(a)

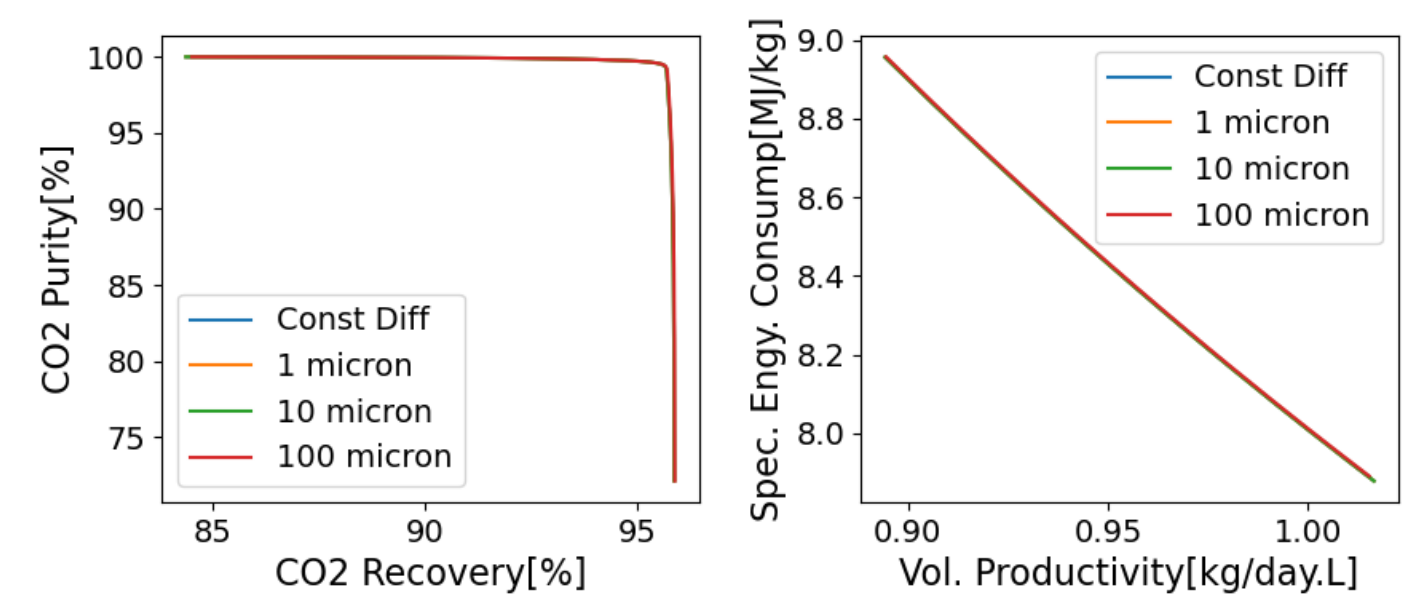

(b)

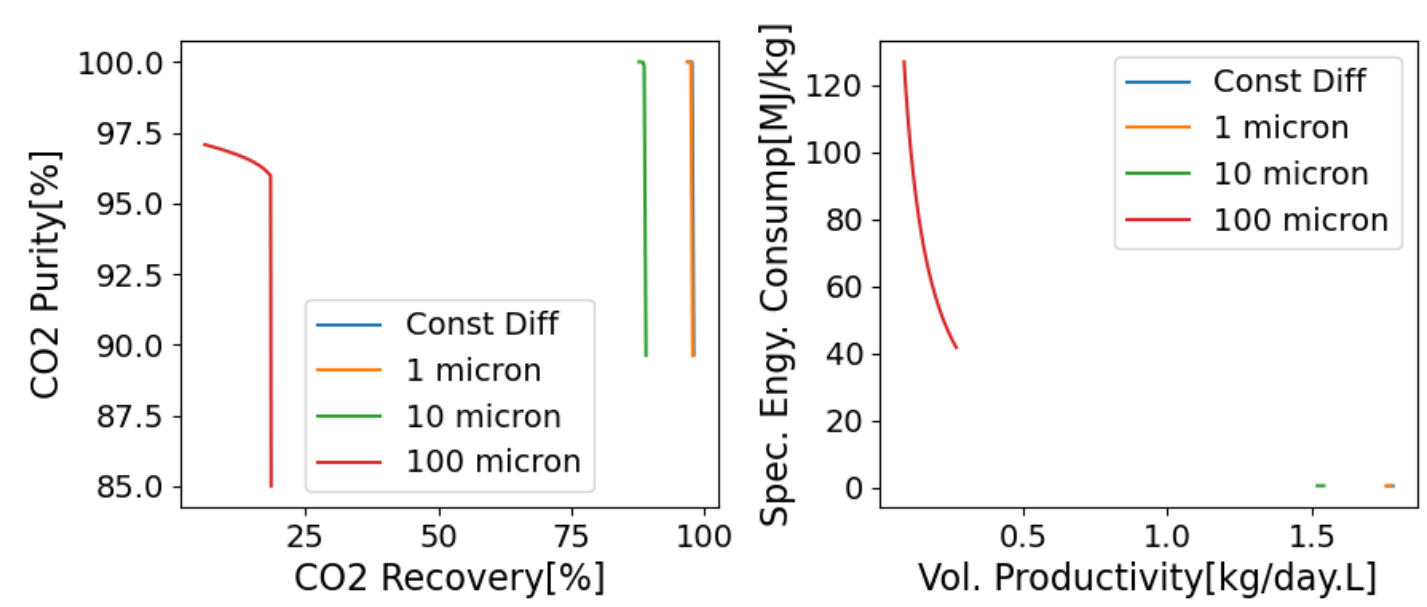

(c)

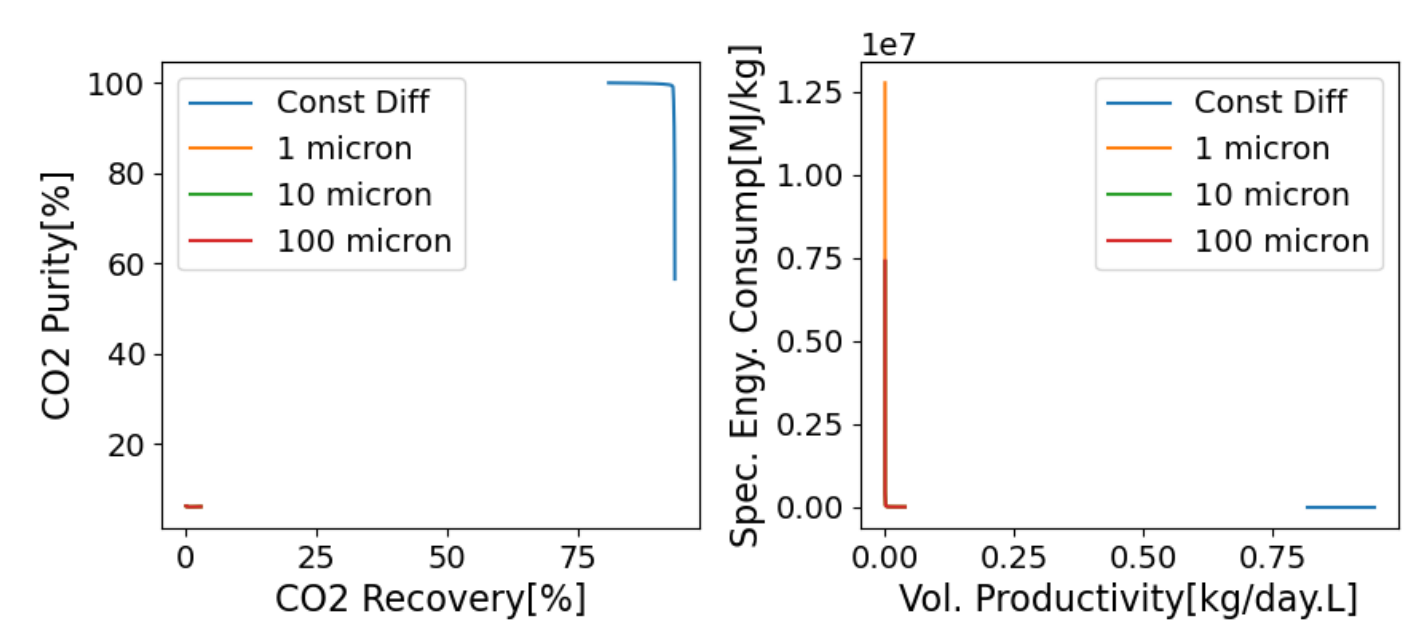

(d)

Supplementary Figure 14. Pareto Plots for Process Based Figures of Merits Purity [%] vs. Recovery [%] on left and Specific Energy [MJ/kg of CO_2] vs. volumetric productivity [kg of CO_2/(day.L) of sorbent] on right, for MOFs (a) VASFON (b) BETGUE, (c) MOCHIW, (d) EVOMOR.

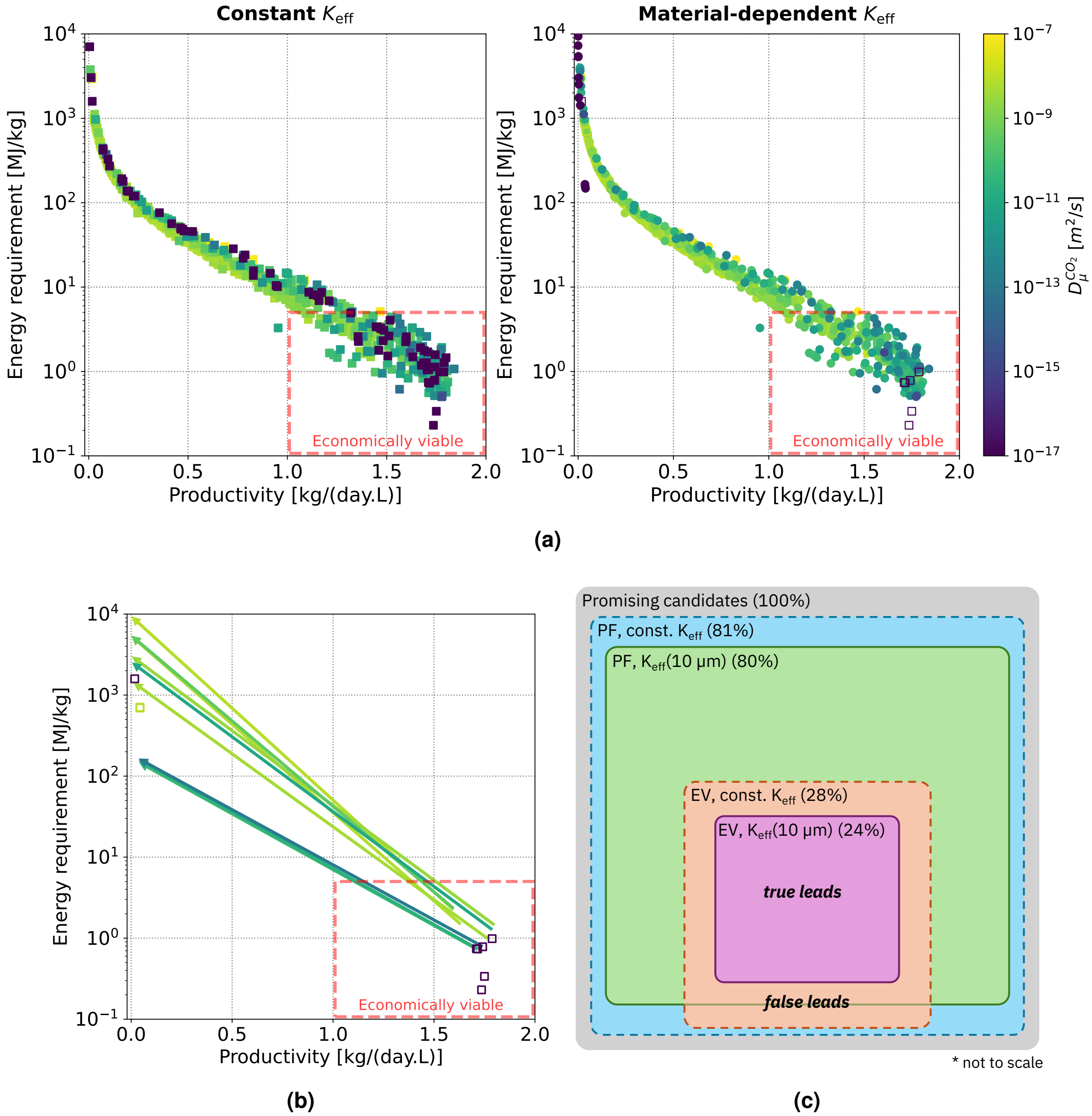

Supplementary Figure 15. **Effect of molecular diffusion on energy requirement and productivity.** (a) Distribution of energy requirement *vs.* productivity for a crystallite diameter $R_{\mu} = 1$ μm, assuming a constant K_{eff} (left) and a material-dependent K_{eff} (right) that accounts for microscopic diffusivity. The respective CO_2 diffusion coefficients are indicated by color. Solid squares represent "process-feasible" (PF) materials, i.e., those whose performance achieved 95% purity and 90% recovery, while open squares mark the positions of excluded materials whose performance failed to meet those targets. The dashed rectangle delineates the "economically viable" (EV) region with productivity higher than $1\,\frac{\text{kg}}{\text{day.L}}$ and energy requirement lower than 5 MJ/kg of CO_2. (b) Effect of microscopic diffusivity in K_{eff} on process performance for crystallite size $R_{\mu} = 1$ μm. Arrows indicate changes undergone by structures for which the inclusion of molecular diffusivity has led to significant process performance drop, removing them from the EV region. (c) Percentages of PF and EV materials with respect to the starting set of "promising candidates" suggested by the active-learning model, assuming constant (dashed lines) or material-specific (solid lines) K_{eff}. The percentage of PF materials dropped from 81% (798 out of 982) to 80% (788 out of 982) upon consideration of microscopic diffusivity. The prevalence of EV materials went from 28% (277 out of 982) to 24% (237 out of 982) of the starting set, or from 35% (277 out of 798) to 30% (237 out of 788) of their respective PF set. This corresponds to a 14% reduction (from 277 to 237) in the original EV population, representing the "false leads".

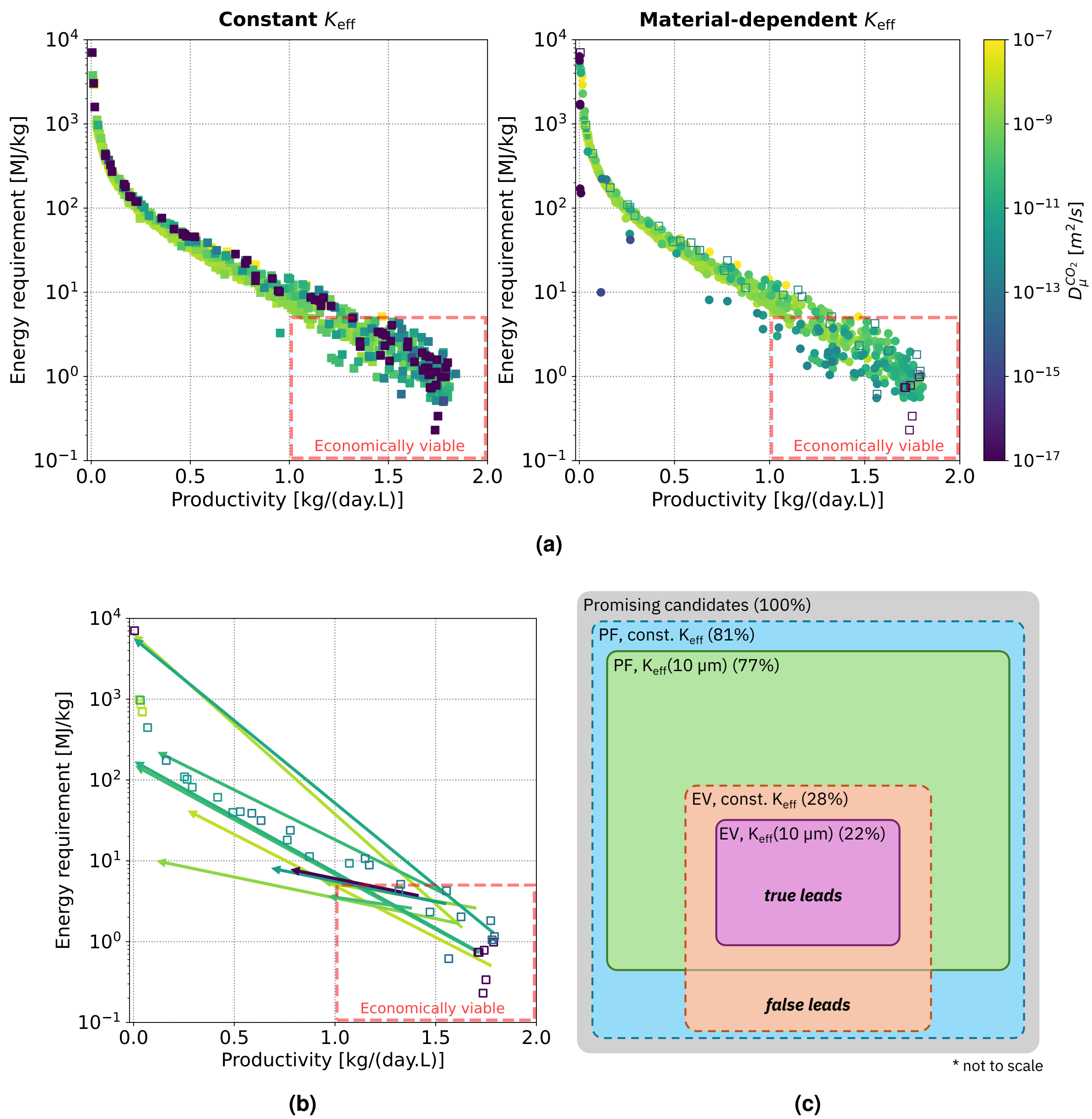

Supplementary Figure 16. Effect of molecular diffusion on energy requirement and productivity. (a) Distribution of energy requirement *vs.* productivity for a crystallite diameter $R_\mu = 100$ μm, assuming a constant K_{eff} (left) and a material-dependent K_{eff} (right) that accounts for microscopic diffusivity. The respective CO_2 diffusion coefficients are indicated by color. Solid squares represent "process-feasible" (PF) materials, i.e., those whose performance achieved 95% purity and 90% recovery, while open squares mark the positions of excluded materials whose performance failed to meet those targets. The dashed rectangle delineates the "economically viable" (EV) region with productivity higher than $1\,\frac{\text{kg}}{\text{day.L}}$ and energy requirement lower than 5 MJ/kg of CO_2. (b) Effect of microscopic diffusivity in K_{eff} on process performance for crystallite size $R_\mu = 100$ μm. Arrows indicate changes undergone by structures for which the inclusion of molecular diffusivity has led to significant process performance drop, removing them from the EV region. (c) Percentages of PF and EV materials with respect to the starting set of "promising candidates" suggested by the active-learning model, assuming constant (dashed lines) or material-specific (solid lines) K_{eff}. The percentage of PF materials dropped from 81% (798 out of 982) to 77% (755 out of 982) upon consideration of microscopic diffusivity. The prevalence of EV materials went from 28% (277 out of 982) to 22% (221 out of 982) of the starting set, or from 35% (277 out of 798) to 29% (221 out of 755) of their respective PF set. This corresponds to a 20% reduction (from 277 to 221) in the original EV population, representing the "false leads".

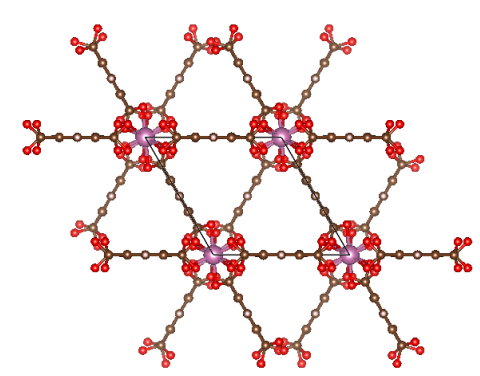

(a) EXOFIH*

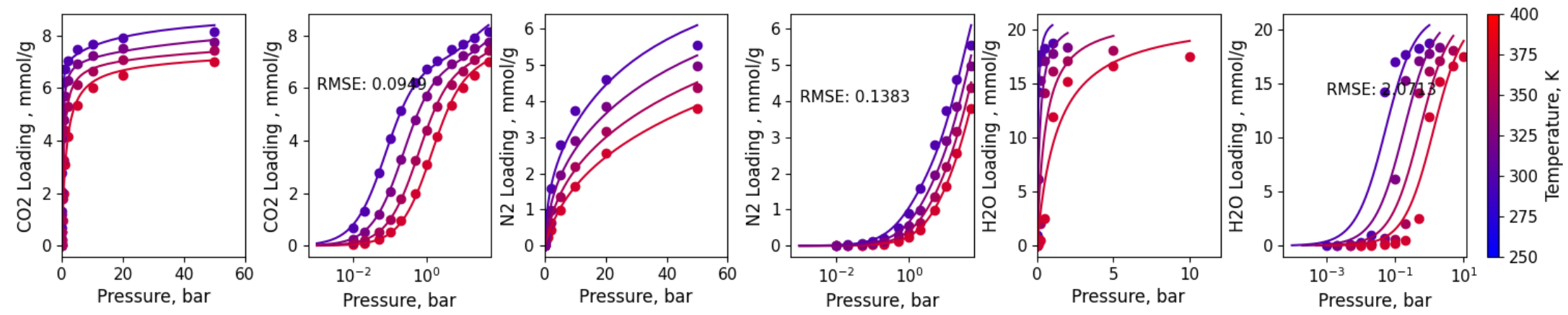

(b) CO_2, N_2 and H_2O adsorption isotherms for EXOFIH*.

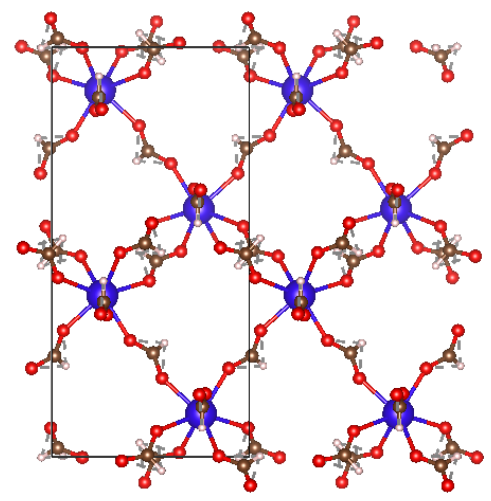

(c) FAHBIC

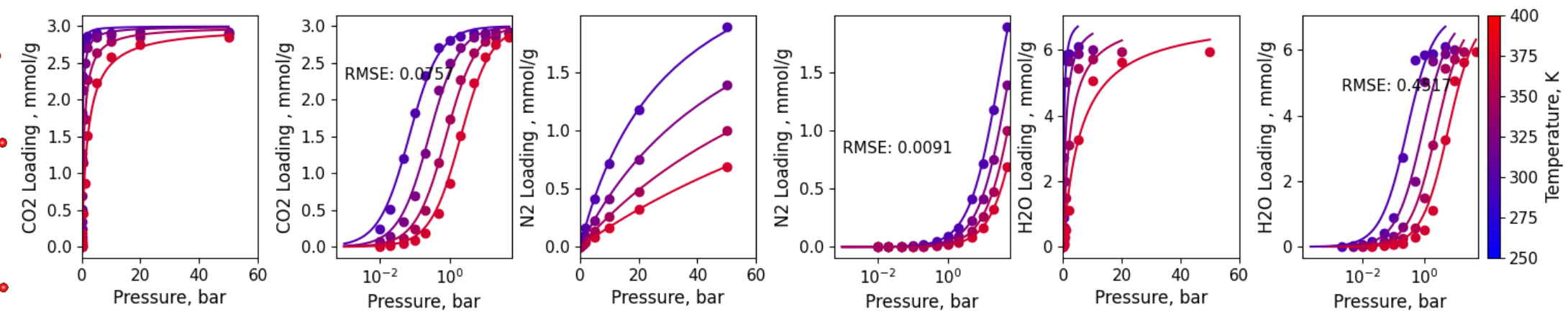

(d) CO_2, N_2 and H_2O adsorption isotherms for FAHBIC.

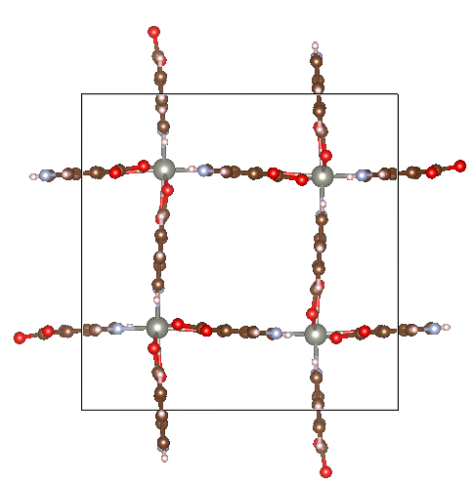

(e) MIMVEJ

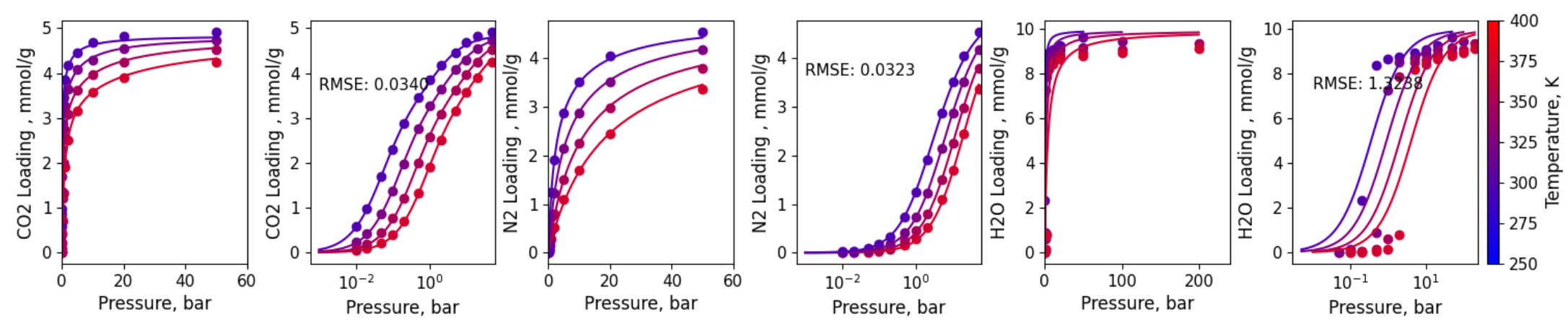

(f) CO_2, N_2 and H_2O adsorption isotherms for MIMVEJ.

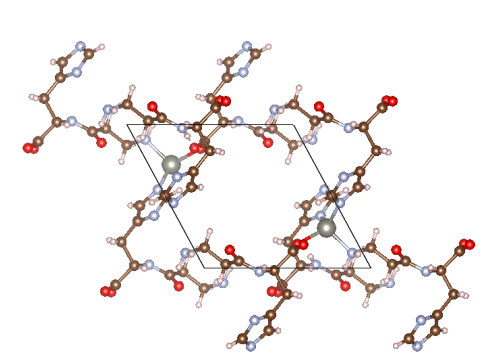

(g) FIPXAF

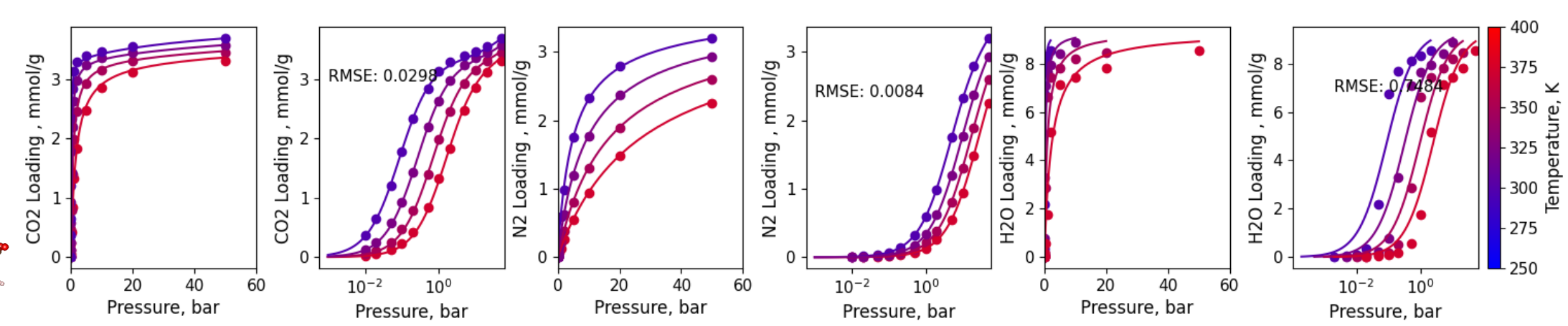

(h) CO_2, N_2 and H_2O adsorption isotherms for FIPXAF.

Supplementary Figure 17. Water-tolerant MOF structures and isotherms (a) EXOFIH*, (c) FAHBIC, (e) MIMVEJ and (g) FIPXAF structures and their associated adsorption isotherms (b), (d), (f) and (h), respectively. CO_2, N_2 and H_2O isotherm data (filled circles) and fitted models (lines). We marked EXOFIH to represent that it was flagged as "bad" by MOSAEC[3].

Supplementary References

1. Jin, X., Jablonka, K. M., Moubarak, E., Li, Y. & Smit, B. Mofchecker: A package for validating and correcting metal-organic framework (mof) structures. *Digit. Discov.* (2025).
2. Rosen, A. S. *et al.* Machine learning the quantum-chemical properties of metal–organic frameworks for accelerated materials discovery. *Matter* **4**, 1578–1597, DOI: https://doi.org/10.1016/j.matt.2021.02.015 (2021).
3. White, A. J., Gibaldi, M., Burner, J., Mayo, R. A. & Woo, T. K. High structural error rates in "computation-ready" mof databases discovered by checking metal oxidation states. *J. Am. Chem. Soc.* **147**, 17579–17583 (2025).
4. Cohn, D., Atlas, L. & Ladner, R. Improving generalization with active learning. *Mach. Learn.* **15**, 201–221 (1994).
5. The MathWorks Inc., Natick, Massachusetts, United States. *MATLAB version: 9.13.0 (R2022b)* (2022).
6. The MathWorks Inc., Natick, Massachusetts, United States. *Statistics and Machine Learning Toolbox: 12.4 (R2022b)* (2022).
7. The MathWorks Inc., Natick, Massachusetts, United States. *Signal Processing Toolbox: 9.1 (R2022b)* (2022).
8. The MathWorks Inc., Natick, Massachusetts, United States. *Optimization Toolbox version: 9.4 (R2022b)* (2022).
9. Tibshirani, R. Regression shrinkage and selection via the lasso. *J. Royal Stat. Soc. Ser. B (Methodological)* **58**, 267–288 (1996).
10. Strobl, C., Boulesteix, A.-L., Kneib, T., Augustin, T. & Zeileis, A. Conditional variable importance for random forests. *BMC Bioinforma.* **9**, 307 (2008).
11. Fryer, D., Strümke, I. & Nguyen, H. Shapley values for feature selection: The good, the bad, and the axioms. *Ieee Access* **9**, 144352–144360 (2021).
12. Lundberg, S. M. & Lee, S.-I. A unified approach to interpreting model predictions. In *Proceedings of the 31st International Conference on Neural Information Processing Systems*, NIPS'17, 4768–4777 (Curran Associates Inc., Red Hook, NY, USA, 2017).
13. Shapley, L. S. A value for n-person games. In Kuhn, H. W. & Tucker, A. W. (eds.) *Contributions to the Theory of Games II*, 307–317 (Princeton University Press, Princeton, 1953).
14. Coley, C. W., Rogers, L., Green, W. H. & Jensen, K. F. Scscore: Synthetic complexity learned from a reaction corpus. *J. Chem. Inf. Model.* **58**, 252–261 (2018).
15. Abbass, H., Sarker, R. & Newton, C. Pde: a pareto-frontier differential evolution approach for multi-objective optimization problems. In *Proceedings of the 2001 Congress on Evolutionary Computation (IEEE Cat. No.01TH8546)*, vol. 2, 971–978 vol. 2, DOI: 10.1109/CEC.2001.934295 (2001).
16. Logan, W., Ankit, A., Choudhary, A. & Wolverton, C. A general-purpose machine learning framework for predicting properties of inorganic materials. *npj Comput. Mater.* **2**, 16028 (2016).
17. Ward, L. *et al.* Matminer: An open source toolkit for materials data mining. *Comput. Mater. Sci.* **152**, 60–69 (2018).
18. Choudhary, K. *et al.* The joint automated repository for various integrated simulations (jarvis) for data-driven materials design. *npj Comput. Mater.* **6**, 173– (2020).
19. Choudhary, K., DeCost, B. & Tavazza, F. Machine learning with force-field-inspired descriptors for materials: Fast screening and mapping energy landscape. *Phys. Rev. Mater.* **2**, 083801–09 (2018).
20. Xie, T. & Grossman, J. C. Crystal graph convolutional neural networks for an accurate and interpretable prediction of material properties. *Phys. Rev. Lett.* **120**, 145301 (2018).
21. Kearnes, S., McCloskey, K., Berndl, M., Pande, V. & Riley, P. Molecular graph convolutions: moving beyond fingerprints. *J. Comput. Mol. Des.* **30**, 595–608 (2016).
22. Sanchez-Lengeling, B. & Aspuru-Guzik, A. Inverse molecular design using machine learning: Generative models for matter engineering. *Science* **361**, 360–365, DOI: 10.1126/science.aat2663 (2018). https://www.science.org/doi/pdf/10.1126/science.aat2663.
23. Townsend, J., Micucci, C. P., Hymel, J. H., Maroulas, V. & Vogiatzis, K. D. Representation of molecular structures with persistent homology for machine learning applications in chemistry. *Nat. Commun.* **11**, 3230 (2020).
24. Jiang, J. *et al.* Topological representations of crystalline compounds for the machine learning prediction of materials properties. *npj Comput. Mater.* **7** (2021).

25. Dubbeldam, D., Torres-Knoop, A. & Walton, K. S. On the inner workings of Monte Carlo codes. *Mol. Simul.* **39**, 1253–1292 (2013).

26. Dubbeldam, D., Calero, S., Ellis, D. E. & Snurr, R. Q. RASPA: molecular simulation software for adsorption and diffusion in flexible nanoporous materials. *Mol. Simul.* **42**, 81–101 (2016).

27. Wilmer, C. E., Kim, K. C. & Snurr, R. Q. An extended charge equilibration method. *The journal physical chemistry letters* **3**, 2506–2511 (2012).

28. Moghadam, P. Z. *et al.* Targeted classification of metal–organic frameworks in the Cambridge structural database (CSD). *Chemical Science* **11**, 8373–8387 (2020).

29. Rick, S. W. A reoptimization of the five-site water potential (tip5p) for use with ewald sums. *The J. chemical physics* **120**, 6085–6093 (2004).

30. White Jr, K. P. An effective truncation heuristic for bias reduction in simulation output. *Simulation* **69**, 323–334 (1997).

31. Oliveira, F. L., Luan, B., Esteves, P. M., Steiner, M. & Neumann Barros Ferreira, R. pyMSER - An Open-Source Library for Automatic Equilibration Detection in Molecular Simulations. *J. Chem. Theory Comput.* **20**, 8559–8568 (2024).

32. Langmuir, I. The adsorption of gases on plane surfaces of glass, mica and platinum. *J. Am. Chem. society* **40**, 1361–1403 (1918).

33. Virtanen, P. *et al.* SciPy 1.0: fundamental algorithms for scientific computing in Python. *Nat. Methods* **17**, 261–272 (2020).

34. Arrhenius, S. Über die Reaktionsgeschwindigkeit bei der Inversion von Rohrzucker durch Säuren. *Zeitschrift für physikalische Chemie* **4**, 226–248 (1889).

35. Van't Hoff, J. H. & Hoff, J. H. *Etudes de dynamique chimique* (F. Muller & Company, 1884).

36. Talu, O. & Myers, A. L. Molecular simulation of adsorption: Gibbs dividing surface and comparison with experiment. *AIChE journal* **47**, 1160–1168 (2001).

37. Dubbeldam, D., Ford, D. C., Ellis, D. E. & Snurr, R. Q. A new perspective on the order-n algorithm for computing correlation functions. *Mol. Simul.* **35**, 1084–1097 (2009).

38. Frenkel, D. & Smit, B. *Understanding molecular simulation: from algorithms to applications* (Elsevier, 2023).

39. Dubbeldam, D., Beerdsen, E., Calero, S. & Smit, B. Dynamically corrected transition state theory calculations of self-diffusion in anisotropic nanoporous materials. *The Journal of Physical Chemistry B* **110**, 3164–3172 (2006).

40. Tovar, T. M. *et al.* Diffusion of co2 in large crystals of cu-btc mof. *J. Am. Chem. Soc.* **138**, 11449–11452 (2016).

41. Jannatabadi, A. A., Bastani, D., Norouzbahari, S. & Ghadimi, A. Co2 and ch4 diffusivities through synthesized zif-8 nanocrystals: An experimental and theoretical investigation. *Microporous Mesoporous Mater.* **324**, 111292 (2021).

42. Forse, A. C. *et al.* Influence of pore size on carbon dioxide diffusion in two isoreticular metal–organic frameworks. *Chem. materials* **32**, 3570–3576 (2020).

43. Kurihara, T., Inukai, M. & Mizuno, M. Slow co2 diffusion governed by steric hindrance of rotatory ligands in small pores of a metal–organic framework. *The J. Phys. Chem. Lett.* **13**, 7023–7028 (2022).

44. Peksa, M., Lang, J. & Stallmach, F. 13c nmr study of diffusion anisotropy of carbon dioxide adsorbed in nanoporous dmof-1. *Microporous Mesoporous Mater.* **205**, 11–15 (2015).

45. Verstreken, M. F. K. *et al.* Mind the Gap: The Role of Mass Transfer in Shaped Nanoporous Adsorbents for Carbon Dioxide Capture. *J. Am. Chem. Soc.* **146**, 23633–23648, DOI: 10.1021/jacs.4c03086 (2024). Publisher: American Chemical Society.

46. Moosavi, S. M. *et al.* A data-science approach to predict the heat capacity of nanoporous materials. *Nat. materials* **21**, 1419–1425 (2022).

47. Lin, J.-B. *et al.* A scalable metal-organic framework as a durable physisorbent for carbon dioxide capture. *Science* **374**, 1464–1469 (2021).

48. Kancharlapalli, S., Gopalan, A., Haranczyk, M. & Snurr, R. Q. Fast and accurate machine learning strategy for calculating partial atomic charges in metal–organic frameworks. *J. Chem. Theory Comput.* **17**, 3052–3064 (2021).

49. Oliveira, F. L. *et al.* Crafted: An exploratory database of simulated adsorption isotherms of metal-organic frameworks. *Sci. Data* **10**, 230 (2023).
