# Supplementary material for: Molecular diffusion enhanced performance evaluation of metal-organic frameworks for CO2 capture
Source: Nat Commun. 2026 Jun 17;17:7654. doi: 10.1038/s41467-026-73904-5 (PMC13434109; doi:10.1038/s41467-026-73904-5)
Supplement: Supplementary file 2 [file 41467_2026_73904_MOESM2_ESM.pdf]

nature portfolio

Peer Review File

Molecular diffusion enhanced performance evaluation of metal-organic frameworks for CO_2 capture

Corresponding Author: Dr Mathias Steiner

This file contains all reviewer reports in order by version, followed by all author rebuttals in order by version.

Version 0:

Reviewer comments:

Reviewer #1

(Remarks to the Author)
The study by Rodrigo Neumann Barros Ferreira et al. addresses an important and often overlooked aspect of metal-organic framework (MOF) performance for carbon capture: the influence of diffusion. Most MOF screening studies neglect diffusion effects, so this work is a valuable contribution to the field. However, despite the topic's relevance, I have significant technical concerns that prevent me from recommending publication in its current form.

Major Concerns:
1. Realism of Diffusion Limitations in MOFs The authors investigate diffusion limitations within the pores of MOF crystals. However, it is well established that such limitations can be mitigated by reducing crystal size. At sufficiently small sizes, macro-diffusion becomes the dominant transport mechanism. While there is likely a lower bound to feasible crystal size reduction, the manuscript does not clearly justify whether the studied regime is realistic. The motivation behind the selected crystal sizes should be explicitly stated, along with evidence that diffusion remains a relevant bottleneck in practical applications.

2. Neglect of Water in Flue Gas The study focuses exclusively on dry flue gases, but in real-world conditions, flue gas typically contains significant amounts of water. The presence of water can drastically alter adsorption behavior and diffusion properties, potentially undermining the conclusions of this study. A discussion of how water affects the system, or at least an acknowledgment of this limitation, is necessary for a more complete assessment.

3. Lack of Essential Methodological Details Several critical aspects of the methodology are either vague or insufficiently detailed, making it difficult to assess the validity of the findings. Examples include:
o he statement, "We have identified those providing the best balance between energy cost, productivity, and recovery," is too general. These metrics should be explicitly quantified to support the conclusions.

o The description of the active-learning model for predicting CO_2 and N_2 adsorption free energy is unclear. Adsorption free energy depends on temperature and partial pressure, and its relevance to the process should be clarified. It appears that the authors may be considering the Henry regime, but if so, this should be explicitly stated. Furthermore, Henry's selectivity is known to be a poor predictor of performance in many cases (see DOI:10.1038/s41586-024-07683-8).

o The discussion of diffusion coefficients lacks critical details. The authors compute the self-diffusion coefficient, whereas the transport diffusion coefficient is the more relevant parameter for process performance. While these values coincide at zero loading, they diverge significantly at saturation (where the self-diffusion coefficient drops to zero while the transport diffusion coefficient approaches infinity). The manuscript should specify the conditions under which the diffusion coefficient is computed and justify its relevance to the studied system.

Conclusion
The topic of this study is undoubtedly important, and considering diffusion in MOF-based carbon capture is a necessary step forward. However, the manuscript raises too many concerns in its current form. Addressing these issues is essential before the work can be considered for publication.

In addition, I do not see how the authors can justify the title. My understanding of the manuscript is that diffusion limitation reduces the performance of materials, while the title suggests an enhancement related to diffusion. The title is intriguing and got my attention, but I was disappointed after reading the manuscript.

(Remarks on code availability)

Reviewer #2

(Remarks to the Author)
The article by Ferreira et al. highlights the importance of molecular diffusion, a factor often overlooked in screening studies. Its key contribution is providing a systematic approach to incorporating diffusion into screening methods. The study is interesting. However, the implementation has significant limitations that reduce its practical relevance:
• The model does not account for the effect of water. While diffusion could be an important factor in material exclusion, water presence in the flue gas stream is even more critical. The authors assume a drying step upstream of the capture unit, which considering the flue gas volumes and scale of the operation, can render a very costly process. Hence, a much more realistic scenario would be to incorporate the water in the flue gas stream and conduct the screening study under such conditions.
• The active learning model targets high CO2/N2 selectivity. Thus, selectivity is used as a primary screening criterion for top-performing materials, despite studies indicating that selectivity alone is a poor predictor of real-world performance. There is a clear lack of integration between the process model and the molecular properties calculations in the ML model feedback loop. Such integration would provide a much more interesting and relevant approach, where molecular diffusion can properly be incorporated in the analysis at the screening stage. In my opinion, the title of the article is overpromising and not a true reflection of what it is done in the study.
• I have several concerns when it comes to the process modelling part as the text is confusing. In pg 9 (“process level simulations” section), it is mentioned that: “The competitive adsorption of CO2, N2 and H2O is modelled as a multi-component mixed Langmuir isotherm derived from the combination of pure component isotherms”. The adsorption step was conducted under dry conditions so not clear to me for which step this competitive adsorption was calculated. Obviously, since they are making use of direct heating with steam in the purge and desorption steps of the process, the water isotherms need then to be known and calculated for such steps. There is no mention at all about water isotherms in the manuscript and they are not presented. This is a major concern and there is a lack of clarity on how they have done the calculations.
• The details for the calculation of the energy cost are not provided in the text and there are misleading statements: for instance, caption in Figure 4: “Effect of molecular diffusion on CO2 capture cost and productivity”. It is the energy cost what it is provided and not the CO2 capture cost.
• I find Figure 4b-d very confusing and suggest replotting for clarity purposes.
• Typographical errors need to be corrected.
While the study takes an interesting first step in emphasizing diffusion, the subsequent analysis requires substantial improvements before it can fully support the claims made in the title.

(Remarks on code availability)

Reviewer #3

(Remarks to the Author)
This manuscript describes molecular simulations and process-scale simulations of carbon dioxide capture performance of MOFs. The work is useful since the CO2 separation performances of large number of materials has been computed. However, in its current form, I do not think that the work can be published in highly presitigous Nature journal because the experimental validation -perhaps the most critical part- is missing. Without the experiömental synthesis and testing of the MOF, I believe thr results do not have the value to be published in Nature. My other comments related to simulation techniques and results are as follows:
1-The abstract starts by saying 'evaluating 10,143 MOFs for post-combustion carbon dioxide capture' but I believe many materials should have been eliminated due to structural problems or pore size. Is this really the final number of materials that gone to simulations?
2- For the charges, EQEQ method was used, did the authors validate the accuracy of this method compared to DDEC charges. Since CO2 was studies, using a highly accurate partial charge assignment emthod must be priority, and since DDEC charges are available for most MOFs, I do not why the authors prefered an approximate method.
3- A recent work by Woos group published in Chemical Science showed that the Core MOF database has a lot of incorrect structures, did the authors check their MOFs and compare with the ones reported as corrected structures in that work?

(Remarks on code availability)

Version 1:

Reviewer comments:

Reviewer #3

(Remarks to the Author)
I now read the rebuttal letter provided by the authors. I still keep my original comment on the chemical problems of the MOFs studied in this work. The authors mentioned that they used mofchecker to eliminate 3,246 CoRE-MOF-2019 structures that have problems. However this is not enough as I explained in my previous round of comments.1- Tom Woo's group developed a tool called mosaec to check the accuracy -chemical correctness- of the MOF structures and the results of moseac tool does not always agree with the mofchecker. Saying in other words, structures labeled as 'correct' by mofchecker can still have problems such as wrong oxidation states as identified by moseac tool. Therefore, I strongly suggest the authors to check their MOFs by both tools- please see the recent JACS article on this. 2- Another development is that the new CoRE MOF 2025 database just came out a few weeks ago-so it is very important for the authors to check with this database, because many MOFs seem to be included in CoREMOF 2019 are now labedled as incorrect-not computation ready-. I believe without prooving the structures' chemical correctness, identfying best performing materials for a target application is highly misleading.

(Remarks on code availability)
I now read the rebuttal letter provided by the authors. I still keep my original comment on the chemical problems of the MOFs studied in this work. The authors mentioned that they used mofchecker to eliminate 3,246 CoRE-MOF-2019 structures that have problems. However this is not enough as I explained in my previous round of comments.1- Tom Woo's group developed a tool called mosaec to check the accuracy -chemical correctness- of the MOF structures and the results of moseac tool does not always agree with the mofchecker. Saying in other words, structures labeled as 'correct' by mofchecker can still have problems such as wrong oxidation states as identified by moseac tool. Therefore, I strongly suggest the authors to check their MOFs by both tools- please see the recent JACS article on this. 2- Another development is that the new CoRE MOF 2025 database just came out a few weeks ago-so it is very important for the authors to check with this database, because many MOFs seem to be included in CoREMOF 2019 are now labedled as incorrect-not computation ready-. I believe without prooving the structures' chemical correctness, identfying best performing materials for a target application is highly misleading.

Reviewer #4

(Remarks to the Author)
The manuscript reports the inclusion of molecular diffusion for process-scale simulations of CO2 capture in MOFs. It can be seen that a great deal of works have been done. Screening stable new MOFs with excellent carbon capture performance at process-scale simulation is important. However, there are some major concerns for the publication of manuscript.
1.The validation of methodology for calculating the diffusion coefficients is not sufficient. In section S3.2.2, to validate the methodology, the authors lists the comparison of experimental and simulated diffusion coefficients of 6 MOFs in Table S8. However, the sample numbers are quite small comparing with the large numbers of estimated MOFs. How the situations for other MOF materials? Moreover, there is huge difference between the experimental and simulated results for CID-Me.
2.Filling columns in Industrial application need to constantly undergo changes in temperature and pressure gradients, so adsorbents must remain stable during multiple repetitive cycle. Stability testing, however, is a very time-consuming process. Did authors consider this aspect?
3.For machine-learning process, the training data was extracted by fitting a model functions to the GCMC-simulated adsorption isotherms instead of experimental data, Why?The most challenge for model training in machine learning is the accuracy of data. In my opinion, it will be more convincing to use experimental data for training.
4.As authors states in the manuscript "we suggest to include micro-pore gas diffusivity, provided for each MOF structure through GCMC simulations, into the process simulation of CO2 capture performance, it should be MD simulations instead of GCMC simulations.

(Remarks on code availability)

Reviewer #5

(Remarks to the Author)
The manuscript titled "Molecular Diffusion-Enhanced Performance Evaluation of Metal–Organic Frameworks for CO_2 Capture" introduces a workflow that employs molecular-level simulations and active learning techniques to evaluate the Core-MOF database for CO2 capture. It clearly demonstrates the importance of material-dependent properties, such as the gas diffusion coefficient, when ranking the performance of MOFs.

The work is thoroughly carried out and accompanied by sufficient details to substantiate the authors' claims and ensure reproducibility. However, I have a few points that might need clarification:

1. How was the energy requirement plotted in Figures 4-6 obtained?

2. The energy requirement depends on the heat capacity of the material, which in general varies with temperature. Did the energy requirement presented account for the structure- and temperature-dependent heat capacity? If not, a comment on the expected change in the distribution of the energy requirement may be beneficial. Authors may refer to https://doi.org/10.1038/s41563-022-01374-3 and https://doi.org/10.1103/PRXEnergy.2.023005 for materials dependent heat capacity and how the affect heat/energy requirements.

(Remarks on code availability)
The GitHub repository includes a README file that provides a step-by-step guide on how to use the code.

Version 2:

Reviewer comments:

Reviewer #3

(Remarks to the Author)
After my comments about the chemical correctness of the structures, the authors in the second round identified 224 materials as incorrect. They use the word 'bad' following the Woo groups description, but bad simply means these materials not MOFs or at least they are incorrect MOF structures. I think this is not a small change, and I am surprised to read that authors think that their general conclusion does not change after the removal of 224 MOFs among thousands. The figures clearly show the changes. I keep my original decision, the current work is not publishable, the study must be performed using the correct MOF database, removing wrong structures is not enough, please start with correct and corrected (not just remove) structures.

(Remarks on code availability)

Reviewer #6

(Remarks to the Author)
In my assessment, the authors have made a sincere and substantial effort to address the reviewer’s concerns, and the central issue raised by Reviewer #3 has largely been resolved. While MOSAEC is a useful error checking tool in MOFs, it should not be treated as a definitive or exhaustive measure of chemical correctness. I do not think it is appropriate to conclude that all structures flagged by MOSAEC must necessarily be removed. Importantly, the authors have explicitly acknowledged this limitation and have demonstrated it by visualizing the structures flagged by MOSAEC, showing that these structures are in fact computation-ready. For additional check, authors are encourneged to use recently developed MOFClassifier which uses a PU-CGCNN method to classify CR and NCR MOFs for further validating their top MOFs.

https://pubs.acs.org/doi/full/10.1021/jacs.5c10126

Some additional remarks
1. partial atomic charges estimated from EQeq may not be the same as the partial atomic charges from DDEC06 and other DFT-derived charges, and it will impact CO2 isotherm which will impact the ranking of the materials. Authors are encouraged to provide the impact of using high-fidelity DDEC06 charges on the ranking of their "economically viable" materials. Some fast ML models have been developed to estimate the DFT-quality partial atomic charges, such as PACMOF and PACMAN.

2. There has been some work on CO2/N2 separation with integrated molecular simulation + process modeling on recent MOF DBs which may be included for references.

https://www.sciencedirect.com/science/article/abs/pii/S2590238525001833

(Remarks on code availability)

Reviewer #7

(Remarks to the Author)
I read both the manuscript and the rebuttal letter from the authors. And while the validation of the computed diffusion coefficients is necessarily limited (only 6 MOF data) and some discrepancies with experiment remain substantial, I do not think this undermines the manuscript’s central contribution. In my view, the work should be interpreted primarily as a high-throughput, internally consistent screening study showing that inclusion of structure-specific molecular diffusion can alter process-level conclusions. Given the scale of the screening and the lack of practical higher-accuracy alternatives for transport calculations across hundreds of MOFs, the chosen methodology appears reasonable for this purpose, even though the exact ranking of individual materials may vary with the underlying force field, charge model, and diffusivity formalism.

(Remarks on code availability)

Open Access This Peer Review File is licensed under a Creative Commons Attribution 4.0 International License, which permits use, sharing, adaptation, distribution and reproduction in any medium or format, as long as you give appropriate credit to the original author(s) and the source, provide a link to the Creative Commons license, and indicate if changes were made.
In cases where reviewers are anonymous, credit should be given to 'Anonymous Referee' and the source.
The images or other third party material in this Peer Review File are included in the article's Creative Commons license, unless indicated otherwise in a credit line to the material. If material is not included in the article's Creative Commons license and your intended use is not permitted by statutory regulation or exceeds the permitted use, you will need to obtain permission directly from the copyright holder.
To view a copy of this license, visit https://creativecommons.org/licenses/by/4.0/

Reviewer #1

The study by Rodrigo Neumann Barros Ferreira et al. addresses an important and often overlooked aspect of metal-organic framework (MOF) performance for carbon capture: the influence of diffusion. Most MOF screening studies neglect diffusion effects, so this work is a valuable contribution to the field. However, despite the topic's relevance, I have significant technical concerns that prevent me from recommending publication in its current form.

The authors thank the reviewer for recognizing the importance of the topic, and the value that this work can bring to the field. We are grateful for the suggestion and comments, for they have spurred the team to further improve the presentation of the work. Below, we provide a point-by-point response to the concerns raised by the reviewer and present a revised version for your consideration.

Major Concerns

1. **Realism of Diffusion Limitations in MOFs:** The authors investigate diffusion limitations within the pores of MOF crystals. However, it is well established that such limitations can be mitigated by reducing crystal size. At sufficiently small sizes, macro-diffusion becomes the dominant transport mechanism. While there is likely a lower bound to feasible crystal size reduction, the manuscript does not clearly justify whether the studied regime is realistic. The motivation behind the selected crystal sizes should be explicitly stated, along with evidence that diffusion remains a relevant bottleneck in practical applications.

The reviewer is right in stating that decreasing crystal size allows one to overcome the microscopic diffusion limitation – which is exactly what we show in Figure 5. However, reducing the crystal size to sub-micron level may not always be an accessible option due to specific synthesis recipe of each MOF. In addition, a process application might require a minimum crystal size for practical reasons.

When working with commercial materials, we are restricted to off-the-shelf options. In this case, synthesis customization is not an option. To demonstrate the validity of our choice of representative diameters, we purchased 5 representative materials from chemical providers and performed bright field microscopy to determine their crystal size distributions.

1. Basolite F300 [link] ($C_9H_3FeO_6$), a.k.a. Fe-BTC, Iron 1,3,5-benzenetricarboxylate. Measured with a Zeiss EC Epiplan-Neofluar Dic 100x / 0.9 objective. 100x / 0.9 objective.), a.k.a. Fe-BTC, Iron 1,3,5-benzenetricarboxylate.
2. AC MOF-74 [link] ($C_8H_4O_8Mg_2$), a.k.a. Mg-MOF-74; Mg-DOBDC; CPO-27(Mg); Mg_2(dhtp). Measured with a Zeiss EC Epiplan-Neofluar Dic 100x / 0.9 objective. 100x / 0.9 objective.
3. Basolite Z1200 [link] ($C_8H_{10}N_4Zn$), a.k.a. 2-Methylimidazole zinc salt, ZIF-8. Measured with a Zeiss EC Epiplan-Neofluar Dic 100x / 0.9 objective., ZIF-8.

4. Basolite C300 [link] ($C_{18}H_6Cu_3O_{12}$), a.k.a. Copper benzene-1,3,5-tricarboxylate, Cu-BTC MOF, HKUST-1. Measured with a Zeiss LD Plan-Neofluar 63x / 0.75 objective.
5. Basolite Z377 [link] ($C_{54}H_{30}O_{13}Zn_4$), a.k.a. MOF 177. Measured with a Zeiss EC Epiplan 10x / 0.25 objective.

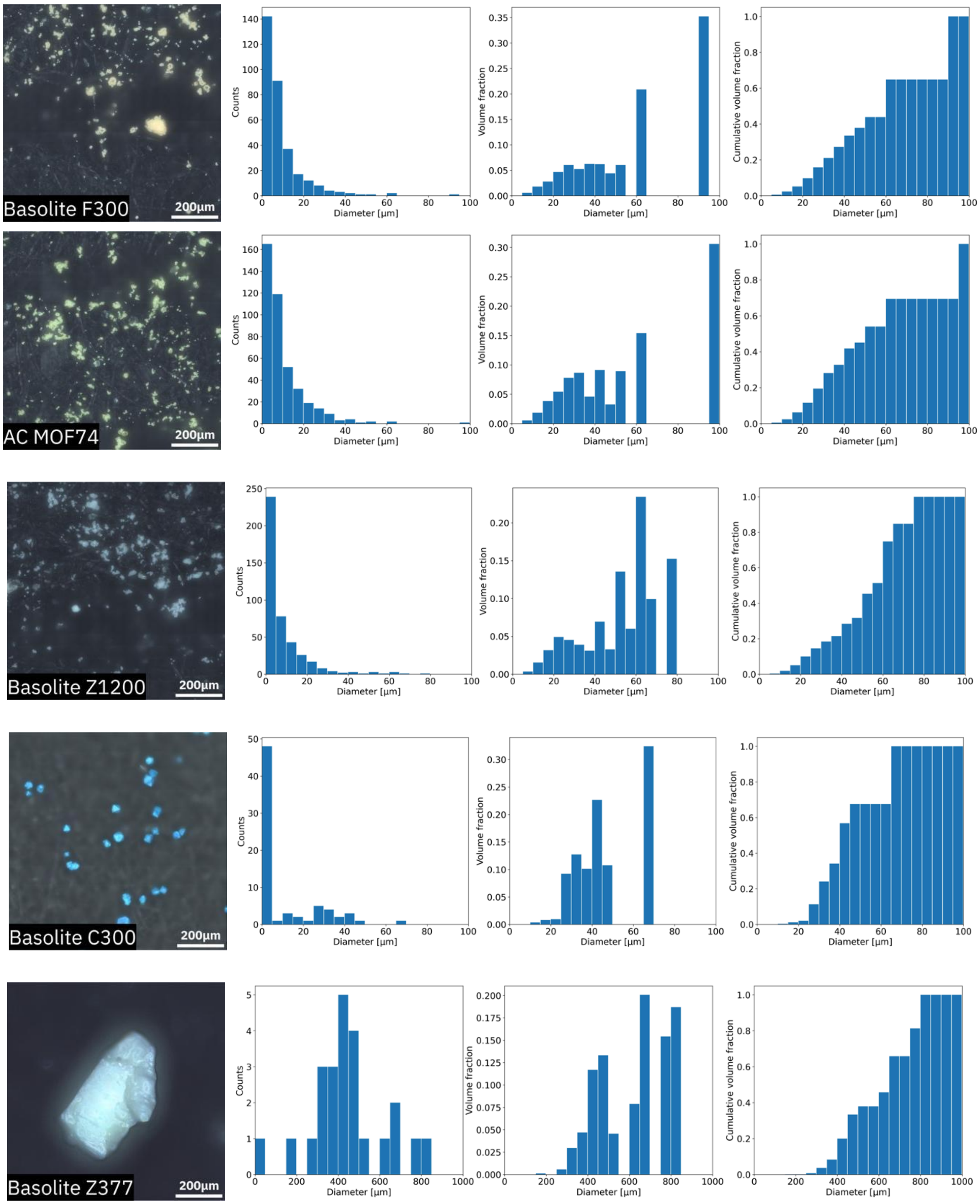

Figure L1: Bright field microscopy images and crystal size distributions for material: Basolite F300, AC MOF-74, Basolite Z1200, Basolite C300 and Basolite Z377, from top to bottom. The graphics are provided by private communication with Matheus Esteves Ferreira (IBM), and are reproduced with permission from the author.

The measurements were made on a WITEC Alpha RAS microscope and are shown on the leftmost column of Figure L1. Although we took images of an area as large as 20 µm × 20 µm, we always show a 1 µm × 1 µm area to allow for a straightforward visual

comparison. ImageJ/Fiji was used to estimate the crystallite diameters. On the second column of Figure L2, we plot the histogram of particle diameters. As expected, the particle counts are clustered at the lower end of the histogram, with the notable exception of Basolite Z377 which show crystal diameters of hundreds of micrometers. Despite appearing with higher frequency, micrometer-sized particles contribute relatively little to the volumetric fraction of the material. Sub-micrometric particles, if at all present, would contribute even less to the volume. As shown in the third and fourth columns of Figure 2, the volumetric fraction is dominated by crystals with tens (or hundreds) of micrometers.

Turning to the scientific literature for further evidence, we find that even for the well-known and widely studied Mg-MOF-74 [https://doi.org/10.1021/acsami.9b13965, https://doi.org/10.1039/D4RA03943B], special synthesis methods (e.g. chemical etching) are needed for reducing the crystal size to sub-micron level. Most lab-scale synthesis recipes are not directly scalable for industrials applications that require tons of active material. Hence, at screening level, it becomes imperative to know how a material's rank order is affected by crystal size. The MOFs whose performance is more sensitive to crystal size pose a higher scale-up risk, given the strict requirement of sub-micron crystal sizes.

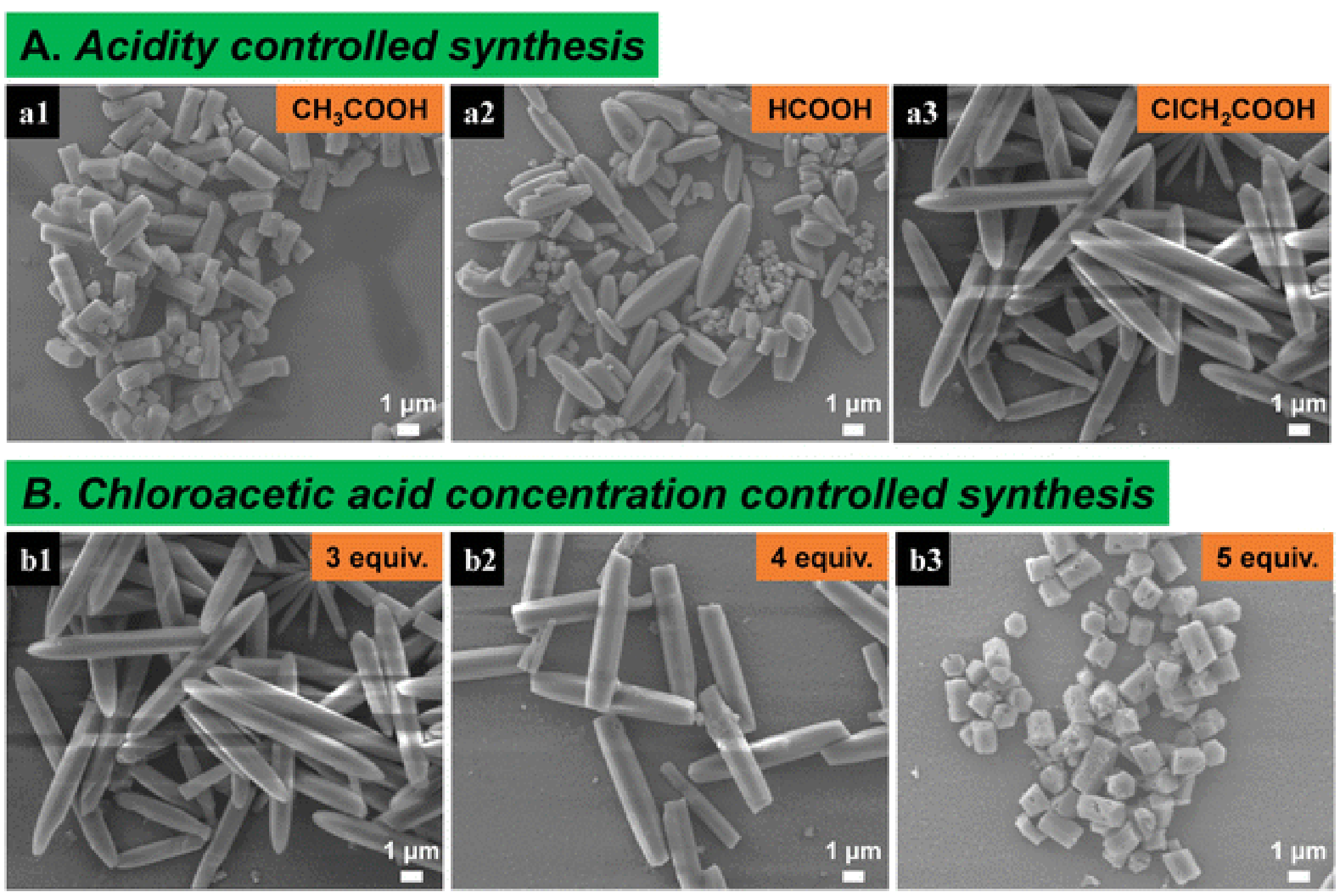

Figure L2: (A) SEM images of Mg-MOF-74 synthesized with capping agents of various acidity. (B) SEM images of Mg-MOF-74 synthesized with different amounts of chloroacetic acid. The graphics are taken from the publication "Size and morphology control over MOF-74 crystals" by C. Wu et al. (https://doi.org/10.1039/d4ra03943b) and reused under license CC BY-NC 3.0.

In summary, we observe, both in our own measurements and in those reported in the literature, that most MOF samples have crystal diameter between a few micrometers and a few tens of micrometers. This observation supports our choice of representative crystal sizes for the simulations reported in the manuscript: the minimum crystal size we chose (1 μm) is the typical, crystal size we see at the lower end of the distribution. The 10 μm size represents the typical, high-end of the distribution, while the 100 μm diameter is more of an outlier, or a worst-case scenario, and was used to confirm the crystal size sensitivity seen across scales.

To provide more context to the reader regarding our choices of representative crystal sizes, we added the following to the *"Molecular- to process-level evaluation"* sub-section of the *"Results"*.

> *"To illustrate the crystallite size dependence of the results, we have chosen three characteristic diameters: 1, 10, and 100 μm. The 1-μm and 10-μm values characterize the lower and upper bounds, respectively, of typical crystal size distributions taken from commercially available samples. The 100-μm value serves as an extrapolation for confirming the sensitivity observed at the 10 μm-level. Nevertheless, from a volume fraction perspective, larger crystallites dominate despite their lower occurrence."*

2. **Neglect of Water in Flue Gas:** The study focuses exclusively on dry flue gases, but in real-world conditions, flue gas typically contains significant amounts of water. The presence of water can drastically alter adsorption behavior and diffusion properties, potentially undermining the conclusions of this study. A discussion of how water affects the system, or at least an acknowledgment of this limitation, is necessary for a more complete assessment.

The point raised by the reviewer, namely, the inclusion of water adsorption in the process-level screening of carbon capture and sequestration (CCS) materials, is the ultimate challenge the scientific community is facing. To our knowledge, no research group has fully addressed this issue in process-level screening of MOFs. There have been a few reports addressing the issue, including one published in *Nature* [https://doi.org/10.1038/s41586-019-1798-7], but it solely relies on microscopic figures-of-merit such as selectivity.

The inherent challenge of inclusion of water in process-level materials screening comprises:

1. Simulating the adsorption of water at the molecular level with high accuracy.

2. Handling high affinity of water, as it becomes even more challenging to incorporate multicomponent adsorption in a process model.
3. Managing techno-economic uncertainty around drying the flue gas before CCS vs. uncertain performance implications for MOFs in humid CCS, including stability.

On the other hand, by looking at the experimental evidence for Zeolite 13X, we would like to point out that high affinity to water is not necessarily an application killer. Zeolite 13X has very high CO_2 capacity and good CO_2/N_2 selectivity. However, it was not considered as lead CCS sorbent due to the susceptibility of its performance to water adsorption. In a 2008 paper by Webley *et al.* [https://doi.org/10.1007/s10450-007-9100-y], it was shown that under cyclic steady state process conditions, water remains confined in the minority (<25%) part of the adsorption bed and, if designed properly, water leaves with CO_2 and the majority (>75%) of the bed remains dry and open for CO_2/N_2 separation. In such case, the performance of the process is still driven primarily by dry CO_2 and N_2 adsorption-based separation process. With above experimental result and possibility of flue gas drying, we believe our screening study with emphasis on inclusion of diffusivity has a sound scientific outcome.

Due to its hydrophobicity, with free energy of water adsorption as small as –8.1 kJ/mol, CALF-20 [https://doi.org/10.1126/science.abi7281] has been hailed as a promising material for CO_2 capture in humid environments. However, it has lower CO_2/N_2 selectivity (ΔG_{N2} - ΔG_{CO2}) compared to top 20 MOF candidates highlighted in our manuscript. A recent publication by Chung *et al.* [https://doi.org/10.1016/j.matt.2025.102140] on the CoRE-MOF-2024 database confirms that for 4% CO_2 flue gas (NGCC), CALF-20 does not achieve 90% recovery and 95% purity targets within a TSA process. The authors show that the choice of materials is essentially reduced to one (namely, `CIXDIZ_ASR`), out of entire CoRE-MOF-2024 database with 40,000+ materials, if one limits the starting candidate set to MOFs with low water affinity, like CALF-20. To minimize the energy requirement and maximize the productivity, one must carefully balance the tradeoff between lowering water affinity and increasing CO_2/N_2 selectivity, to enable wider choice of materials for scalable CCS process. This tradeoff in unavoidable, as both CO_2 and H_2O are polar molecules, with similar binding site preferences.

Without disputing the importance of evaluating water affinity, we would like to remind that the inclusion of material-specific, microscopic diffusion is another major unresolved challenge for the scientific community. This observation was acknowledged by all referees of the manuscript. In the present work, we initially set out to address the diffusion-related challenge and, therefore, made practical assumptions in relation to water that the work could be executed in a reasonable project time. While we acknowledge that this approach creates certain limitations, as is common in highly complex and multifaceted problems, we have focused on the inclusion of diffusion in the screening methodology. A methodology that treats, at the same time, the water

adsorption and material-specific microscopic diffusion at the highest level of detail, would be highly preferable, but might be less practical from an application perspective. To subsume, we believe the need for inclusion of microscopic diffusion is a top priority in scenarios where CO_2/N_2 selectivity is a target figure-of-merit, as it steers the search towards materials with smaller pores.

Notwithstanding, as we are building the simulation capabilities to address the ultimate materials screening challenge in a follow-up publication, we have carried out Widom particle insertion and Grand Canonical Monte Carlo (GCMC) for H_2O at 4 temperatures for the 982 MOFs simulated in the current manuscript. We include this additional adsorption data in the revised manuscript and in the released dataset.

In the revised manuscript, we address in the *"Discussion"* section the effects of including water in our analysis.

> *"In this work, we have focused on methodologically including microscopic diffusivity in computational material screening. To that end, we had to make reasonable simplifications to other equally challenging dimensions of material performance evaluation. In particular, we have assumed dry NGCC flue gas throughout the results reported in this manuscript. In order to address performance implications related to the presence of water, out of the 982 MOFs under consideration, we have removed those with higher water affinity than CALF-20 [https://doi.org/10.1126/science.abi7281] (ΔG_{H2O} ≤ −8.1 kJ/mol). As shown in Figure 6, 4 out of the 381 remaining MOFs, referred to as "water-tolerant", fall within the economically viable region. Note, that these 4 MOFs have CO_2, N_2 and H_2O affinities that are similar to or better than those reported for CALF-20, see supplementary information subsection S4.3. Interestingly, two of them have one-dimensional square pores similar to CALF-20. We would like to emphasize that, by virtue of including diffusion in the screening workflow, these water-tolerant MOFs would not be kinetically limited."*

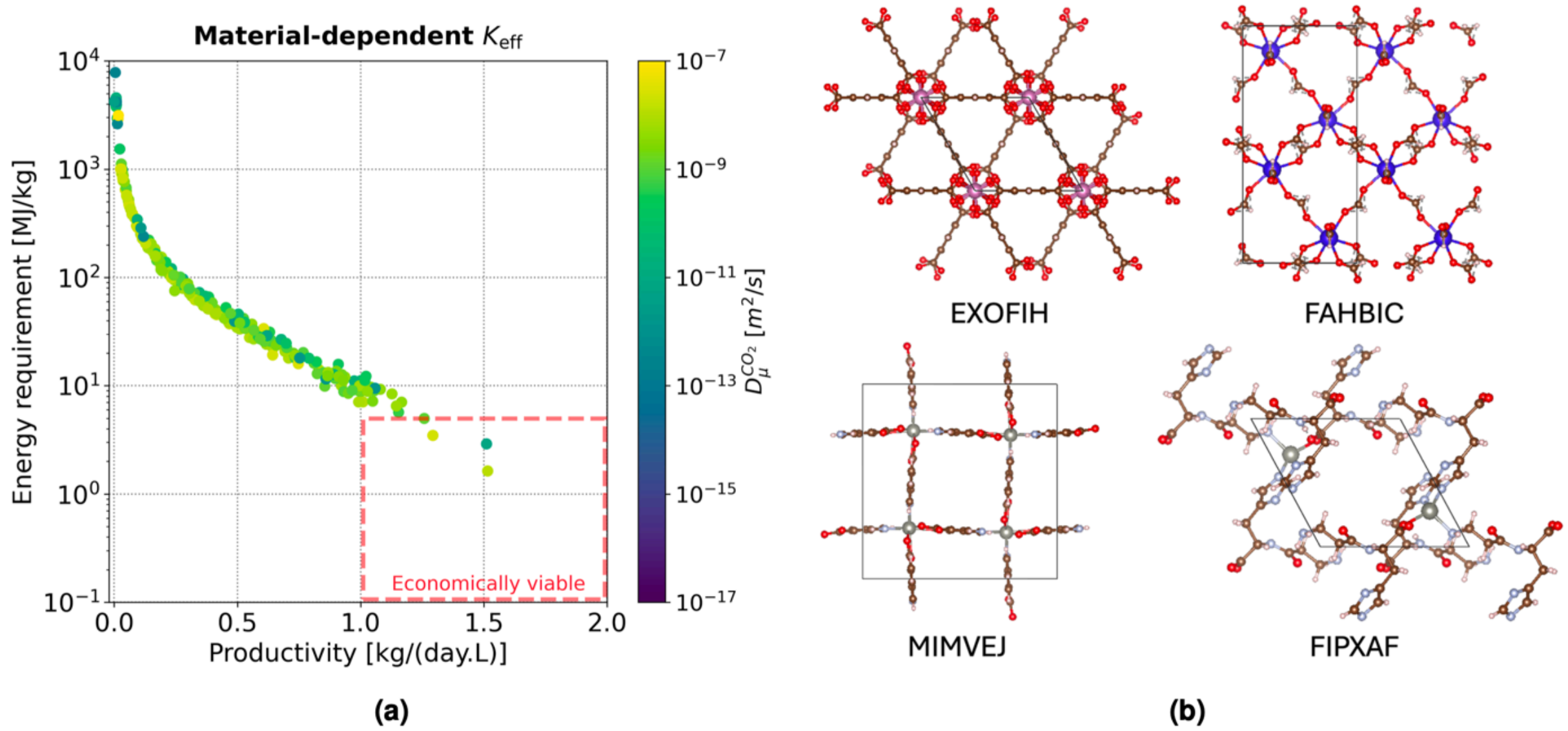

Figure 6. **Water-tolerant MOF candidates.** (a) Distribution of energy requirement *vs.* productivity for water-tolerant MOFs, i.e., with $\Delta G_{H_2O} > -8.1$ kJ/mol, assuming a crystallite diameter of $R_\mu = 10$ μm. We have identified 4 promising MOF structures in the economically viable region. (b) Crystal structures of the economically viable, water-tolerant MOF candidates.

We briefly describe the Widom particle insertion methodology and outcomes in the supplementary section S3.1

> *"We have used the Widom particle insertion method [https://doi.org/10.1002/aic.690470521] to probe the energy required for (or gained by) adding a probe molecule to the system, starting from an empty framework. CO_2, N_2 and H_2O were considered as probe molecules at temperatures T = 298, 323, 348 and 373 K. Each simulation performed 200,000 test insertions. From that, we have derived Henry constants at four temperatures, to which we have fitted ΔH and ΔS, from which we have then calculated the free energy of adsorption ΔG at 30 ºC in Table S11."*

Table S11. Free energy of adsorption at 30 °C for CO_2, N_2 and H_2O of the top 20 MOFs reported in this work, and CALF-20 (for comparison). All values calculated using the Widom particle insertion method.

MOF name	ΔG_{CO_2}, kJ/mol	ΔG_{N_2}, kJ/mol	ΔG_{H_2O}, kJ/mol
BOHGOU	-14.5	10.3	-65.2
VASFON	-14.6	3.9	-86.1
NATXIR	-13.8	7.2	-74.4
CDLGLU11	-13.9	13.3	-37.2
VASFED	-16.2	3.9	-90.1
CAKXET	-15.4	5.0	-97.7
AZAFIS	-18.6	0.9	-60.7
MOCJAQ	-12.7	11.6	-76.3
ESUMOU	-14.1	11.2	-52.0
CDLGLU01	-17.4	12.6	-39.1
MUXHIW01	-16.2	12.8	-66.9
MOCHES	-22.1	5.9	-99.4
IKETAU	-19.4	2.2	-89.2
RATVEP	-11.2	10.8	-63.1
MOCHIW	-23.1	5.8	-106.0
SUKREX	-16.5	2.6	-79.1
IPIJUM	-15.1	3.9	-67.3
NARMUR	-15.7	6.5	-106.5
MOCJEU	-11.6	12.5	-74.2
VIDXOX	-12.5	4.3	-66.9
CALF-20	-9.3	2.2	-8.1

Since the selection of these MOFs was mostly driven by CO_2/N_2 separation performance and CO_2 capacity, we expected that many of them would have strong water affinity, as both CO_2 and H_2O are polar molecules. As shown in Table S11, we report the free energy of adsorption of H_2O in the Henry region for the top 20 MOFs (the same as listed in Table S10). The ΔG_{H2O} values are very high compared to those of ΔG_{CO2} and ΔG_{N2}. The process modeling of these MOFs would be very challenging based on their very high H_2O affinity and the example water isotherm curves reported in Figure S10.

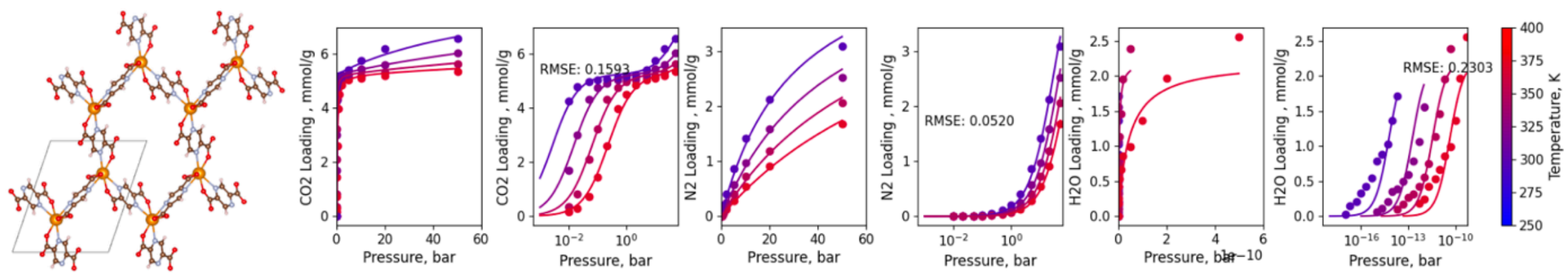

(a) VASFON (b) CO_2, N_2 and H_2O adsorption isotherms for VASFON.

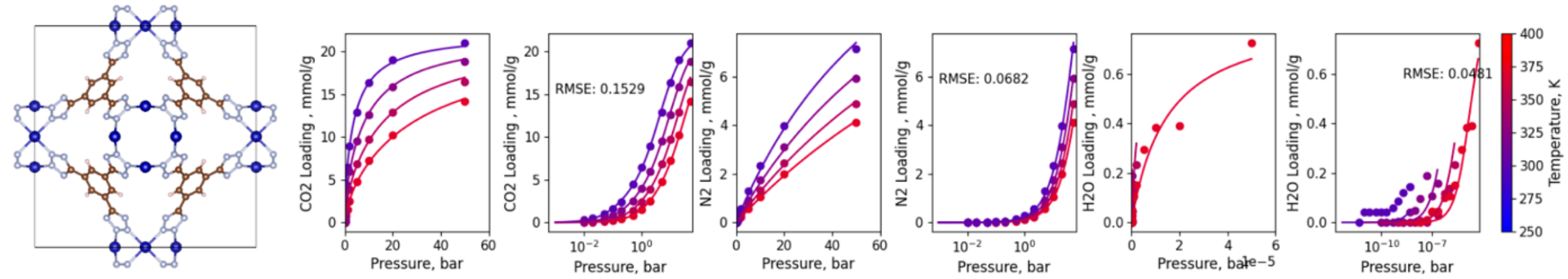

(c) BETGUE (d) CO_2, N_2 and H_2O adsorption isotherms for BETGUE.

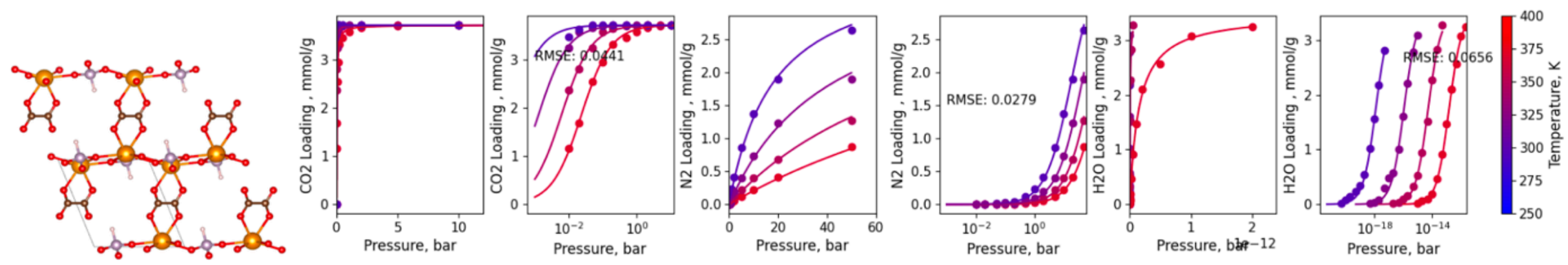

(e) MOCHIW (f) CO_2, N_2 and H_2O adsorption isotherms for MOCHIW.

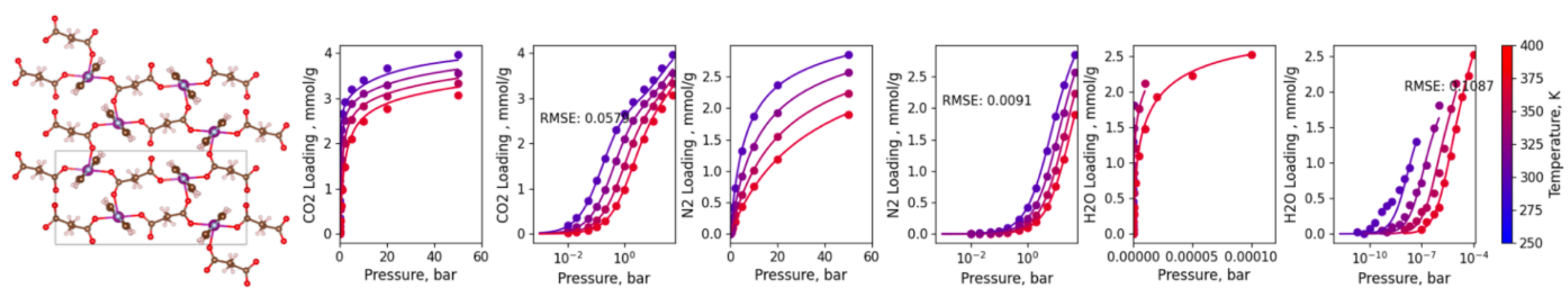

(g) EVOMOR (h) CO_2, N_2 and H_2O adsorption isotherms for EVOMOR.

Figure S10. MOF Structures and Isotherms (a) VASFON, (c) BETGUE, (e) MOCHIW and (g) EVOMOR structures and their associated adsorption isotherms (b), (d), (f) and (h), respectively. CO_2, N_2 and H_2O isotherm data (filled circles) and fitted models (lines).

Please refer to the revised manuscript for the full-resolution figure.

In order to further investigate the impact of water with our screening approach, we removed all MOFs which had water affinity higher than the CALF-20 benchmark, with *ΔG_{H2O} of –8.1 kJ/mol. The remaining 381 MOFs with lower water affinity are ranked according to volumetric productivity. We refer to them as "water-tolerant". As shown in Figure 6, 4 water-tolerant MOFs qualify for the "economically viable" criteria, i.e., Volumetric Productivity greater than 1 kg/(day.L) and Energy requirement lower than 5 MJ/kg. In Table S12, we present the relative rank order and free energy of adsorption for the top 4 water-tolerant MOFs. We would like to point out that these MOFs not only show lower water affinity, but also potentially higher CO_2/N_2 selectivity compared to*

CALF-20. In Figure S16, we present the crystal structures of these MOFs as well as full isotherms for CO_2, N_2 and H_2O. Few things we would like to highlight: 2 *out of 4 water-tolerant MOFs show one-dimensional square channels, which is a similar pore shape to that of CALF-20. The 4 economically viable, water-tolerant MOFs show substantially lower affinity to water compared to the top 20 MOFs discussed earlier in Table S11. This positive result reiterates our earlier point that balanced screening approach which allows one to test a trade-off between water affinity and CO_2/N_2 selectivity will enable scalable materials discovery.*

In the revised manuscript, we address the water affinity in the supplementary section entitled *"Water affinity of top performing MOFs for dry flue gas separation"*.

S4.3 Water affinity of top performing MOFs for dry flue gas separation

In our screening study, we have used dry flue gas to screen MOFs. To estimate the affinity of the screened MOFs towards water, we have calculated the Henry region free energy of adsorption for CO_2, N_2, H_2O and compared them to well-known CALF-20 [https://doi.org/10.1126/science.abi7281], see Table S11. Given that CO_2/N_2 selectivity and CO_2 capacity were included as criteria in the active learning material selection, but water affinity was not, the screening steered us towards a certain type of materials. The top 20 MOFs reported in our study have higher CO_2/N_2 selectivity (ΔG_{N2} - ΔG_{CO2}) than CALF-20, but also much higher water affinity (ΔG_{H2O}). This result reaffirms the inherent tradeoff between CO_2/N_2 selectivity and water affinity. In this work, we show that multiple MOF structures can achieve 90% recovery, and 95% purity target due to high selectivity under dry flue gas conditions.

To translate the Henry-region water affinities into adsorption isotherm data, we have simulated water adsorption isotherms for all MOFs reported in this manuscript. We have used a re-optimized TIP5P force field [https://doi.org/10.1063/1.1652434] to simulate water isotherms with a methodology similar to the one used for creating the CO_2 and N_2 isotherms reported in section S3.1. We plot water isotherm data for four representative MOFs in Figure S10. The high affinity of water is reflected in the very low partial pressure at which the materials saturate with water, at about 10^{-10} to 10^{-15} bar.

In order to assess the impact of water adsorption on the performance rank ordering, out of the 982 MOFs under consideration, we have removed those structures with higher water affinity than that of CALF-20, i.e. ΔG_{H2O} < -8.1 kJ/mol. The 381 remaining MOFs are referred to as "water-tolerant". As a key result shown in Figure 6, 4 out of the 381 remaining MOFs fall within the economically viable region. We provide their relative rank order, as well as their Widom-based free energies of adsorption in Table S12. Although these MOFs rank lower compared to the high selectivity MOFs in Table S10, they still show

CO_2, N_2 and H_2O affinities comparable to those of CALF-20. In Figure S16, we provide the structures and CO_2, N_2 and H_2O isotherms for these 4 economically viable, water-tolerant MOFs. Interestingly, 2 out of 4 have one-dimensional square channels similar to CALF-20.

Table S12. Rank order and free energy of adsorption at 30 °C for CO_2, N_2 and H_2O of the top 4 water-tolerant MOFs (i.e., with $\Delta G_{H_2O} > -8.1$ kJ/mol), and CALF-20 (for comparison). All ΔG values calculated using the Widom particle insertion method.

MOF	K_{eff} = const.	R_μ =1 μm	R_μ =10 μm	R_μ =100 μm	ΔG_{CO_2}, kJ/mol	ΔG_{N_2}, kJ/mol	ΔG_{H_2O}, kJ/mol
EXOFIH	173	147	145	118	-10.5	-0.4	-8.0
FAHBIC	177	150	148	150	-7.8	5.9	-5.1
MIMVEJ	246	208	206	187	-10.3	-1.3	1.6
FIPXAF	260	222	220	200	-9.0	0.8	-5.6
CALF-20					-9.3	2.2	-8.1

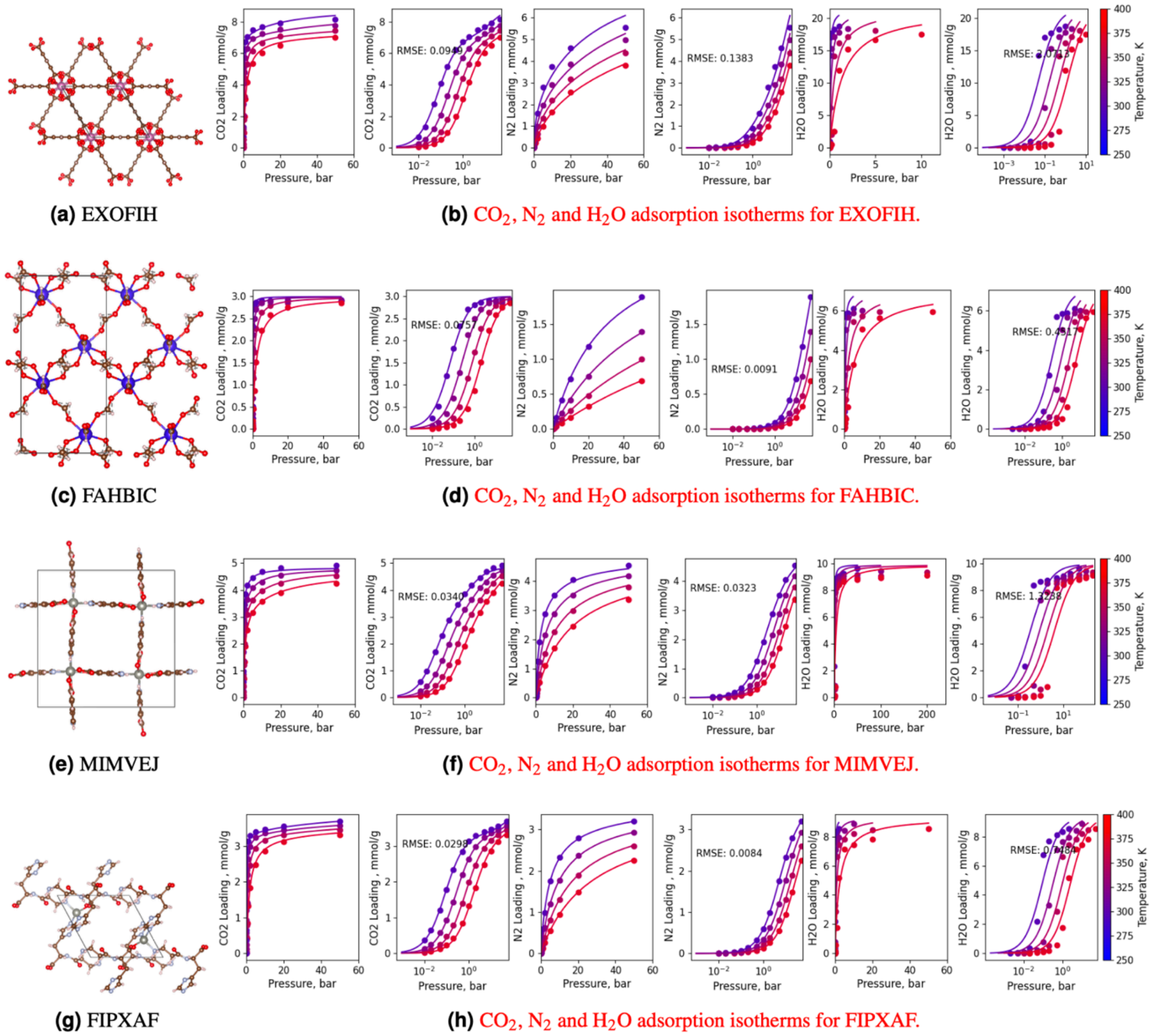

Figure S16. **Water-tolerant MOF structures and isotherms** (a) EXOFIH, (c) FAHBIC, (e) MIMVEJ and (g) FIPXAF structures and their associated adsorption isotherms (b), (d), (f) and (h), respectively. CO_2, N_2 and H_2O isotherm data (filled circles) and fitted models (lines).

3. **Lack of Essential Methodological Details:** Several critical aspects of the methodology are either vague or insufficiently detailed, making it difficult to assess the validity of the findings. Examples include:

- The statement, "We have identified those providing the best balance between energy cost, productivity, and recovery," is too general. These metrics should be explicitly quantified to support the conclusions.

We agree with the reviewer that the statement, in its original form, was too general. This statement was in the first paragraph of the *"Results"* section and was used merely as an introduction to the detailed presentation of results that immediately followed. The

ranking of materials considers the productivity as the first criterion and the energy requirement as the second. Only materials whose separation performance achieves 95% CO_2 purity are considered, as this is a requirement for post-combustion capture applications.

We changed the statement to make it more specific; it now reads as this:

> *"Within the remaining 6,484 MOFs, we have identified those structures providing the highest productivity associated with the lowest energy requirement, as defined in subsection S4.2, while achieving a purity of at least 95% as required for CO_2 capture applications at NGCC power plants. In the following sections, we detail how this evaluation was performed."*

- The description of the active-learning model for predicting CO_2 and N_2 adsorption free energy is unclear. Adsorption free energy depends on temperature and partial pressure, and its relevance to the process should be clarified. It appears that the authors may be considering the Henry regime, but if so, this should be explicitly stated. Furthermore, Henry's selectivity is known to be a poor predictor of performance in many cases (see DOI: 10.1038/s41586-024-07683-8).

As pointed out by reviewers, we had not provided sufficient explanation in the main text on the calculation of free energies of adsorption based on the isotherm data from GCMC simulations in the context of the ML model. The expanded explanation of the isotherm model and free energy calculation is found in the supplementary section S3.1 but we have expanded it now in S2, bullet point 7. To address the reviewer's concerns, we have now provided additional details. The free energies are calculated at an adsorption temperature of 30 ºC. The free energies are based on the fitted isotherm model described in section S3.1.

We share the reviewer's concern that just using free energies or free energy differences will basically represent Henry-region selectivity. To account for CO_2 capacity (at 4% CO_2) in addition to free energy difference, we would have to consider the CO_2 free energy of adsorption (ΔG_{CO2}) and CO_2 saturation capacity separately. However, we found that for 4% CO_2 at 1 bar, most of the MOFs under consideration were far from saturated – hence the 4% CO_2 adsorption capacity is well captured by ΔG_{CO2} in the Henry regime. Thus, to represent the CO_2/N_2 selectivity and CO_2 capacity simultaneously, in the multi-objective Pareto search procedure described in the S2 (bullet 7), we weighted CO_2 binding free energy twice as much as N_2 binding free energy (ΔG_{N2} - 2 x ΔG_{CO2}).

To validate our choice of free energy of adsorption as described above, we have calculated the equilibrium CO_2 loading as fraction of the saturation loading (q/q_{sat}) for the 4% NGCC flue gas using the multicomponent isotherm models for 982 MOFs screened in this manuscript. The equilibrium fractional coverage, plotted as histogram in Figure

S4a, confirms that 759 out of 982 MOFs show fractional CO_2 loading less than 15% - putting them well within the Henry region of adsorption.

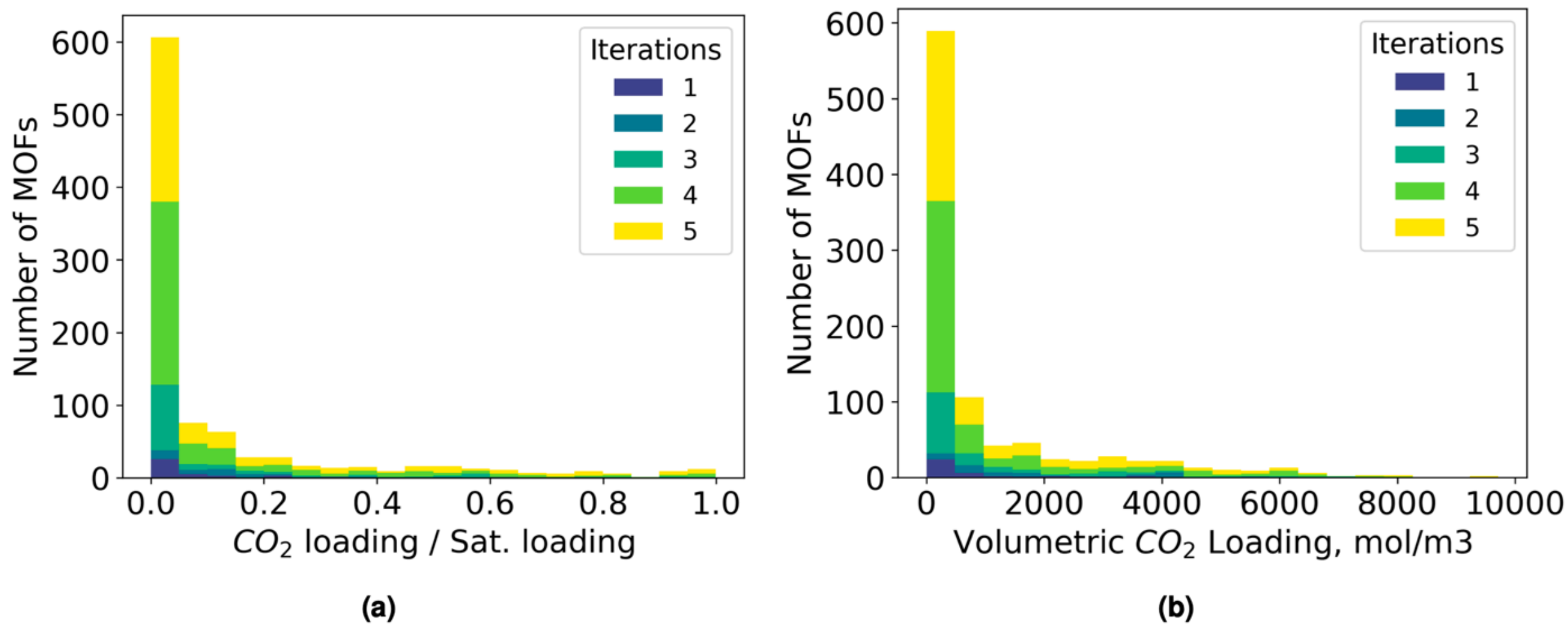

Figure S4. Distribution of (a) fractional and (b) volumetric CO_2 loading calculated using isotherm model for 4% CO_2 NGCC flue gas at 30 °C. The stacked bars show the 982 recommended materials across 5 active learning iterations.

For improving clarity, we have added additional explanation on the calculation of free energies of adsorption in the main manuscript, *"Results"*,

> *"The material selection improves CO_2 vs. N_2 selectivity as well as CO_2 capacity, and selects materials with lower SCScore [https://doi.org/10.1021/acs.jcim.7b00622] as well as lower prediction uncertainty. Given that selectivity has been shown to be a poor descriptor of separation performance [https://doi.org/10.1038/s41586-024-07683-8], we have assigned higher priority to ΔG_{CO2}, as it represents both selectivity and capacity of 4% CO_2 NGCC flue gas in the Henry regime. Further details are provided in the Methods section."*

in the "Methods" section,

> *"The Pareto search is performed by optimally trading-off ΔG_{N2} against $-2\times\Delta G_{CO2}$ to maximize CO_2/N_2 selectivity and CO_2 capacity simultaneously. Within each Pareto front, the selected materials are sorted and filtered based on their SCScore and based on the prediction uncertainty for adsorption free energy."*

as well as in the Supporting Information section S2

> *"2. **Feature Selection**: Using the results from the latest GCMC iteration, select a subset of features that are strong predictors of two key properties, namely, the free adsorption energies of CO_2 (ΔG_{CO2}) and N_2 (ΔG_{N2}) at 30 ºC. Features are selected before the model estimation process. To enforce uniformity, features are*

sub-selected using a combination of Lasso [https://doi.org/10.1111/j.2517-6161.1996.tb02080.x], variable contribution from random-forest ensembles [https://doi.org/10.1186/1471-2105-9-307] and Shapley-inspired [https://dx.doi.org/10.1109/ACCESS.2021.3119110] metrics. Here, the free energy of adsorption (∆G) is calculated based on Enthalpy (∆H) and Entropy (∆S) of adsorption determined by a Langmuir isotherm model fit.

*"7. **Pareto search and MOF selection**: Using the above design criteria, a multi-objective search is conducted over the entire space to simultaneously*

(a) Minimize ΔG_{CO2} (with higher importance) – stronger CO_2 adsorption capacity
(b) Maximize ΔG_{N2} - weaker N_2 adsorption
(c) Minimize SCScore – simpler organic linker
(d) Minimize model prediction uncertainty

This is enforced by doing a formal Pareto search only on the first two criteria above. The Pareto approach follows the formalism established by the Pareto Differential Algorithm (PDE) [https://doi.org/10.1109/CEC.2001.934295]. For each solution in the database, the key properties (i.e., free energies of adsorption for CO_2 and N_2) are calculated using the selected models as well as their associated uncertainty estimates. The Pareto front is estimated using the dual maximization of $-\Delta G_{CO2}$ and ΔG_{N2}. We found that for 4% CO_2 at 1 bar, most of the MOFs under consideration are far from saturated – hence the 4% CO_2 adsorption capacity is well represented by the free energy of CO_2 adsorption in the Henry regime. To account for both selectivity and capacity of CO_2 in the Henry regime, we weighed ΔG_{CO2} more strongly, effectively maximizing ΔG_{N2} - 2 x ΔG_{CO2} in the Pareto search procedure, alongside synthesis complexity score (SCScore) and model prediction uncertainty. To validate our choice of Pareto search using the free energy scheme described above, we plot the distribution of fractional CO_2 loading for all 982 MOFs using a multi-component isotherm model. The fractional loading is calculated using the isotherm model for NGCC composition (4% CO_2) at 30 °C. The histogram in Figure S4a shows that most of the MOFs (759 out of 982) have fractional CO_2 loading below 15% for NGCC flue gas adsorption. This suggests that, for 4% NGCC flue gas, CO_2 loading is well within the Henry region for most of the MOFs considered here."

and supplementary section S3.1

"Multi-site Langmuir models were fitted to the simulated adsorption isotherm data with $S \in \{1,2,3\}$ sites using SciPy optimization library for $i \in \{CO_2, N_2, H_2O\}$ species

$$q_i^{eq}(P,T) = \sum_{j=1}^{S} q_{i,j}^{sat} \frac{b_{i,j}(T)\cdot P}{1+b_{i,j}(T)\cdot P}$$

where $q_{i,j}^{sat}$ and $b_{i,j}(T)$ are the saturation capacity and equilibrium parameter associated with species i and site j, respectively. The S-site Langmuir model that achieved the lowest RMSE for CO_2 was selected to represent the simulated isotherms for all species, as shown in Figure S3. The equilibrium parameters $b_{i,j}(T)$ can be modeled interchangeably by the Arrhenius and Van't Hoff equations by expressing the pre-exponential factor of the former as the temperature-independent term of the latter. For representing the isotherm at adsorption temperature, we calculate the free energy of adsorption, $\Delta G_{i,j}(30\,°C)$, using the estimated parameters from the Van't Hoff equation. Typically, at lower CO_2 partial pressure, such as in NGCC flue gas, the site with the lowest free energy of adsorption for CO_2 plays the most important role. The free energy values of this site are used in the active learning-based materials selection described in supplementary section S2. The resulting analytical expressions for the adsorption as a function of pressure and temperature were input to the process-level model equations with modifications to account for multi-component adsorption as described by supplementary equations S5 as function of pressure, temperature and composition.

In addition to providing extensive documentation on how the free energies of adsorption were obtained, we would like to point out that those were not the sole metrics used to pre-select materials for multi-scale simulations. We have also considered the Synthesis Complexity Score (SCScore) and the model prediction uncertainty. We did so to decouple the microscopic material preselection and macroscopic (process-based) screening in two separate steps. After going through iterative MOF microscopic performance improvement, we have attempted ML modeling for process-based performance of the materials using all available material features, including free energy of adsorption for CO_2 and N_2 (microscopic performance features). This exercise reveals that material performance (for constant diffusivity) can be predominantly explained by the ΔG-related features. This finding indirectly confirms the suitability of our choice of using free energies of adsorption as one of the three criteria for ML materials preselection.

Finally, we thank the reviewer for sharing with us valuable references on this topic. Garcia *et al.* [https://doi.org/10.1038/s41586-024-07683-8] have shown that, as a material-level KPI, Henry selectivity is not a good predictor of TEA- and LCA-level KPIs for the cement plant scenario, which contains much higher CO_2 concentration than in NGCC flue gas. But the same can be said about the process-level KPIs – purity (Pu)

and productivity (P) – even though they are one of the inputs to the subsequent analysis layers. The difference between our observation for 4% CO_2 NGCC flue gas and the conclusions by Garcia *et al.* could be (at least partly) explained by the different CO_2 concentrations in the various scenarios considered. At lower concentrations, as show for our case in Figure S4, the Henry selectivity will be naturally more relevant as a performance indicator.

- The discussion of diffusion coefficients lacks critical details. The authors compute the self-diffusion coefficient, whereas the transport diffusion coefficient is the more relevant parameter for process performance. While these values coincide at zero loading, they diverge significantly at saturation (where the self-diffusion coefficient drops to zero while the transport diffusion coefficient approaches infinity). The manuscript should specify the conditions under which the diffusion coefficient is computed and justify its relevance to the studied system.

We thank the reviewer for pointing out ways of improving the manuscript. In its original form, Supplementary Information subsection S2.2 contained the conditions under which the self-diffusion coefficient was computed. In the revised version, we clarify these details.

> *"The Molecular Dynamics simulations for the calculation of diffusion coefficients were performed aiming for a constant, and finite, volumetric loading of adsorbed molecules following $1/R_c^3$. Given that R_c = 12.8 Å and the simulation box has at least $(2R_c)^3$ volume, this leads to a volumetric loading no larger than 792 mol/m^3, which translates to a low gravimetric loading of the order of 1 mol/kg. This led to different numbers of molecules per simulation box for each material, given that each structure had a different unit cell size."*

In this work, we have used effective mass transfer coefficient to define the mass transfer between the gas phase and sorbent phase. Typically, effective mass transfer describes independent diffusion of CO_2, N_2 and H_2O. In the constant diffusivity approximation, these mass transfer coefficients are based on a commonly used value of 0.15 s^{-1} [https://doi.org/10.1039/C6FD00040A]. This specific value of the mass transfer coefficient corresponds to a 2 mm pellet diameter with intra-particle diffusivity of 5×10^{-8} m^2/s. Given that most publications compare performance of separation at constant diffusivity, we have included microscopic diffusion based on transport diffusivity at low loading, where these coefficients coincide. In the low partial pressure regime of NGCC, or, equivalently, at the desorption temperature of 110 ºC, the CO_2 loading is sufficiently low to justify this approximation.

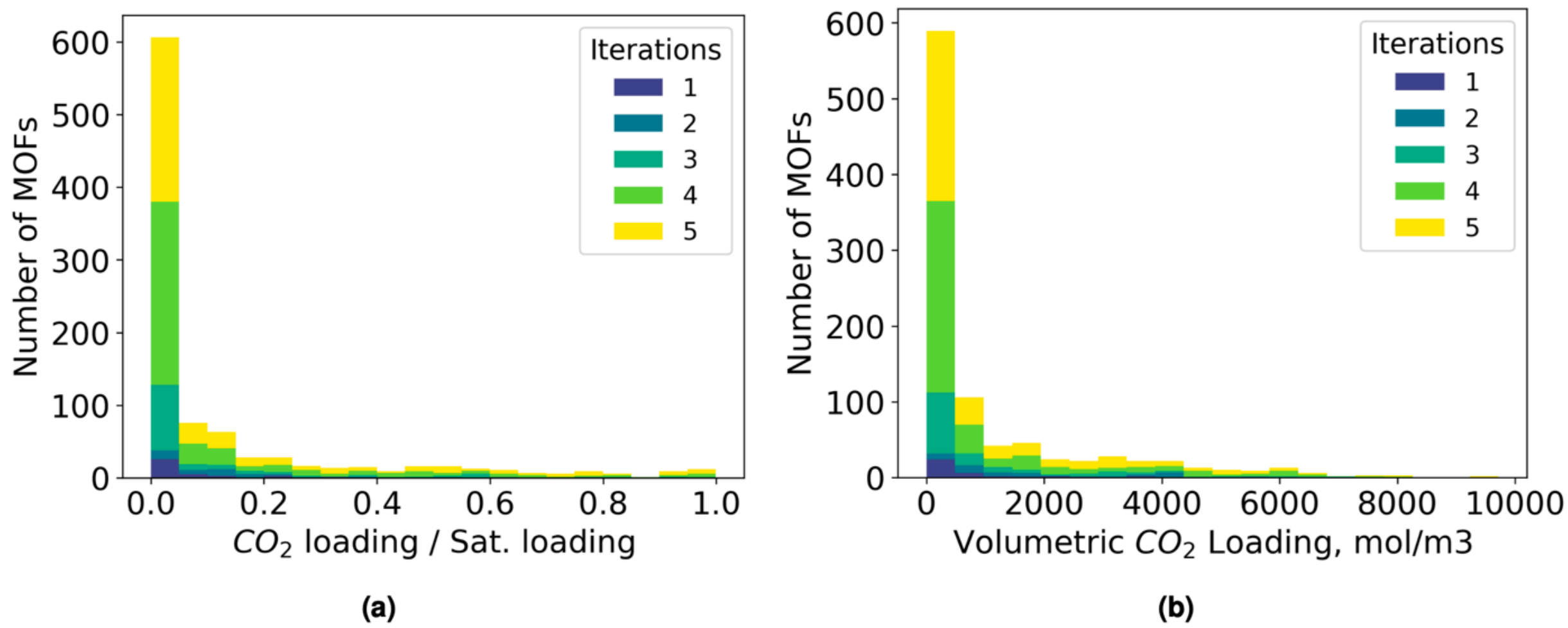

Figure S4. Distribution of (a) fractional and (b) volumetric CO_2 loading calculated using isotherm model for 4% CO_2 NGCC flue gas at 30 °C. The stacked bars show the 982 recommended materials across 5 active learning iterations.

Using the isotherm model developed for all 982 MOFs, we calculate the equilibrium volumetric CO_2 loading at adsorption conditions (4% NGCC flue gas at 30 ºC). The distribution of these volumetric CO_2 loadings in Figure S4b show that 692 out of 982 MOFs have CO_2 loading is less than the 792 mol/m^3 low loading threshold described in section S2.2. In this low loading regime, self-diffusivity approaches transport diffusivity. Under dynamic simulation (both for adsorption and desorption) the actual instantaneous loading should be much lower than the reported equilibrium loading shown. We anticipate that our approximations are sufficiently accurate to provide a simple description of molecular diffusion at screening level.

We have added additional explanation on this approximation to the revised manuscript, in the *"Molecular-level simulations"* subsection of the *"Results"*

> *"Finally, we have used the fitted expression for $D_\mu(T)$ as transport diffusivity under low loading approximation for the process modeling."*

and in the subsection S4.1 of the Supplementary Information, entitled *"Temperature-swing adsorption process"*

> *"We equate the temperature dependent self-diffusivity to transport diffusivity under the approximation of low loading. To justify this, we have calculated equilibrium volumetric CO_2 loadings using a multi-component isotherm model for all 982 MOFs. For 692 of them, the calculated equilibrium loading of CO_2 is lower than the 792 mol/m^3 loading threshold provided in subsection S3.2. The distribution of the equilibrium volumetric CO_2 loading is plotted in Figure S4b."*

Conclusion

The topic of this study is undoubtedly important, and considering diffusion in MOF-based carbon capture is a necessary step forward. However, the manuscript raises too many concerns in its current form. Addressing these issues is essential before the work can be considered for publication.

In addition, I do not see how the authors can justify the title. My understanding of the manuscript is that diffusion limitation reduces the performance of materials, while the title suggests an enhancement related to diffusion. The title is intriguing and got my attention, but I was disappointed after reading the manuscript.

The authors would like to point out that the title *"Molecular diffusion enhanced performance evaluation of metal-organic frameworks for CO_2 capture"* means that it was the *performance evaluation*, i.e., our ability to evaluate the performance of MOFs for carbon capture in computational screening, that was *enhanced* by including molecular diffusion methodologically. We did not mean that the carbon dioxide capture performance of the screened materials was enhanced through the explicit consideration of molecular diffusion. We hope this clarifies the title choice.

Reviewer #2

The article by Ferreira et al. highlights the importance of molecular diffusion, a factor often overlooked in screening studies. Its key contribution is providing a systematic approach to incorporating diffusion into screening methods. The study is interesting.

The authors are very grateful for the recognition of the importance of our work. We hope that the revised version addresses all the perceived limitations and deserves your support for publication in *Nature Communications*.

However, the implementation has significant limitations that reduce its practical relevance:

- The model does not account for the effect of water. While diffusion could be an important factor in material exclusion, water presence in the flue gas stream is even more critical. The authors assume a drying step upstream of the capture unit, which considering the flue gas volumes and scale of the operation, can render a very costly process. Hence, a much more realistic scenario would be to incorporate the water in the flue gas stream and conduct the screening study under such conditions.

The point raised by the reviewer, namely, the inclusion of water adsorption in the process-level screening of carbon capture and sequestration (CCS) materials, is the ultimate challenge the scientific community is facing. To our knowledge, no research group has fully addressed this issue in process-level screening of MOFs. There have been a few reports addressing the issue, including one published in *Nature* [https://doi.org/10.1038/s41586-019-1798-7], but it solely relies on microscopic figures-of-merit such as selectivity.

The inherent challenge of inclusion of water in process-level materials screening comprises:

4. Simulating the adsorption of water at the molecular level with high accuracy.
5. Handling high affinity of water, as it becomes even more challenging to incorporate multicomponent adsorption in a process model.
6. Managing techno-economic uncertainty around drying the flue gas before CCS vs. uncertain performance implications for MOFs in humid CCS, including stability.

On the other hand, by looking at the experimental evidence for Zeolite 13X, we would like to point out that high affinity to water is not necessarily an application killer. Zeolite 13X has very high CO_2 capacity and good CO_2/N_2 selectivity. However, it was not considered as lead CCS sorbent due to the susceptibility of its performance to water adsorption. In a 2008 paper by Webley *et al.* [https://doi.org/10.1007/s10450-007-9100-y], it was shown that under cyclic steady state process conditions, water remains confined in the minority (<25%) part of the adsorption bed and, if designed properly, water leaves with CO_2 and the majority (>75%) of the bed

remains dry and open for CO_2/N_2 separation. In such case, the performance of the process is still driven primarily by dry CO_2 and N_2 adsorption-based separation process. With above experimental result and possibility of flue gas drying, we believe our screening study with emphasis on inclusion of diffusivity has a sound scientific outcome.

Due to its hydrophobicity, with free energy of water adsorption as small as –8.1 kJ/mol, CALF-20 [https://doi.org/10.1126/science.abi7281] has been hailed as a promising material for CO_2 capture in humid environments. However, it has lower CO_2/N_2 selectivity (ΔG_{N2} - ΔG_{CO2}) compared to top 20 MOF candidates highlighted in our manuscript. A recent publication by Chung *et al.* [https://doi.org/10.1016/j.matt.2025.102140] on the CoRE-MOF-2024 database confirms that for 4% CO_2 flue gas (NGCC), CALF-20 does not achieve 90% recovery and 95% purity targets within a TSA process. The authors show that the choice of materials is essentially reduced to one (namely, `CIXDIZ_ASR`), out of entire CoRE-MOF-2024 database with 40,000+ materials, if one limits the starting candidate set to MOFs with low water affinity, like CALF-20. To minimize the energy requirement and maximize the productivity, one must carefully balance the tradeoff between lowering water affinity and increasing CO_2/N_2 selectivity, to enable wider choice of materials for scalable CCS process. This tradeoff in unavoidable, as both CO_2 and H_2O are polar molecules, with similar binding site preferences.

Without disputing the importance of evaluating water affinity, we would like to remind that the inclusion of material-specific, microscopic diffusion is another major unresolved challenge for the scientific community. This observation was acknowledged by all referees of the manuscript. In the present work, we initially set out to address the diffusion-related challenge and, therefore, made practical assumptions in relation to water that the work could be executed in a reasonable project time. While we acknowledge that this approach creates certain limitations, as is common in highly complex and multifaceted problems, we have focused on the inclusion of diffusion in the screening methodology. A methodology that treats, at the same time, the water adsorption and material-specific microscopic diffusion at the highest level of detail, would be highly preferable, but might be less practical from an application perspective. To subsume, we believe the need for inclusion of microscopic diffusion is a top priority in scenarios where CO_2/N_2 selectivity is a target figure-of-merit, as it steers the search towards materials with smaller pores.

Notwithstanding, as we are building the simulation capabilities to address the ultimate materials screening challenge in a follow-up publication, we have carried out Widom particle insertion and Grand Canonical Monte Carlo (GCMC) for H_2O at 4 temperatures for the 982 MOFs simulated in the current manuscript. We include this additional adsorption data in the revised manuscript and in the released dataset.

In the revised manuscript, we address in the *"Discussion"* section the effects of including water in our analysis.

"In this work, we have focused on methodologically including microscopic diffusivity in computational material screening. To that end, we had to make reasonable simplifications to other equally challenging dimensions of material performance evaluation. In particular, we have assumed dry NGCC flue gas throughout the results reported in this manuscript. In order to address performance implications related to the presence of water, out of the 982 MOFs under consideration, we have removed those with higher water affinity than CALF-20 [https://doi.org/10.1126/science.abi7281] (ΔG_{H2O} ≤ −8.1 kJ/mol). As shown in Figure 6, 4 out of the 381 remaining MOFs, referred to as "water-tolerant", fall within the economically viable region. Note, that these 4 MOFs have CO_2, N_2 and H_2O affinities that are similar to or better than those reported for CALF-20, see supplementary information subsection S4.3. Interestingly, two of them have one-dimensional square pores similar to CALF-20. We would like to emphasize that, by virtue of including diffusion in the screening workflow, these water-tolerant MOFs would not be kinetically limited."

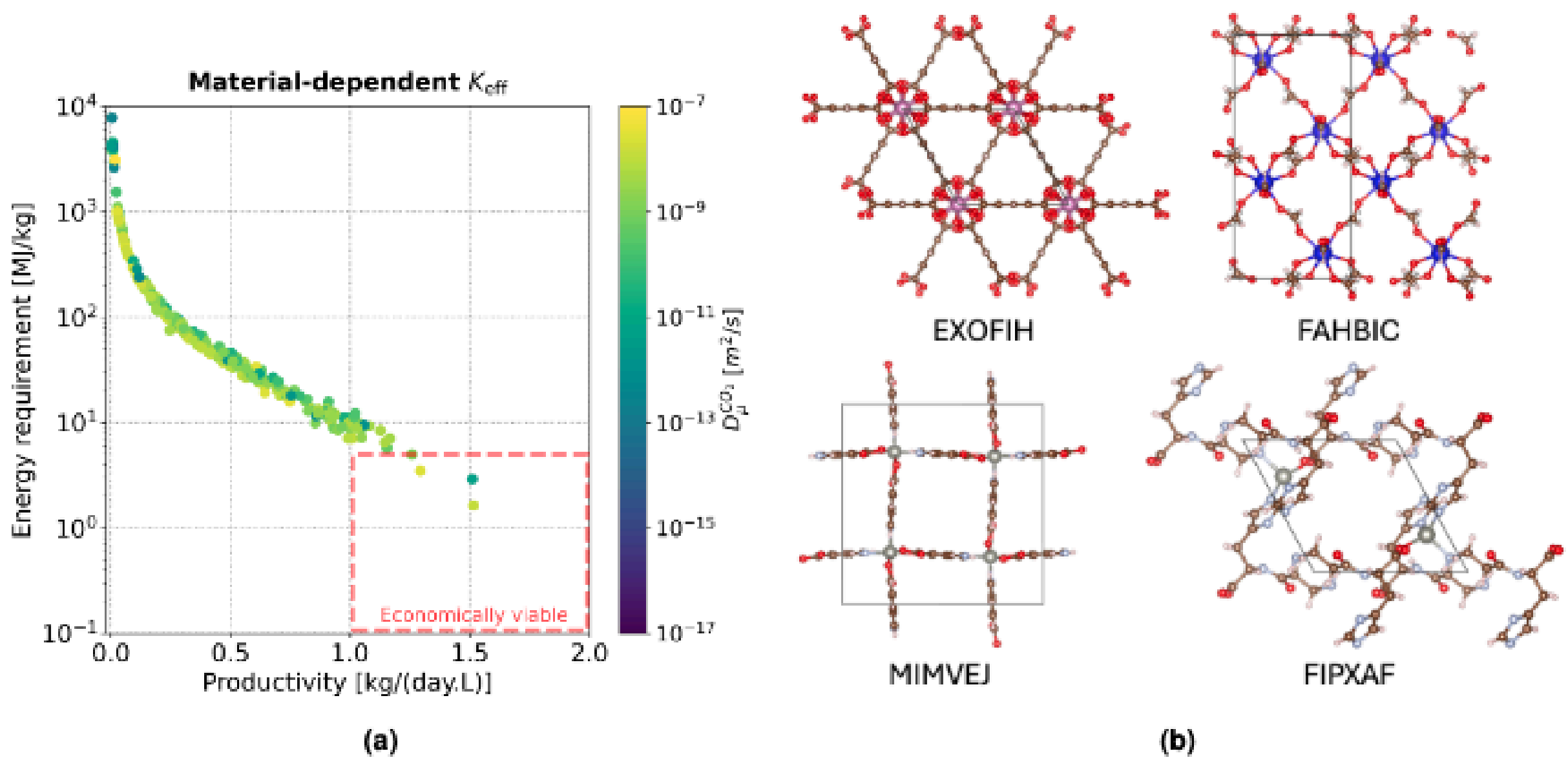

Figure 6. **Water-tolerant MOFs.** (a) Distribution of Energy requirement *vs.* Productivity for water-tolerant MOFs (i.e., with $\Delta G_{H_2O} > -8.1$ kJ/mol), assuming crystallite diameter $R_\mu = 10$ µm. We identify 4 such MOFs in the economically viable region. (b) Crystal structures of the economically viable, water-tolerant MOFs.

We briefly describe the Widom particle insertion methodology and outcomes in the supplementary section S3.1

"We have used the Widom particle insertion method [https://doi.org/10.1002/aic.690470521] to probe the energy required for (or gained by) adding a probe molecule to the system, starting from an empty framework. CO_2, N_2 and H_2O were considered as probe molecules at temperatures T = 298, 323, 348 and 373 K. Each simulation performed 200,000 test insertions. From that, we have derived Henry constants at four temperatures,

to which we have fitted ΔH and ΔS, from which we have then calculated the free energy of adsorption ΔG at 30 ºC in Table S11."

Table S11. Free energy of adsorption at 30 °C for CO_2, N_2 and H_2O of the top 20 MOFs reported in this work, and CALF-20 (for comparison). All values calculated using the Widom particle insertion method.

MOF name	ΔG_{CO_2}, kJ/mol	ΔG_{N_2}, kJ/mol	ΔG_{H_2O}, kJ/mol
BOHGOU	-14.5	10.3	-65.2
VASFON	-14.6	3.9	-86.1
NATXIR	-13.8	7.2	-74.4
CDLGLU11	-13.9	13.3	-37.2
VASFED	-16.2	3.9	-90.1
CAKXET	-15.4	5.0	-97.7
AZAFIS	-18.6	0.9	-60.7
MOCJAQ	-12.7	11.6	-76.3
ESUMOU	-14.1	11.2	-52.0
CDLGLU01	-17.4	12.6	-39.1
MUXHIW01	-16.2	12.8	-66.9
MOCHES	-22.1	5.9	-99.4
IKETAU	-19.4	2.2	-89.2
RATVEP	-11.2	10.8	-63.1
MOCHIW	-23.1	5.8	-106.0
SUKREX	-16.5	2.6	-79.1
IPIJUM	-15.1	3.9	-67.3
NARMUR	-15.7	6.5	-106.5
MOCJEU	-11.6	12.5	-74.2
VIDXOX	-12.5	4.3	-66.9
CALF-20	-9.3	2.2	-8.1

Since the selection of these MOFs was mostly driven by CO_2/N_2 separation performance and CO_2 capacity, we expected that many of them would have strong water affinity, as both CO_2 and H_2O are polar molecules. As shown in Table S11, we report the free energy of adsorption of H_2O in the Henry region for the top 20 MOFs (the same as listed in Table S10). The ΔG_{H2O} values are very high compared to those of ΔG_{CO2} and ΔG_{N2}. The process modeling of these MOFs would be very challenging based on their very high H_2O affinity and the example water isotherm curves reported in Figure S10.

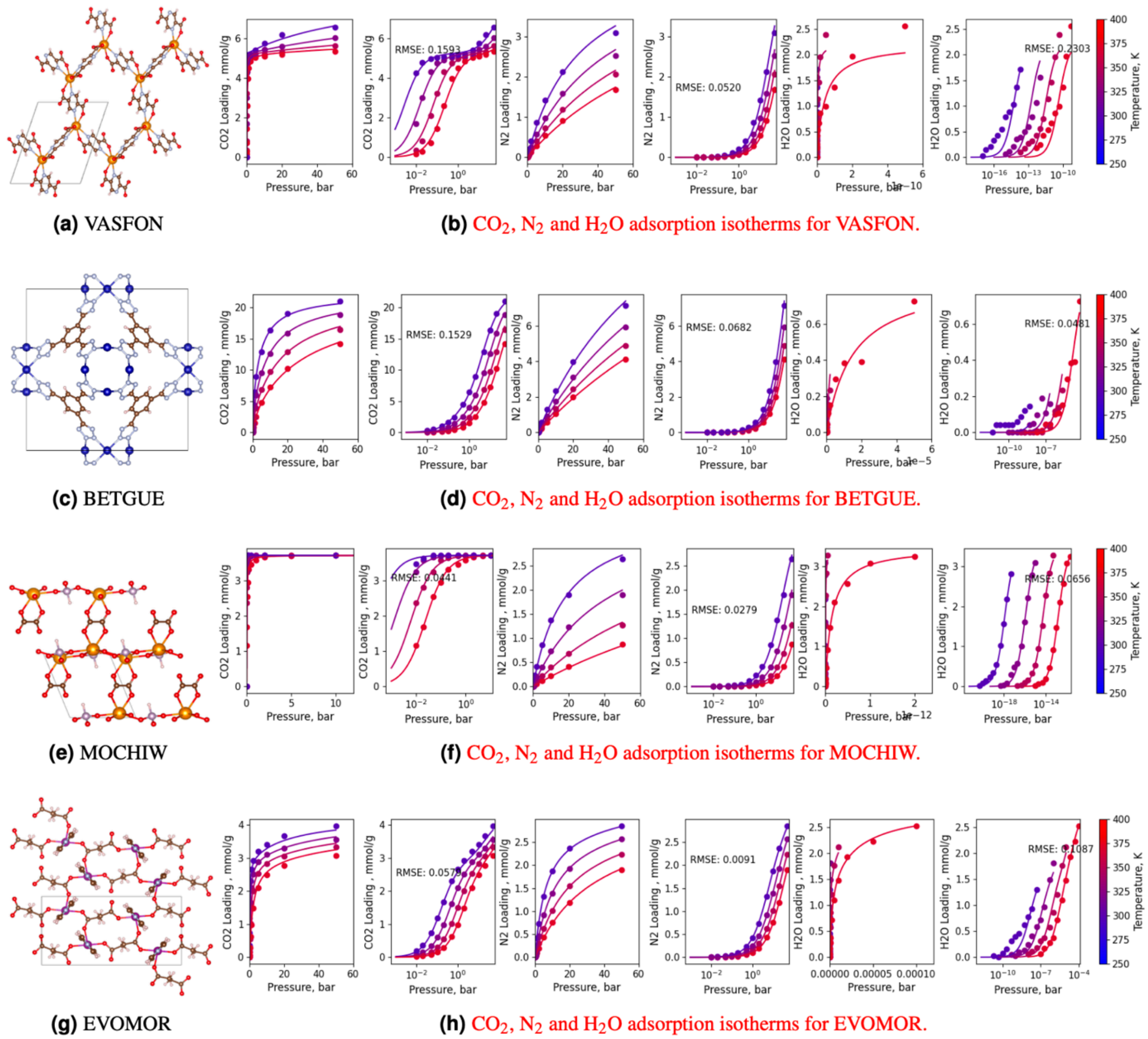

Figure S10. **MOF Structures and Isotherms** (a) `VASFON`, (c) `BETGUE`, (e) `MOCHIW` and (g) `EVOMOR` structures and their associated adsorption isotherms (b), (d), (f) and (h), respectively. CO_2, N_2 and H_2O isotherm data (filled circles) and fitted models (lines).

Please refer to the revised manuscript for the full-resolution figure.

In order to further investigate the impact of water with our screening approach, we removed all MOFs which had water affinity higher than the CALF-20 benchmark, with *ΔG_{H2O} of –8.1 kJ/mol. The remaining 381 MOFs with lower water affinity are ranked according to volumetric productivity. We refer to them as "water-tolerant". As shown in Figure 6, 4 water-tolerant MOFs qualify for the "economically viable" criteria, i.e., Volumetric Productivity greater than 1 kg/(day.L) and Energy requirement lower than 5 MJ/kg. In Table S12, we present the relative rank order and free energy of adsorption for the top 4 water-tolerant MOFs. We would like to point out that these MOFs not only show lower water affinity, but also potentially higher CO_2/N_2 selectivity compared to*

CALF-20. In Figure S16, we present the crystal structures of these MOFs as well as full isotherms for CO_2, N_2 and H_2O. Few things we would like to highlight: 2 *out of 4 water-tolerant MOFs show one-dimensional square channels, which is a similar pore shape to that of CALF-20. The 4 economically viable, water-tolerant MOFs show substantially lower affinity to water compared to the top 20 MOFs discussed earlier in Table S11. This positive result reiterates our earlier point that balanced screening approach which allows one to test a trade-off between water affinity and CO_2/N_2 selectivity will enable scalable materials discovery.*

In the revised manuscript, we address the water affinity in the supplementary section entitled *"Water affinity of top performing MOFs for dry flue gas separation"*.

S4.3 Water affinity of top performing MOFs for dry flue gas separation

In our screening study, we have used dry flue gas to screen MOFs. To estimate the affinity of the screened MOFs towards water, we have calculated the Henry region free energy of adsorption for CO_2, N_2, H_2O and compared them to well-known CALF-20 [https://doi.org/10.1126/science.abi7281], see Table S11. Given that CO_2/N_2 selectivity and CO_2 capacity were included as criteria in the active learning material selection, but water affinity was not, the screening steered us towards a certain type of materials. The top 20 MOFs reported in our study have higher CO_2/N_2 selectivity (ΔG_{N2} - ΔG_{CO2}) than CALF-20, but also much higher water affinity (ΔG_{H2O}). This result reaffirms the inherent tradeoff between CO_2/N_2 selectivity and water affinity. In this work, we show that multiple MOF structures can achieve 90% recovery, and 95% purity target due to high selectivity under dry flue gas conditions.

To translate the Henry-region water affinities into adsorption isotherm data, we have simulated water adsorption isotherms for all MOFs reported in this manuscript. We have used a re-optimized TIP5P force field [https://doi.org/10.1063/1.1652434] to simulate water isotherms with a methodology similar to the one used for creating the CO_2 and N_2 isotherms reported in section S3.1. We plot water isotherm data for four representative MOFs in Figure S10. The high affinity of water is reflected in the very low partial pressure at which the materials saturate with water, at about 10^{-10} to 10^{-15} bar.

In order to assess the impact of water adsorption on the performance rank ordering, out of the 982 MOFs under consideration, we have removed those structures with higher water affinity than that of CALF-20, i.e. ΔG_{H2O} < -8.1 kJ/mol. The 381 remaining MOFs are referred to as "water-tolerant". As a key result shown in Figure 6, 4 out of the 381 remaining MOFs fall within the economically viable region. We provide their relative rank order, as well as their Widom-based free energies of adsorption in Table S12. Although these MOFs rank lower compared to the high selectivity MOFs in Table S10, they still show

CO_2, N_2 and H_2O affinities comparable to those of CALF-20. In Figure S16, we provide the structures and CO_2, N_2 and H_2O isotherms for these 4 economically viable, water-tolerant MOFs. Interestingly, 2 out of 4 have one-dimensional square channels similar to CALF-20.

Table S12. Free energy of adsorption at 30 °C for CO_2, N_2 and H_2O of the 4 water-tolerant MOFs (i.e., with $\Delta G_{H_2O} > -8.1$ kJ/mol), and CALF-20 (for comparison). All ΔG values calculated using the Widom particle insertion method.

MOF	K_{eff} = const.	R_μ =1 µm	R_μ =10 µm	R_μ =100 µm	ΔG_{CO_2}, kJ/mol	ΔG_{N_2}, kJ/mol	ΔG_{H_2O}, kJ/mol
EXOFIH	173	147	145	118	-10.5	-0.4	-8.0
FAHBIC	177	150	148	150	-7.8	5.9	-5.1
MIMVEJ	246	208	206	187	-10.3	-1.3	1.6
FIPXAF	260	222	220	200	-9.0	0.8	-5.6
CALF-20					-9.3	2.2	-8.1

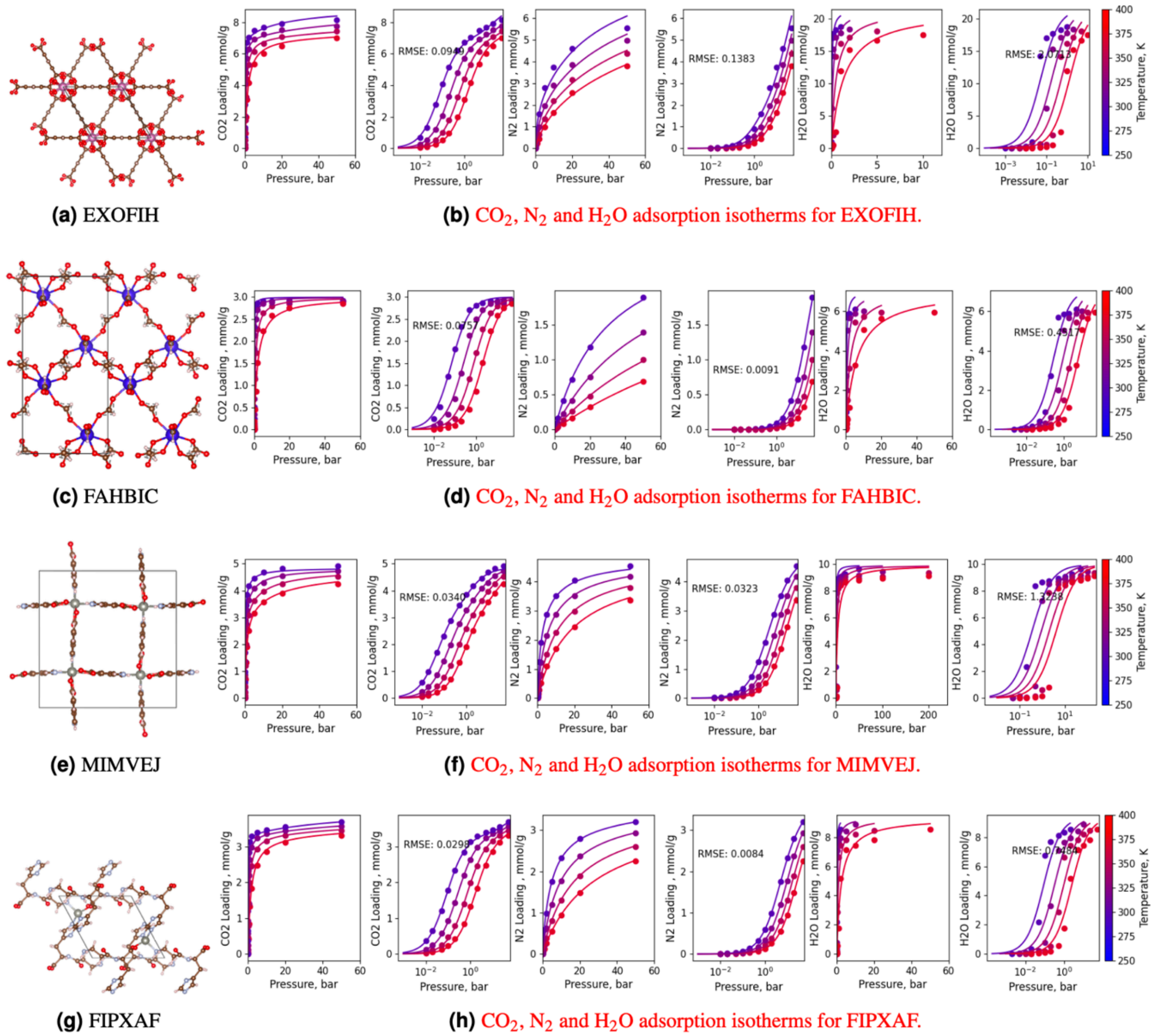

Figure S16. **Water-tolerant MOF structures and isotherms** (a) `EXOFIH`, (c) `FAHBIC`, (e) `MIMVEJ` and (g) `FIPXAF` structures and their associated adsorption isotherms (b), (d), (f) and (h), respectively. CO_2, N_2 and H_2O isotherm data (filled circles) and fitted models (lines).

- The active learning model targets high CO_2/N_2 selectivity. Thus, selectivity is used as a primary screening criterion for top-performing materials, despite studies indicating that selectivity alone is a poor predictor of real-world performance. There is a clear lack of integration between the process model and the molecular properties calculations in the ML model feedback loop. Such integration would provide a much more interesting and relevant approach, where molecular diffusion can properly be incorporated in the analysis at the screening stage. In my opinion, the title of the article is overpromising and not a true reflection of what it is done in the study.

We agree with the reviewer that selectivity alone is a poor predictor of real-world performance. This is why we did not use it as a primary screening criterion for top-performing materials. In the following, we clarify what was done in this work.

The CO_2/N_2 selectivity and CO_2 capacity (ΔG_{N2} - 2xΔG_{CO2}) is used as one of multiple pre-screening criteria employed by the active learning model. This model is only responsible for recommending good leads to undergo an extensive multi-scale simulation campaign and, therefore, does not have the final word on predicting the real-world performance of materials. The Pareto search and MOF recommendation model employed a multi-objective fitness function which aimed to minimize the CO_2 adsorption free energy, maximize the N_2 adsorption free energy, minimize the Synthetic Complexity Score (SCScore) of the organic linker and address the prediction uncertainty. The full description of the active learning model is contained in Supplementary Section S2, entitled "Active learning structure selection model".

We have added more explanation on the criteria used by the active learning model to preselect good leads for simulation in the main manuscript *"Results",*

> *"The material selection improves CO_2 vs. N_2 selectivity as well as CO_2 capacity, and selects materials with lower SCScore [https://doi.org/10.1021/acs.jcim.7b00622] as well as lower prediction uncertainty. Given that selectivity has been shown to be a poor descriptor of separation performance [https://doi.org/10.1038/s41586-024-07683-8], we have assigned higher priority to ΔG_{CO2}, as it represents both selectivity and capacity of 4% CO_2 NGCC flue gas in the Henry regime. Further details are provided in the Methods section."*

in the "Methods" section,

> *"The Pareto search is performed by optimally trading-off ΔG_{N2} against $-2\times\Delta G_{CO2}$ to maximize CO_2/N_2 selectivity and CO_2 capacity simultaneously. Within each Pareto front, the selected materials are sorted and filtered based on their SCScore and based on the prediction uncertainty for adsorption free energy."*

as well as in the Supporting Information section S2

> *"7. **Pareto search and MOF selection**: Using the above design criteria, a multi-objective search is conducted over the entire space to simultaneously*
> *(a) Minimize ΔG_{CO2} (with higher importance) – stronger CO_2 adsorption capacity*
> *(b) Maximize ΔG_{N2} - weaker N_2 adsorption*
> *(c) Minimize SCScore – simpler organic linker*
> *(d) Minimize model prediction uncertainty*

This is enforced by doing a formal Pareto search only on the first two criteria above. The Pareto approach follows the formalism established by the Pareto Differential Algorithm (PDE) [https://doi.org/10.1109/CEC.2001.934295]. For each solution in the database, the key properties (i.e., free energies of adsorption for CO_2 and N_2) are calculated using the selected models as well as their associated uncertainty estimates. The Pareto front is estimated using the dual maximization of -ΔG_{CO2} and ΔG_{N2}. We found that for 4% CO_2 at 1 bar, most of the MOFs under consideration are far from saturated – hence the 4% CO_2 adsorption capacity is well represented by the free energy of CO_2 adsorption in the Henry regime. To account for both selectivity and capacity of CO_2 in the Henry regime, we weighed ΔG_{CO2} more strongly, effectively maximizing ΔG_{N2} - 2 x ΔG_{CO2} in the Pareto search procedure, alongside synthesis complexity score (SCScore) and model prediction uncertainty. To validate our choice of Pareto search using the free energy scheme described above, we plot the distribution of fractional CO_2 loading for all 982 MOFs using a multi-component isotherm model. The fractional loading is calculated using the isotherm model for NGCC composition (4% CO_2) at 30 °C. The histogram in Figure S4a shows that most of the MOFs (759 out of 982) have fractional CO_2 loading below 15% for NGCC flue gas adsorption. This suggests that, for 4% NGCC flue gas, CO_2 loading is well within the Henry region for most of the MOFs considered here."

In addition, we would like to point out that our choice of free energies of adsorption, SCScore of organic linker and uncertainty in the ML model predictions was driven by our intention to split the microscopic material selection and the macroscopic (process-based) screening into two separate steps. After going through iterative MOF microscopic performance improvement, we attempted ML modeling for process-based performance of the materials using all available material features, including free energy of adsorption for CO_2 and N_2 (microscopic performance features). This exercise showed us that material performance (for constant diffusivity) can be predominantly explained by the ΔG-related features. This finding indirectly confirms the suitability of our choice of using free energies of adsorption as one of the three criteria for ML materials preselection.

We acknowledge that an active learning recommendation model that incorporates microscopic diffusion from the very beginning (at pre-screening) would have steered us faster towards the top-performance materials that we eventually found. However, we opted to not include microscopic diffusion into the pre-screening stage in order to establish the constant diffusivity approximation as a baseline, because it is the *de facto* standard way in which similar studies were done. Only by establishing this baseline, we were then able to quantify the percentage of "false leads" that were identified upon appropriately considering the material-specific microscopic diffusivity.

Finally, without belittling the utility of the active learning model to pre-screen the database for promising materials to simulate, we would like to stress that the focus of this work was the incorporation of multiscale diffusivity simulation. The ML model was

there just to support and narrow the candidate list (pre-screening), so we could reserve the most powerful tools only for the most promising candidate materials.

- I have several concerns when it comes to the process modelling part as the text is confusing. In page 9 ("process level simulations" section), it is mentioned that: "The competitive adsorption of CO_2, N_2 and H_2O is modelled as a multi-component mixed Langmuir isotherm derived from the combination of pure component isotherms". The adsorption step was conducted under dry conditions so not clear to me for which step this competitive adsorption was calculated. Obviously, since they are making use of direct heating with steam in the purge and desorption steps of the process, the water isotherms need then to be known and calculated for such steps. There is no mention at all about water isotherms in the manuscript and they are not presented. This is a major concern and there is a lack of clarity on how they have done the calculations.

We thank reviewer for pointing out our oversight. Although we included water in our description of process modeling, it was not at the same level of rigor as for CO_2 and N_2. In our attempt to build a generalized modeling platform, we made provisions for including water as a separate species in our process modeling. However, our treatment of water was simplified in the absence of isotherms and for the reasons explained below.

As shown in Figure S9, for the process modeling of regeneration, we used both indirect heating (with hot water at 105 ºC as the heat transfer fluid) as well as steam for direct heating. By turning off direct or indirect heating, one at a time, we realized that most of the heat transfer is actually driven by indirect heating, and the role of direct steam is reduced to merely that of an inert purge gas. To provide more direct evidence of this peculiar observation, we simulated an example TSA cycle where, instead of using 105 ºC steam directly in the bed during regeneration, we used 30 ºC Nitrogen to see the effect on the solid sorbent temperature at the exit of the bed. In Figure L3, we compare the temperature profiles for the first adsorption and regeneration cycle using each of these two scenarios. It turns out there is hardly any difference between the two temperature profiles. The 30 ºC N_2 purge scenario achieves slightly lower temperature, which could also be due to the higher mass flow rate caused by the lower molecular weight, or the fact that the purge stream is in fact colder. From the materials screening point of view, it is essential track the amount of steam (and, consequently, energy) required in its reduced role as a purge gas. To account for the energy requirement in the absence of calculated isotherms for water, we used CO_2 isotherms and CO_2 diffusivities to represent the water during regeneration. This allowed us to use an inert representation of water that competes with CO_2 at the same time it allowed us to calculate energy requirement of steam purge during regeneration.

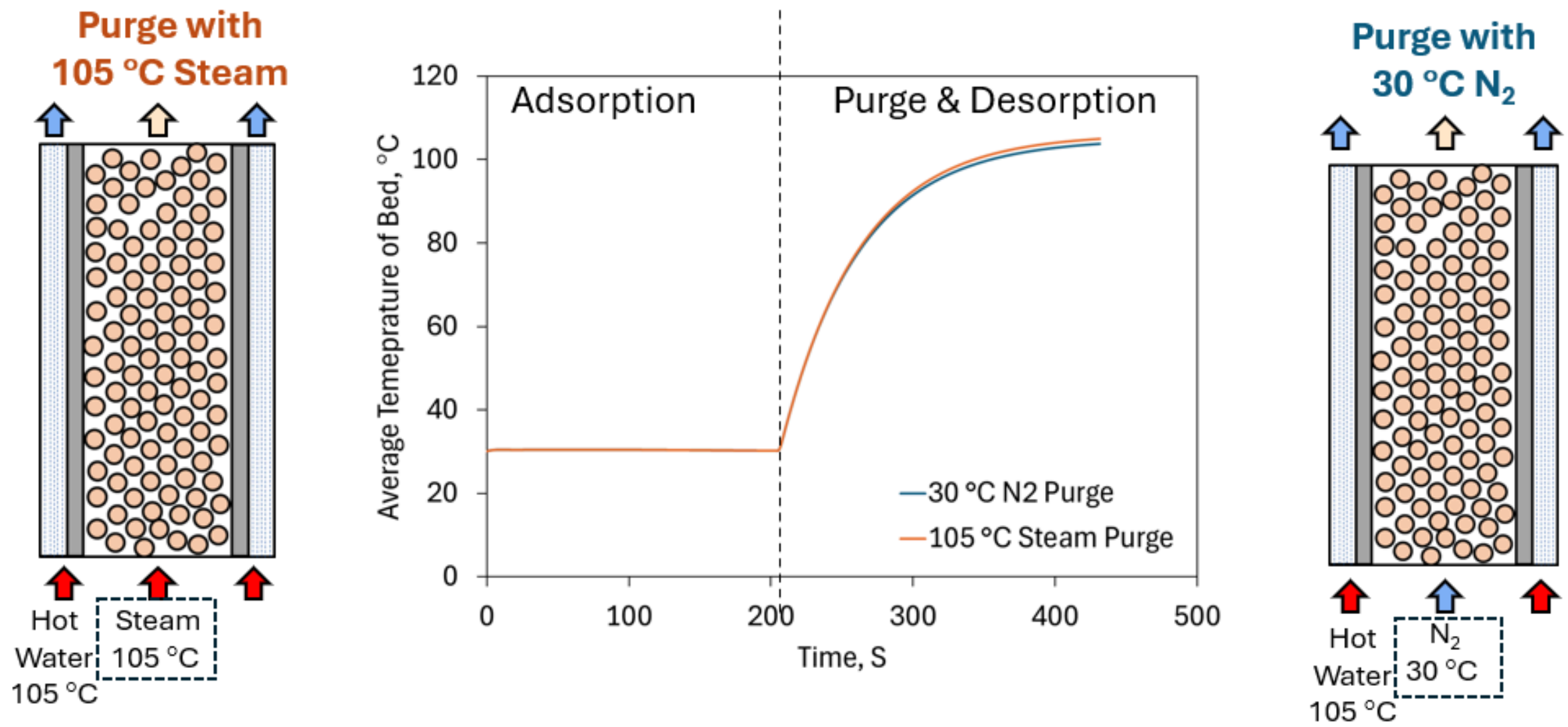

Figure L3. Temperature profile of the bed with both direct and indirect heating with steam (orange), as well as with only indirect heating (blue) during purge and regeneration

For MOFs with high affinity to water, the option of steam regeneration would not be practical, and the only option would be TVSA (temperature vacuum swing adsorption) regeneration. This also raises the point that, for the ultimate materials screening, multiple process modeling options needs to be evaluated simultaneously (e.g., flue gas drying, TSA, PSA, VPSA, TVSA etc.). This type of holistic screening including all possible process options is beyond the scope of current manuscript and has not been addressed by anyone we know so far.

To avoid the misrepresentation and offer further clarification we modify the *"Process-level simulations"* method description,

> *"The competitive adsorption of CO_2 and N_2 is modeled as a multi-component, mixed Langmuir isotherm derived from the combination of pure component isotherms during adsorption. The H_2O is included in the process modeling as an inert purge gas during regeneration, with the purpose of accounting for the steam requirement within the specific energy evaluation."*

and the supplementary section S4.1 accordingly

> *"Heating is achieved by simultaneously flowing steam and pressurized hot water, both at 105 °C, through the packed bed and heating jacket of the vessel, respectively. By comparing the effectiveness of indirect heat transfer between the heating jacket and direct heating by steam, we find that the heat transfer is dominated by the heating jacket, and the role of steam is reduced to that of a mere inert purge medium for desorbed CO_2."*

...

"In the subsequent purge step, the bed is heated as previously described, leading to N_2 desorption and purge from inter-particle bed volume. The N_2-rich purged gas is discarded. In the following desorption step, due to continued heating (up to 105 °C), stronger-bound CO_2 is desorbed and collected from the outlet as product alongside the steam used for heating the bed. In order to represented steam as a purge medium with reasonably competitive isotherm, we have resorted to representing the steam (H_2O) isotherm and the water diffusivity as if they were the same as in CO_2. The main purpose of the simplifying assumptions is to represent the steam/energy requirement at screening-level accuracy."

- *The details for the calculation of the energy cost are not provided in the text and there are misleading statements: for instance, caption in Figure 4: "Effect of molecular diffusion on CO_2 capture cost and productivity". It is the energy cost what it is provided and not the CO_2 capture cost.*

We apologize for the lack of clarity and for the imprecise use of the term "energy cost". Throughout the manuscript, what we present is always the "energy requirement", expressed in MJ/kg. We have changed the entire manuscript accordingly to standardize the nomenclature used.

The energy requirement is calculated by integrating the heat flux through the external heating jacket and the specific energy of the steam used in steam purge with respect to cooling water as the reference during the heating steps of the TSA cycle (N_2 purge and CO_2 desorption). The reported specific energy does not include the energy of CO_2 compression, or any additional energy needed for pumps, blower fans etc.

We provide additional details on the calculation of "energy requirement" in the supporting information subsection S4.2, to which we refer the readers when the expression is first used.

"The process modeling outcome also includes the trade-offs between the process-based figures of merits (i.e., CO_2 purity, recovery, specific energy consumption and volumetric productivity) reported in Figure S13. The energy requirement is calculated by integrating the total energy input from external heating jacket and specific energy of steam used to purge N_2 and CO_2, during N_2 purge and CO_2 desorption steps, respectively. Our specific energy calculation does not include any additional contribution, including CO_2 compression, nor pump or blower energy."

- I find Figure 4b-d very confusing and suggest replotting for clarity purposes.

We acknowledge that these are complex and busy figures, especially when shrunk to fit one third of a column width. For clarity, we moved sub-figures (b) and (d) to the Supplementary Information, where the results for crystallite radii R_μ = *1 μm* and R_μ = *100 μm* are discussed. We only keep (the original) sub-figure (c) in the main manuscript, as it relates to crystallite radius R_μ = *10 μm*, which is the one used as a reference throughout the main manuscript.

To better explain what can be learned from panel (c), we added a new panel (d) which puts into perspective the changes in performance caused by the inclusion of microscopic diffusion in the evaluation. We introduce terminology in the *"Process-level optimization and materials ranking"* subsection to help us describe these results.

> *"We introduce terminology for describing groups of materials at different performance screening levels. We call "promising candidates", those out of the 6,484 initial structures that were preselected by the active learning model to undergo multi-scale simulations. Among those, we term "process-feasible" (PF) those whose simulated process-level performance achieves 95% CO_2 purity and 90% CO_2 recovery targets. Finally, the subset of PF materials that achieve CO_2 productivity higher than 1 kg/(day.L) and energy requirement lower than 5 MJ/kg of CO_2 is labeled "economically viable" (EV)."*
>
> *...*
>
> *"In Figure 4b and Figure 4c, we have identified the structures whose process-level performance was most affected. We compare their performance coordinates before and after inclusion of molecular diffusivity for R_μ = 10 μm. For reference, we plot open symbols in their original positions taken from the left panel of Figure 4a. The arrows indicate MOFs that moved out of the EV region once their structure-dependent, molecular diffusivity was included into the calculation of K_{eff}. The percentage of promising candidates that achieved the purity/recovery process feasibility targets was 81% under the constant K_{eff} approximation, but some of them no longer achieved these targets after the inclusion of microscopic diffusivity. The relative reduction in the PF population grows from 1%, to 2%, to 5% with increasing crystallite diameter R_μ = 1, 10 and 100μm, respectively. We now turn our attention to the subset of EV materials, which correspond to 28% of the promising candidates and 35% of the PF population, under the constant K_{eff} approximation. In this case, the relative reduction of the EV population was 14%, 16% and 20% for R_μ = 1, 10 and 100 μm, respectively. We term these materials as "false leads, as they would have been wrongly ranked high despite their low performance due to diffusivity constraints.*

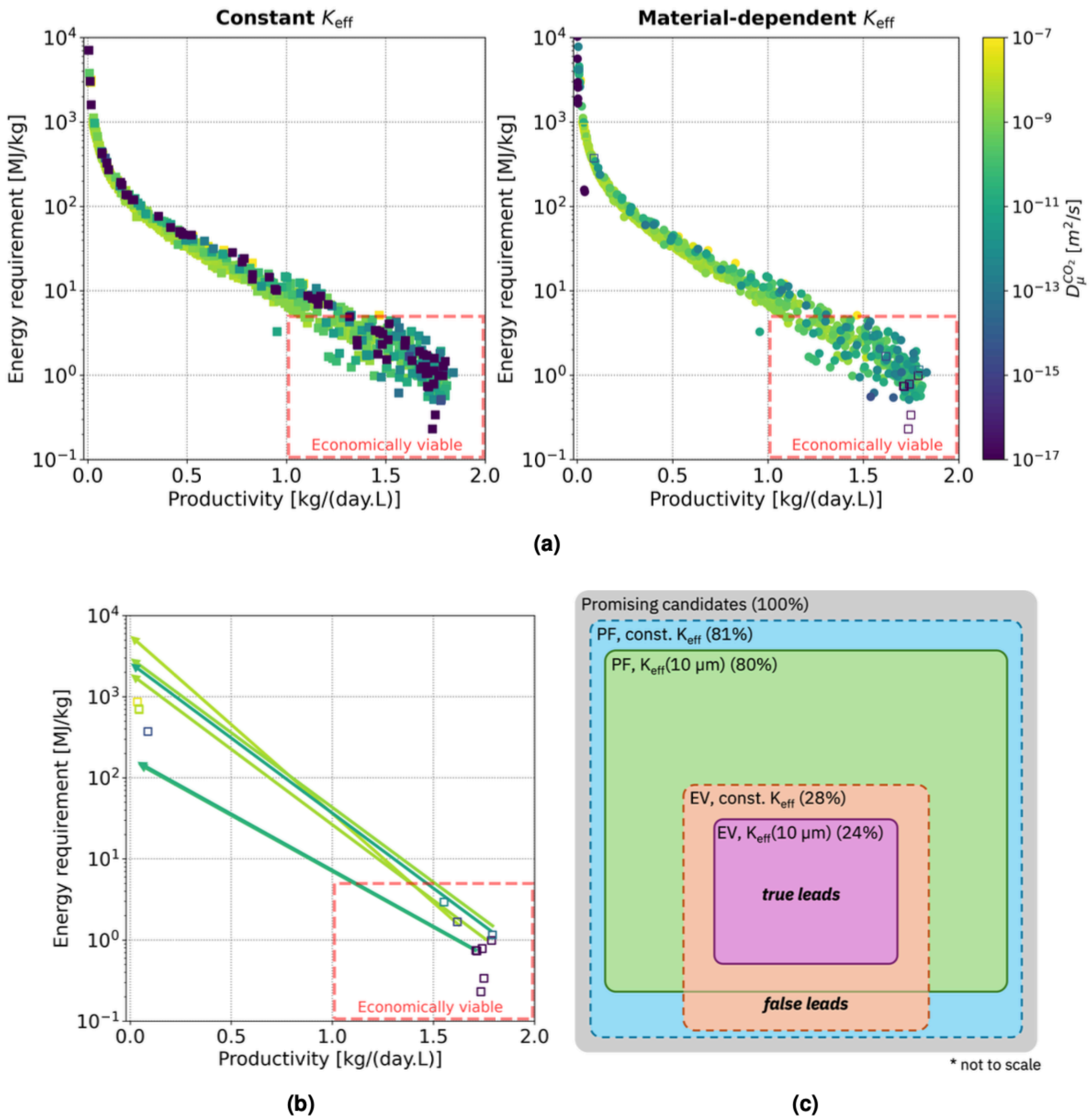

Figure 4. **Effect of molecular diffusion on energy requirement and productivity.** (a) Distribution of energy requirement *vs.* productivity for a crystallite diameter $R_\mu = 10$ μm, assuming a constant K_{eff} (left) and a material-dependent K_{eff} (right) that accounts for microscopic diffusivity. The respective CO_2 diffusion coefficients are indicated by color. Solid squares represent "process-feasible" (PF) materials, i.e., those whose performance achieved 95% purity and 90% recovery, while open squares mark the positions of excluded materials whose performance failed to meet those targets. The dashed rectangle delineates the "economically viable" (EV) region with productivity higher than 1 $\frac{\text{kg}}{\text{day.L}}$ and energy requirement lower than 5 MJ/kg of CO_2. (b) Effect of microscopic diffusivity in K_{eff} on process performance for crystallite size $R_\mu = 10$ μm. Arrows indicate changes undergone by structures for which the inclusion of molecular diffusivity has led to a significant drop in process performance, pushing them out of the EV region. (c) Percentages of PF and EV materials with respect to the starting set of "promising candidates" as selected by the active-learning model, assuming constant (dashed lines) or material-specific (solid lines) K_{eff}. The percentage of PF materials dropped from 81% (798 out of 982) to 80% (784 out of 982) upon inclusion of microscopic diffusivity. The prevalence of EV materials went from 28% (277 out of 982) to 24% (234 out of 982) of the starting set, or from 35% (277 out of 798) to 30% (234 out of 784) of their respective PF set. This corresponds to a 16% reduction (from 277 to 234) in the original EV population, representing the "false leads".

- Typographical errors need to be corrected.

We have fixed several typographical errors previously not flagged by our text editor software. Besides that, we standardized the US/UK spelling of some words to the US standard.

While the study takes an interesting first step in emphasizing diffusion, the subsequent analysis requires substantial improvements before it can fully support the claims made in the title.

We hope that the reviewer considers the current revised version as substantially improved and meritorious of publication in *Nature Communications*.

Reviewer #3

This manuscript describes molecular simulations and process-scale simulations of carbon dioxide capture performance of MOFs. The work is useful since the CO_2 separation performances of large number of materials has been computed. However, in its current form, I do not think that the work can be published in highly prestigious Nature journal because the experimental validation -perhaps the most critical part- is missing. Without the experimental synthesis and testing of the MOF, I believe the results do not have the value to be published in Nature. My other comments related to simulation techniques and results are as follows:

We would like to thank the reviewer for the time and effort dedicated to evaluating our manuscript. We agree that *Nature Communications* is indeed a prestigious and impactful journal within the *Nature* journal family. However, we respectfully disagree with the notion that the absence of experimental validation should preclude our findings from being considered a valuable contribution to the journal's readership. Over the years, several important computational studies (such as [https://doi.org/10.1038/ncomms2697, https://doi.org/10.1038/ncomms15396, https://doi.org/10.1038/ncomms13945, https://doi.org/10.1038/s41467-020-17755-8, https://doi.org/10.1038/s41467-022-30994-1, https://doi.org/10.1038/s41467-025-58499-7, https://doi.org/10.1038/s41467-025-58503-0] just to cite a few) were published here. We have chosen to publish our findings in *Nature Communications* because, as a leading open-access journal, it supports the code and data releases in open-science repositories such as Zenodo [https://doi.org/10.5281/zenodo.13948293]. This allows for validation and reuse of our results by the scientific community.

Our results demonstrate that the commonly used assumption — that diffusion in micropores is negligible when assessing the performance of porous materials at the process level — is not universally valid. This approximation is frequently employed in the evaluation of materials for CO_2 capture, based on the argument that the diffusion path length within micropores is orders of magnitude smaller than that in macroporous or interstitial regions. Consequently, for a typical diffusion coefficient of the order of 10^{-6} m^2/s, mass transport through the macroscopic regions is presumed to dominate. This has been widely accepted based on the results for the diffusion into large pore zeolites [https://doi.org/10.1007/s10450-013-9554-z, https://doi.org/10.1007/s10450-013-9545-0, https://doi.org/10.1016/j.ces.2005.03.024].

However, our findings show that microporous materials exhibit a wide distribution of diffusion coefficients, deviating by several orders of magnitude from the standard value often used to justify this simplification. Notably, this is supported not only by our simulation results but also by the experimental diffusion data available in the literature, which reveal significant discrepancies from the typical values employed in this approximation, as illustrated in Table S8.

For a significant number of materials — particularly those with narrow micropores, where pronounced confinement effects lead to high CO_2 affinity — the diffusion coefficient can be considerably low, as exemplified by CID-Me. For such materials, the traditional approximation is no longer valid, and neglecting diffusion within micropores results in computational process-level performance assessments that do not reflect the material's real-world behavior, as demonstrated by our findings.

As the urgency of generating high-performance CO_2 capture materials increases, computational modeling has gained momentum, both in academia and in industry. Especially when evaluating thousands of potential materials leads across different scales — from the atomistic to the industrial process level — computational screening is not only the most economically viable and fastest approach, but in many cases, the only feasible option. Therefore, we believe that our scientific results and methodological advancements indeed merit publication in *Nature Communications*.

To enrich the discussion on the calculation of diffusion coefficients from an experimental standpoint, we add the Table S8 to the Supplementary Information section *"S3.2.2 Validation against experiments"*, alongside the following text.

> ***"S3.2.2 Validation against experiments***
>
> *To validate our methodology for calculating the microscopic diffusion coefficient, we turn to the literature for experimental estimates. In Table S8, we see the experimentally determined CO_2 diffusion coefficient for several porous materials. The conditions under which the simulations were performed always mimic those in the experiments reported in the references. Our simulation results are generally of the same order of magnitude as the experimental benchmark, except for Zn_2(dobdc), for which our estimate is one order of magnitude higher. This overestimation might be due to an incomplete treatment of the Zn open-metal site using a generic Lennard-Jones force field."*

Material	Experimental (m^2/s)	Simulated (m^2/s)	Reference
HKUST-1	1.7×10^{-9}	5.2×10^{-9}	Tovar *et al.*[85]
ZIF-8	1.9×10^{-10}	1.72×10^{-10}	Jannatabadi *et al.*[86]
Zn_2(dobdc)	1.5×10^{-9}	2.0×10^{-8}	Forse *et al.*[87]
Zn_2(dobpdc)	5.9×10^{-9}	1.13×10^{-8}	Forse *et al.*[87]
CID-Me	$10^{-13} - 10^{-15}$	2.86×10^{-24}	Kurihara *et al.*[88]
D-MOF1	6.2×10^{-9}	5.83×10^{-9}	Peksa *et al.*[89]

Table S8. Experimental and simulated CO_2 diffusion coefficients reported in the literature.

1. The abstract starts by saying 'evaluating 10,143 MOFs for post-combustion carbon dioxide capture' but I believe many materials should have been

eliminated due to structural problems or pore size. Is this really the final number of materials that gone to simulations?

The material evaluation procedure consists of multiple steps, including consistency checks, machine-learning recommendations and Physics-based multi-scale simulations.

From the original 10,143 structures of the CoRE-MOF-2019 database, we have excluded 3,659 after porosity and sanity checks based on **`mofchecker`**. This information is provided in the first paragraph of the "*Results*" section.

> *"As reference database for our study, we have selected the non-disordered, all solvent removed (ASR) portion of CoRE-MOF-2019 (v1.1.2). From the initial dataset of 10,143 structures, we have removed 3,659 spurious MOF structures using **`mofchecker`** as well as an element-specific, charge-based heuristics."*

The remaining 6,484 candidates were then evaluated by the machine-learning recommendation model to select promising materials for the Physics-based simulation suite. The final number of materials that have undergone all stages of screening, including Physics-based simulations at the molecular and process levels, was 982. This information is provided in the second paragraph of the *"Active learning-based structure selection"* sub-section of the *"Results"*:

> *"Overall, 982 materials were subjected to GCMC simulations over 5 active learning iterations."*

We would like to point out that we implemented additional consistency checks, in addition to **`mofchecker`,** for charge-based elimination of MOFs in the CoRE-MOF-2019 database. We have included this additional step in the response to the last comment below.

2. For the charges, EQEQ method was used, did the authors validate the accuracy of this method compared to DDEC charges. Since CO_2 was studied, using a highly accurate partial charge assignment method must be priority, and since DDEC charges are available for most MOFs, I do not why the authors preferred an approximate method.

We acknowledge that databases such as ARC-MOF (Jan/2023) [https://doi.org/10.1021/acs.chemmater.2c02485], CoRE-MOF-2024 (Dec/2024) [https://doi.org/10.1016/j.matt.2025.102140] and MOSAEC-DB (Jan/2025) [https://doi.org/10.1039/D4SC07438F] provide high-quality partial charges derived from DFT electronic density. However, these databases have only been made available recently, or, in the case of MOSAEC-DB, are still in the process of being published.

Note, that our own manuscript preprint (Nov/2024) [https://doi.org/10.26434/chemrxiv-2024-3bh17] predates most of these works.

Importantly, at the time of planning and initiating our work, between 2021 and 2022, the only database containing DDEC partial charges was a subset of CoRE-MOF-2014 [https://doi.org/10.1021/acs.chemmater.5b03836, https://doi.org/10.1021/cm502594j], which included just 2,932 structures, which was not sufficient for the development of this work. While machine-learning models trained on this database have been proposed to predict atomic partial charges, such as PACMOF [https://doi.org/10.1021/acs.jctc.0c01229] and MPNN [https://doi.org/10.1021/acs.jpcc.0c04903], we have considered that incorporating such models would introduce an additional layer of uncertainty and complexity to our analysis, diverting the focus from our core objective — i.e., evaluating the impact of micropore diffusion in MOF on multiscale performance assessments.

Calculating DDEC (or equivalent) charges for each structure in our dataset would have required computational resources and execution times inconsistent within the timeframe planned for this study. Therefore, we decided to use EQeq charges, which, although approximate and subject to certain limitations, offer a favorable cost-benefit ratio [https://doi.org/10.1002/slct.201701934, https://doi.org/10.1038/s41597-023-02116-z]. Furthermore, the widespread use of EQeq in high-throughput studies in the literature [https://doi.org/10.1021/acs.langmuir.6b02803, https://doi.org/10.1039/C4CS00070F, https://doi.org/10.26434/chemrxiv-2025-mz223] also supported our choice, as it facilitates direct comparison with published results. For clarifying our choice of an approximate partial charge assignment method such as EQeq, we have added the following text to the end of the first paragraph of the *"Molecular-level simulation"* subsection of the "Methods" section.

> *"Despite the emergence of structure databases with DFT-derived partial atomic charges [https://doi.org/10.1021/acs.chemmater.2c02485, https://doi.org/10.1016/j.matt.2025.102140, https://doi.org/10.1039/D4SC07438F], we have chosen EQeq charges* which, although approximate and subject to certain limitations, offer certain benefits*."*

3. A recent work by Woos group published in Chemical Science showed that the Core MOF database has a lot of incorrect structures, did the authors check their MOFs and compare with the ones reported as corrected structures in that work?

Despite the immense value added by the creation of standardized structure databases, they do not come without issues. Perfectly cleaning and fixing all entries in databases containing tens of thousands of structures is not a simple task and can only be achieved

iteratively [https://doi.org/10.1039/D0RA02498H]. We are aware of the need to filter out incorrect structures at the earliest stages of computational screening.

In this work, we have first used **`mofchecker`** to identify critical structural errors, such as missing metal atoms as a necessary condition for a structure to be classified as a MOF, overlapping atoms, hyper-coordinated atoms, e.g., carbon with five bonds or hydrogen with more than one bond, missing hydrogen atoms, among others. We have also implemented additional checks in addition to **`mofchecker`** for charge-based elimination of MOFs in the CoRE-MOF-2019 database. With our enhanced structure elimination criteria, we have flagged 3,659 structures in the CoRE-MOF-2019 database, while **`mofchecker`** originally eliminated only 3,246 structures. We provide the breakdown of the elimination criteria in Table S1, alongside detailed explanation on the charge-based filter in the Supplementary Information.

Table S1. Percentage of CoRE-MOF-2019 structures flagged by each individual check.

#	Elimination criteria	% structures flagged
1	`has_carbon`	5.3%
2	`has_hydrogen`	10.8%
3	`has_atomic_overlaps`	0.6%
4	`has_overcoordinated_c`	0.6%
5	`has_overcoordinated_n`	0.1%
6	`has_overcoordinated_h`	0.5%
7	`has_undercoordinated_c`	10.4%
8	`has_undercoordinated_n`	7.3%
9	`has_undercoordinated_rare_earth`	0.8%
10	`has_metal`	0.0%
11	`has_lone_molecule`	11.1%
12	`has_high_charges`	1.4%
13	`is_porous`	0.1%
14	`has_suspicicious_terminal_oxo`	0.6%
15	`outside_qmof_min_max`	9.9%
	`mofchecker` only	32.0%
	`mofchecker` and `outside_qmof_min_max`	36.1%

Out of the 10,143 structures analyzed, 3,659 presented at least one of these issues and were excluded from further analysis. This corresponds to approximately 36% of the structures, a value that is relatively close to the 46% reported by Woo and collaborators [https://doi.org/10.1039/D4SC07438F]. But it is important to note that their heuristic for identifying chemically incorrect structures differs from the one implemented in

`mofchecker`, which may explain the difference in the reported percentages. Unfortunately, since neither the code used by Woo *et al.* nor the list of problematic structures is publicly available, we are currently unable to quantify the overlap between the two sets of flagged structures. However, based on our careful inspection of the structures flagged as incorrect by **`mofchecker`**, it is very likely that nearly all of them would also fall within the set identified as chemically invalid by Woo et al. Therefore, we are confident that the structures selected for our study are chemically sound, and the results derived from them are valid.

In the following, we detail how the charge-based filter was implemented. By default, **`mofchecker`** flags the structures with EQeq charges outside the normal range from –3 to +3. But we find that a fixed range is not sufficient due to different elements having different oxidation states and different charge ranges. In order to collect suitable, element-specific charge ranges, we have used the structures in the QMOF database [https://doi.org/10.1016/j.matt.2021.02.015] for calculating the charge distribution for each element in the periodic table. We find that EQeq charges computed for the QMOF structures are well behaved and show narrower distributions than structures in CoRE-MOF-2019, likely because the structures are DFT optimized. An example partial charge distribution for Barium in CoRE-MOF-2019 and QMOF databases is shown in Figure S1. We have flagged as "spurious" all CoRE-MOF-2019 structures containing atoms whose partial charge falls outside the 1st to 99th percentile range for this specific element. Therefore, in our example, we have excluded all CoRE-MOF-2019 structures containing Ba atoms with partial charge below 1.44 or above 2.33.

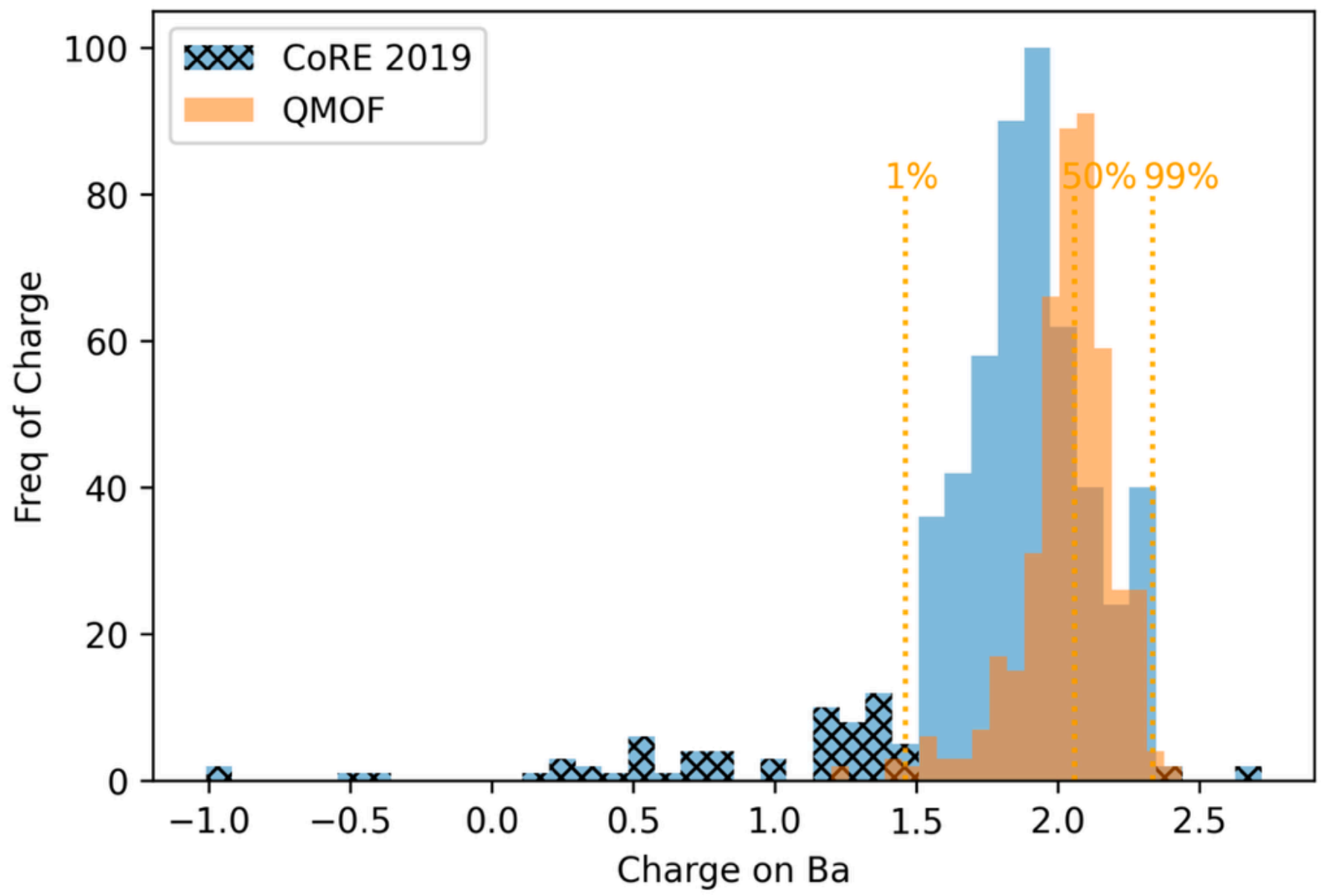

Figure S1. **Ba charge histogram** Partial charge distribution for Barium in the QMOF (orange) and CoRE-MOF-2019 (blue) databases, overlaid to the 1st, 50th and 99th percentiles of the QMOF distribution. The hashed regions show the excluded MOF structures with unreasonable Ba charges.

We have expanded upon the description of the spurious structure removal step in the main manuscript, in first paragraph of the *"Results",*

> *"From the initial dataset of 10,143 structures, we have removed 3,659 spurious MOF structures using* ***mofchecker*** *and an element-specific, charge-based heuristics."*

in the "Methods" section,

> ***Spurious structure removal***
>
> *"We have used* ***mofchecker*** *to eliminate 3,246 CoRE-MOF-2019 structures from our data set based on a suite of composition, coordination and consistency checks. An additional set of 413 structures were eliminated by an element-specific, charge-based heuristics. The heuristic aims at the removal of structures whose atoms present partial charges at the top, or bottom, 1% of a particular element charge distribution. The breakdown of elimination criteria by percentage and acceptable charge ranges per element are provided in the Supplementary Information."*

and Supplementary Information

> ***Removal of spurious MOF structures***

*Besides using **`mofchecker`** to eliminate 3,246 out of 10,142 structures from the CoRE-MOF-2019 database due to a combination of composition, coordination and consistency checks, we have also implemented our own charge-based filtering criteria. We found that the charge-based filter from **`mofchecker`**, which flags atoms with EQeq charges outside of the [−3, +3] range, was not sufficient to eliminate all spurious structures from the CoRE-MOF-2019 database, mainly due to different elements having different oxidation states and different charge ranges. We have, therefore, collected a set of acceptable charge ranges by considering the 1st and 99th percentiles of the element-specific charge distributions in the QMOF database. The Application of this additional filter has removed another 413 structures from the data set, leaving only 6,484 candidate structures for further evaluation. The breakdown of elimination criteria and their respective percentages is provided in Table S1.*

We find that EQeq charges computed for the QMOF structures are well behaved and show narrower distributions than for CoRE-MOF-2019, possibly because the structures were DFT-optimized. As an example, we provide in Figure S1 the charge distributions for Barium (Ba) in the QMOF and CoRE-MOF-2019 databases. In this example, the heuristics led us to exclude all CoRE-MOF-2019 structures containing Ba atoms with partial charges below 1.44 or above 2.33, as these are considered outliers. In Table S2 and Table S3, we provide the charge distribution metrics for all elements individually.

Table S2. Element-specific EQeq charge distribution in the QMOF database[58], from Hydrogen (H) to Ruthenium (Ru). We eliminated MOF structures with charges outside of the range from the 1st to the 99th percentile.

Element	Minimum	1st Percentile	50th Percentile	99th Percentile	Maximum
H	-0.49	-0.03	0.05	0.24	0.66
Li	0.53	0.53	0.56	0.58	0.59
Be	1.47	1.47	1.48	1.48	1.48
B	0.02	0.04	1.21	3.18	3.36
C	-1.25	-0.28	0.00	0.50	1.05
N	-0.60	-0.31	-0.19	0.05	0.19
O	-2.02	-1.07	-0.42	-0.08	0.17
F	-1.44	-0.90	-0.42	-0.27	-0.07
Na	0.52	0.56	0.59	0.60	0.61
Mg	1.41	1.43	1.47	1.48	1.49
Al	2.26	2.36	2.39	2.40	2.42
Si	0.51	0.51	0.91	2.98	2.98
P	-0.69	-0.09	0.41	0.64	1.02
S	-1.13	-0.60	0.02	0.73	1.21
Cl	-1.01	-0.76	-0.35	-0.14	0.02
K	0.43	0.58	0.66	0.71	0.77
Ca	1.45	1.46	1.51	1.53	1.55
Sc	2.28	2.28	2.34	2.35	2.35
Ti	3.05	3.06	3.11	3.14	3.15
V	1.58	1.66	1.82	1.86	1.87
Cr	1.10	1.10	1.52	1.65	1.65
Mn	0.99	1.15	1.29	1.35	1.45
Fe	0.81	0.97	1.16	1.29	1.33
Co	0.92	1.01	1.16	1.23	1.26
Ni	0.12	0.94	1.10	1.15	1.18
Cu	0.60	0.70	0.86	0.95	1.01
Zn	0.79	1.11	1.20	1.24	1.29
Ga	1.85	1.85	2.05	2.12	2.15
Ge	1.05	1.05	1.35	2.00	2.14
As	0.08	0.08	0.41	0.53	0.53
Se	-0.96	-0.92	-0.07	0.66	0.67
Br	-1.00	-0.71	-0.23	-0.07	0.08
Rb	0.58	0.62	0.69	0.72	0.76
Sr	1.44	1.46	1.53	1.54	1.55
Y	2.31	2.36	2.39	2.40	2.40
Zr	3.03	3.15	3.21	3.22	3.23
Nb	1.27	1.27	1.64	1.74	1.74
Mo	0.93	0.96	1.21	1.28	1.34
Tc	1.14	1.14	1.16	1.17	1.17
Ru	0.79	0.81	0.93	1.01	1.01

Table S3. Element-specific EQeq charge distribution in the QMOF database[58], from Rhodium (Rh) to Plutonium (Pu). We eliminated MOF structures with charges outside of the range from the 1st to the 99th percentile.

Element	Minimum	1st Percentile	50th Percentile	99th Percentile	Maximum
Rh	0.75	0.76	0.88	0.93	0.93
Pd	0.54	0.57	0.72	0.84	0.86
Ag	0.40	0.44	0.54	0.61	0.67
Cd	0.98	1.10	1.21	1.25	1.28
In	1.84	1.87	1.98	2.03	2.04
Sn	0.63	0.65	1.30	1.33	1.34
Sb	0.20	0.21	0.45	1.37	1.37
Te	-0.86	-0.85	-0.60	0.51	0.60
I	-1.02	-0.77	-0.38	0.04	0.20
Cs	0.64	0.65	0.71	0.79	0.79
Ba	1.20	1.46	2.06	2.34	2.44
La	1.44	1.48	1.70	1.77	1.88
Ce	2.10	2.10	2.16	2.20	2.20
Pr	1.87	1.98	2.08	2.10	2.11
Nd	1.43	1.67	2.37	2.61	2.71
Sm	0.87	1.44	2.20	2.41	2.54
Eu	1.82	1.85	2.09	2.23	2.23
Gd	0.97	1.23	1.71	1.86	1.95
Tb	1.12	1.46	2.05	2.18	2.20
Dy	1.06	1.40	1.99	2.13	2.32
Ho	1.22	1.36	1.95	2.08	2.34
Er	1.02	1.33	1.90	2.06	2.09
Tm	1.44	1.44	1.59	1.62	1.66
Yb	1.41	1.44	1.57	1.60	1.60
Lu	1.06	1.06	1.28	1.32	1.32
Hf	1.21	1.24	1.47	1.52	1.53
W	0.00	0.11	0.53	1.29	1.30
Re	0.66	0.66	0.76	1.02	1.02
Ir	0.21	0.21	0.32	1.17	1.17
Pt	0.03	0.11	0.31	0.48	0.48
Au	0.14	0.24	0.28	0.46	0.51
Hg	-0.08	-0.04	0.12	0.29	0.33
Tl	0.55	0.55	0.65	0.77	0.77
Pb	1.03	1.14	1.24	1.30	1.30
Bi	1.70	1.77	1.85	1.92	1.92
Th	0.03	0.03	0.04	0.06	0.06
U	-0.01	0.01	0.04	0.15	0.23
Np	0.02	0.02	0.04	0.06	0.06
Pu	0.02	0.02	0.04	0.04	0.04

Reviewer #3

I now read the rebuttal letter provided by the authors. I keep my original comment on the chemical problems of the MOFs studied in this work. The authors mentioned that they used mofchecker to eliminate 3,246 CoRE-MOF-2019 structures that have problems. However, this is not enough as I explained in my previous round of comments.

The authors thank the reviewer for the careful reading of the manuscript and for considering it again after the first revision.

1. Tom Woo's group developed a tool called MOSAEC to check the accuracy – chemical correctness – of the MOF structures and the results of MOSAEC tool does not always agree with the mofchecker. Saying in other words, structures labeled as 'correct' by mofchecker can still have problems such as wrong oxidation states as identified by MOSAEC tool. Therefore, I strongly suggest the authors to check their MOFs by both tools – please see the recent JACS article on this.

In the revised manuscript, we applied a MOSAEC-based filter, in addition to MOFChecker, to our results. This new filter removes materials flagged as "bad" by the MOSAEC tool [White *et al.*, 2025]. **Overall, we find that the impact of MOSAEC filtering on our results, including the rankings and conclusions provided, is small. Specifically,**

- None of the 4 representative structures (i.e., VASFON, BETGUE, MOCHIW and EVOMOR) that we used to showcase the typical behaviors that might arise upon the inclusion of molecular diffusivity were flagged as "bad" by MOSAEC. Therefore Figure 1, Figure S10, Figure S11, Figure S12 and Figure S13 remain unaltered.
- None of the 20 top-performing MOFs that we highlighted in Table S10 and Table S11 were flagged as "bad" by MOSAEC. Therefore, our conclusions with respect to the effect of molecular diffusivity in the process-level performance of carbon capture materials remain intact.
- The material named "AHUTIH" that we used as an example for plotting the CO_2, N_2 and H_2O isotherms in Figure S3 was flagged as "bad" by MOSAEC. We, therefore, replaced it by "APAZOI". This change does not have any major implications, given that the purpose of this figure was simply to illustrate the typical shapes and value ranges of the simulated isotherms.

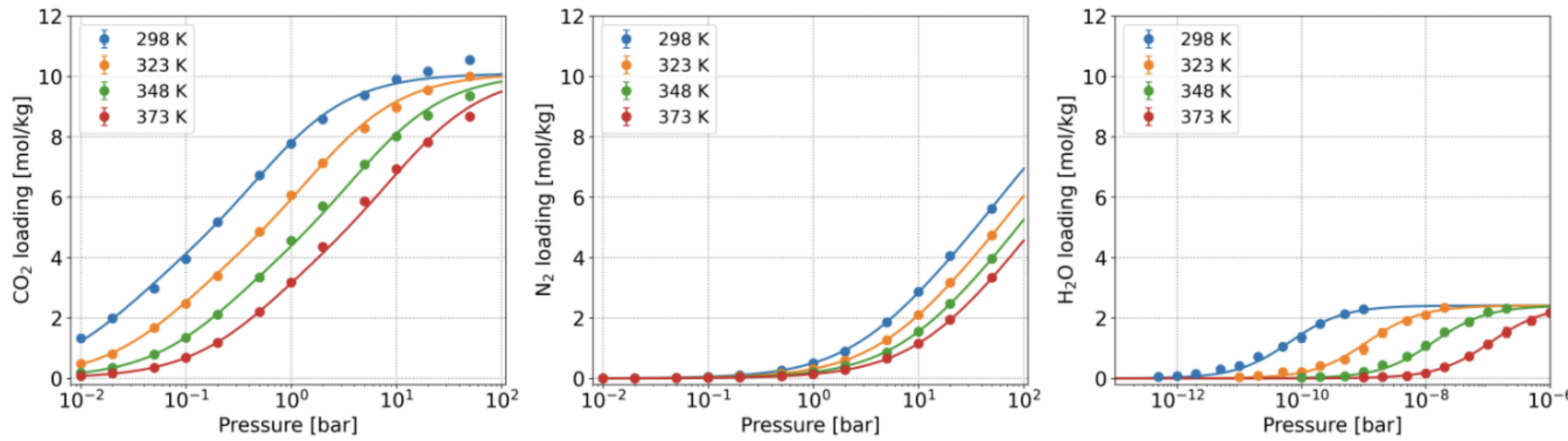

Figure S3. Simulated isotherms and adjusted Langmuir models. In this example for material APAZOI, symbols represent the simulated adsorption values along an isotherm and lines represent the best fit Langmuir isotherm model, at different temperatures, for CO_2 (left), N_2 (center) and H_2O (right).

- The distribution of materials in the Energy Requirement vs. Productivity panel, as depicted in Figure 4(a), remains qualitatively the same. 224 materials of little relevance were removed, but the overall distribution of points in the panel remains largely the same, leaving the overall conclusions unchanged. The same applies to Figure S14 and Figure S15, which are simply variants of Figure 4 with different values of crystallite radius R_μ.

Figure 4(a) – Including MOSAEC "bad" materials

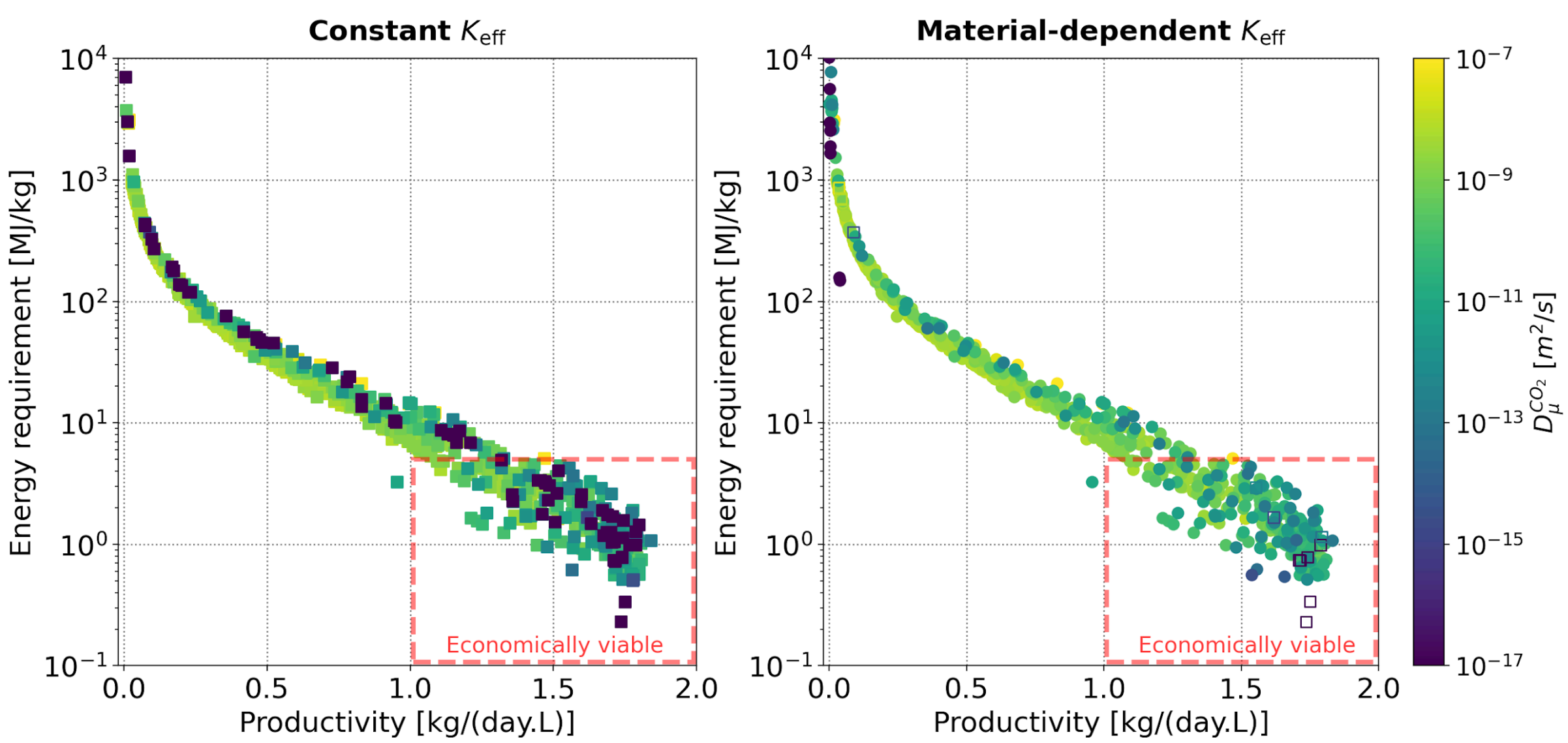

Figure 4(a) – Excluding MOSAEC “bad” materials

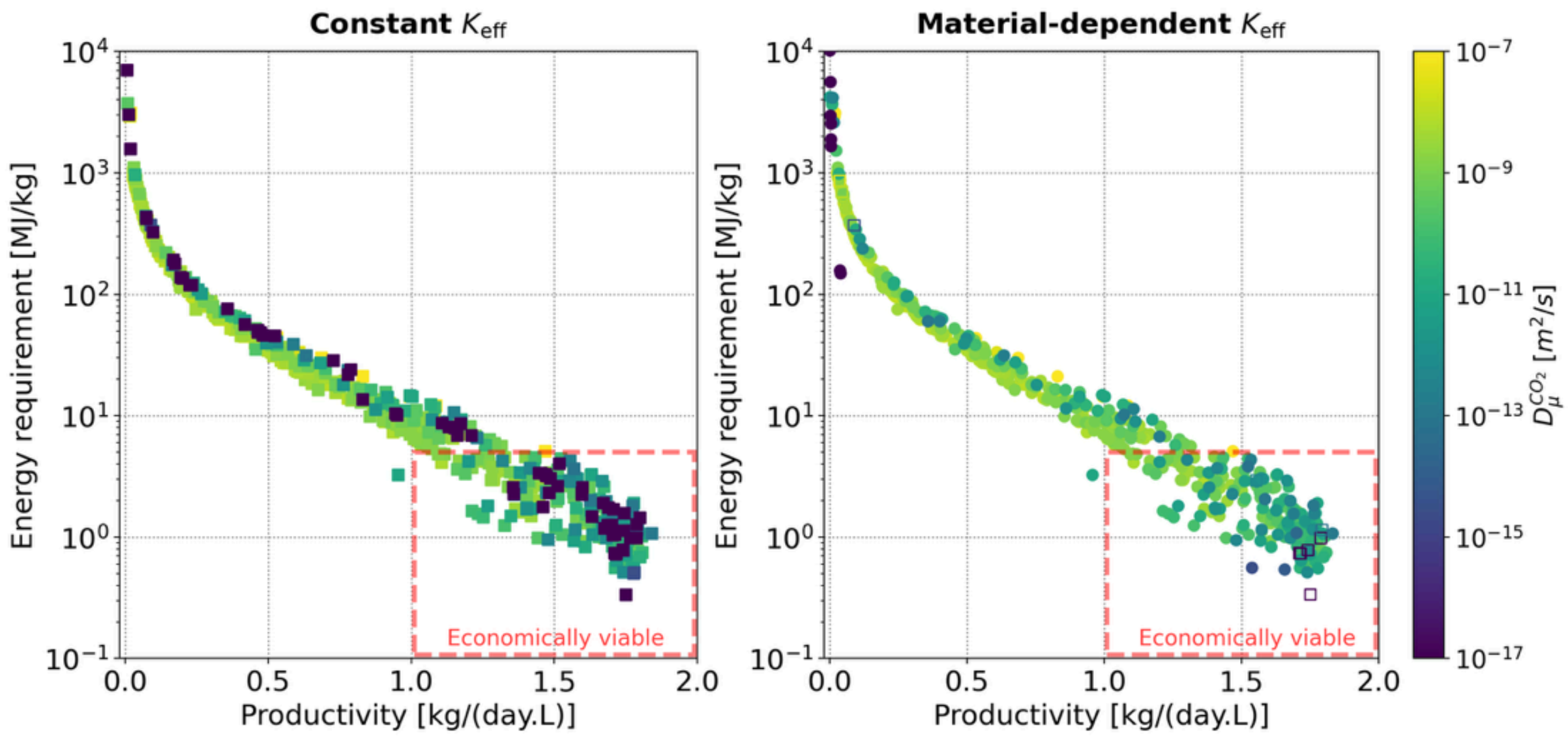

- The distribution of materials in the Productivity vs. R_μ panel, depicted in Figure 5, undergoes very small modifications. Overall, the data distribution in the plot remains largely unaltered, supporting essentially the same conclusions as before.

Figure 5 – Including MOSAEC “bad” materials

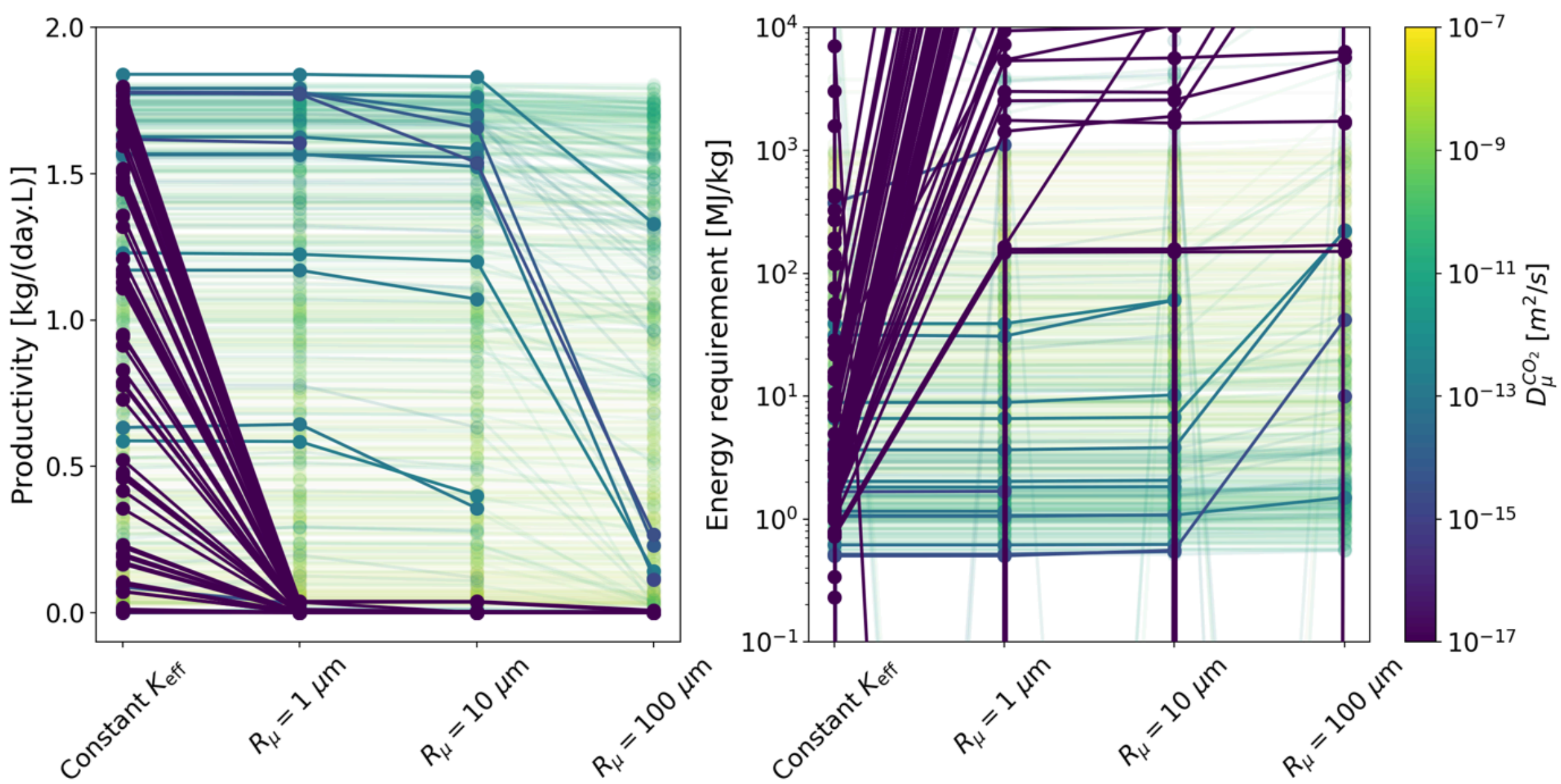

Figure 5 – Excluding MOSAEC “bad” materials

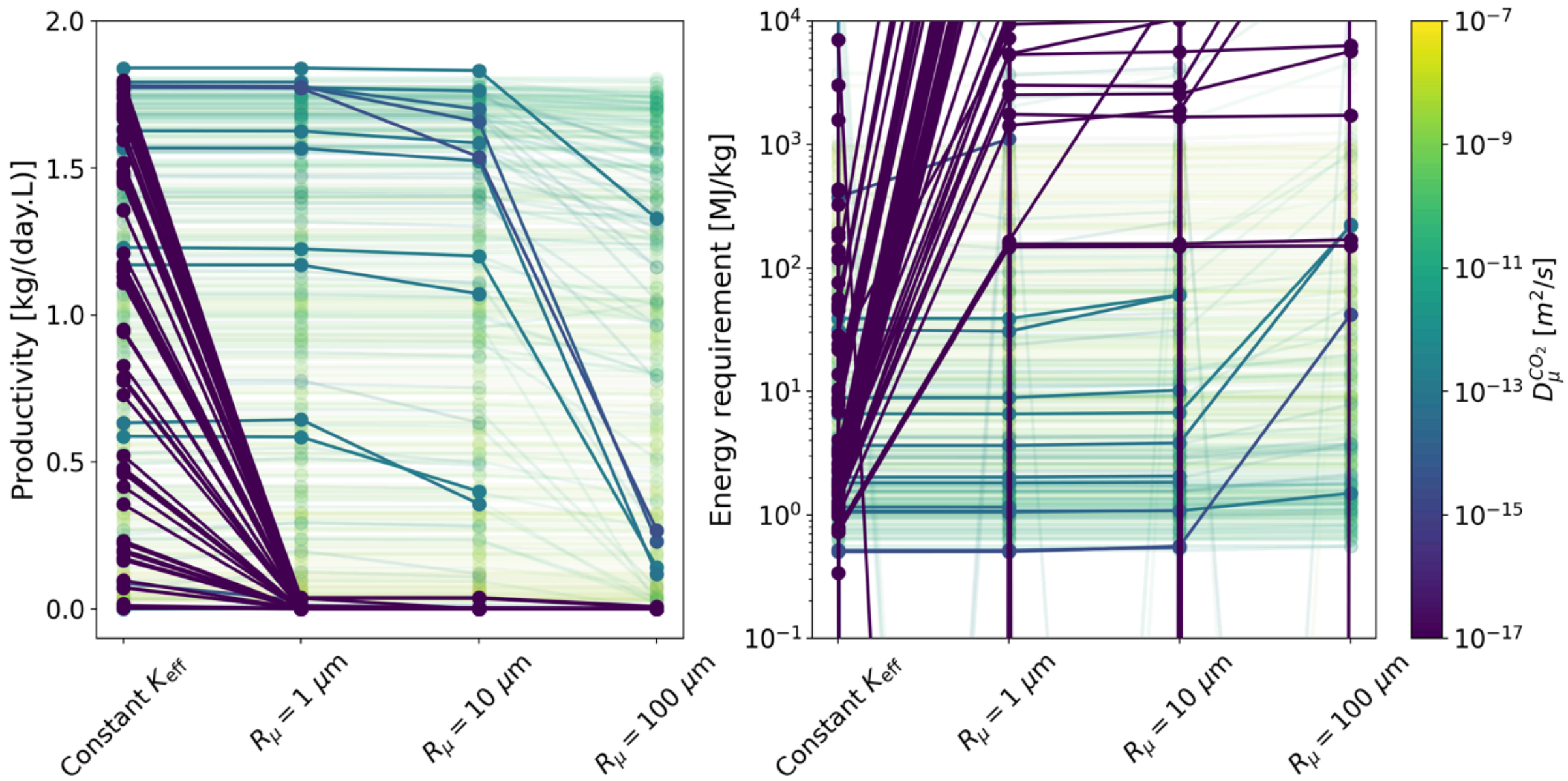

- 1 of the 4 previously identified water-tolerant, economically viable MOFs, i.e., EXOFIH, was flagged as “bad” by MOSAEC. The 4 MOFs are shown in Figure 6, Table S12 and Figure S16. Figure 6(a) appears largely unaltered, except for the noticeable removal of EXOFIH from the economically viable region.

Figure 6(a)

Including MOSAEC “bad” materials Excluding MOSAEC “bad” materials

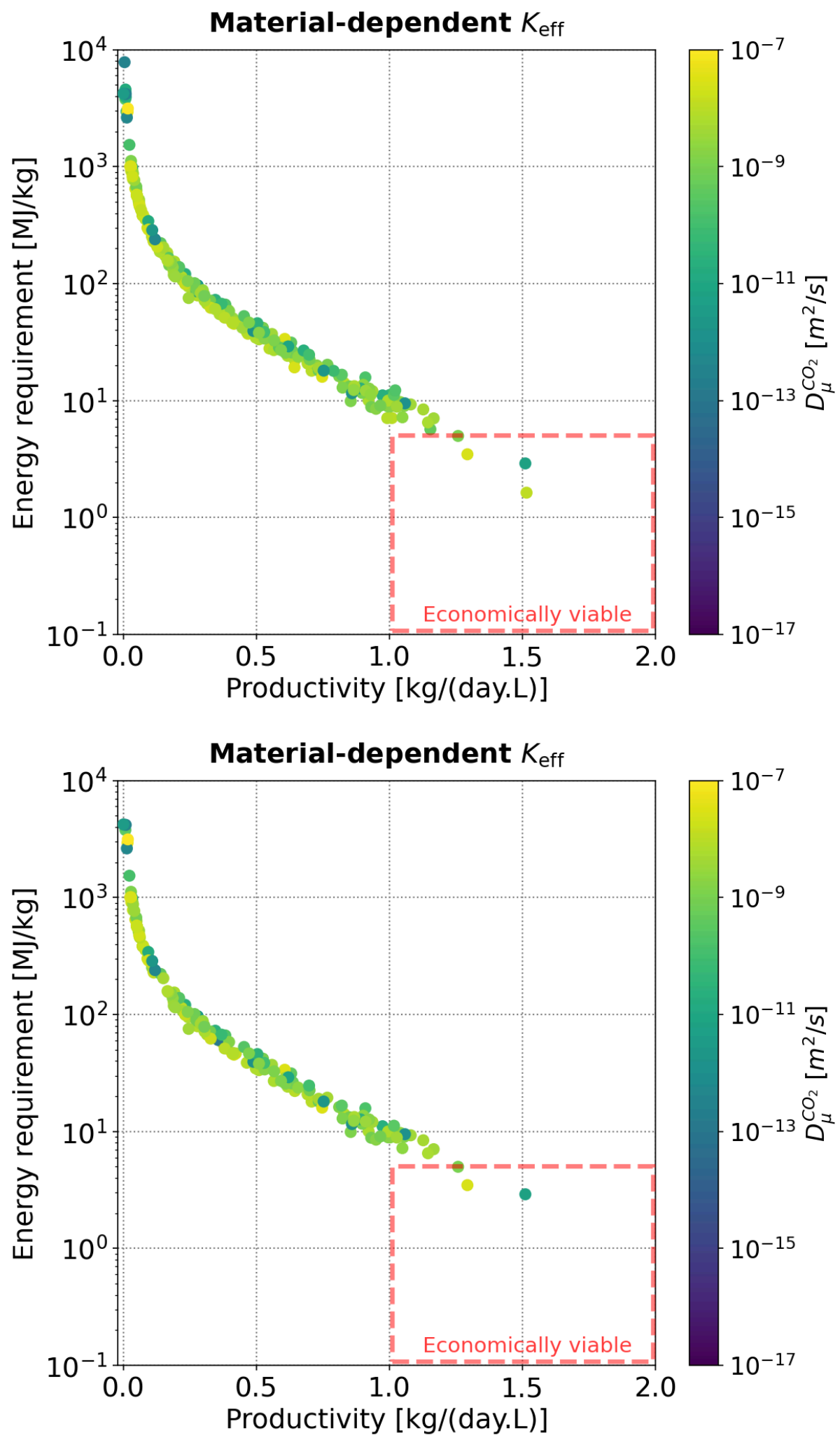

For a deeper understanding of why these structures were labeled as “bad” by MOSAEC and to assess the impact of the classification on our results, we conducted a case-by-case analysis of all 224 structures flagged as “bad”. To our surprise, a large number of these structures did not present any identifiable chemical issues. However, we noticed a recurring pattern: MOFs featuring open-metal sites (OMS) were systematically classified as “bad” by MOSAEC.

In the figure below, we show several well-known MOFs that contain OMS, such as Zn-MOF-74 (KOSLIP), with the same OMS as Mg-MOF-74. In addition, we show SNU-50 (ALAMUW) and PMOF-4 (YODWOF), both containing well-established metal-based secondary building units (SBUs) that are built from the M–paddlewheel motif. SNU-50 (ALAMUW), for instance, was reported to be stable after activation [Prasad *et al.*, 2010]. Similarly, MOFs based on well-known, stable SBUs such as Zr_6

cluster on NU-1100 (BOLZIN), as well as MOFs featuring Zn_4O clusters such as PUGSUH or UBULIO, were labeled as “bad” by MOSAEC.

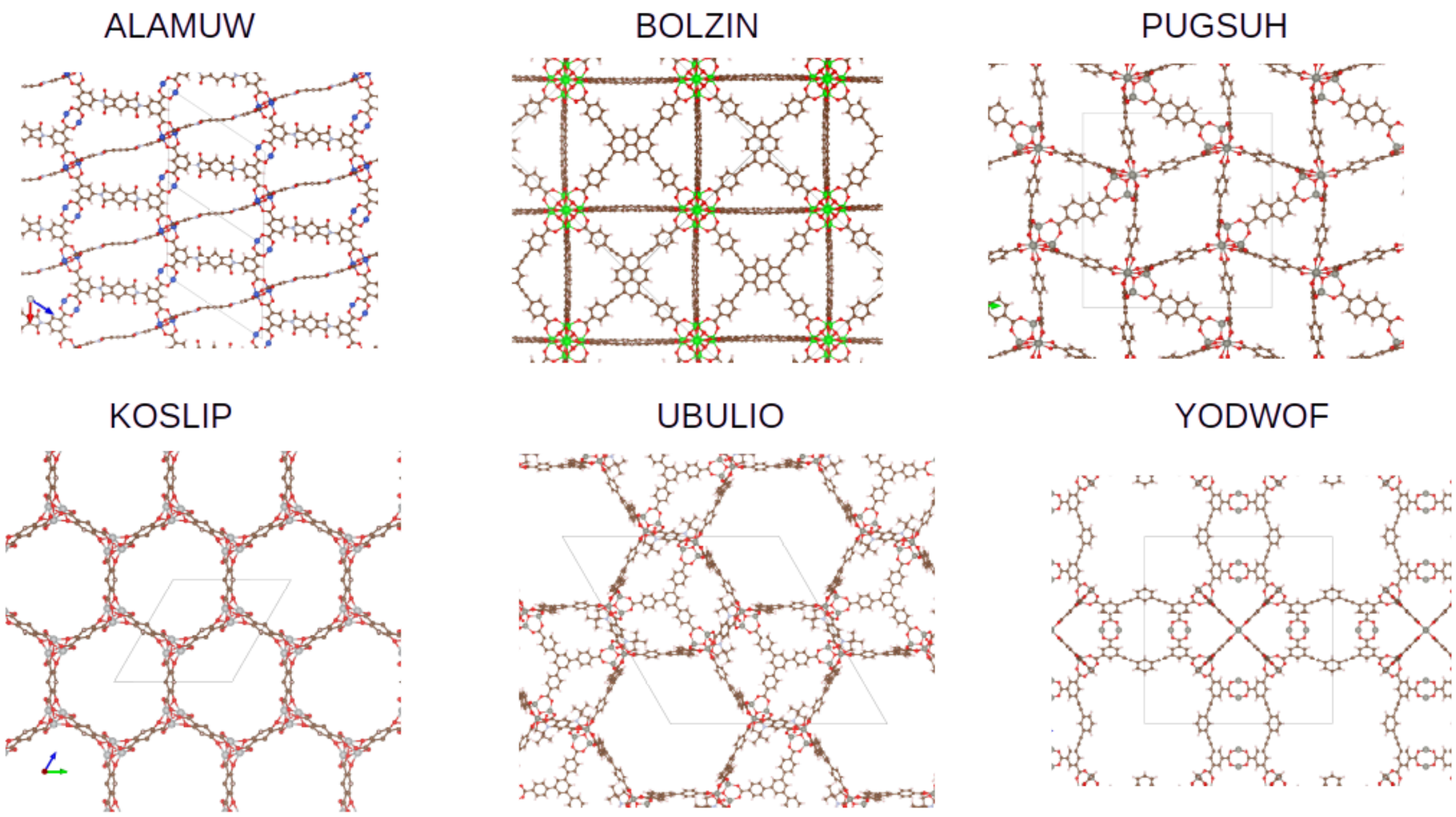

In view of these findings, we chose to limit the modifications in the main manuscript to Figure 6 and the surrounding text. We chose to clearly identify in the revised figure EXOFIH as a structure flagged as “bad” by MOSAEC, rather than to remove all flagged structures from the plots. In Figure 6, flagged structures are represented by empty circles, instead of filled circles, for comparison.

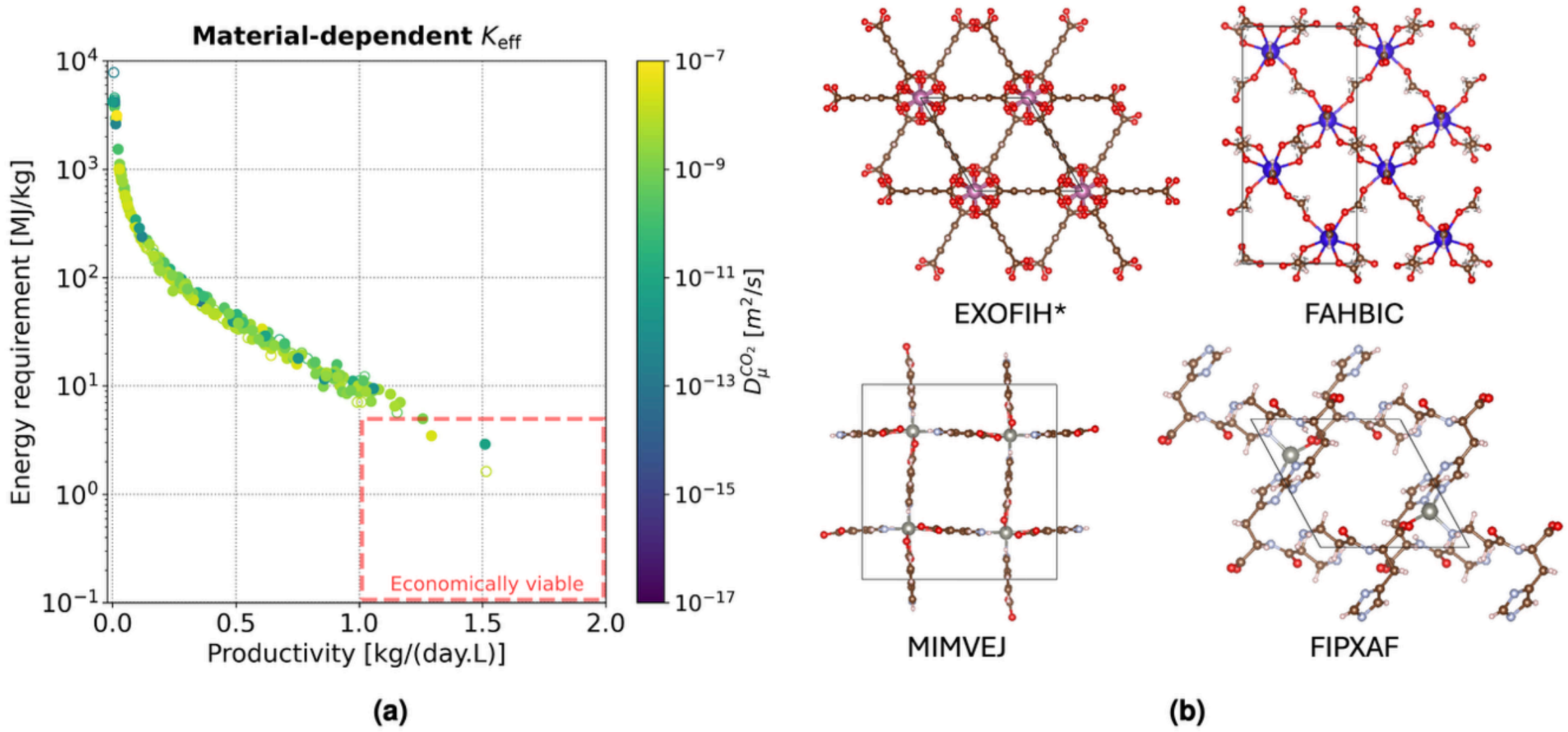

Figure 6. **Water-tolerant MOF candidates.** (a) Distribution of energy requirement *vs.* productivity for water-tolerant MOFs, i.e., with $\Delta G_{H_2O} > -8.1$ kJ/mol, assuming a crystallite diameter of $R_\mu = 10$ µm. We have identified 4 promising MOF structures in the economically viable region. Empty symbols represent structures flagged as "bad" by MOSAEC[37] (b) Crystal structures of the economically viable, water-tolerant MOF candidates. We marked EXOFIH to represent that it was flagged as "bad" by MOSAEC[37].

In the paragraph that precedes Figure 6, we have included the following passages

> *To further validate of our findings, we performed an additional, structural quality check based on metal oxidation states, by using MOSAEC [White et al., 2025].*
>
> *We note that one of the structures, namely EXOFIH, was flagged as "bad" by MOSAEC.*

In the Methods subsection entitled "Spurious structure removal", a paragraph was added to explain this additional layer of structure quality checks.

> *For the analysis of water-tolerant MOFs, see Figure 6, we applied additional, spurious structure removal criteria. White et al. proposed a pipeline that further refines the identification of spurious structures, leading to the removal of 224 structures that were flagged as "bad"" by MOSAEC [White et al., 2025].*

For completeness and consistency, we have applied the following changes to the Supplementary Information (SI), showing the effect of the MOSAEC filter on our results.

In Table S12, material EXOFIH was marked with * to call attention to the fact that it has been flagged as "bad" by the MOSAEC tool.

Table S12. Rank order and free energy of adsorption at 30 °C for CO_2, N_2 and H_2O of the top 4 water-tolerant MOFs (i.e., with $\Delta G_{H_2O} > -8.1$ kJ/mol), and CALF-20 (for comparison). All ΔG values calculated using the Widom particle insertion method. We marked EXOFIH to represent that it was flagged as "bad" by MOSAEC[37].

MOF	K_{eff} = const.	R_μ =1 µm	R_μ =10 µm	R_μ =100 µm	ΔG_{CO_2}, kJ/mol	ΔG_{N_2}, kJ/mol	ΔG_{H_2O}, kJ/mol
EXOFIH*	173	147	145	118	-10.5	-0.4	-8.0
FAHBIC	177	150	148	150	-7.8	5.9	-5.1
MIMVEJ	246	208	206	187	-10.3	-1.3	1.6
FIPXAF	260	222	220	200	-9.0	0.8	-5.6
CALF-20					-9.3	2.2	-8.1

In Figure S16, the material EXOFIH was marked with * to call attention to the fact that it has been flagged as "bad" by the MOSAEC tool.

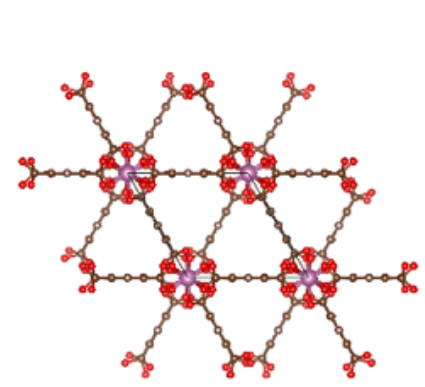

(a) EXOFIH*

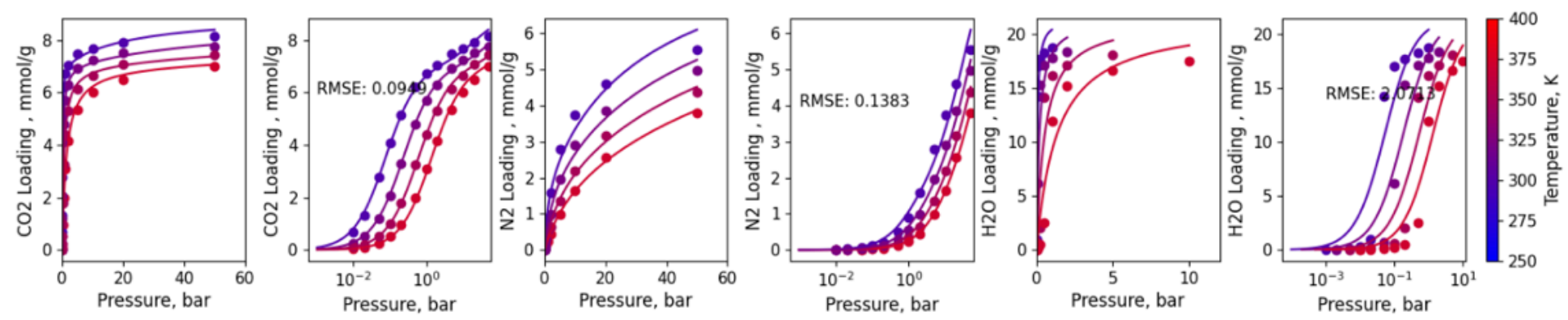

(b) CO_2, N_2 and H_2O adsorption isotherms for EXOFIH*.

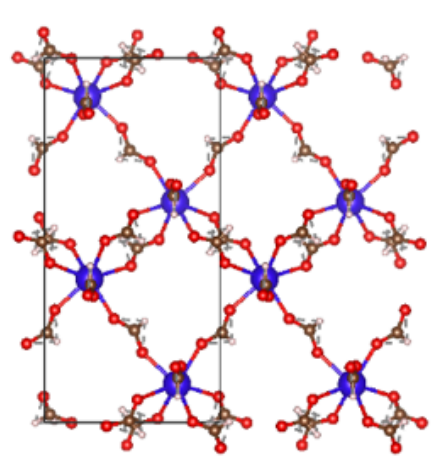

(c) FAHBIC

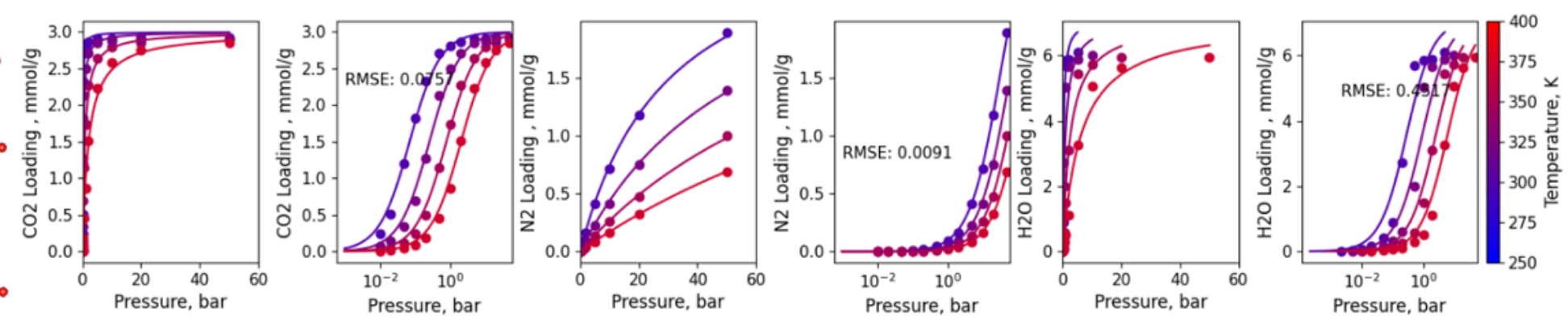

(d) CO_2, N_2 and H_2O adsorption isotherms for FAHBIC.

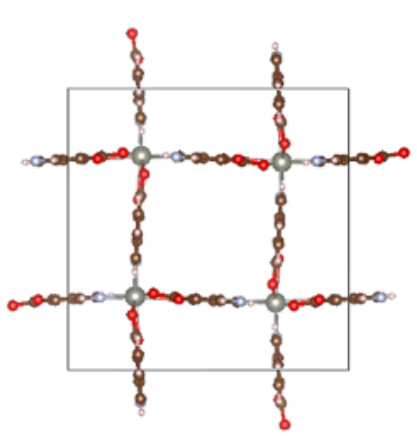

(e) MIMVEJ

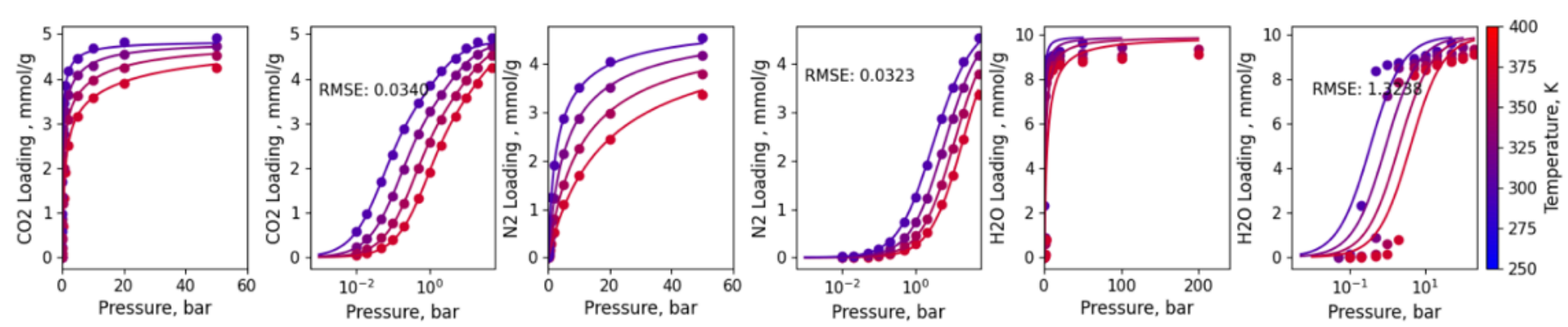

(f) CO_2, N_2 and H_2O adsorption isotherms for MIMVEJ.

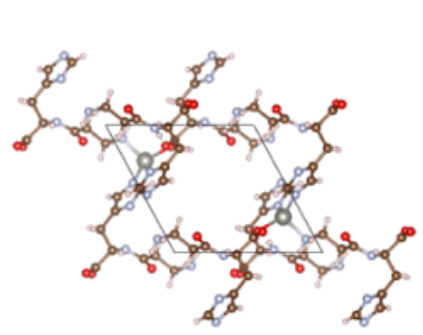

(g) FIPXAF

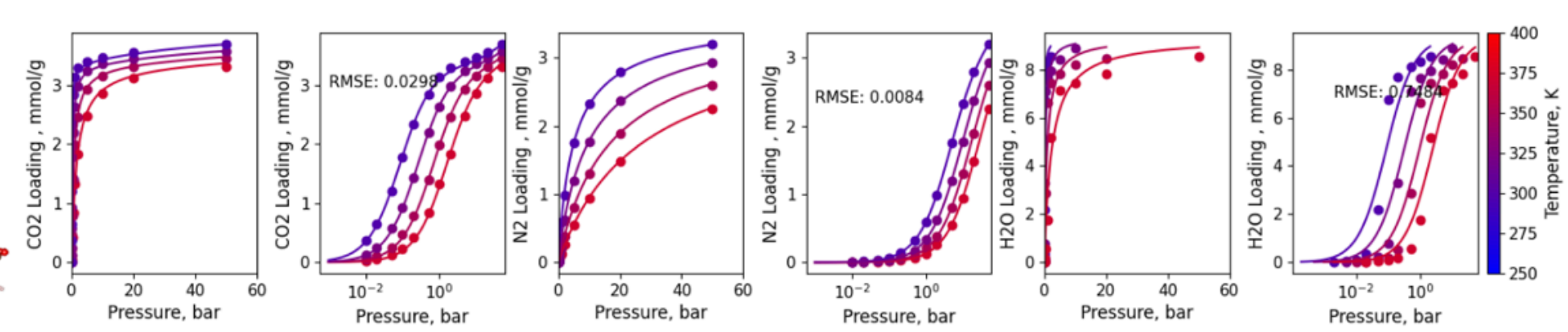

(h) CO_2, N_2 and H_2O adsorption isotherms for FIPXAF.

Figure S16. **Water-tolerant MOF structures and isotherms** (a) EXOFIH*, (c) FAHBIC, (e) MIMVEJ and (g) FIPXAF structures and their associated adsorption isotherms (b), (d), (f) and (h), respectively. CO_2, N_2 and H_2O isotherm data (filled circles) and fitted models (lines). We marked EXOFIH to represent that it was flagged as "bad" by MOSAEC[37].

We have also included in the SI the variants of Figure 4(a) and Figure 5 in which structures flagged as “bad” by MOSAEC were removed.

> *We also performed an additional, structural quality check based on metal oxidation states, as implemented by MOSAEC. The overall effect of removing structures flagged as “bad” by MOSAEC was insignificant, except in the case of water-tolerant MOFs, in which one of the four structures identified in the economically viable region was labeled as such, see Figure 6. To demonstrate the effect on Figure 4a and Figure 5, we removed for comparison in Figure S2 structures flagged as "bad" by MOSAEC. Overall, both plots appear very similar. A few scattered data points were removed, but the overall distributions, ranges, and trends remain the same.*

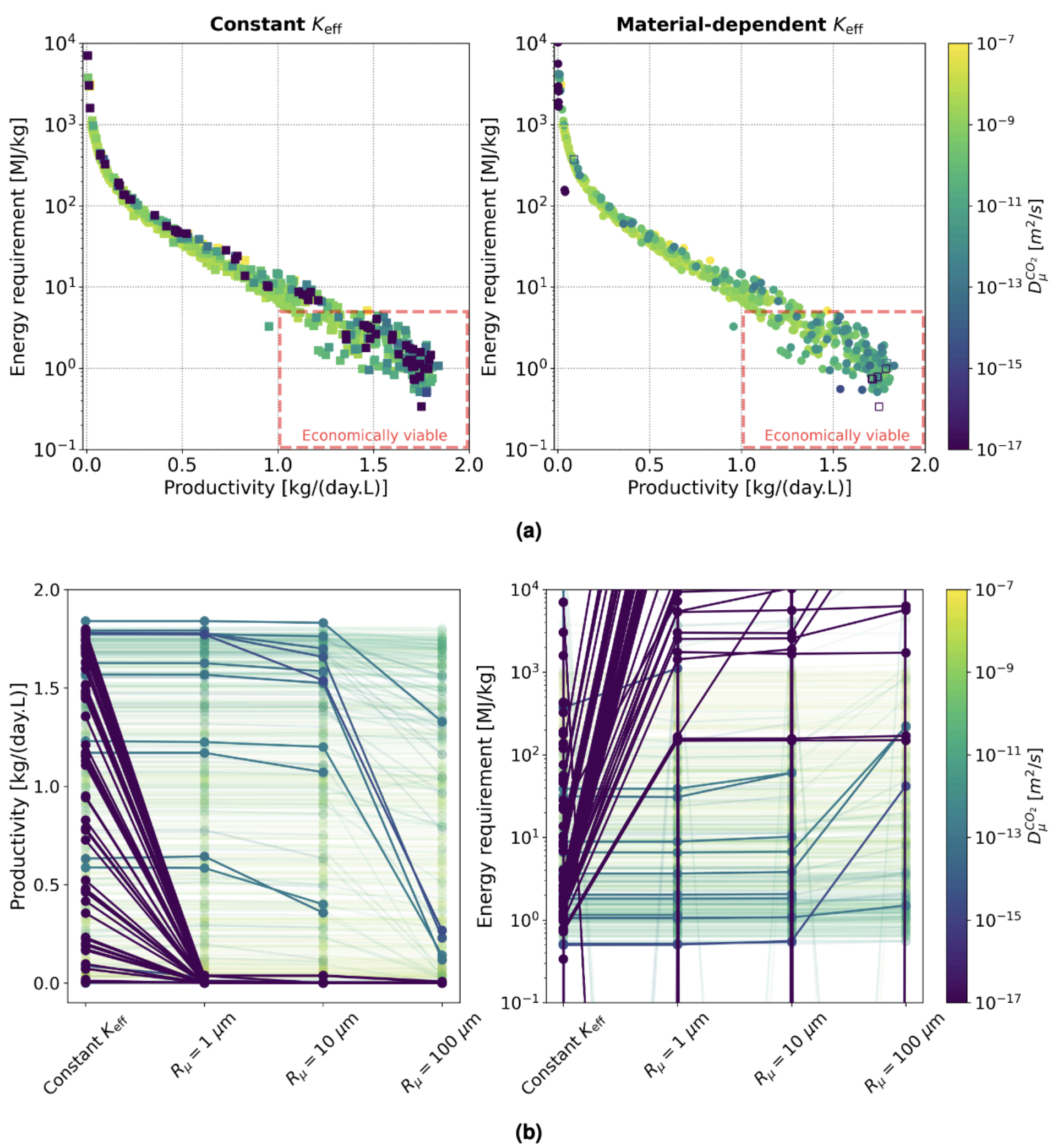

Figure S2. Effect of removing structures flagged as “bad” by MOSAEC. (a) Distribution of energy requirement *vs.* productivity for a crystallite diameter $R_\mu = 10$ μm, assuming a constant K_{eff} (left) and a material-dependent K_{eff} (right) that accounts for microscopic diffusivity. (b) Effect of molecular diffusion on CO_2 capture performance ranking. Productivity (left) and energy requirement (right) based on constant K_{eff}, as well as structure-dependent K_{eff} for representative crystallite sizes $R_\mu = 1,\ 10$ and 100 μm.

2. Another development is that the new CoRE-MOF-2025 database just came out a few weeks ago – so it is very important for the authors to check with this database, because many MOFs seem to be included in CoRE-MOF-2019 are now labeled as incorrect – not computation ready. I believe without proving the structures' chemical correctness, identifying best performing materials for a target application is highly misleading.

In the latest version of the CoRE-MOF-2025 dataset available on Zenodo [Guobin *et al.*, 2025], the authors provided a list of structures that did not pass their structural quality checks. There are three independent checks: mofchecker, Chen & Manz, and MOSAEC. **Overall, we find that the impact of MOSAEC filtering on our results, including the rankings and conclusions provided, is small. Specifically,**

- None of the four representative structures (i.e., VASFON, BETGUE, MOCHIW and EVOMOR) that we included to discuss the effect of molecular diffusivity inclusion were classified as "Not Computational-Ready", or "NCR", by CoRE-MOF-2025. Therefore, Figure 1, Figure S10, Figure S11, Figure S12 and Figure S13 remain unchanged.
- None of the 20 Top-performing MOFs in Table S10 and Table S11 were classified as "Not Computational-Ready", or "NCR", by CoRE-MOF-2025. Therefore, our conclusions with regard to the effect of molecular diffusivity in the process-level performance of carbon capture materials remain intact.
- None of the water-tolerant, economically viable MOF were flagged as "Not Computational-Ready", or "NCR", by CoRE-MOF-2025. We note that EXOFIH was flagged by MOSAEC and identified as such. The MOFs are shown in Figure 6, Table S12 and Figure S16.

We would like to point out that the negligible effect of removing "Not Computational-Ready", or "NCR", structures from CoRE-MOF-2025 is not entirely unexpected. It can be understood as a consequence of two factors:

- MOFChecker was already applied to the original dataset discussed in the manuscript. Therefore, MOFChecker was responsible for most of the exclusions that would overlap to a large extent with the method reported by [Chen & Manz, 2020].
- MOSAEC was applied for revising the dataset, as discussed above, so we would not expect to find any large amounts of spurious structures by applying additional filters.

We, therefore, limit the changes in the revised version of the manuscript to the changes described above, see discussion of MOSAEC filtering above.

Reviewer #4

The manuscript reports the inclusion of molecular diffusion for process-scale simulations of CO_2 capture in MOFs. It can be seen that a great deal of works have been done. Screening stable new MOFs with excellent carbon capture performance at process-scale simulation is important.

The authors thank the reviewer for the careful reading of the manuscript and for recognizing the importance and volume of the work.

However, there are some major concerns for the publication of manuscript.

1. The validation of methodology for calculating the diffusion coefficients is not sufficient. In section S3.2.2, to validate the methodology, the authors list the comparison of experimental and simulated diffusion coefficients of 6 MOFs in Table S8. However, the sample numbers are quite small comparing with the large numbers of estimated MOFs. How the situations for other MOF materials? Moreover, there is huge difference between the experimental and simulated results for CID-Me.

We share the reviewer's concern with the proper validation of the $D_{\mu}^{CO_2}$ simulation methodology, especially given that it is a new (and automated) protocol presented here for the first time. In fact, we went to great lengths for procuring experimental references to compare with. However, we found there is very little data published with regard to the experimental determination of the CO_2 diffusion coefficient. However, we have included all the references we could find into Table S8.

The goal of Table S8, rather than to display perfect agreement between simulation and experiments, is to analyze the strengths and limitations of the proposed simulation protocol. In our simulations, we do not consider framework flexibility, nor chemisorption, given the nature of the interatomic potential used. In the case of CID-Me, the "dynamics of adsorbed CO_2 is related to the rotational motion of ligands located in the narrow pore windows" [Kurihara *et al.*, 2022], an effect we are currently not able capture with the non-bonded Lennard-Jones potential and rigid framework methodology applied here.

To increase the level of clarity with respect to the strengths and limitations of the proposed methodology, we amended section S3.2.2 in the following way:

> *Our simulation results are generally of the same order of magnitude as the experimental benchmark, except for Zn_2(dobdc) and CID-Me. The former is overestimated by an order of magnitude, probably due to an incomplete treatment of the Zn open-metal site using a generic Lennard-Jones force field incapable of describing chemisorption. The latter is underestimated by several orders of magnitude, incorrectly classifying it as "confined diffusion".*

These two mismatches exemplify the limitations of the proposed methodology due to the choice of a non-bound, long-range classical potential. Although we are not able to experimentally validate all our simulations due to the scarcity of experimental data, the entries in Table S8 provide an example of the types of behavior encountered. In rigid, physisorbent materials we expect to obtain agreement of simulated and experimental diffusion coefficients. For chemisorbents and flexible structures, however, we expect to overestimate and underestimate diffusion coefficients, respectively. Nevertheless, the proposed methodology could be enhanced by replacing the Lennard-Jones interatomic potential by a more accurate one, keeping all other computational steps in place.

2. Filling columns in Industrial application need to constantly undergo changes in temperature and pressure gradients, so adsorbents must remain stable during multiple repetitive cycle. Stability testing, however, is a very time-consuming process. Did authors consider this aspect?

In our analysis, we did not consider the aspect of stability against cycling. As the scientific study limits the steps that can be included at this time, we agree such a step must be performed before a material can be applied at industrial scale. We note that a "complete" evaluation of carbon capture materials would be multi-dimensional and extremely complex. In our work, as in any scientific study, we had to limit the scope and leave certain screening dimensions unexplored. The contribution of the present manuscript is to include an often-neglected dimension in the performance evaluation, namely molecular diffusion, and demonstrate its effect on process-level performance simulation. We hope that, by shining light on an important factor that drives process-scale performance, we contribute to future efforts by helping identify which screening dimensions are worth including.

We included the following sentence in the last paragraph of the Discussion to point to possible extensions of our work.

Future work could include stability and synthesizability dimensions in the screening protocol.

3. For machine-learning process, the training data was extracted by fitting a model function to the GCMC-simulated adsorption isotherms instead of experimental data. Why? The most challenge for model training in machine learning is the accuracy of data. In my opinion, it will be more convincing to use experimental data for training.

We agree that the biggest challenge in model training is gathering large amounts of high-accuracy training data. While experimental data are, in principle, more trustworthy, they are not produced as abundantly as simulated data. Large aggregation of

experimental data obtained from different sources also raises significant concerns with regard to reproducibility and compatibility.

As an example, we consider the NIST/ARPA-E Database of Novel and Emerging Adsorbent Materials [Siderius *et al.*, 2015], which contains 9326 registered adsorbent materials. The database contains 3459 entries with experimental isotherm data which could, in principle, be used as training data for machine-learning models. However, it is very well documented in the literature that “for the well-studied case of CO_2 adsorption, there are only 15 of the thousands of known MOFs for which enough experiments have been reported to allow strong conclusions to be drawn about the reproducibility of these measurements” [Park *et al.*, 2017]. Had we used this database as source of training data, our model would have incorporated this uncertainty and lack of reproducibility, which is highly undesirable.

In the design of our work, we defined as a key objective the production of a large set of training data with well-defined, documented and reproducible conditions. The data were produced in a systematic fashion by performing automated simulations. For validation and reuse, we have shared data and simulation protocols openly with the community to allow for investigation, cross-validation and extensions. The simulation protocol, albeit limited in many ways, produces the volume and kind of reproducible data needed to train a machine-learning model. While our data are not experimental, we nevertheless believe they provide a rigorous base for studying the implications of molecular effects in process simulations.

4. As authors states in the manuscript “we suggest including micro-pore gas diffusivity, provided for each MOF structure through GCMC simulations, into the process simulation of CO_2 capture performance”, it should be MD simulations instead of GCMC simulations.

Thank you very much for capturing this inconsistency. In the revised version, we replaced “GCMC” by “MD”, as suggested.

Reviewer #5

The manuscript titled "Molecular Diffusion-Enhanced Performance Evaluation of Metal–Organic Frameworks for CO_2 Capture" introduces a workflow that employs molecular-level simulations and active learning techniques to evaluate the CoRE-MOF database for CO_2 capture. It clearly demonstrates the importance of material-dependent properties, such as the gas diffusion coefficient, when ranking the performance of MOFs. The work is thoroughly carried out and accompanied by sufficient details to substantiate the authors' claims and ensure reproducibility.

The authors thank the reviewer for the careful reading of the manuscript and for recognizing the significance of our work.

However, I have a few points that might need clarification:

1. How was the energy requirement plotted in Figures 4-6 obtained?

The Energy Requirement was obtained by solving the energy balance equations for the various phases and components, as described in Equation S6. The first paragraph of the Results section reads

> *Within the remaining 6,484 MOFs, we have identified those structures providing the highest productivity associated with the lowest energy requirement, as defined in Section S4, while achieving a purity of at least 95% as required for CO_2 capture applications at NGCC power plants.*

And then, in section S4, we write

> *The energy requirement is calculated by integrating the total energy input from external heating jacket and specific energy of steam used to purge N_2 and CO_2, during N_2 purge and CO_2 desorption steps, respectively. Our specific energy calculation does not include any additional contribution, including CO_2 compression, nor pump or blower energy.*

The detailed set of coupled equations S6, which is part of section S4, is copied below.

$$\frac{\partial T_g}{\partial t} = \frac{1}{\varepsilon \rho_g C_g}\left[\lambda_g \frac{\partial^2 T_g}{\partial z^2} - \varepsilon \rho_g C_g u_g \frac{\partial T_g}{\partial z} + \frac{6(1-\varepsilon)}{2R_p} h_{g,s}(T_s - T_g) + \frac{\pi d_{is} h \varepsilon}{V_s} h_{g,w}(T_w - T_g) + \frac{\pi d_{ot} h_{nT} \varepsilon}{V_s} h_{g,w} t\right] \quad \text{(S6)}$$

$$\frac{\partial T_s}{\partial t} = \frac{1}{(1-\varepsilon)\rho_s C_s}\left[\lambda_s \frac{\partial^2 T_s}{\partial z^2} - (1-\varepsilon)^2 \rho_s^2 C_s \sum_{i,k} \frac{\partial q_{i,k}}{\partial t} \Delta H_{i,k} + \frac{6(1-\varepsilon)}{2R_p} h_{g,s}(T_g - T_s) + \frac{\pi d_{is} h (1-\varepsilon)}{V_w} h_{s,w}(T_w - T_s)\right]$$

$$\frac{\partial T_w}{\partial t} = \frac{1}{\rho_w C_w}\left[\lambda_w \frac{\partial^2 T_w}{\partial z^2} + \frac{\pi d_{is} h \varepsilon}{V_w} h_{g,w}(T_g - T_w) + \frac{\pi d_{is} h (1-\varepsilon)}{V_w} h_{s,w}(T_s - T_w) + \frac{\pi d_{os} h}{V_w} h_{e,w}(T_e - T_w)\right]$$

$$\frac{\partial T_e}{\partial t} = \frac{1}{\rho_e C_e}\left[\lambda_e \frac{\partial^2 T_e}{\partial z^2} - \rho_e C_e u_e \frac{\partial T_e}{\partial z} + \frac{\pi d_{os} h}{V_e} h_{w,e}(T_w - T_e)\right]$$

2. The energy requirement depends on the heat capacity of the material, which in general varies with temperature. Does the energy requirement account for the structure – and temperature – dependent heat capacity? If not, a comment on the expected change in the distribution of the energy requirement may be beneficial. Authors may refer to https://doi.org/10.1038/s41563-022-01374-3 and https://doi.org/10.1103/PRXEnergy.2.023005 for materials-dependent heat capacity and how they affect heat/energy requirements.

Yes, we used the above-mentioned reference [Moosavi *et al.*, 2022] to compute material-dependent heat capacity. For every MOF in the data set, we used this machine learning model to calculate the heat capacity at 7 representative temperatures (250 – 400 K). We use this calculated heat capacity to fit a 4th order polynomial which is used in the process model to calculate temperature dependent heat capacity during the TSA cycle simulations.

To clarify this point, we have added the following paragraph to Section S4.1:

> *For each material, we used a machine-learning model [Moosavi et al., 2022] to calculate the heat capacity at seven representative temperature points, ranging from 250 K to 400 K. The calculated heat-capacity values were fit with a fourth-order polynomial, which is used in the process model for calculating temperature-dependent heat capacities during the TSA-cycle simulations.*

The paper is also cited by footnote c on Table S9.

Remarks on code availability

The GitHub repository includes a README file that provides a step-by-step guide on how to use the code.

We appreciate this notice. We have made an intentional effort to publish the code, enabling validation and reuse by the scientific community.

Reviewer #3

After my comments about the chemical correctness of the structures, the authors in the second round identified 224 materials as incorrect. They use the word 'bad' following the Woo groups description, but bad simply means these materials not MOFs or at least they are incorrect MOF structures. I think this is not a small change, and I am surprised to read that authors think that their general conclusion does not change after the removal of 224 MOFs among thousands. The figures clearly show the changes. I keep my original decision, the current work is not publishable, the study must be performed using the correct MOF database, removing wrong structures is not enough, please start with correct and corrected (not just remove) structures.

We thank reviewer # 3 for the assessment of the revised version of the manuscript.

As detailed in our previous response, the incorrect structures identified by MOSAEC did not significantly affect our main results. In their assessment of our work, reviewers #6 and #7 have arrived at the same conclusion.

Based on our observation that several structures were incorrectly labelled as “bad” by MOSAEC, we caution that structural check tools should not be considered to deliver “definitive proof” of incorrectness.

Reviewer #6

In my assessment, the authors have made a sincere and substantial effort to address the reviewer's concerns, and the central issue raised by Reviewer #3 has largely been resolved. While MOSAEC is a useful error checking tool in MOFs, it should not be treated as a definitive or exhaustive measure of chemical correctness. I do not think it is appropriate to conclude that all structures flagged by MOSAEC must necessarily be removed. Importantly, the authors have explicitly acknowledged this limitation and have demonstrated it by visualizing the structures flagged by MOSAEC, showing that these structures are in fact computational-ready. For additional check, authors are encouraged to use recently developed MOFClassifier (https://doi.org/10.1021/jacs.5c10126) which uses a PU-CGCNN method to classify CR and NCR MOFs for further validating their top MOFs.

The authors thank reviewer #6 for the assessment of the manuscript and the recommendation to publish it. We welcome the suggestion to incorporate MOFClassifier as an additional structural check tool. In the Discussion section of the revised version of the manuscript, we have included the following statement:

> *Future work should include structural checks with MOFClassifier[39] and address stability and synthesizability within the screening protocol.*

Some additional remarks

1. Partial atomic charges estimated from EQeq may not be the same as the partial atomic charges from DDEC06 and other DFT-derived charges, and it will impact CO2 isotherm which will impact the ranking of the materials. Authors are encouraged to provide the impact of using high-fidelity DDEC06 charges on the ranking of their "economically viable" materials. Some fast ML models have been developed to estimate the DFT-quality partial atomic charges, such as PACMOF and PACMAN.

The authors thank the reviewer for the suggestion.

In order to quantify the impact of the charge assignment scheme in the process-level rank ordering of MOFs, we re-ran end-to-end simulations for all (four) water-tolerant, economically-viable materials in Figure 6. We used a machine-learning based DDEC06 charge prediction model, namely PACMOF, to prepare CIF input files for our end-to-end workflow.

After re-running all molecular- to process-level simulations, following the same protocol as before, but using PACMOF- instead of EQeq-derived partial charges, we observe that despite small performance changes, all four materials remained "economically-viable" (as previously defined) and maintained their relative rank ordering. We re-calculated free energy-based water tolerance (ΔG_{H2O}) and confirmed

that two of the materials remained "water-tolerant" (as previously defined), with MIMVEJ displaying water affinity 6x lower than the industry benchmark CALF-20.

This lower sensitivity to the choice of charge assignment scheme resonates with findings published previously by some of the authors in

> Oliveira, F. L., Cleeton, C., Neumann Barros Ferreira, R., Luan, B., Farmahini, A. H., Sarkisov, L., & Steiner, M. (2023). CRAFTED: An exploratory database of simulated adsorption isotherms of metal-organic frameworks. Scientific Data, 10(1), 230.

In order to incorporate this feedback into the main manuscript, we added the following sentence to the Discussion.

> *To assess the impact of using EQeq charges, we re-ran the end-to-end simulation workflow for the (four) water-tolerant, economically-viable MOFs using DDEC06 charges computed with PACMOF[38]. Their relative rank order with regard to CO_2 capture performance remained unchanged. The refined water tolerance screening is provided in Supplementary Note 4.4.*

Furthermore, in the Supplementary Note 4.4, we provide additional evidence for the limited impact of switching the charge assignment scheme in this particular context.

> ***Impact of charge assignment scheme on the rank ordering of water-tolerant, economically-viable MOFs***
>
> *For quantifying the impact of the charge assignment scheme in the process-level rank ordering of MOFs, we re-ran end-to-end simulations for all four water-tolerant, economically-viable materials in Figure 6. We used PACMOF[48], a machine-learning based DDEC06 charge prediction model, to prepare CIF input files for our end-to-end workflow.*
>
> *We observe that despite small performance changes, all materials remained economically-viable and maintained their relative rank. We re-calculated the free energy-based water tolerance, ΔG_{H2O}, and confirmed that two of the materials remained water-tolerant, with MIMVEJ displaying a water affinity 6x lower than the industry benchmark CALF-20.*
>
> *The lower sensitivity to the choice of charge assignment scheme is in line with findings published previously by some of the authors[49].*

Supplementary Table 13. Comparison of CO_2 capture performance and H_2O tolerance metrics when using EQeq and PACMOF partial atomic charges. **Pr**oductivity (higher is preferable), **En**ergy **Re**quirement (lower is preferable) and free energy of H_2O adsorption (higher is preferable) for the four economically-viable, water-tolerant MOFs.

	EQeq			PACMOF		
	**Pr., kg/(day.L)**	**En. Re., MJ/kg**	**ΔG_{H_2O}, kJ/mol**	**Pr., kg/(day.L)**	**En. Re., MJ/kg**	**ΔG_{H_2O}, kJ/mol**
EXOFIH*	1.5	1.6	-8.0	1.5	1.4	-6.4
FAHBIC	1.5	2.9	-5.1	1.4	2.9	-20.8
FIPXAF	1.3	5.0	-5.6	1.1	4.8	-11.7
MIMVEJ	1.3	3.5	1.6	1.2	3.5	-1.0
CALF-20			-8.1			-5.6

2. There has been some work on CO2/N2 separation with integrated molecular simulation + process modeling on recent MOF DBs (https://doi.org/10.1016/j.matt.2025.102140) which may be included for references.

The authors confirm that the paper of Zhao *et al.* (2025) on CoRE-MOF-DB is already referenced in the main manuscript.

Reviewer #7

I read both the manuscript and the rebuttal letter from the authors. And while the validation of the computed diffusion coefficients is necessarily limited (only 6 MOF data) and some discrepancies with experiment remain substantial, I do not think this undermines the manuscript's central contribution. In my view, the work should be interpreted primarily as a high-throughput, internally consistent screening study showing that inclusion of structure-specific molecular diffusion can alter process-level conclusions. Given the scale of the screening and the lack of practical higher-accuracy alternatives for transport calculations across hundreds of MOFs, the chosen methodology appears reasonable for this purpose, even though the exact ranking of individual materials may vary with the underlying force field, charge model, and diffusivity formalism.

The authors thank reviewer #7 for the assessment of the manuscript and the recommendation to publish it.
